# Research ethics committee decision-making in relation to an efficient neonatal trial

**DOI:** 10.1136/archdischild-2016-310935

**Published:** 2016-09-14

**Authors:** C Gale, M J Hyde, N Modi

**Affiliations:** Section of Neonatal Medicine, Department of Medicine, Imperial College London, Chelsea and Westminster Hospital Campus, London, UK

**Keywords:** Ethics Committees, Research, Ethics, Research, Electronic Health Records, Informed Consent, Intensive Care, Neonatal

## Abstract

**Objective:**

Randomised controlled trials, a gold-standard approach to reduce uncertainties in clinical practice, are growing in cost and are often slow to recruit. We determined whether methodological approaches to facilitate large, efficient clinical trials were acceptable to UK research ethics committees (RECs).

**Design:**

We developed a protocol in collaboration with parents, for a comparative-effectiveness, randomised controlled trial comparing two widely used blood transfusion practices in preterm infants. We incorporated four approaches to improve recruitment and efficiency: (i) point-of-care design using electronic patient records for patient identification, randomisation and data acquisition, (ii) short two-page information sheet; (iii) explicit mention of possible inclusion benefit; (iv) opt-out consent with enrolment as the default. With the support of the UK Health Research Authority, we submitted an identical protocol to 12 UK REC.

**Setting:**

RECs in the UK.

**Main outcome:**

Number of REC granting favourable opinions.

**Results:**

The use of electronic patient records was acceptable to all RECs; one REC raised concerns about the short parent information sheet, 10 about inclusion benefit and 9 about opt-out consent. Following responses to queries, nine RECs granted a favourable final opinion and three rejected the application because they considered the opt-out consent process invalid.

**Conclusions:**

A majority of RECs in this study consider the use of electronic patient record data, short information sheets, opt-out consent and mention of possible inclusion benefit to be acceptable in neonatal comparative-effectiveness research. We identified a need for guidance for RECs in relation to opt-out consent processes. These methods provide opportunity to facilitate large randomised controlled trials.

**What is already known on this topic?**

- High-quality randomised controlled trials are the gold-standard way to reduce uncertainties in clinical care, but are increasingly expensive and burdensome.
- Evidence-based methods to make randomised controlled trials more efficient exist, but are seldom used in the UK.
- It is not known the degree to which regulation, such Research Ethics Committees review, contributes to inefficiency in randomised clinical trials.

**What this study adds?**

- The UK Research Ethics Committees find the use of electronic patient records, short participant information sheets and mention of inclusion benefit to be acceptable in neonatal comparative effectiveness research.
- There is inconsistency between the UK Research Ethics Committees in relation to the validity of opt-out consent processes for neonatal comparative effectiveness research.
- The wider application of these methods may facilitate larger, more efficient randomised controlled trials.

## Introduction

The advancement of patient care requires incremental reductions in the uncertainties found in routine practice, in addition to the development of new therapies. Rigorous randomised controlled trials are recognised as the gold-standard methodological approach to achieve these aims, but commonly require large sample sizes to detect clinically relevant effects. Concerns have been expressed about the rising financial burden and regulatory complexities of conventional randomised controlled trials. It has also been estimated that up to 85% of research investment is wasted,^1^ with examples including inefficient delivery and unduly restrictive regulation.^2^

Large trials require effective recruitment and efficient data acquisition.^3^ Methods to increase recruitment include informing participants of the potential benefits of enrolling in a clinical trial,^4^ consent processes that involve opportunity to opt out with enrolment as the default^5^ and short, simple participant information sheets.^6^ ^7^ The efficiency of data acquisition can be improved by using electronic patient records (EPRs) in what are referred to as ‘point-of-care trials’.^8^ However, despite evidence for the effectiveness of these approaches,^9^ ^10^ take-up has been limited and the extent to which regulatory barriers are contributory is unclear.

A key component of regulation is to ensure studies conform to agreed principles of research ethics. In many countries such as the UK, this is the responsibility of independent research ethics committees (RECs). The UK RECs were established in 1991 to review research conducted within the National Health Service (NHS). For multisite research, approval is sought from a single REC. The UK Health Research Authority (HRA) that has responsibility for RECs was established in 2011 to promote and protect the interests of patients in health research and streamline regulation.

We aimed to determine the acceptability to the UK RECs of methods to facilitate efficient, large, simple clinical trials. We tested the hypotheses that methods would be acceptable and decision-making consistent.

## Methods

The WithHolding Enteral feeds Around packed red cell Transfusion (WHEAT) trial is a multicentre, comparative effectiveness, point-of-care trial. We designed the WHEAT trial to address a clinical uncertainty identified as important by service users and clinicians in the field of preterm birth.^11^ The aim of the WHEAT trial is to compare two practices that are both in wide current clinical use (withholding enteral feeding or continuing normal feeding) during blood transfusion in preterm babies with the hope of reducing the incidence of necrotising enterocolitis (see box 1). WHEAT is a real trial being developed by an investigator group with the support of the UK neonatal clinical care community, has incorporated parent involvement from inception and is supported by a National Institute of Health Research Enabling Involvement grant.^12^

Box 1 **Summary of study submitted to Research Ethics Committees for the WithHolding Enteral feeds Around packed red cell Transfusion (WHEAT) study**

▸ The purpose of WHEAT is to compare two practices that are widely used in neonatal units across the UK and around the world to see if one reduces the risk of necrotising enterocolitis (NEC) in babies born early (premature).

▸ NEC is a serious gut disease that affects about 1 in 20 very premature babies (approximately 500 each year); about 1 in 3 of these babies will die of NEC and survivors often have long-term health and developmental problems. In 2014, prevention of NEC was ranked the third most important research priority by parents and perinatal health professionals.

▸ Premature babies receive frequent milk feeds (every 1 to 4 hours) and they often need blood transfusions because they become anaemic (they do not have enough red blood cells). Some doctors worry that feeding babies during a blood transfusion may increase the risk of NEC. Others, however, think that it is more dangerous to stop feeds. Because of this, the way babies are cared for during blood transfusions varies across the country; some babies have milk feeds stopped before, during and after a transfusion (around 12 hours in total), while others have feeds continued.

▸ The purpose of WHEAT is to determine which approach is best. We will do this by comparing babies who have feeds stopped with those who have feeds continued during blood transfusions. Whether feeds will be stopped or continued will be decided by randomisation. Randomisation is done by computer and ensures that each baby has an equal chance of receiving either approach. WHEAT will compare standard UK practices and involves nothing new. WHEAT will take place in neonatal units all over the UK and will involve about 4500 babies.

▸ Professional opinion (National Health Service Blood and Transplant, National Institute of Health Research Children's Clinical Research Network and most UK neonatal units) supports the need for a trial like WHEAT.

The WHEAT trial includes the following approaches to facilitate efficient data collection and recruitment:

1. A point-of-care trial design where all trial data are extracted from an existing national neonatal EPR via the National Neonatal Research Database (NNRD). In the UK, 430 clearly defined data items (the Neonatal Data Set—http://www.datadictionary.nhs.uk/data_dictionary/messages/clinical_data_sets/data_sets/national_neonatal_data_set/national_neonatal_data_set_-_episodic_and_daily_care_fr.asp?shownav=0) are extracted from a national summary EPR system to form the NNRD. Data protection complies with national standards, appropriate regulatory approvals are held and all parents are offered the opportunity to opt out of inclusion of their baby's data in the NNRD.
2. A short (2-page, 1000-word) parent information sheet (see online supplementary data 1).
3. Explicit mention in the patient information sheet of the possibility of inclusion benefit through participation: “Each of the two options in the WHEAT study is currently used by doctors in the UK because we do not know which one is better. For babies not taking part in the WHEAT study, the choice of whether or not to stop feeds is made according to the preference of the local medical team. This non-evidence based approach to neonatal care may involve more risk than being in a study like WHEAT which involves a carefully designed protocol and consistent monitoring”.
4. A consent process involving enrolment as the default unless a parent opts out. This was described as follows: “Parents of babies that meet the inclusion criteria will be approached by members of the local clinical team after their baby is admitted to the neonatal unit, usually in the first 24 hours. Parents will be provided with the parent information sheet that explains the WHEAT trial and makes it clear that both arms (withholding feeds and continuing feeds) represent current clinical practice. The parent information sheet will make it clear that opt-out consent is being requested. This means that if they do not want their baby to take part in the WHEAT trial, they can inform the local clinical team at any point. If they do not do ‘opt out’ in this way then their baby will be enrolled into WHEAT trial and will be randomised to one of the two study arms. The local team will record in the electronic health record that they have explained the WHEAT trial and the ‘opt-out’ consent process to parents.”

The lay ‘Summary of the study’ and the ‘Summary of the main [ethical] issues’ from the NHS REC form are shown in boxes 1 and 2, respectively. The full Integrated Research Application System (IRAS) form is provided in online supplementary appendix 2.

Box 2 **Summary of main (ethical) issues submitted to Research Ethics Committees for the WithHolding Enteral feeds Around packed red cell Transfusion (WHEAT) study**

Opt-out consent

▸ In present day practice, consent to either continue or withhold feeds is not sought from parents. In WHEAT, parents will be offered opportunity to opt-out of randomisation at any time and without having to give a reason. This reduces the risk of ‘injurious misconception’ where parents reject trial participation because of the burden of decision-making and an exaggerated perception of risk (Snowdon *et al*^13^). Full informed opt-out consent in the WHEAT trial will be a continuing process in which parents will be able to review their decision over the course of their relationship with neonatal unit staff. Parent opt-out will be recorded in the baby’s electronic health record.

▸ The opt-out approach is preferable because an opt-in approach is associated with a selection bias towards a healthier sample (Junghans *et al*^14^) thus reducing the generalisability of study outcomes.

▸ The opt-out approach has been used previously in neonatal research, particularly in comparative effectiveness research such as WHEAT where there is no research-related risk (as opposed to normal care-related risk).

Extracting trial data from routinely recorded clinical information

▸ The large sample size required to detect a clinically relevant effect on NEC would be expensive and challenging for standard trial methodology. WHEAT is a registry trial, this means that all trial data will be obtained from a database that holds routinely recorded clinical information, the National Neonatal Research Database (NNRD).

▸ All neonatal units in England and Wales contribute data to the NNRD, NNRD data are a National Health Service Information Standard (ISB1595) and are extracted quarterly from real-time neonatal electronic health records. The NNRD is approved by the Caldicott Guardians and Lead Clinicians of all contributing neonatal units, the National Research Ethics Service (10/H0803/151) and the Confidentiality Advisory Group of the Health Research Authority (805(f)/2010). The quality and completeness of data that will be used in WHEAT have been validated against clinical paper notes and clinical trial Case Report Forms and high levels of agreement have been shown. All parents of babies admitted to neonatal units are given an information sheet that explains the NNRD and its uses (service evaluations, audit and approved research) and that if they wish they may choose to opt out of clinical data entered into the electronic health records being held in the NNRD.

▸ Extracting trial data in this way will be more efficient than conventional, duplicative, data collection and will increase value and reduce waste in research (Chalmers I *et al*^15^).

▸ Use of the NNRD to support the trial has an additional fail-safe benefit in that a baby's data cannot be used for WHEAT unless there is a positive entry in the baby's electronic health record that the parent information sheet has been given and discussed with the parents; this entry will be signed by the person registering this on the system.

Inclusion benefit

▸ We have stated that there may be a benefit of participating in clinical trials in the patient information sheet as follows: “Each of the two options in the WHEAT study is currently used by doctors in the UK because we do not know which one is better. For babies not taking part in the WHEAT study, the choice of whether or not to stop feeds is made according to the preference of the local medical team. This non-evidence based approach to neonatal care may involve more risk than being in a study like WHEAT which involves a carefully designed protocol and consistent monitoring”. Patients enrolled in randomised controlled trials, including those allocated to the control arm, have better outcomes than comparable non-participants.

▸ This has been most recently demonstrated in a large neonatal trial (Carlo *et al*^16^), but the same benefit has been detected in previous neonatal (Schmidt *et al*^17^) and adult randomised trials (reviewed in Braunholtz *et al*^18^). We believe we have a duty to provide parents with clear information about both the benefits and the risks of research participation.

10.1136/fetalneonatal-2016-310935.supp1 Supplementary data

### Role of the HRA

We approached the HRA who agreed to assist with this study. All REC chairs in England were contacted by the HRA and invited to take part in a process that would involve an identical study submitted to multiple RECs for the purpose of comparing consistency, using the standard online submission process and without identifying that the application was part of a multiple submission.

Of the RECs that agreed to participate, the HRA identified those that did not share a common administrative coordinator, in order to reduce the likelihood of the study being identified as part of a multiple submission. From these, we selected all RECs for which it would be possible to attend meetings.

### REC application

We prepared a REC IRAS application (see online supplementary data 1), participant information sheet (see online supplementary data 2), study protocol, researcher CV, valid sponsor letter and insurance certificate (both from Imperial College London). The application was booked centrally by the HRA to the selected RECs; application documents were uploaded individually to each REC by the researchers. REC coordinators who recognised that an identical application had been submitted to multiple committees were contacted by the HRA and taken into confidence so that REC members remained unaware of the identity of the study.

10.1136/fetalneonatal-2016-310935.supp2 Supplementary data

Written responses to the RECs were standardised to ensure all committees received the same information upon which to base their opinion. Comments from individual REC and responses from the researchers are provided in online supplementary appendix 3. When challenged in relation to the four methodological approaches outlined above, the researchers responded by defending the ethical validity of the proposed approach and did not agree to its removal from the application. Where committees proposed other changes to the application, for example, requiring the information" sheet to include details of local Patient Advisory and Liaison Services, the researchers acceded to the REC suggestions (described in more detail in online supplementary data 3–14).

10.1136/fetalneonatal-2016-310935.supp3 Supplementary data

10.1136/fetalneonatal-2016-310935.supp4 Supplementary data

10.1136/fetalneonatal-2016-310935.supp5 Supplementary data

10.1136/fetalneonatal-2016-310935.supp6 Supplementary data

10.1136/fetalneonatal-2016-310935.supp7 Supplementary data

10.1136/fetalneonatal-2016-310935.supp8 Supplementary data

10.1136/fetalneonatal-2016-310935.supp9 Supplementary data

10.1136/fetalneonatal-2016-310935.supp10 Supplementary data

10.1136/fetalneonatal-2016-310935.supp11 Supplementary data

10.1136/fetalneonatal-2016-310935.supp12 Supplementary data

10.1136/fetalneonatal-2016-310935.supp13 Supplementary data

10.1136/fetalneonatal-2016-310935.supp14 Supplementary data

It was decided in advance that the REC decisions arising from this study would not be used as formal REC approval for the WHEAT trial. REC approval following a future application, to a single REC, would be required before starting the WHEAT trial.

### Study outcomes

The primary outcome was the number of RECs granting a final favourable opinion.

Secondary outcomes were:

- the number of RECs that raised queries regarding each of the four approaches proposed to increase trial efficiency;
- process metrics: time to decision from submission of the application, measured using the HRA methodology (in which the ‘clock’ stops while response is being prepared by the researchers);
- amount of correspondence required to reach a final REC decision.

## Results

Eighty-eight UK RECs were asked by the HRA to take part in a blinded, multiple submission exercise, in advance of the study. Twenty-seven of 88 agreed to participate, no reasons were given by the 61 RECs that chose not to participate. Six of the 27 RECs that agreed to participate were flagged to receive paediatric studies. Flagged RECs have specific expertise in reviewing a particular type of study, in this case research involving children. Six RECs meetings were attended by either MJH or CG in person and they were available by telephone for the other six. Four RECs telephoned MJH during their meeting.

MJH attended two meetings and both MJH and CG attended four meetings. One REC asked the researchers to attend on two occasions, with the second meeting in order to discuss applicant responses to the decision letter issued by the RECs after the first meeting. The first of these was attended by MJH and the follow-up meeting was attended by both MJH and CG.

Four RECs coordinators recognised that an identical application had been submitted to multiple committees and were informed in confidence of the study by the HRA.

One REC rejected the application outright without correspondence with the researchers. Eleven RECs required written responses from the researchers, of these nine provided a final favourable opinion and two rejected the application.

Eleven RECs asked for study documents to be amended (median 2 documents; range 1–7). We provided written responses comprising a median of 3 (range 1–13) pages and 941 words (range 164–5227). The median time from REC meeting to final decision was 14 working days (range 4–33).

### REC comments on the four methodological aspects under study

Details of the responses received relating to the proposed methodological approaches are listed in table 1. No REC raised concerns about the proposed point-of-care design using the EPR data. One REC raised concerns about the short parent information sheet. The committee initially requested “The participant information sheet be re-written/re-formatted in line with [HRA] guidance [for Clinical Trials of Investigational Medical Products]”. In correspondence, we explained that HRA guidance is not mandatory, does not fit all research and that there is evidence of a negative correlation between increasing length of parent information sheet and participants’ understanding of the research; we also demonstrated parent involvement in the design of the information sheet. The short parent information sheet was subsequently accepted by this REC.

**Table 1:** Summary of responses received from individual Research Ethics Committees

Research Ethics Committee	Paediatric flagged	Attended in person	Discussed on phone	Decision	Opt-out consent	Streamlined PIS	Inclusion benefit	Electronic healthcare data
1	Yes	No	No	Accept, with modifications	No concerns raised	No concerns raised	Initial judgement: “In the section ‘Are there any benefits for my baby’ please delete the third sentence starting ‘This non-evidence based approach …’” Subsequently accepted modified wording	No concerns raised
2	Yes	Yes	No	Accept, with modifications	No concerns raised	No concerns raised	Initial judgement: “Please amend the sentence ‘This non-evidence based approach to neonatal care may involve more risk than being in a study like WHEAT which involves a carefully designed protocol and consistent monitoring’ under the heading ‘Are there any benefits for my baby?’ as it could be considered coercive”. Subsequently accepted modified wording	No concerns raised
3	Yes	Yes	No	Accept, with modifications	Required the following to be added to the protocol “The person conducting the consent discussion should provide a clear, well written documentation in patient paper notes to document the entire consent process and clarifying the option taken (opt in/ opt out) by the parents”	No concerns raised	No concerns raised	No concerns raised
4	No	Yes	No	Reject—after further discussion	Judgement: “The REC remained unhappy with an opt-out process. They discussed the possibility of providing a consent form for parents to evidence they had opted out, but on reflection agreed if this was possible it should be possible to provide an opt-in consent form and change the process accordingly. … The Committee agreed it was essential parents were asked to sign an agreement (either opt-out or opt-in) to ensure they personally understood exactly what they were consenting their children to. The Committee felt the concept of an opt-out study would indicate to parents there was no risk involved in the study, which would influence their decision to participate. … They acknowledged there were justifications for opt-out studies, but did not believe they applied in this case. The REC require the consent process to be changed to an ‘opt in’ system, and a consent form provided for review. This should be provided to parents alongside the information sheet, and the protocol updated to reflect this”	Initial judgement: “The participant information sheet should be rewritten/reformatted in line with NRES guidance, to ensure all pertinent areas are covered”. Having demonstrated that there was nothing absent in the PIS that was suggested by NRES guidelines, abbreviated PIS was accepted	Initial judgement: “The sentence within the ‘Are there any benefits for my baby?’ section stating ‘This non evidence based approach to neonatal care may involve more risk than being in a study like WHEAT which involves a carefully designed protocol and consistent monitoring’ must be removed. Note: Giving this kind of information to potential participants by inclusion in a general information leaflet about research is considered to be reasonable, though the particular wording should be reviewed within the institution guidelines” Subsequently accepted modified wording	No concerns raised
5	No	No	Yes	Accept, with modifications	Initial judgement: “The Committee requests further justification for the proposed use of opt-out consent”. AND “The Committee requests clarification of the process should a parent decide to opt out of the study, and confirmation that no undue pressure or influence would be placed on them, even if unintentionally (for example, by asking them to sign a form to opt out, when this is not required at the time of initial consent)”. Subsequently accepted opt-out consent with condition we provided parents with a card to prove their participation and explaining the opt-out process	No concerns raised	Initial judgement: “Please remove the final sentence of the section ‘Are there any benefits for my baby?’. It is disingenuous to include this statement about “non-evidence based approach” in the PIS since evidence based care simply means care that is compatible with the current state of evidence”. Subsequently accepted modified wording	No concerns raised
6	Yes	No	Yes	Accept, with modifications	No concerns raised	No concerns raised	Initial judgement: “Removal of the following sentence in the Participant Information Sheet: “This non-evidence based approach to neonatal care may involve more risk than being in a study like WHEAT which involves a carefully designed protocol and consistent monitoring”. Subsequently accepted modified wording	No concerns raised
7	Yes	Yes	No	Reject—after further discussion	Initial judgement: “The Committee decided that, as there is an opportunity to do so, consent should be sought from parents. The design should be changed from opt out to opt in. Therefore, please submit a consent form for completion by parents”	No concerns raised	Initial judgement: “Please remove the last sentence from the section headed Are there any benefits for my baby? In the Participant Information Sheet”. Subsequently accepted modified wording	No concerns raised
8	No	No	Yes	Reject	“The Committee did not accept that it was appropriate for patients to be entered into this study without prior consent from parents. The ‘opt-out’ consent is not a concept that the Committee recognises; it is recruitment without consent. This raised many ethical issues which have not been addressed. The Committee also considered that written evidence of consent would protect researchers from future action by parents or authorities in the event of adverse outcomes, which are not rare in this very vulnerable patient population”	No concerns raised	“The Committee thought the Parent Information Sheet was coercive in places, stating it was better to be in the study than not”	No concerns raised
9	No	Yes	No	Accept, with modifications	No concerns raised	No concerns raised	Initial judgement: “Are there any Benefits…? Section—delete the last sentence”. Subsequently accepted modified wording	No concerns raised
10	No	Yes	No	Accept, with modifications	Initial judgement: “An opt out form should be included for parents to keep for their own records”	No concerns raised	No concerns raised	No concerns raised
11	Yes	Yes	No	Accept, with modifications	Initial judgement: “Please formally document the parents’ decision to not opt out of the study”. Subsequently accepted recording this in the electronic health record	No concerns raised	Initial judgement: “Please remove the last sentence of the ‘Are there any benefits to my baby?’ Section of the Patient Information and replace with ‘taking part in a research study may confer non-specific benefits’”. Accepted modified wording	No concerns raised
12		No	No	Accept, with modifications	Initial judgement: “The ‘opt out’ option is mentioned in the study does not seem to be ethical. With the ‘opt out’ there is always a subtle push towards taking part in the study. This should be changed to ‘opt in’ as usual”. Subsequently accepted, after committee were presented with further data supporting the use of opt-out consent in this setting.	“PIS as presented is bit complex and long, especially for parents in this situation. PIS could be made brief and be simplified with lay reader friendly language”. Subsequently accepted after demonstrating parent involvement in drafting	Initial judgement: “Since there are no real benefits for babies or parents for taking part in the study, any reference to benefits of taking part should be removed from the PIS, as it could be undue persuasion”. Subsequently accepted modified wording	No concerns raised

NRES, National Research Ethics Service; PIS, Patient Information Sheet; REC, Research Ethics Committee; WHEAT, WithHolding Enteral feeds Around packed red cell Transfusion.

All nine RECs that provided a favourable opinion raised concerns about the mention of inclusion benefit in the patient information sheet. Following correspondence, the following modified statement was considered acceptable and included in the patient information sheet approved by all RECs:

Each of the two options in the WHEAT study is currently used by doctors in the UK because we do not know which one is better. For babies not taking part in the WHEAT study, the choice of whether or not to stop feeds is made according to the preference of the local medical team. Taking part in a research study may confer non-specific benefits.

Three RECs provided an unfavourable opinion because they considered opt-out consent invalid. One committee accepted that while there were justifications for an opt-out approach these did not apply in relation to the WHEAT study (further explanation not provided). Two other RECs indicated they considered this approach to consent to be more universally invalid. One committee stated “as there is an opportunity to do so, [opt in] consent should be sought from parents”, while another indicated that “the ‘opt-out’ consent is not a concept that the Committee recognises” deeming it to be ‘recruitment without consent’*.* The remaining nine RECs provided a favourable opinion for the WHEAT trial incorporating opt-out consent.

At the end of the study, we produced a feedback document for RECs (see online supplementary data 15). This was delivered by the HRA to all the UK REC chairs and was accompanied by a response from the HRA (see online supplementary data 16).

10.1136/fetalneonatal-2016-310935.supp15 Supplementary data

10.1136/fetalneonatal-2016-310935.supp16 Supplementary data

## Discussion

We show that using EPR data in neonatal trials, short participant information sheets, opt out consent and explicitly mentioning inclusion benefit are acceptable to a majority of the NHS RECs included in this study. We identify inconsistency between RECs in relation to whether ‘opt-out’ consent is ethically valid, with 3 of 12 RECs rejecting our application on these grounds. We also show that the time taken by the UK NHS RECs to reach a final opinion was considerably less than the 60-day target set by the HRA.

Strengths include blinding of RECs to the comparative nature of the study, a priori defined outcomes and the use of a genuine clinical trial developed with extensive parent and multidisciplinary input. To our knowledge, this is the first blinded attempt to examine the consistency of the UK REC decision-making since the introduction in 2004 of a single UK-wide ethics application process. Previously, the Research Ethics Service has used an internal exercise known as *shared ethical debate* to examine consistency. This approach is limited because the committee is aware that it is part of an exercise to check consistency (and not a ‘real’ application) and may therefore subject it to greater scrutiny than normal, or conversely conduct only cursory discussions and reach a decision without the same degree of scrutiny exercised for ‘real’ applications. To date, none of these internal exercises has been published in the peer-reviewed literature. Additionally, *shared ethical debates* do not involve discussion with researchers, an important consideration as many issues raised by applications are addressed in dialogue at committee meetings or in correspondence. The key limitations of our study are the uncertain generalisability of our findings to other patient groups and beyond comparative effectiveness trials of commonly used treatments.

Inconsistencies in REC decision-making are long-recognised. The HRA has proposed a number of initiatives to make decisions more consistent.^19^ These include greater engagement with researchers and a formal mechanism for capturing and sharing past decisions in relation to specific principles. We urge the HRA to continue to monitor consistency, evaluate the effectiveness of these initiatives and create a searchable repository of decisions.

Stakeholders (including parents, parent representatives and health professionals) were involved throughout the development of WHEAT, and specifically considered opt-out consent to be valid and appropriate. This is in keeping with national^20^ and international^21^ guidance for comparative effectiveness research. Although most RECs considered and incorporated this stakeholder involvement when reaching their decision, it is concerning that three RECs appeared to give less weight to the views of stakeholders, rejecting our application on the ground of opt-out consent. While opt-out consent is unlikely to be appropriate for studies where there is risk from exposure to non-routine or novel experimental agents or practices, it may actually be preferable for comparative effectiveness research as it results in higher recruitment and a less biased sample.^22^ In determining the ethically appropriate limits for opt-out consent, it is essential to involve patients, public and frontline health professionals, in addition to academics and ethicists. This may be better served through a case-by-case approach, rather than more rigid ‘top-down’ guidance.

Inclusion benefit is well described in the context of neonatal randomised controlled trials,^4^ ^23^ most recently in the SUPPORT trial^24^ where infants in both treatment groups had lower rates of death than similar infants not enrolled in the trial. The Declaration of Helsinki states that “each potential subject must be adequately informed of the… anticipated benefits and potential risks of the study”.^25^ We hold that informing research participants of potential benefits is as essential as discussing the risk of harm. We accept that the evidence for inclusion benefit is controversial, in particular in relation to point-of-care comparative effectiveness trials that aim for high participation rates. We suggest that, where possible, clinical trials should endeavour to collect aggregate anonymised primary outcome data from comparable patient groups not enrolled in the trial to inform future discussion about the potential harms and benefits of research participation.

Using routinely available EPR data in *point-of-care trials* or *randomised registry trials* has substantial potential to increase efficiency.^26^ Such trials are ongoing in the UK (http://www.nets.nihr.ac.uk/projects/hta/1449127) and internationally.^27^ It is reassuring that no REC raised concerns about this approach given the potential to reduce research waste across many medical disciplines.

In many countries, researchers are required to obtain independent ethics review at each participating site for a multicentre study. This adds considerable administrative burden and also increases the likelihood of inconsistency in research ethics review^28–30^ that is largely hidden in the UK by the single approval system. Our study in conjunction with international evidence indicates that inconsistency in decision-making is commonplace across countries and healthcare systems; this serves neither patients nor researchers well and risks breakdowns in trust. This highlights the importance of sound policies to improve REC consistency.

Our study indicates that where an REC raises concerns that a researcher feels are unjustified, a clear written defence explaining the ethical basis, citing supporting precedent and demonstrating meaningful patient-public involvement is warranted. If this still results in an unfavourable opinion, we suggest resubmission to another REC including detailed justification of the approach adopted, in accordance with the HRA guidance.

Well-designed and appropriately powered clinical trials are essential to resolve uncertainty. Such trials require effective recruitment and data collection. It is reassuring that the majority of the UK RECs consider all four proposed approaches, namely point-of-care trial designs using the EPR data, short patient information sheets, recognition of the possibility of inclusion benefit and opt-out consent for comparative effectiveness research to be valid. We suggest that these approaches should be applied more widely to facilitate large, simple trials, reduce research waste and speed reductions in uncertainties in care.
