# Supplementary material for: Research ethics committee decision-making in relation to an efficient neonatal trial
Source: Arch Dis Child Fetal Neonatal Ed. 2016 Sep 14;102(4):F291–8. doi: 10.1136/archdischild-2016-310935 (PMC5537508; doi:10.1136/archdischild-2016-310935)
Supplement: Supplementary data [file fetalneonatal-2016-310935supp001.pdf]

Welcome to the Integrated Research Application System

IRAS Project Filter

The integrated dataset required for your project will be created from the answers you give to the following questions. The system will generate only those questions and sections which (a) apply to your study type and (b) are required by the bodies reviewing your study. Please ensure you answer all the questions before proceeding with your applications.

Please enter a short title for this project (maximum 70 characters)
WHEAT: WithHolding Enteral feeds Around packed red cell Transfusion

1. Is your project research?

(•) Yes ( ) No

2. Select one category from the list below:

( ) Clinical trial of an investigational medicinal product
( ) Clinical investigation or other study of a medical device
( ) Combined trial of an investigational medicinal product and an investigational medical device
(•) Other clinical trial to study a novel intervention or randomised clinical trial to compare interventions in clinical practice
( ) Basic science study involving procedures with human participants
( ) Study administering questionnaires/interviews for quantitative analysis, or using mixed quantitative/qualitative methodology
( ) Study involving qualitative methods only
( ) Study limited to working with human tissue samples (or other human biological samples) and data (specific project only)
( ) Study limited to working with data (specific project only)
( ) Research tissue bank
( ) Research database

If your work does not fit any of these categories, select the option below:

( ) Other study

2a. Will the study involve the use of any medical device without a CE Mark, or a CE marked device which has been modified or will be used outside its intended purposes?

( ) Yes (•) No

2b. Please answer the following question(s):

a) Does the study involve the use of any ionising radiation?	( ) Yes	(•) No
b) Will you be taking new human tissue samples (or other human biological samples)?	( ) Yes	(•) No
c) Will you be using existing human tissue samples (or other human biological samples)?	( ) Yes	(•) No

3. In which countries of the UK will the research sites be located?*(Tick all that apply)*

[x] England

- [ ] Scotland
- [x] Wales
- [ ] Northern Ireland

3a. In which country of the UK will the lead NHS R&D office be located:

- (•) England
- ( ) Scotland
- ( ) Wales
- ( ) Northern Ireland
- ( ) This study does not involve the NHS

4. Which review bodies are you applying to?

- [x] NHS/HSC Research and Development offices
- [ ] Social Care Research Ethics Committee
- [x] Research Ethics Committee
- [ ] National Information Governance Board for Health and Social Care (NIGB)
- [ ] National Offender Management Service (NOMS) (Prisons & Probation)

For NHS/HSC R&D offices, the CI must create Site-Specific Information Forms for each site, in addition to the study-wide forms, and transfer them to the PIs or local collaborators.

5. Will any research sites in this study be NHS organisations?

(•) Yes ( ) No

5a. Are all the research costs and infrastructure costs for this study provided by an NIHR Biomedical Research Centre, NIHR Biomedical Research Unit, NIHR Collaboration for Leadership in Health Research and Care (CLAHRC) or NIHR Research Centre for Patient Safety & Service Quality in all study sites?

( ) Yes (•) No

If yes, NHS permission for your study will be processed through the NIHR Coordinated System for gaining NHS Permission (NIHR CSP).

5b. Do you wish to make an application for the study to be considered for NIHR Clinical Research Network (CRN) support and inclusion in the NIHR Clinical Research Network (CRN) Portfolio? Please see information button for further details.

(•) Yes ( ) No

If yes, NHS permission for your study will be processed through the NIHR Coordinated System for gaining NHS Permission (NIHR CSP) and you must complete a NIHR Clinical Research Network (CRN) Portfolio Application Form immediately after completing this project filter and before completing and submitting other applications.

6. Do you plan to include any participants who are children?

(•) Yes ( ) No

7. Do you plan at any stage of the project to undertake intrusive research involving adults lacking capacity to consent for themselves?

( ) Yes (•) No

Answer Yes if you plan to recruit living participants aged 16 or over who lack capacity, or to retain them in the study following loss of capacity. Intrusive research means any research with the living requiring consent in law. This includes use of identifiable tissue samples or personal information, except where application is being made to the NIGB Ethics and

Confidentiality Committee to set aside the common law duty of confidentiality in England and Wales. Please consult the guidance notes for further information on the legal frameworks for research involving adults lacking capacity in the UK.

8. Do you plan to include any participants who are prisoners or young offenders in the custody of HM Prison Service or who are offenders supervised by the probation service in England or Wales?

○ Yes ◉ No

9. Is the study or any part of it being undertaken as an educational project?

○ Yes ◉ No

10. Will this research be financially supported by the United States Department of Health and Human Services or any of its divisions, agencies or programs?

○ Yes ◉ No

11. Will identifiable patient data be accessed outside the care team without prior consent at any stage of the project (including identification of potential participants)?

○ Yes ◉ No

Integrated Research Application System
Application Form for Other clinical trial or investigation

The Chief Investigator should complete this form. Guidance on the questions is available wherever you see this symbol displayed. We recommend reading the guidance first. The complete guidance and a glossary are available by selecting Help.

Please define any terms or acronyms that might not be familar to lay reviewers of the application.

Short title and version number: (maximum 70 characters - this will be inserted as header on all forms)
WHEAT: WithHolding Enteral feeds Around packed red cell Transfusion

PART A: Core study information

1. ADMINISTRATIVE DETAILS

A1. Full title of the research:

The WHEAT trial: WithHolding Enteral feeding Around packed red cell Transfusions in preterm neonates, a multicentre, superiority, randomised registry trial

A3-1. Chief Investigator:

	Title	Forename/Initials	Surname
	Dr	Chris	Gale

Post	NIHR Clinical Lecturer
Qualifications	MBBS, MSc, PhD, DIC
Employer	Imperial College London
Work Address	Section of Academic Neonatal Medicine,<br>Imperial College London, Chelsea and Westminster Campus,<br>369 Fulham Road, London
Post Code	SW10 9NH
Work E-mail	christopher.gale@imperial.ac.uk
* Personal E-mail	
Work Telephone	02033153519
* Personal Telephone/Mobile	
Fax	

** This information is optional. It will not be placed in the public domain or disclosed to any other third party without prior consent.*
A copy of a current CV (maximum 2 pages of A4) for the Chief Investigator must be submitted with the application.

A4. Who is the contact on behalf of the sponsor for all correspondence relating to applications for this project?
This contact will receive copies of all correspondence from REC and R&D reviewers that is sent to the CI.

	Title	Forename/Initials	Surname
	Mi		

Address

Post Code
E-mail
Telephone
Fax

A5-1. Research reference numbers. *Please give any relevant references for your study:*

Applicant's/organisation's own reference number, e.g. R & D (if available):	
Sponsor's/protocol number:	
Protocol Version:	1.3
Protocol Date:	11/08/2014
Funder's reference number:	
Project website:	

Registry reference number(s):
The Department of Health's Research Governance Framework for Health and Social Care and the research governance frameworks for Wales, Scotland and Northern Ireland set out the requirement for registration of trials. Furthermore: Article 19 of the World Medical Association Declaration of Helsinki adopted in 2008 states that "every clinical trial must be registered on a publicly accessible database before recruitment of the first subject"; and the International Committee of Medical Journal Editors (ICMJE) will consider a clinical trial for publication only if it has been registered in an appropriate registry. Please see guidance for more information.

International Standard Randomised Controlled Trial Number (ISRCTN):
ClinicalTrials.gov Identifier (NCT number):

Additional reference number(s):

Ref.Number Description	Reference Number

A5-2. Is this application linked to a previous study or another current application?

○ Yes ◉ No

Please give brief details and reference numbers.

2. OVERVIEW OF THE RESEARCH

To provide all the information required by review bodies and research information systems, we ask a number of specific questions. This section invites you to give an overview using language comprehensible to lay reviewers and members of the public. Please read the guidance notes for advice on this section.

A6-1. Summary of the study. *Please provide a brief summary of the research (maximum 300 words) using language easily understood by lay reviewers and members of the public. Where the research is reviewed by a REC within the UK Health Departments Research Ethics Service, this summary will be published on the website of the National Research Ethics Service following the ethical review.*

The purpose of WHEAT is to compare two practices that are widely used in neonatal units across the UK and around the world to see if one reduces the risk of necrotising enterocolitis (NEC) in babies born early (premature).

NEC is a serious gut disease that affects about 1 in 20 very premature babies (approximately 500 each year); about 1 in 3 of these babies will die of NEC and survivors often have long-term health and developmental problems. In 2014 prevention of NEC was ranked the third most important research priority by parents and perinatal health professionals.

Premature babies receive frequent milk feeds (every 1-3 hours) and they often need blood transfusions because they

become anaemic (they do not have enough red blood cells). Some doctors worry that feeding babies during a blood transfusion may increase the risk of NEC. Others, however, think that it is more dangerous to stop feeds. Because of this, the way babies are cared for during blood transfusions varies across the country; some babies have milk feeds stopped before, during and after a transfusion (around 12 hours in total) while others have feeds continued.

The purpose of WHEAT is to determine which approach is best. We will do this by comparing babies who have feeds stopped with those who have feeds continued during blood transfusions. Whether feeds will be stopped or continued will be decided by randomisation. Randomisation is done by computer and ensures that each baby has an equal chance of receiving either approach. WHEAT will compare standard UK practices and involves nothing new. WHEAT will take place in neonatal units all over the UK and will involve about 4,500 babies.

Professional opinion (NHS Blood and Transplant, NIHR Children's Clinical Research Network and most UK neonatal units) supports the need for a trial like WHEAT.

A6-2. Summary of main issues. *Please summarise the main ethical, legal, or management issues arising from your study and say how you have addressed them.*

Not all studies raise significant issues. Some studies may have straightforward ethical or other issues that can be identified and managed routinely. Others may present significant issues requiring further consideration by a REC, R&D office or other review body (as appropriate to the issue). Studies that present a minimal risk to participants may raise complex organisational or legal issues. You should try to consider all the types of issues that the different reviewers may need to consider.

The main issues arising from the WHEAT trial are:
1. RANDOMISATION IN A COMPARATIVE EFFECTIVENESS STUDY
- WHEAT is a comparative effectiveness study: the treatment strategies that will be compared are both considered "standard of care" and used in neonatal units across the UK and worldwide; a recent survey (Power et al., Arch Dis Child 2014) showed that approximately 1 in 3 neonatal units in the UK currently withhold feeds around blood transfusion while 2 in 3 neonatal units continue feeds. The strategy that confers clinical benefit is unknown (Keir et al., Arch Dis Child 2013) and so neonatal clinicians are in equipoise about withholding or continuing feeds during blood transfusion. We carried out a national survey of neonatal units in 2014, 97 neonatal units (87% of survey responders) would be willing to randomised babies in a trial like WHEAT.
- In present day practice the choice of treatment strategy is based upon non-evidenced clinician preference. In contrast, in WHEAT the choice of treatment strategy will be made by randomisation, a technique that ensures every baby has a fair and equal chance of allocation to either strategy. WHEAT will resolve an important uncertainty in care and will meet the NHS Constitution Principle 3, to "use research to improve the current and future health and care of the population".

2. OPT-OUT CONSENT
- In present day practice consent to either continue or withhold feeds is not sought from parents. In WHEAT parents will be offered opportunity to opt-out of randomisation at any time and without having to give a reason. This reduces the risk of "injurious misconception" where parents reject trial participation because of the burden of decision making and an exaggerated perception of risk (Snowdon et al., Clinical Ethics 2007). Full informed opt-out consent in the WHEAT trial will be a continuing process in which parents will be able to review their decision over the course of their relationship with neonatal unit staff. Parent opt-out will be recorded in the baby's electronic health record.
- The "opt-out approach is preferable because an "opt-in" approach is associated with a selection bias towards a healthier sample (Junghans C et al., BMJ 2005) thus reducing the generalisability of study outcomes.
- The "opt-out" approach has been used previously in neonatal research particularly in comparative effectiveness research such as WHEAT where there is no research-related risk (as opposed to normal care related risk).

3. EXTRACTING TRIAL DATA FROM ROUTINELY RECORDED CLINICAL INFORMATION
- The large sample size required to detect a clinically relevant effect on NEC would be expensive and challenging for standard trial methodology. WHEAT is a registry trial, this means that all trial data will be obtained from a database that holds routinely recorded clinical information, the National Neonatal Research Database (NNRD).
- All neonatal units in England and Wales contribute data to the NNRD, NNRD data are an NHS Information Standard (ISB1595) and are extracted quarterly from real-time neonatal Electronic Health Records. The NNRD is approved by the Caldicott Guardians and Lead Clinicians of all contributing neonatal units, the National Research Ethics Service (10/H0803/151) and the Confidentiality Advisory Group of the Health Research Authority (805(f)/2010). The quality and completeness of data that will be used in WHEAT have been validated against clinical paper notes and clinical trial Case Report Forms (CRF) and high levels of agreement have been shown. All parents of babies admitted to neonatal units are given an information sheet that explains the NNRD and its uses (service evaluations, audit and approved research) and that if they wish they may choose to opt-out of clinical data entered into the Electronic Health Records being held in the NNRD.
- Extracting trial data in this way will be more efficient than conventional, duplicative, data collection and will increase

value and reduce waste in research (Chalmers I et al., Lancet 2014).
- Use of the NNRD to support the trial has an additional fail-safe benefit in that a baby's data cannot be used for WHEAT unless there is a positive entry in the baby's Electronic Health Record that the parent information sheet has been given and discussed with the parents, this entry will be e-signed by the person registering this on the system.

4. TWINS AND MULTIPLE BIRTHS
Around a third of preterm babies admitted to neonatal care are part of a multiple birth set. It is generally considered preferable to randomise multiple births individually, thus recognising their individual right to the fair test of randomisation. Despite this in WHEAT we have elected to randomise all babies that are part of the same multiple birth set (e.g. both twins) to the same intervention. As outcomes are correlated within multiple birth sets this means that more babies need to be recruited to achieve a sample size of sufficient power. This approach has been chosen because the view of the parent member of the WHEAT trial development group (HR) and parent advocate groups (TAMBA - Twins And Multiple Birth Association; Bliss, the national charity for babies born "too early, too small, too sick") is that twins should be randomised to the same intervention group in un-blinded studies such as WHEAT.

5. INCLUSION BENEFIT
We have stated that there may be a benefit of participating in clinical trials in the patient information sheet as follows:

"Each of the two options in the WHEAT study is currently used by doctors in the UK because we do not know which one is better. For babies not taking part in the WHEAT study, the choice of whether or not to stop feeds is made according to the preference of the local medical team.
This non-evidence based approach to neonatal care may involve more risk than being in a study like WHEAT which involves a carefully designed protocol and consistent monitoring."

Patients enrolled in randomised controlled trials, including those allocated to the control arm, have better outcomes than comparable non-participants. This has been most recently demonstrated in a large neonatal trial (Carlo et al., 2013 NEJM), but the same benefit has been detected in previous neonatal (Schmidt et al., J Perinatol 1999) and adult randomised trials (reviewed in Braunholtz et al., J Clin Epidemiol 2001). We believe we have a duty to provide parents with clear information about both the benefits and the risks of research participation.

3. PURPOSE AND DESIGN OF THE RESEARCH

A7. Select the appropriate methodology description for this research. *Please tick all that apply*:

- [ ] Case series/ case note review
- [ ] Case control
- [ ] Cohort observation
- [ ] Controlled trial without randomisation
- [ ] Cross-sectional study
- [ ] Database analysis
- [ ] Epidemiology
- [ ] Feasibility/ pilot study
- [ ] Laboratory study
- [ ] Metanalysis
- [ ] Qualitative research
- [ ] Questionnaire, interview or observation study
- [x] Randomised controlled trial
- [ ] Other (please specify)

A10. What is the principal research question/objective? *Please put this in language comprehensible to a lay person.*

In babies born <30 weeks gestation (Patient) does withholding milk feeds before, during and immediately after a blood transfusion (Intervention), compared to continued feeding (Control), reduce the incidence of severe necrotising enterocolitis (Outcome)?

A11. What are the secondary research questions/objectives if applicable? *Please put this in language comprehensible to*

a lay person.

In babies born <30 weeks gestation does withholding milk feeds before, during and immediately after a blood transfusion, compared to continued feeding (Control), reduce the incidence of:
1. Necrotising enterocolitis of lesser severity
2. Death before discharge from the neonatal unit

In babies born <30 weeks gestation does withholding feeds before, during and immediately after a blood transfusion, compared to continued feeding (Control), reduce the length of time babies stay on the neonatal unit before going home.

A12. What is the scientific justification for the research? *Please put this in language comprehensible to a lay person.*

IMPORTANCE: Necrotising enterocolitis (NEC) is a feared disease that affects 5-10% of very preterm infants (about 500 babies annually); over 100 babies die from NEC in England each year and 45% of survivors suffer neurodevelopmental impairment. NEC incurs sizable healthcare costs: NEC that requires surgery increases length of stay by over 6 weeks.

NEED FOR EVIDENCE:
- There is an association between milk feeding during blood transfusion and NEC, plausibly due to altered blood flow and intestinal ischaemia. Preterm infants are among the most transfused patient groups, more than 90% of babies under 30 weeks gestation at birth will receive a blood transfusion and babies transfused received a mean of 4 packed red cell transfusions during their neonatal unit admission (range of 1-27 transfusions).
- In a 2012 systematic review two observational studies were identified and showed an association between withholding feeds during blood transfusion and a reduced incidence of NEC. The authors concluded that “withholding feeding during transfusion may reduce the risk of NEC, although the level of evidence is low” and suggested that randomised controlled trials be carried out. We have updated this systematic review (one further observational study was identified) and performed a meta-analysis that showed a halving of NEC (odds ratio 0.53) following withholding feeds during blood transfusion. There are no published randomised controlled trials that have examined this research question.

RELEVANCE:
- Current UK clinical practice varies, reflecting the limited evidence base. We conducted a national survey in 2014 that showed that 1 in 3 UK neonatal units routinely withhold feeds during blood transfusions, while other units continue to feed. Both approaches being compared in WHEAT are considered to be standard UK practice.
- The simple practice of withholding milk feeds during and round the time of packed red cell transfusion might prevent deaths from NEC and reduce the significant long-term health and neurodevelopmental burden associated with this disease. Conversely, given the trophic effects of human milk that contains a number of growth factors and immunological agents, it is biologically plausible that episodes of withholding feeds in preterm babies might adversely affect intestinal integrity and development, and increase the risk of NEC. Equally, if withholding enteral feeds has no effect on the development of NEC this practice can be safely discontinued and the associated negative nutritional effects avoided.

JUSTIFICATION:
- Professional and parent opinion supports the need for a trial; prevention of NEC has been identified by service users and health professionals as the third most important treatment uncertainty in the field of preterm birth (NIHR James Lind Alliance preterm birth priority setting partnership, Duley et al., Lancet 2014), preceded only by “prevention of preterm birth” and “prevention of infection”.
- The National Blood and Transplant Serious Hazards Of Transfusion (SHOT) report in 2012 and 2013 reported cases of NEC possibly associated with packed red cell transfusion and called for prospective studies to investigate a causal relationship. In addition there have been multiple published calls for a large scale randomized controlled trial from academics, clinicians and nursing professionals.

A13. Please summarise your design and methodology. *It should be clear exactly what will happen to the research participant, how many times and in what order. Please complete this section in language comprehensible to the lay person. Do not simply reproduce or refer to the protocol. Further guidance is available in the guidance notes.*

The hypothesis being tested is that withholding enteral feeds around blood transfusion leads to a reduction in severe necrotising enterocolitis (NEC).

DESIGN: Randomised, controlled, parallel arm, open, multicentre, superiority registry trial; central block randomisation, variable block sizes, 1:1 allocation; stratification by site, minimisation by sex, gestational age and birth weight.

A randomised registry trial is a randomised trial where electronic health records (EHR) are used to improve efficiency

and reduce costs: identification and recruitment of research participants is incorporated into the EHR, and all trial data are extracted from an existing database containing data extracted from the EHR.

OUTCOMES:
Primary: Severe NEC (diagnosis by histology OR at laparotomy/post mortem OR recorded on death certificate)
Secondary: Death before hospital discharge; length of hospital stay; NEC of lesser severity.

SETTING: 110 UK neonatal units.

INTERVENTION: Stopping milk feeds 4 hours before, during and for 4 hours after blood transfusion and providing intravenous fluid (this is standard care for approximately.

CONTROL: No alteration in enteral feeding. The control represents the most common practice in the UK (approximately 2/3 of neonatal units do not alter enteral feeds during blood transfusion).

SAMPLE SIZE: A total sample of 4650 would provide 80% power (5% significance) to detect a plausible risk reduction in severe NEC from 4% to 2.5%.

TRIAL DATA COLLECTION: All trial data will be obtained from an existing research database, the National Neonatal Research Database (NNRD). This holds patient level clinical data from 2007 to the present extracted quarterly from the EHR of all admissions to NHS neonatal units in England and Wales. Data items are a NHS Information Standard and appropriate approvals are held. NNRD data has been validated against clinical trial data and patient notes. During the WHEAT trial, key data items (allocation and outcome data) will be validated by local clinicians using an established national system.

DURATION: 42 months, 36 month trial, 4 months follow up, 2 months write up.

PARTICIPANT TIMELINE: All preterm infants born at <30 weeks gestational age that survive delivery are admitted to a neonatal unit immediately after birth. In UK NHS neonatal units all babies have admission details (such as gestational age and birth weight) entered onto an EHR system shortly after admission. In neonatal units participating in the WHEAT trial the EHR will be programmed to automatically flag babies that meet the inclusion criteria for the WHEAT trial to clinical staff. Clinical staff will then approach the parents of eligible babies, explain WHEAT and the opt-out consent process and provide them with a copy of the parent information sheet. The clinical staff member will then record that this explanation has been given to parents on the EHR. A nationally agreed UK standard (audited as part of the National Neonatal Audit Programme) is that all parents are spoken to by a senior member of staff within 24 hours of neonatal unit admission, we expect that explanation of the WHEAT trial will occur within the same period.

Where parents choose not to opt out their baby will be randomised to either be fed or not fed during blood transfusions. In practice this means that when the clinical team looking after a participating baby decide that a blood transfusion is clinically indicated AND the baby is receiving milk feeds the baby will either:

1. Be made nil by mouth from 4 hours before the blood transfusion, during the blood transfusion (this lasts about 4 hours) and for 4 hours after the blood transfusion is finished (approximately 12 hours in total). During this period the baby will be placed on intravenous fluid or nutrition as per local unit policy.

OR

2. Have milk feeds continued before, during and after the blood transfusion.

If the baby requires any further blood transfusions during their neonatal unit stay they will remain allocated to the same intervention group.

There will be no other involvement of the baby or their family in the WHEAT trial. All trial data (including outcome data) will be extracted from existing EHR data that is entered in the course of routine clinical care.

A14-1. In which aspects of the research process have you actively involved, or will you involve, patients, service users, and/or their carers, or members of the public?

- [x] Design of the research
- [x] Management of the research
- [ ] Undertaking the research
- [ ] Analysis of results

- [x] Dissemination of findings
- [ ] None of the above

Give details of involvement, or if none please justify the absence of involvement.
Choice of research question: “What interventions are most effective to prevent NEC?” is ranked the third most important research question by service users and health professionals in the NIHR James Lind Alliance Preterm Birth Priority Setting Partnership (Duley et al., Lancet 2014), of which Chris Gale was a steering group member.

Design of the research: We convened parent focus groups that confirmed strong support, informed trial design, and contributed to outcome measure selection. WHEAT has received favourable lay review and is supported by the NIHR Enabling Involvement Fund.   A mother of preterm twins (HR) and a representative of Bliss (ZC), the charity for babies "born too soon, too small or too sick", are co-applicants and have helped design the trial as members of the trial development group.

Management of the research: HR and ZC are co-applicants and will be involved in ongoing management of the trial as members of the trial steering group.

Dissemination of findings: The dissemination of trial findings will be led by HR and ZC.

4. RISKS AND ETHICAL ISSUES

RESEARCH PARTICIPANTS

A15. What is the sample group or cohort to be studied in this research?

Select all that apply:

- [ ] Blood
- [ ] Cancer
- [ ] Cardiovascular
- [ ] Congenital Disorders
- [ ] Dementias and Neurodegenerative Diseases
- [ ] Diabetes
- [ ] Ear
- [ ] Eye
- [ ] Generic Health Relevance
- [ ] Infection
- [ ] Inflammatory and Immune System
- [ ] Injuries and Accidents
- [ ] Mental Health
- [ ] Metabolic and Endocrine
- [ ] Musculoskeletal
- [ ] Neurological
- [ ] Oral and Gastrointestinal
- [x] Paediatrics
- [ ] Renal and Urogenital
- [ ] Reproductive Health and Childbirth
- [ ] Respiratory
- [ ] Skin
- [ ] Stroke

Gender:	Male and female participants
Lower age limit: 22	Weeks gestational age
Upper age limit: 30	Weeks gestational age

A17-1. Please list the principal inclusion criteria (list the most important, max 5000 characters).

Gestational age at birth <30 weeks (up to and including 29+6 weeks).

Babies will be recruited shortly after birth (and therefore before they are scheduled to have a blood transfusion in most cases). Blood transfusion is almost universal in babies of this gestational age (90-95% will need at least one blood transfusion prior to discharge).

A17-2. Please list the principal exclusion criteria (list the most important, max 5000 characters).

No exclusion criteria.

RESEARCH PROCEDURES, RISKS AND BENEFITS

A18. Give details of all non-clinical intervention(s) or procedure(s) that will be received by participants as part of the research protocol. *These include seeking consent, interviews, non-clinical observations and use of questionnaires.*

Please complete the columns for each intervention/procedure as follows:

1. Total number of interventions/procedures to be received by each participant as part of the research protocol.
2. If this intervention/procedure would be routinely given to participants as part of their care outside the research, how many of the total would be routine?
3. Average time taken per intervention/procedure (minutes, hours or days)
4. Details of who will conduct the intervention/procedure, and where it will take place.

Intervention or procedure	1	2	3	4
Explaining the trial and the opt-out consent procedure.	1	0	30 minutes	A member of the local clinical, nursing or research team

A19. Give details of any clinical intervention(s) or procedure(s) to be received by participants as part of the research protocol. *These include uses of medicinal products or devices, other medical treatments or assessments, mental health interventions, imaging investigations and taking samples of human biological material. Include procedures which might be received as routine clinical care outside of the research.*

Please complete the columns for each intervention/procedure as follows:

1. Total number of interventions/procedures to be received by each participant as part of the research protocol.
2. If this intervention/procedure would be routinely given to participants as part of their care outside the research, how many of the total would be routine?
3. Average time taken per intervention/procedure (minutes, hours or days).
4. Details of who will conduct the intervention/procedure, and where it will take place.

Intervention or procedure	1	2	3	4
Withholding enteral feeds before, during and after transfusion of packed red cells with concurrent provision of intravenous fluid/dextrose.<br><br>Eligible babies have on average 4 transfusions (range 1-27)	4	0	12 hours	Neonatal nurse on the neonatal unit.<br><br>This intervention will only apply to half of the enrolled infants (those randomised to the intervention arm). This practice is routine clinical care in approximately 1/3 of UK neonatal units.
Siting an additional intravenous cannula	1	0	10	Neonatal doctor or nurse on the neonatal unit.

for provision of i.v. fluid.	minutes	This intervention will only apply to half of the enrolled infants (those randomised to the intervention arm), and only in a limited number of cases (estimated to be one here - usually there will be sufficient i.v. access).<br><br>This practice is routine clinical care in approximately 1/3 of UK neonatal units.

A20. Will you withhold an intervention or procedure, which would normally be considered a part of routine care?

○ Yes ◉ No

A21. How long do you expect each participant to be in the study in total?

Participants will be in the study until they are discharged from neonatal care. The mean duration of neonatal care for babies in the WHEAT trial (<30 weeks gestational age) is 12 weeks.

A22. What are the potential risks and burdens for research participants and how will you minimise them?

For all studies, describe any potential adverse effects, pain, discomfort, distress, intrusion, inconvenience or changes to lifestyle. Only describe risks or burdens that could occur as a result of participation in the research. Say what steps would be taken to minimise risks and burdens as far as possible.

There are 2 potential burdens of withholding feeds around blood transfusion:

1. HUNGER: The parent member of the WHEAT trial development group (HR) has identified hunger in infants allocated to the intervention arm as a potential burden, although it must also be noted that withholding feeds, and the possible hunger associated with it, is routine practice in approximately 1 in 3 UK neonatal units.

Babies that are randomised to receive the intervention (withholding enteral feeds around blood transfusion) will undergo a 12 hour period where they receive no enteral (milk) feeds. During this period they will receive intravenous nutrition or dextrose (the choice of which be left to the discretion of to the clinical team). It is not known when preterm babies start to experience hunger and if they do, whether this is alleviated by intravenous dextrose or nutrition. The provision of intravenous dextrose or nutrition will be provided to all babies to ensure they do not become hypoglycaemic or dehydrated. This practice will minimise as much as possible the potential discomfort of hunger. HR has helped to develop the patient information sheet to explain this.

2. A SECOND I.V. DRIP: In a limited number of cases a second intravenous drip (in addition to the intravenous drip that will be used to transfuse the blood) may be needed to infuse intravenous dextrose or nutrition to babies allocated to the withholding feeds arm. This will only represent a deviation from routine practice in the 2/3 of UK units that continue feeds during blood transfusions and only in babies randomised to the intervention group. This will only apply to a small number of babies - babies that are fully milk fed but need to be fed milk every 3 hours or less.

For babies receiving feeds every 4 hours:
- The intravenous drip that will be needed to transfuse blood can be sited 4 hours earlier and used to infuse dextrose or nutrition before the transfusion.
- The same intravenous drip can be used following the blood transfusion to infuse dextrose or nutrition.
- During the blood transfusion itself (which lasts for 4 hours) intravenous dextrose or nutrition can be safely discontinued.

For babies already requiring intravenous fluid or nutrition in addition to enteral feeds:
- The amount of intravenous fluid or nutrition can be increased to compensate for the enteral feeds that will be withheld.

A23. Will interviews/ questionnaires or group discussions include topics that might be sensitive, embarrassing or upsetting, or is it possible that criminal or other disclosures requiring action could occur during the study?

○ Yes ◉ No

A24. What is the potential for benefit to research participants?

Recent evidence from a large multicentre neonatal comparative effectiveness trial (a study that compares treatments that are accepted and used in day to day practice, like the WHEAT trial) demonstrates that infants participating in the trial had lower rates of death and disability when compared to both infants who did not participate and to historical controls (Carlo et al., 2013 NEJM). This supports previous neonatal studies that have shown a similar "inclusion benefit" (Schmidt et al., J Perinatol 1999).

For this reason we have stated that there may be a benefit of participating in a clinical trial such as WHEAT in the patient information sheet:

"Each of the two options in the WHEAT study is currently used by doctors in the UK because we do not know which one is better. For babies not taking part in the WHEAT study, the choice of whether or not to stop feeds is made according to the preference of the local medical team.
This non-evidence based approach to neonatal care may involve more risk than being in a study like WHEAT which involves a carefully designed protocol and consistent monitoring."

A25. What arrangements are being made for continued provision of the intervention for participants, if appropriate, once the research has finished? *May apply to any clinical intervention, including a drug, medical device, mental health intervention, complementary therapy, physiotherapy, dietary manipulation, lifestyle change, etc.*

Enrolment in the WHEAT trial will end when a baby is discharged from the neonatal unit. The intervention that is being tested in the WHEAT trial is only applicable to babies who are admitted to neonatal units (it is not applicable to paediatric intensive care units for example). For this reason continued provision of the intervention (withholding enteral feeds during blood transfusion) is not an issue in the WHEAT trial.

A26. What are the potential risks for the researchers themselves? *(if any)*

There are no risks to the researchers; the WHEAT trial will take place entirely within the neonatal unit.

RECRUITMENT AND INFORMED CONSENT

In this section we ask you to describe the recruitment procedures for the study. Please give separate details for different study groups where appropriate.

A27-1. How will potential participants, records or samples be identified? Who will carry this out and what resources will be used? *For example, identification may involve a disease register, computerised search of social care or GP records, or review of medical records. Indicate whether this will be done by the direct care team or by researchers acting under arrangements with the responsible care organisation(s).*

All surviving preterm infants born at <30 weeks gestational age are admitted to a neonatal unit immediately after birth. In UK NHS neonatal units admission details are entered onto an electronic health record system. In the WHEAT trial, the electronic health record in participating neonatal units will be modified to automatically identify (flag) eligible infants (gestational age <30 weeks at birth) to the clinical team.

A27-2. Will the identification of potential participants involve reviewing or screening the identifiable personal information of patients, service users or any other person?

○ Yes ◉ No

Please give details below:
Only the direct clinical team will be involved in screening patient information.

A28. Will any participants be recruited by publicity through posters, leaflets, adverts or websites?

○ Yes ◉ No

A29. How and by whom will potential participants first be approached?

The parents of eligible babies will be approached by members of the clinical team. This may be neonatal doctors or nurses and will usually occur on the neonatal unit. It is an accepted national standard (and forms part of the National Neonatal Audit Progamme) that parents speak to a senior member of the neonatal team within 24 hours of their baby being admitted to a neonatal unit. We expect that the first explanation of the WHEAT trial and of "opt out" consent will take place over the same period.

In some circumstances (for example if a mother is admitted for monitoring or management of preterm labour) potential parents may be initially approached before the baby is delivered. In this circumstance the approach will still be by members of the neonatal clinical team, and the trial will be explained again after the baby is delivered.

A30-1. Will you obtain informed consent from or on behalf of research participants?

(●) Yes ( ) No

If you will be obtaining consent from adult participants, please give details of who will take consent and how it will be done, with details of any steps to provide information (a written information sheet, videos, or interactive material). Arrangements for adults unable to consent for themselves should be described separately in Part B Section 6, and for children in Part B Section 7.

If you plan to seek informed consent from vulnerable groups, say how you will ensure that consent is voluntary and fully informed.

Informed opt-out consent will be sought on behalf of all participants.

If you are not obtaining consent, please explain why not.

Please enclose a copy of the information sheet(s) and consent form(s).

A30-2. Will you record informed consent (or advice from consultees) in writing?

( ) Yes (●) No

If No, how will it be recorded?

Informed opt-out consent will be obtained from parents.

The WHEAT trial will be explained to parents shortly after their baby is admitted to the neonatal unit. Parents will be provided with the parent information sheet that explains the WHEAT trial and makes it clear that both arms (withholding feeds and continuing feeds) represent current clinical practice.

The parent information sheet will make it clear that opt-out consent is being requested. This means that if they do not want their baby to take part in the WHEAT trial they can inform the local clinical team at any time.

After the WHEAT trial and the "opt out" consent procedure have been explained to parents/carers this will be recorded in the Electronic Health Record. As a fail safe, randomisation to intervention or control arms will not be possible until this explanation has been recorded.

A31. How long will you allow potential participants to decide whether or not to take part?

Parents will be approached shortly after their baby is admitted to the neonatal unit (usually within the first 24 hours). Parents will be able to opt out of the WHEAT trial at any point.

A32. Will you recruit any participants who are involved in current research or have recently been involved in any research prior to recruitment?

(●) Yes
( ) No
( ) Not Known

If Yes, please give details and justify their inclusion. If Not Known, what steps will you take to find out?

Because both trial arms represent standard clinical practice there is no reason to exclude participants who are or have recently been involved in other research as even outside the WHEAT trial preterm babies will be exposed to one arm; all the WHEAT trial is doing is randomising this decision (rather than relying on a non-evidence based clinician preference).

A33-1. What arrangements have been made for persons who might not adequately understand verbal explanations or written information given in English, or who have special communication needs?*(e.g. translation, use of interpreters)*

Interpreters will be used to explain the study and to obtain informed opt-out consent.

A33-2. What arrangements will you make to comply with the principles of the Welsh Language Act in the provision of information to participants in Wales?

All study documents will be translated into Welsh.

A34. What arrangements will you make to ensure participants receive any information that becomes available during the course of the research that may be relevant to their continued participation?

The Trial Steering Committee and Data Monitoring Committee (DMC) will be responsible for searching for relevant information that might become available during the course of the research study. If relevant information becomes available this will be reviewed by the DMC who will independently decide whether further action is needed and if so what this will entail.

A35. What steps would you take if a participant, who has given informed consent, loses capacity to consent during the study? *Tick one option only.*

- ○ The participant and all identifiable data or tissue collected would be withdrawn from the study. Data or tissue which is not identifiable to the research team may be retained.
- ◉ The participant would be withdrawn from the study. Identifiable data or tissue already collected with consent would be retained and used in the study. No further data or tissue would be collected or any other research procedures carried out on or in relation to the participant.
- ○ The participant would continue to be included in the study.
- ○ Not applicable – informed consent will not be sought from any participants in this research.
- ○ Not applicable – it is not practicable for the research team to monitor capacity and continued capacity will be assumed.

Further details:

If you plan to retain and make further use of identifiable data/tissue following loss of capacity, you should inform participants about this when seeking their consent initially.

CONFIDENTIALITY

In this section, personal data means any data relating to a participant who could potentially be identified. It includes pseudonymised data capable of being linked to a participant through a unique code number.

Storage and use of personal data during the study

A36. Will you be undertaking any of the following activities at any stage (including in the identification of potential participants)?*(Tick as appropriate)*

- ☑ Access to medical records by those outside the direct healthcare team
- ☐ Access to social care records by those outside the direct social care team
- ☐ Electronic transfer by magnetic or optical media, email or computer networks
- ☐ Sharing of personal data with other organisations

- [ ] Export of personal data outside the EEA
- [x] Use of personal addresses, postcodes, faxes, emails or telephone numbers
- [ ] Publication of direct quotations from respondents
- [ ] Publication of data that might allow identification of individuals
- [ ] Use of audio/visual recording devices
- [ ] Storage of personal data on any of the following:
  - [ ] Manual files (includes paper or film)
  - [x] NHS computers
  - [ ] Social Care Service computers
  - [ ] Home or other personal computers
  - [ ] University computers
  - [ ] Private company computers
  - [ ] Laptop computers

Further details:
Medical records will be accessed by members of the study team for the purposes of monitoring and audit, informed consent will be obtained prior to doing this.

A37. Please describe the physical security arrangements for storage of personal data during the study?

Trial data will be extracted from and held as part of the National Neonatal Research Database (NNRD), based at Chelsea and Westminster NHS Foundation Trust. Approval for the NNRD is held from Caldicott Guardians and Lead Clinicians of all neonatal units in England and Wales, National Research Ethics Service (10/H0803/151), and Confidentiality Advisory Group of the Health Research Authority (8-05(f)/2010), while the Neonatal Data Set has been approved by the Review of Central Returns office (ROCR-Lite/OR/2027/FT6/001PMAND).

Data will be held in the secure Information Technology environment of Chelsea and Westminster Hospital.

A38. How will you ensure the confidentiality of personal data?*Please provide a general statement of the policy and procedures for ensuring confidentiality, e.g. anonymisation or pseudonymisation of data.*

Data will be held as part of the National Neonatal Research Database (NNRD): appropriate NNRD, Chelsea and Westminster Trust IT and NHS Codes will be followed to ensure confidentiality of personal data.

A40. Who will have access to participants' personal data during the study? *Where access is by individuals outside the direct care team, please justify and say whether consent will be sought.*

Medical records will be accessed by members of the study team for the purposes of monitoring and audit, informed consent will be obtained prior to doing this.

Storage and use of data after the end of the study

A41. Where will the data generated by the study be analysed and by whom?

Data generated by the study will be analysed by the study team and at the National Neonatal Research Database at Chelsea and Westminster NHS Foundation Trust.   Data will not be exported outside the UK.

A42. Who will have control of and act as the custodian for the data generated by the study?

	Title	Forename/Initials	Surname
	Prof	Neena	Modi
Post	Professor of Neonatal Medicine		
Qualifications	MBBS, MD		
Work Address	4th Floor<br>Chelsea and Westminster Hospital<br>London		
Post Code	SW10 9NH		
Work Email	n.modi@imperial.ac.uk		
Work Telephone	+44 (0)20 8237 5102		
Fax	+44 (0)20 8746 8887		

A43. How long will personal data be stored or accessed after the study has ended?

- ○ Less than 3 months
- ○ 3 – 6 months
- ○ 6 – 12 months
- ○ 12 months – 3 years
- ◉ Over 3 years

If longer than 12 months, please justify:
Personal data will be destroyed after 10 years as per Imperial College policy.

A44. For how long will you store research data generated by the study?

Years: 10
Months: 0

A45. Please give details of the long term arrangements for storage of research data after the study has ended. *Say where data will be stored, who will have access and the arrangements to ensure security.*

Research data will be stored as part of the National Neonatal Research Database, as part of a national resource. Access to anonymised study data will be granted to appropriate organisations following applications to the WHEAT trial steering group. Access to identifiable information will be limited to the WHEAT trial PIs (NM and CG).

INCENTIVES AND PAYMENTS

A46. Will research participants receive any payments, reimbursement of expenses or any other benefits or incentives for taking part in this research?

○ Yes ◉ No

A47. Will individual researchers receive any personal payment over and above normal salary, or any other benefits or incentives, for taking part in this research?

○ Yes ◉ No

A48. Does the Chief Investigator or any other investigator/collaborator have any direct personal involvement (e.g. financial, share holding, personal relationship etc.) in the organisations sponsoring or funding the research that may give rise to a possible conflict of interest?

○ Yes ◉ No

NOTIFICATION OF OTHER PROFESSIONALS

A49-1. Will you inform the participants' General Practitioners (and/or any other health or care professional responsible for their care) that they are taking part in the study?

○ Yes ◉ No

If Yes, please enclose a copy of the information sheet/letter for the GP/health professional with a version number and date.

PUBLICATION AND DISSEMINATION

A50-1. Will the research be registered on a public database?

The Department of Health's Research Governance Framework for Health and Social Care and the research governance frameworks for Wales, Scotland and Northern Ireland set out the requirement for registration of trials. Furthermore: Article 19 of the World Medical Association Declaration of Helsinki adopted in 2008 states that "every clinical trial must be registered on a publicly accessible database before recruitment of the first subject"; and the International Committee of Medical Journal Editors (ICMJE) will consider a clinical trial for publication only if it has been registered in an appropriate registry. Please see guidance for more information.

◉ Yes ○ No

Please give details, or justify if not registering the research.
The WHEAT trial will be registered on ClinicalTrials.gov immediately following REC approval (Research Ethics approval is required for registration on ClinicalTrials.gov)

Please ensure that you have entered registry reference number(s) in question A5-1.

A51. How do you intend to report and disseminate the results of the study? *Tick as appropriate:*

- [x] Peer reviewed scientific journals
- [ ] Internal report
- [x] Conference presentation
- [x] Publication on website
- [x] Other publication
- [ ] Submission to regulatory authorities
- [ ] Access to raw data and right to publish freely by all investigators in study or by Independent Steering Committee on behalf of all investigators
- [ ] No plans to report or disseminate the results
- [ ] Other (please specify)

Dissemination to academic, nursing and medical professionals will be through peer reviewed publications, conference presentations and through professional networks and organisations. Dissemination of results to parents, patients and the public will be led by HR and AC (the parent and parent advocate members of the trial steering group respectively) and will be via charity websites and social media outlets.

A52. If you will be using identifiable personal data, how will you ensure that anonymity will be maintained when publishing the results?

Data will be presented in aggregate form. Due to the large size of WHEAT this will ensure that anonymity is maintained.

A53. Will you inform participants of the results?

(●) Yes ( ) No

Please give details of how you will inform participants or justify if not doing so.
Parents of babies that have participated in the WHEAT trial will be informed about the results by post. A telephone number and email address for the investigator team will be provided with this information so that any questions that parents may have can be answered. This will occur before trial results are made available in any other form.

5. Scientific and Statistical Review

A54-1. How has the scientific quality of the research been assessed? *Tick as appropriate:*

- [ ] Independent external review
- [ ] Review within a company
- [x] Review within a multi−centre research group
- [ ] Review within the Chief Investigator's institution or host organisation
- [x] Review within the research team
- [ ] Review by educational supervisor
- [x] Other

Justify and describe the review process and outcome. If the review has been undertaken but not seen by the researcher, give details of the body which has undertaken the review:

For all studies except non-doctoral student research, please enclose a copy of any available scientific critique reports, together with any related correspondence.

For non-doctoral student research, please enclose a copy of the assessment from your educational supervisor/ institution.

A56. How have the statistical aspects of the research been reviewed? *Tick as appropriate:*

- [ ] Review by independent statistician commissioned by funder or sponsor
- [ ] Other review by independent statistician
- [ ] Review by company statistician
- [ ] Review by a statistician within the Chief Investigator’s institution
- [x] Review by a statistician within the research team or multi−centre group
- [ ] Review by educational supervisor
- [ ] Other review by individual with relevant statistical expertise
- [ ] No review necessary as only frequencies and associations will be assessed – details of statistical input not required

In all cases please give details below of the individual responsible for reviewing the statistical aspects. If advice has been provided in confidence, give details of the department and institution concerned.

Department
Institution
Work Address

Post Code
Telephone
Fax

Mobile
E-mail

Please enclose a copy of any available comments or reports from a statistician.

A57. What is the primary outcome measure for the study?

Necrotising enterocolitis confirmed by histology of resected bowel OR visual inspection at laparotomy OR visual inspection at post mortem examination OR recorded on the death certificate (part 1a, 1b or 2).

A58. What are the secondary outcome measures? *(if any)*

1. Necrotising enterocolitis – case definition based on clinical factors
2. All-cause mortality before neonatal discharge
3. Length of stay

A59. What is the sample size for the research? *How many participants/samples/data records do you plan to study in total? If there is more than one group, please give further details below.*

Total UK sample size:	4650
Total international sample size (including UK):	4650
Total in European Economic Area:	4650

Further details:
In 2012 4528 babies were born at <30 weeks in neonatal units in England and Wales and would therefore be eligible for the WHEAT trial. Assuming that 50% of units take part in the WHEAT trial and 70% of eligible babies are recruited (based on a recruitment rate of 84% in a recent neonatal randomised trial: BOOST II), this would approximate to 1585 babies recruited per year, and target recruitment (4650 babies) would be achieved in 36 months.

A60. How was the sample size decided upon? *If a formal sample size calculation was used, indicate how this was done, giving sufficient information to justify and reproduce the calculation.*

Among babies born in 2012 at less than 30 gestational weeks and in units contributing to the NNRD (n= 4528), the incidence of NEC defined according to the WHEAT trial primary outcome is 4%. This is in keeping with international figures.

An updated meta-analysis of observational studies (including 3 historical control studies) finds a pooled odds ratio of 0.53 [95% CI 0.36, 0.78] for development of NEC in the withholding feeds group (p=0.001, heterogeneity I2=0%).

There is evidence that the treatment effect observed in observational studies may not be truly representative of (and may over-estimate) the treatment effect found in randomised trials. This appears particularly marked in historical control studies, where 70% of studies were found to have a >50% difference in odds ratio when compared to randomised studies (Sack H et al., Am J Med 1982).

To account for possible over-estimation of the effect size by 50%, the effect size used for the sample size calculation been reduced by 33%. Assuming 4% incidence as a baseline, this conservative effect size gives an incidence of 2.5% in the intervention group. Assuming a 5% significance level, we would require 2325 per group for 80% power. An absolute risk reduction of this size (1.5%) would be clinically important.

A61-1. Will participants be allocated to groups at random?

(•) Yes ( ) No

If yes, please give details of the intended method of randomisation:
Infants will be randomly assigned to either intervention or control groups with a 1:1 allocation as per computer generated randomisation sequence stratified by site using permutated blocks of random sizes and minimised b sex, gestational age and birthweight centile for gestational age. The block sizes will not be disclosed to ensure concealment.

Minimisation will be into the following catagories:

1. Sex.
2. Gestational age: 28+0-29+6 weeks OR <28 weeks
3. Birthweight centile for gestational age: >10th centile OR <10th centile

Participants will be randomised using an central randomisation service which will be incorporated into the EHR (badger.net platform).

A62. Please describe the methods of analysis (statistical or other appropriate methods, e.g. for qualitative research) by which the data will be evaluated to meet the study objectives.

Demographic factors and clinical characteristics collected as part of baseline data collection will be summarised with counts (percentages) for categorical variables, mean (standard deviation [SD]) for normally distributed continuous variables, or median (interquartile [IQR] or entire range) for other continuous variables.

Outcomes for participants will be analysed in the groups to which they are assigned regardless of deviation from the protocol or treatment received (intention to treat analysis). Comparative statistical analysis will entail calculating the relative risk (RR) (95% CI) for the primary outcome (99% CIs for all other dichotomous outcomes), the mean difference (99% CI) for normally distributed continuous outcomes, or the median difference (99% CI) for skewed continuous variables.

The two groups will be compared using regression analysis, adjusting for the stratification factors to account for the correlation between treatment groups introduced by balancing the randomisation. Both the crude unadjusted and adjusted estimates will be presented, but the primary inference will be based on the adjusted analysis.

6. MANAGEMENT OF THE RESEARCH

A63. Other key investigators/collaborators. *Please include all grant co-applicants, protocol co-authors and other key members of the Chief Investigator's team, including non-doctoral student researchers.*

	Title	Forename/Initials	Surname
	Prof	N	Modi

Post: Professor of Neonatal Medicine
Qualifications:
Employer: Imperial College London
Work Address: 4th Floor
Chelsea and Westminster Hospital
London
Post Code: SW10 9NH
Telephone: +44 (0)20 8237 5102
Fax: +44 (0)20 8746 8887
Mobile:
Work Email: n.modi@imperial.ac.uk

	Title	Forename/Initials	Surname
	Prof	T P	van Staa

Post: Professor of eHealth Research
Qualifications:
Employer: Farr Institute Health eResearch Centre, University of Manchester
Work Address:

Post Code	
Telephone	
Fax	
Mobile	
Work Email	tjeerd.vanstaa@manchester.ac.uk

	Title	Forename/Initials	Surname
	Dr	M	Turner
Post	Clinical Academic and Neonatologist		
Qualifications			
Employer	University of Liverpool		
Work Address			
Post Code			
Telephone	01517959558		
Fax			
Mobile			
Work Email	mturner@liverpool.ac.uk		

	Title	Forename/Initials	Surname
	Dr	J	Dorling
Post	Clinical Associate Professor (Neonatal Medicine)		
Qualifications			
Employer	University of Nottingham		
Work Address	Room E/E1720<br>Queens Medical Centre Queen's Medical Centre<br>Nottingham		
Post Code	NG7 2UH		
Telephone	0115 8230635		
Fax			
Mobile			
Work Email	Jon.Dorling@nuh.nhs.uk		

	Title	Forename/Initials	Surname
	Mrs	H	Robberts
Post	Parent of preterm twins		
Qualifications			
Employer			
Work Address			
Post Code			
Telephone			
Fax			
Mobile			
Work Email	helenrobberts@hotmail.com		

	Title	Forename/Initials	Surname

	Miss	Z	Chivers
Post	Innovations Manager		
Qualifications			
Employer	Bliss (National charity for babies born too soon, too small, too sick)		
Work Address			
Post Code			
Telephone	020 7378 1122		
Fax			
Mobile			
Work Email	zoec@bliss.org.uk		

	Title	Forename/Initials	Surname
	Mrs	Amanda	Forster
Post	Neonatal Nurse		
Qualifications			
Employer	James Cook University Hospital NHS Trust		
Work Address	Neonatal Unit<br>James Cook University Hospital, Marton Road,<br>Middlesbrough, Cleveland		
Post Code	TS4 3BW		
Telephone	01642854871		
Fax			
Mobile			
Work Email	Amanda.forster@stees.nhs.uk		

A64. Details of research sponsor(s)

A64-1. Sponsor

Lead Sponsor

Status:
- ( ) NHS or HSC care organisation
- (•) Academic
- ( ) Pharmaceutical industry
- ( ) Medical device industry
- ( ) Other

Commercial status: Non-Commercial

If Other, please specify:

Contact person

Name of organisation
Given name
Family name
Address
Town/city

Post code
Country
Telephone
Fax
E-mail

Is the sponsor based outside the UK?

○ Yes ◉ No

Under the Research Governance Framework for Health and Social Care, a sponsor outside the UK must appoint a legal representative established in the UK. Please consult the guidance notes.

A65. Has external funding for the research been secured?

☐ Funding secured from one or more funders
☑ External funding application to one or more funders in progress
☐ No application for external funding will be made

What type of research project is this?

◉ Standalone project
○ Project that is part of a programme grant
○ Project that is part of a Centre grant
○ Project that is part of a fellowship/ personal award/ research training award
○ Other

Other – please state:

Please give details of funding applications.

Organisation
Address

Post Code
Telephone
Fax
Mobile
Email

Funding Application Status: ○ Secured ◉ In progress

Date Funding decision expected:

Amount:

Duration
Years:
Months:

A66. Has responsibility for any specific research activities or procedures been delegated to a subcontractor (other than a co-sponsor listed in A64-1) ? *Please give details of subcontractors if applicable.*

○ Yes ◉ No

A67. Has this or a similar application been previously rejected by a Research Ethics Committee in the UK or another country?

○ Yes ◉ No

Please provide a copy of the unfavourable opinion letter(s). You should explain in your answer to question A6-2 how the reasons for the unfavourable opinion have been addressed in this application.

A68-1. Give details of the lead NHS R&D contact for this research:

Organisation
Address

Post Code
Work Email
Telephone
Fax
Mobile

Details can be obtained from the NHS R&D Forum website: http://www.rdforum.nhs.uk

A68-2. Select Comprehensive Local Research Network for this NHS organisation:

To support communication between the REC and R&D contacts for this study, please select the Comprehensive Local Research Network (CLRN) for this NHS organisation. This CLRN will be the Lead CLRN for your study.

London (NW)

For information about support and advice available through the Lead CLRN and the CLRNs for participating sites see http://www.crncc.nihr.ac.uk/about_us/processes/csp. A map showing the CLRNs is available at http://www.crncc.nihr.ac.uk/about_us/ccrn.

A69-1. How long do you expect the study to last in the UK?

Planned start date: 01/06/2015
Planned end date: 31/03/2019
Total duration:

Years: 3 Months: 9 Days: 31

A71-1. Is this study?

- ( ) Single centre
- (•) Multicentre

A71-2. Where will the research take place? *(Tick as appropriate)*

- [x] England
- [ ] Scotland
- [x] Wales
- [ ] Northern Ireland
- [ ] Other countries in European Economic Area

Total UK sites in study 110

Does this trial involve countries outside the EU?

( ) Yes (•) No

A72. Which organisations in the UK will host the research? *Please indicate the type of organisation by ticking the box and give approximate numbers if known:*

- [x] NHS organisations in England 110
- [ ] NHS organisations in Wales
- [ ] NHS organisations in Scotland
- [ ] HSC organisations in Northern Ireland
- [ ] GP practices in England
- [ ] GP practices in Wales
- [ ] GP practices in Scotland
- [ ] GP practices in Northern Ireland
- [ ] Joint health and social care agencies (eg community mental health teams)
- [ ] Local authorities
- [ ] Phase 1 trial units
- [ ] Prison establishments
- [ ] Probation areas
- [ ] Independent (private or voluntary sector) organisations
- [ ] Educational establishments
- [ ] Independent research units
- [ ] Other (give details)

Total UK sites in study: 110

A73-1. Will potential participants be identified through any organisations other than the research sites listed above?

( ) Yes (•) No

A74. What arrangements are in place for monitoring and auditing the conduct of the research?

Central statistical monitoring will be used at the National Neonatal Research Database to monitor patterns of recruitment at sites and within the data; outlier data will be investigated and may trigger ‘for cause’ site monitoring.

The Head of Trials and the Senior Trials Programmer (at the Manchester Clinical Trials Unit) will develop an appropriate central monitoring plan for the trial and review the output to identify any unexpected patterns or problems. The Head of Trials will decide if any action needs to be taken.

The local researchers assisted by the NIHR Local Research Network (LRN) will be responsible for the day-to-day smooth running of the trial at a recruiting site. They will encourage recruitment, provide staff education and training, and monitor data collection completeness and quality.

The local researchers will submit formal site visit reports to the Machester Clinical Trials Unit. No other routine monitoring will be carried out unless the central monitoring exercises raise cause for concern. Likewise, sites will only be audited if central monitoring indicates a necessity.

A75-1. What arrangements will be made to review interim safety and efficacy data from the trial? Will a formal data monitoring committee or equivalent body be convened?

A Data Monitoring Committee (DMC) independent of the applicants and of the Trial Steering Committee (TSC) will review the progress of the trial at least twice per year and provide advice on the conduct of the trial to the TSC and (via the TSC) to the funder, sponsor and REC.

If a formal DMC is to be convened, please forward details of the membership and standard operating procedures to the Research Ethics Committee when available. The REC should also be notified of DMC recommendations and receive summary reports of interim analyses.

A75-2. What are the criteria for electively stopping the trial or other research prematurely?

In the light of interim data and emerging evidence from other studies, the DMEC will inform the Trial Steering Committee if, in their view, there is proof beyond reasonable doubt that the data indicate that any part of the protocol is indicated or contraindicated either for all infants or for a particular subgroup of trial participants.

A76. Insurance/ indemnity to meet potential legal liabilities

***Note:** in this question to NHS indemnity schemes include equivalent schemes provided by Health and Social Care (HSC) in Northern Ireland*

A76-1. What arrangements will be made for insurance and/or indemnity to meet the potential legal liability of the sponsor(s) for harm to participants arising from the management of the research? *Please tick box(es) as applicable.*

Note: Where a NHS organisation has agreed to act as sponsor or co-sponsor, indemnity is provided through NHS schemes. Indicate if this applies (there is no need to provide documentary evidence). For all other sponsors, please describe the arrangements and provide evidence.

- [ ] NHS indemnity scheme will apply (NHS sponsors only)
- [x] Other insurance or indemnity arrangements will apply (give details below)

Imperial College indemnity arrangements will apply, please see attached documentation.

Please enclose a copy of relevant documents.

A76-2. What arrangements will be made for insurance and/ or indemnity to meet the potential legal liability of the sponsor(s) or employer(s) for harm to participants arising from the design of the research? *Please tick box(es) as applicable.*

Note: Where researchers with substantive NHS employment contracts have designed the research, indemnity is provided through NHS schemes. Indicate if this applies (there is no need to provide documentary evidence). For other protocol authors (e.g. company employees, university members), please describe the arrangements and provide evidence.

- [ ] NHS indemnity scheme will apply (protocol authors with NHS contracts only)
- [x] Other insurance or indemnity arrangements will apply (give details below)

Imperial College indemnity arrangements will apply, please see attached documentation.

Please enclose a copy of relevant documents.

A76-3. What arrangements will be made for insurance and/ or indemnity to meet the potential legal liability of investigators/collaborators arising from harm to participants in the conduct of the research?

Note: Where the participants are NHS patients, indemnity is provided through the NHS schemes or through professional indemnity. Indicate if this applies to the whole study (there is no need to provide documentary evidence). Where non-NHS sites are to be included in the research, including private practices, please describe the arrangements which will be made at these sites and provide evidence.

- [x] NHS indemnity scheme or professional indemnity will apply (participants recruited at NHS sites only)
- [ ] Research includes non-NHS sites (give details of insurance/ indemnity arrangements for these sites below)

Please enclose a copy of relevant documents.

A77. Has the sponsor(s) made arrangements for payment of compensation in the event of harm to the research participants where no legal liability arises?

(●) Yes ( ) No

If Yes, please give details of the compensation policy:
Imperial College "no-fault" indemnity for clinical studies.

Please enclose a copy of relevant documents.

A78. Could the research lead to the development of a new product/process or the generation of intellectual property?

( ) Yes ( ) No ( ) Not sure

A79. Please select the level of commercial participation in this project.

( ) None
( ) Industry funding, but not industry sponsored
( ) Industry funding and industry sponsored
( ) Industry sponsored, but not industry funded

A80. Please select the main subject area of research. Additional sub-topics may be selected, if required

- [ ] Age and Ageing
- [ ] Anaesthetics
- [ ] Cancer (includes malignant haematology
- [ ] Cardiovascular
- [ ] Clinical
- [ ] Critical Care
- [ ] Dementias and Neurodegenerative Diseases
- [ ] Dermatology
- [ ] Diabetes
- [ ] Ear, Nose and Throat
- [ ] Gastrointestinal
- [ ] Genetics

- [ ] Health Services Research
- [ ] Hepatology
- [ ] Immunology and Inflammation
- [ ] Infectious Disease and Microbiology
- [ ] Injuries and Accidents
- [ ] Medicines for Children (does not include Paediatrics)
- [ ] Mental Health
- [ ] Metabolic and Endocrine
- [ ] Musculoskeletal (Rheumatoid Arthritis is a separate category)
- [ ] Nervous System Disorders
- [ ] Non-malignant Haematology
- [ ] Ophthalmology
- [ ] Oral and Dental
- [x] Paediatrics (does not include Medicines for Children)
- [ ] Primary Care
- [ ] Public Health Research
- [ ] Renal
- [ ] Reproductive Health and Childbirth
- [ ] Respiratory
- [ ] Rheumatoid Arthritis
- [ ] Stroke
- [ ] Surgery
- [ ] Urogenital

PART B: Section 7 - Children

1. Please specify the potential age range of children under 16 who will be included and give reasons for carrying out the research in this age group.

The WHEAT trial will include premature infants under 30 weeks gestation. The condition that the intervention arm in the WHEAT trial is trying to prevent, necrotising enterocolitis, is found almost exclusively in premature infants.

2. Indicate whether any children under 16 will be recruited as controls and give further details.

The WHEAT trial is a randomised trial so recruited infants (less than 30 weeks gestation) will be randomly assigned (with an equal chance) to the control or intervention arms. Both the control and intervention arms represent current clinical practice in the UK.

3-2. Please describe the arrangements for seeking informed consent from a person with parental responsibility and/or from children able to give consent for themselves.

Parents of babies that meet the inclusion criteria will be approached by members of the local clinical team after their baby is admitted to the neonatal unit, usually in the first 24 hours. Parents will be provided with the parent information sheet that explains the WHEAT trial and makes it clear that both arms (withholding feeds and continuing feeds) represent current clinical practice.

The parent information sheet will make it clear that opt-out consent is being requested. This means that if they do not want their baby to take part in the WHEAT trial they can inform the local clinical team at any point. If they do not do "opt out" in this way then their baby will be enrolled into WHEAT and will be randomised to one of the two study arms.

The local team will record in the electronic health record that they have explained the WHEAT trial and the "opt out" consent process to parents.

4. If you intend to provide children under 16 with information about the research and seek their consent or agreement, please outline how this process will vary according to their age and level of understanding.

This is not applicable, infants eligible for WHEAT are too young (premature babies).

Copies of written information sheet(s) for parents and children, consent/assent form(s) and any other explanatory material should be enclosed with the application.
