# Supplementary material for: Research ethics committee decision-making in relation to an efficient neonatal trial
Source: Arch Dis Child Fetal Neonatal Ed. 2016 Sep 14;102(4):F291–8. doi: 10.1136/archdischild-2016-310935 (PMC5537508; doi:10.1136/archdischild-2016-310935)
Supplement: Supplementary data [file fetalneonatal-2016-310935supp002.pdf]

The WHEAT Study (With-Holding Enteral feeds Around Transfusion)

We understand this is a difficult time and it may not seem a good moment to ask you to think about something extra, but we want you to know about an important study for premature babies (babies born before their due date).

What is the purpose of the WHEAT study?
It is quite common for premature babies to have blood transfusions because they become anaemic (they do not have enough red blood cells). Premature babies are also vulnerable to a bowel condition called necrotising enterocolitis (NEC). This can be serious and can have long-term effects on how babies grow and develop. Some doctors worry that feeding babies during a blood transfusion may increase the risk of NEC, others however, think that it is more risky to stop feeds; the problem is that we do not know. Because of this, the way babies are cared for during blood transfusions varies across the country; some babies have feeds stopped before, during and after a transfusion (around 12 hours in total), and others have feeds continued. The purpose of WHEAT is to determine which approach is best. WHEAT is taking place in neonatal units all over the UK and will involve about 4,500 babies.

Why has my baby been chosen?
All babies that are born more than 10 weeks before their due date can take part.

Does my baby have to take part?
We are comparing practices that already take place in neonatal units in the UK and are offering every baby the opportunity to participate. Your baby does not have to take part if you don't want them to, in this case please tell a member of the local clinical team (names and contact details are provided at the end of this leaflet) that you would like to "opt-out" (have your baby excluded from the WHEAT study).

If you do want your baby to take part in WHEAT, you don't need to do anything.

What will happen if I opt-out?
If you "opt-out" your baby will still have feeds either stopped or continued during transfusions in the same way as in WHEAT but the decision will be made by the local clinical team and the policy of the neonatal unit, and information about your baby will not be included in the study.

What will happen if I do not opt-out (agree to my baby participating in WHEAT)?
If your baby needs a blood transfusion, and is receiving milk feeds, the decision about whether to stop or continue feeds during the transfusion will be decided by a process called "randomisation". Randomisation is done by computer and means that every baby has an equal chance of either having feeds stopped or continued. If your baby is randomised to have feeds stopped this will be for 4 hours before, after, and during this and any subsequent blood transfusions. It is quite common for premature babies to have their feeds withheld for a number of reasons. When this happens babies are given nutrition into a vein by drip to ensure their blood sugar level does not drop and to reduce any feelings of hunger they might have. Babies in WHEAT who have their milk feeds stopped around a blood transfusion will be given nutrition into a vein in the same way. If your baby is randomised to have feeds continued, there will be no change in how your baby is fed.

Babies in WHEAT will not have any extra tests and in all other respects will be looked after in the same way as a premature baby not taking part in the study.

Will my baby definitely have a blood transfusion?
Not all premature babies need a blood transfusion, but many do and some need more than one. Babies in WHEAT will have no more and no less than other premature babies.

Are there any risks for my baby?
There are no risks for your baby from taking part in WHEAT.

Are there any benefits for my baby?
Each of the two options in the WHEAT study is currently used by doctors in the UK because we do not know which one is better. For babies not taking part in WHEAT, the choice of whether or not to stop feeds is made according to the preference of the local medical team. This non-evidence based approach to neonatal care may involve more risk than being in a study like WHEAT which involves a carefully designed protocol and consistent monitoring.

What if relevant new information becomes available?
If new information becomes available during the study this will be evaluated by an independent Data Monitoring Committee who will advise whether or not WHEAT should continue.

Will my taking part in WHEAT be kept confidential?
We will record relevant information about you and your baby; this will be kept securely and will only be seen by the study team and authorized regulatory authorities. You and your baby will not be identified in any report or publication about WHEAT.

What will happen to the results of WHEAT?
The results will be used to improve the way premature babies are looked after. The results will be published in a medical journal. We will also send a copy of the results to you.

Who is managing and funding WHEAT?
WHEAT is managed by the Clinical Trials Unit at the University of Manchester. It is funded by the United Kingdom Medical Research Council.

Who has reviewed WHEAT?
This study has been reviewed by National Institute for Health Research and a national Research Ethics Committee, an independent group of people who ensure the study is ethical and conducted properly. This study has been approved by the XXXXX Research Ethics Committee.

What if there is a problem?
If at any stage you have any questions about the study or the way it has been carried out, please contact the Local Principal Investigator or Local Research Nurse (contact details below).

What if I change my mind and decide to withdraw my baby from WHEAT at a later date?
You can change your mind and opt-out of WHEAT at any time and without having to give a reason. Just let us know.

Thank you for reading this leaflet; please discuss WHEAT with the doctor or nurse who is looking after your baby if you have any questions.

**Local Principal Investigator**	**Local Research Nurse**	**Lead Investigators**
		Professor Neena Modi &<br>Dr Chris Gale<br>Imperial College London,<br>0203 315 5101<br>n.modi@imperial.ac.uk<br>christopher.gale@imperial.ac.uk
