# Supplementary material for: Research ethics committee decision-making in relation to an efficient neonatal trial
Source: Arch Dis Child Fetal Neonatal Ed. 2016 Sep 14;102(4):F291–8. doi: 10.1136/archdischild-2016-310935 (PMC5537508; doi:10.1136/archdischild-2016-310935)
Supplement: Supplementary data [file fetalneonatal-2016-310935supp003.pdf]

Health Research Authority

27 November 2014

Dr Chris Gale
NIHR Clinical Lecturer
Imperial College London
Section of Academic Neonatal Medicine,
Imperial College London, Chelsea and Westminster Campus,
369 Fulham Road, London
SW10 9NH

Dear Dr Gale,

**Study title:**	**The WHEAT trial: WithHolding Enteral feeding Around packed red cell Transfusions in preterm neonates, a multicentre, superiority, randomised registry trial**
**REC reference:**	
**Protocol number:**	
**IRAS project ID:**	

The Research Ethics Committee reviewed the above application at the meeting held on

We plan to publish your research summary wording for the above study on the HRA website, together with your contact details, unless you expressly withhold permission to do so. Publication will be no earlier than three months from the date of this favourable opinion letter. Should you wish to provide a substitute contact point, require further information, or wish to make a request to postpone publication, please contact the REC Manager.

Ethical opinion

The members of the Committee present gave a favourable ethical opinion of the above research on the basis described in the application form, protocol and supporting documentation, subject to the conditions specified below.

Conditions of the favourable opinion

The favourable opinion is subject to the following conditions being met prior to the start of the study:

Application

1. Please amend the IRAS form (A 61) and study protocol, if necessary, to refer to greater, or less than, or equal to, the 10th centile, in order to include this group in the study analysis.

PIS

2. In the section ‘Are there any benefits for my baby’ please delete the third sentence starting ‘This non-evidence based approach …’

3. Please amend the section ‘Are there any risks for my baby’ to state ‘There are minimal risks for your baby from taking part equivalent to those in the use of a standard care approach’.

4. Please amend the section ‘Why has my baby been chosen’ to begin ‘Your baby has been chosen because …’

5. Please amend the section ‘Who has reviewed WHEAT’ to state ‘… and the Berkshire Research Ethics Committee …’. Please then delete the last sentence of this section.

6. ’

You should notify the REC in writing once all conditions have been met (except for site approvals from host organisations) and provide copies of any revised documentation with updated version numbers. The REC will acknowledge receipt and provide a final list of the approved documentation for the study, which can be made available to host organisations to facilitate their permission for the study. Failure to provide the final versions to the REC may cause delay in obtaining permissions.

Management permission or approval must be obtained from each host organisation prior to the start of the study at the site concerned.

Management permission (“R&D approval”) should be sought from all NHS organisations involved in the study in accordance with NHS research governance arrangements.

Guidance on applying for NHS permission for research is available in the Integrated Research Application System or at http://www.rdforum.nhs.uk.

Where a NHS organisation’s role in the study is limited to identifying and referring potential participants to research sites (“participant identification centre”), guidance should be sought from the R&D office on the information it requires to give permission for this activity.

For non-NHS sites, site management permission should be obtained in accordance with the procedures of the relevant host organisation.

Sponsors are not required to notify the Committee of approvals from host organisations.

Registration of Clinical Trials

All clinical trials (defined as the first four categories on question 2 of the IRAS filter page) must be registered on a publically accessible database within 6 weeks of recruitment of the first participant (for medical device studies, within the timeline determined by the current registration and publication trees).

There is no requirement to separately notify the REC but you should do so at the earliest opportunity e.g. when submitting an amendment. We will audit the registration details as part of the annual progress reporting process.

To ensure transparency in research, we strongly recommend that all research is registered but for non-clinical trials this is not currently mandatory.

If a sponsor wishes to contest the need for registration they should contact , the HRA does not, however, expect exceptions to be made. Guidance on where to register is provided within IRAS.

It is the responsibility of the sponsor to ensure that all the conditions are complied with before the start of the study or its initiation at a particular site (as applicable).

Ethical review of research sites

NHS Sites

The favourable opinion applies to all NHS sites taking part in the study taking part in the study, subject to management permission being obtained from the NHS/HSC R&D office prior to the start of the study (see “Conditions of the favourable opinion” below).

Non NHS sites

The Committee has not yet completed any site-specific assessment(s) (SSA) for the non-NHS research site(s) taking part in this study. The favourable opinion does not therefore apply to any non-NHS site at present. I will write to you again as soon as an SSA application(s) has been reviewed. In the meantime no study procedures should be initiated at non-NHS sites.

Summary of discussion at the meeting

Social or scientific value; scientific design and conduct of the study

- The Committee considered whether it would be more appropriate to conduct a cluster randomisation design, rather than a patient randomisation trial considering the potential difficulties of using both practices within the same centre, particularly given that the applicants do not propose to take individual, opt in, consent. However the Committee noted that a cluster methodology would give rise to statistical weakness. Moreover, noting that both practices are currently used within the same centres the Committee concluded that its initial concerns about the trial design and management had been addressed.

- The Committee noted that, at IRAS A61 you discuss greater than, ***and*** less than, the 10th centile. The Committee noted that you should amend one of these to be greater, or less than, or equal to, so as to include the 10th centile in the study analysis.

- The Committee questioned whether the study was truly in equipoise noting that some two thirds of units currently feed babies. The Committee questioned this predominance of one practice over another; it expected a 50/50 distribution to reflect true equipoise. After further discussion it concluded that

neither practice had an evidence base so there was no need to doubt equipoise.

- The Committee noted that your answer to IRAS A20 could be considered to be inaccurate as at least some babies would not be receiving standard care (albeit lacking an evidence base) as a result of randomisation. The Committee concluded that this inaccuracy raised no ethical issues that had not been considered in the overall design of the trial.

- The Committee questioned whether the feed used in the study would be standardised. Whilst it concluded that, it would be mixed to meet the needs of the individual baby, it observed that it would nevertheless follow a standard formulation.

- The Committee questioned why you did not exclude those individuals participating in other research trials, particularly drug trials, the activity of which may influence the results of this study. Considering the number of existing potentially confounding factors the Committee considered it would be best to exclude any individual participating in another study. After further discussion and following the advice of expert members, the Committee concluded that exclusion of all participants in other trials would result in a bias given the preponderance of research in neonatal units.

Recruitment arrangements and access to health information, and fair participant selection

- The Committee recognised the unusual strategy – linked to NNRD and involving ‘opt out’ consent, but had no ethical concerns in this area regarding the proposed study.

Favourable risk benefit ratio; anticipated benefit/risks for research participants (present and future)

- The Committee was initially concerned about your proposal to withhold feeding from babies for up to 12 hours. It was concerned that the babies would become hungry, and therefore distressed, due to this. The Committee balanced its concerns by noting that initial treatment for Necrotising Enderocolitis (NEC) is withdrawal of feeding for up to 12 hours. The Committee’s concerns were further assuaged by the advice of two expert members both of whom were clear that enteral, as opposed to intravenous, feeding was rare in neonatal units notwithstanding the burdens of this particular trial. The Committee considered the possibility of collecting data to measure babies’ stress (resulting from hunger) levels as a secondary outcome measure. The Committee considered this to be particularly important as if the study shows only a small benefit of one practice over another it may be necessary to conclude that a small reduction in the incidence of NEC might not be acceptable if balanced against increased stress levels in babies. The Committee resolved to take no further action on the advice of the expert member who noted that hunger was an ‘unknown’ in the neonatal unit and could not be distinguished from other stressors.

Informed consent process and the adequacy and completeness of participant information

- The Committee’s main attention was devoted to the issue of ‘opt out’ consent and, after considerable debate, it agreed that it was both defensible and preferable to more typical approaches; the Committee commended the strategy. It concurred with you that, considering the number of participants to be recruited, the sensitivity of parents situation and the fact that both practices are currently used in routine care, with limited evidence base, ‘opt out’ consent was appropriate.

- The Committee questioned why you needed to undertake the study and why you could not simply conduct a review of retrospective data. It noted however, that to do so, would mean that the care of babies whose data were included in the study, would not be standardised, thus introducing potentially confounding factors into the research.

Suitability of supporting information

- The Committee sought confirmation that all information sheets and other documentation would be presented to participants on appropriately headed paper.

- The Committee noted that the PIS states that there is a risk to babies of not entering the trial and there will be some ‘inclusion benefit’ as a result of participation. While concluding that the risk of a non-evidence based healthcare approach could be used to justify the ‘opt out’ consent model the Committee concluded that it should not be presented to participants as a danger of not participating, due to the coercive effect that this could have.

Other general comments

- The Committee noted that you discuss in the application that you will inform participants of the results of the study. Noting that you would have to write to 4500 sets of parents the Committee was unclear how feasible this would be. Furthermore, the Committee questioned what procedures you had in place to ascertain whether babies are still alive before contacting the parents. After some discussion the Committee concluded that there were no significant ethical issues resulting from informing participants of the results (general) of the study. Whilst you were inconsistent with regard to your strategy to inform parents, the Committee was content to leave the matter to your judgement.

- The Committee complemented you for the proper use of a registry, noting that you intended to use information from, and add to, an existing registry, rather than set up a new one.

Suitability of the summary of the research

- The Committee assessed your answer to IRAS A6-1 and considered that it was suitable for publication on the HRA website.

Approved documents

The documents reviewed and approved at the meeting were:

Document	*Version*	*Date*
Covering letter on headed paper		05 September 2014

Evidence of Sponsor insurance or indemnity (non NHS Sponsors only)		05 September 2014
Letter from sponsor		05 September 2014
Participant information sheet (PIS)		02 September 2014
REC Application Form [REC_Form_10092014]		10 September 2014
Research protocol or project proposal		11 August 2014
Summary CV for Chief Investigator (CI)	1	05 September 2014

Membership of the Committee

The members of the Ethics Committee who were present at the meeting are listed on the attached sheet.

After ethical review

<u>Reporting requirements</u>

The attached document “After ethical review – guidance for researchers” gives detailed guidance on reporting requirements for studies with a favourable opinion, including:

- Notifying substantial amendments
- Adding new sites and investigators
- Notification of serious breaches of the protocol
- Progress and safety reports
- Notifying the end of the study

The HRA website also provides guidance on these topics, which is updated in the light of changes in reporting requirements or procedures.

User Feedback

The Health Research Authority is continually striving to provide a high quality service to all applicants and sponsors. You are invited to give your view of the service you have received and the application procedure. If you wish to make your views known please use the feedback form available on the HRA website: http://www.hra.nhs.uk/about-the-hra/governance/quality-assurance/

HRA Training

We are pleased to welcome researchers and R&D staff at our training days – see details at http://www.hra.nhs.uk/hra-training/

Please quote this number on all correspondence

With the Committee's best wishes for the success of this project.

Yours sincerely,

Chair

E-mail:

Enclosures:	*List of names and professions of members who were present at the meeting and those who submitted written comments*
	After ethical review – guidance for researchers
Copy to:	*Chelsea and Westminster NHS Foundation Trust*

NRES Committee South Central - Berkshire

Attendance at Committee meeting on 18 November 2014

Committee Members:

Name	*Profession*	*Present*	*Notes*
	Social Scientist	Yes	
	Pharmaceutical Consultant	Yes	
	Retired Midwife and Clinical Governance Manager	Yes	
	R&D Research Co-ordinator	Yes	
	Retired Corporate Lawyer	Yes	
	Director	Yes	
	Aviation Safety Consultant	Yes	
	Consultant Paediatrician	Yes	
	Co-ordinator for QA in Research	Yes	
	Head of the School of Health and Social Care	Yes	
	Lead Pharmacist for Elderly Care, Neuro-rehabilitation, Dermatology and Clinical Governance	Yes	
	Medical Director	Yes	
	Senior Research Support Associate	Yes	

Also in attendance:

Name	*Position (or reason for attending)*
	REC Manager
	Observer
	Observer
	Observer

Imperial College London

Section of Neonatal Medicine
Imperial College London

4th Floor, Lift Bank D
Chelsea and Westminster Hospital
369 Fulham Road
London, SW10 9NH
Tel: +44 (0)203 3153519
Fax: +44 (0)203 3157998

christopher.gale@imperial.ac.uk

12th January 2015

Dr Christopher Gale MBBS MSc PhD MRCPCH

Dear,

**Study title:**	**The WHEAT trial: With Holding Enteral feeding Around packed red cell Transfusions in preterm neonates, a multicentre, superiority, randomised registry trial**
**REC reference:**	
**Protocol number:**	
**IRAS project ID:**	

Thank you for taking the time to review the WHEAT trial, please find attached the revised trial documents reflecting the conditions for the favourable opinion.

Application
1. Please amend the IRAS form (A 61) and study protocol, if necessary, to refer to greater, or less than, or equal to, the 10th centile, in order to include this group in the study analysis.
   - The IRAS form (A61) and the protocol (page 15) have been modified to read: “Birth weight centile for gestational age: ≥10th centile OR <10th centile” in order to include the 10th centile group.

PIS
2. In the section ‘Are there any benefits for my baby’ please delete the third sentence starting ‘This non-evidence based approach …’
   - This has been deleted.

3. Please amend the section ‘Are there any risks for my baby’ to state ‘There are minimal risks for your baby from taking part equivalent to those in the use of a standard care approach’.
   - This has been amended.

4. Please amend the section ‘Why has my baby been chosen’ to begin ‘Your baby has been chosen because …’
   - This has been amended.

5. Please amend the section ‘Who has reviewed WHEAT’ to state ‘… and the Berkshire Research Ethics Committee …’. Please then delete the last sentence of this section.
   - This has been amended.

I hope these responses provide sufficient clarification, please do not hesitate to contact us if you require any further information.

Documents attached:

Document	Version	Date
Participant Information Sheet	1.4	12 January 2015
Protocol	1.4	12 January 2015
IRAS Form		12 January 2015

Yours Sincerely,

Dr Chris Gale
NIHR Clinical Lecturer in Paediatrics

Health Research Authority

15 January 2015

Dr Chris Gale
NIHR Clinical Lecturer
Imperial College London
Section of Academic Neonatal Medicine,
Imperial College London, Chelsea and Westminster Campus,
369 Fulham Road, London
SW10 9NH

Dear Dr Gale,

Study title: **The WHEAT trial: WithHolding Enteral feeding Around packed red cell Transfusions in preterm neonates, a multicentre, superiority, randomised registry trial**
REC reference:
Protocol number:
IRAS project ID:

Thank you for your letter of 12 January 2015. I can confirm the REC has received the documents listed below and that these comply with the approval conditions detailed in our letter dated 01 December 2014

Documents received

The documents received were as follows:

Document	*Version*	*Date*
Other [Response Letter]	1	12 January 2015
Other [Updated PIS]	1.4	12 January 2015
Other [Supporting Reference]	1	12 January 2015
Other [Amended Protocol]	1.4	12 January 2015

Approved documents

The final list of approved documentation for the study is therefore as follows:

Document	*Version*	*Date*
Covering letter on headed paper		05 September 2014

Evidence of Sponsor insurance or indemnity (non NHS Sponsors only)		05 September 2014
Letter from sponsor		05 September 2014
Other [Response Letter]	1	12 January 2015
Other [Updated PIS]	1.4	12 January 2015
Other [Supporting Reference]	1	12 January 2015
Other [Amended Protocol]	1.4	12 January 2015
REC Application Form [REC_Form_10092014]		10 September 2014
Summary CV for Chief Investigator (CI)	1	05 September 2014

You should ensure that the sponsor has a copy of the final documentation for the study. It is the sponsor's responsibility to ensure that the documentation is made available to R&D offices at all participating sites.

Please quote this number on all correspondence

Yours sincerely,

Copy to: *Chelsea and Westminster NHS Foundation Trust*
