# Supplementary material for: Research ethics committee decision-making in relation to an efficient neonatal trial
Source: Arch Dis Child Fetal Neonatal Ed. 2016 Sep 14;102(4):F291–8. doi: 10.1136/archdischild-2016-310935 (PMC5537508; doi:10.1136/archdischild-2016-310935)
Supplement: Supplementary data [file fetalneonatal-2016-310935supp004.pdf]

REC Application Review and Advice Proforma

Study Title: **The WHEAT trial: WithHolding Enteral feeding Around packed red cell Transfusions in preterm neonates, a multicentre, superiority, randomised registry trial**			
REC Reference Number:		Name of Chief Investigator:	Dr Chris Gale
Date proforma submitted to applicant	24/9/2014	Date proforma returned by applicant:	

Please use the applicant response column to provide written clarification to the points raised and to indicate whether additional or revised documents have been submitted. This proforma should be emailed to the REC Manager by **29th September 2014.** Please note the advice on this document is advice provide by the REC Manager and one Committee member only. **The comments on this document do not form an ethical opinion.** The Research Ethics Committee will review and discuss the application at the meeting and will confirm the ethical opinion in writing within 10 working days of the meeting date. **The Committee may request further clarifications and revisions to supporting documents following the meeting.**

1. General Advice – Requests for further information/clarification

Issue identified	***Applicant response***
Consent opt-out	Opt out consent will be recorded on the BadgerNet or Neonatal.net data systems which are used in all Neonatal Units in England and Wales to collect daily clinical data on all babies in their care. It is managed by CleverMed. The outcome data is retrieved from the same system and it will be a requirement for unit participation in our study that they are using this data system. We cannot provide a screenshot of the data collection screen as we that will have to be done by CleverMed. This will involve a formal application to them after obtaining ethics approval. We have already been in dialogue with them, and this is simply a formality. We confirm that we will not start the study without this being in place, and would be happy to submit a screenshot as an amendment to the committee before starting the study. We have previously collected study data in this manner, and have modified the data collection interface in use on units to do so. We have not previously recorded opt out

	consent in this manner. We have one other study running in preterm infants which is using the opt-out consent design, the PREMFOOD study; like the WHEAT trial, this study is a comparison of two routine practices in neonatal care.
Has permission been obtained to extract data from National Neonatal Research Database (NNRD)?	Yes, we have permissions in place to collect the data from the NNRD, which is run by a team headed by Professor Neena Modi, a principal investigator on the WHEAT study, and the database is hosted at Imperial College London.
No sites listed in part C of NHS REC form	We hope that WHEAT will be carried out in neonatal units across the UK. We did not upload any research sites in Section C because they are not finalised as yet. We are currently applying for funding for the study, and although we have been in consultation with representatives of neonatal units throughout the country and 97 English neonatal units have agreed to take part, they have not formally signed up yet. For this reason we plan to submit the required SSI forms at a later date, as the IRAS website suggests is acceptable. The research will be hosted at Imperial College who are sponsors for the research.
Who provided scientific review – no details given in A54	The study rationale was based on a large-scale questionnaire exercise to every neonatal unit in the country. Many of the lead consultants not only replied to the questionnaire, but commented on the design of the study. The team organising the study is listed in the application, A63, and are clearly a multi-disciplinary team, with input from patient charities and PPI participation. Within the research team based at Imperial College it has been reviewed and commented on by our clinical research team, the non-clinical, basic science researchers, our two in group statisticians and the data analysis team based at NDAU, the Neonatal Data Analysis Unit.
Are any details of the DMC and TSC, such as the charter and composition available?	Memberships of the DMC and TSC have not been finalised yet. The planned composition is as follows:<br>TSC: Independent chair - to be appointed, Dr Chris Gale, Prof Neena Modi, Prof Tjeerd-Pieter van Staa, Dr Mark Turner, Dr Jon Dorling, Helen Robberts, Zoe Chivers, Amanda Forster, Trial Statistician – to be appointed, Independent member - to be appointed, Independent member - to be appointed,<br>DMC: Independent chair - to be appointed, Trial statistician - to be appointed, Neonatal clinician - to be appointed, Neonatal clinician - to be appointed, Independent statistician - to be appointed

2. Suggested changes/additions to supporting documents

Description of suggestions	***Applicant response – Please indicate if revised documents have been provided and ensure the changes are tracked or highlighted***
PIS – use of the word 'important' in opening sentence.	We would be happy to remove this word, if that is what the committee require.
PIS – risk from additional (or second) IV line.	We expect the number of babies requiring a second IV line to be minimal. We have not described the "risk" of the second iv line because it is not a "risk" related to the research; it represents an accepted variation in clinical practice that is routine in approximately 1 in 3 neonatal units in the UK (in these units, where withholding feeds around transfusion is standard, inclusion in WHEAT may actually involve the "benefit" of no second IV line). Currently all babies born in the UK are exposed to this "risk" but it is based upon clinician preference and unit of birth.
Should there be a poster to go in the ward to increase awareness?	We have not developed a poster describing the WHEAT trial, but agree that a poster would be of value in raising awareness about the WHEAT trial and the opt-out consent process.

Signed by REC Chair: ………………………………………………………………….   Date……………………………………………………

15 October 2014

Dr Chris Gale
NIHR Clinical Lecturer
Imperial College London
Section of Academic Neonatal Medicine
Imperial College London
Chelsea and Westminster Campus
369 Fulham Road
London
SW10 9NH

Dear Dr Gale

**Study Title:**	**The WHEAT trial: WithHolding Enteral feeding Around packed red cell Transfusions in preterm neonates, a multicentre, superiority, randomised registry trial**
**REC reference:**	
**Protocol number:**	
**IRAS project ID:**	

The Research Ethics Committee reviewed the above application at the meeting held on . Please thank Dr Matthew Hyde for attending to discuss the application.

Provisional opinion

The Committee would be content to give a favourable ethical opinion of the research, subject to receiving a complete response to the request for further information set out below.

Authority to consider your response and to confirm the Committee's final opinion has been delegated to a meeting of the Sub-committee of the REC.

Further information or clarification required

1. Please clarify why twenty-four hours has been chosen as the time-frame for families to be told about the study.

2. Please confirm that the staff involved on the units will be fully aware of the study and trained to take part. Please consider producing a leaflet to increase awareness among staff members.

3. Please provide the full composition of the data monitoring committee. Please clarify why it only meets twice a year and if there are any stopping parameters.

4. Please provide a Poster to be put up in neonatal wards.

5. Please amend the sentence 'This non-evidence based approach to neonatal care may involve more risk than being in a study like WHEAT which involves a carefully designed protocol and consistent monitoring.' under the heading 'Are there any benefits for my baby?' as it could be considered coercive.

If you would find it helpful to discuss any of the matters raised above or seek further clarification from a member of the Committee, you are welcome to contact .

When submitting a response to the Committee, the requested information should be electronically submitted from IRAS. A step-by-step guide on submitting your response to the REC provisional opinion is available on the HRA website using the following link: http://www.hra.nhs.uk/nhs-research-ethics-committee-rec-submitting-response-provisional-opinion/

Please submit revised documentation where appropriate underlining or otherwise highlighting the changes which have been made and giving revised version numbers and dates. You do not have to make any changes to the REC application form unless you have been specifically requested to do so by the REC.

The Committee will confirm the final ethical opinion within a maximum of 60 days from the date of initial receipt of the application, excluding the time taken by you to respond fully to the above points. A response should be submitted by no later than .

Summary of the discussion at the meeting

Social or scientific value; scientific design and conduct of the study

The committee stated that this was a very interesting study.

The committee noted that the study proposed to use an opt-out consent process and this might result in neonatal units who would normally feed babies through any blood transfusions having to stop feeds and so insert additional IV lines. The committee what would happen if the unit said they didn't want to take part as it would be a change in their normal procedures or the parents said no because it was a change to the normal care in that unit.

Dr Hyde stated they will explain to families that approximately a third of units withhold feeds at the current time, however there is no evidence which is better. Both feeding and withholding feeding are used as standard of care and what you get depends on the whim of the treating physician. This study is a comparative effectiveness study so they are being randomised to standard of care whichever arm they go into. Dr Hyde stated that the study is part of a move towards evidence based care.

The committee queried if this would all be explained to the unit.

Dr Hyde stated he would presume that this would all be explained to the unit and they would receive training in it. Dr Hyde confirmed that a baby would only be randomised into the study once the parent had said they did not want to opt-out. Dr Hyde stated they are currently using an opt-out consent methodology in another study which is working well. Dr Hyde stated they are happy to provide screen-shots of the BadgerNet system to show that they won't be able to randomise until they know the family does not want to opt-out.

<u>Recruitment arrangements and access to health information, and fair participant selection</u>

The committee noted in private discussion that interpreters would be provided where needed.

The committee noted in private discussion that the study used an opt-out consent method.

The committee noted that all families would approached and the study explained to them within twenty-four hours. The committee stated this is a very stressful time for the family and they know that the majority of information they get in this time does not sink in and so queried if this time could be extended to seventy-two hours.

Dr Hyde stated he would need to check with Dr Gale whether they could extend this time frame. However he knows it is national policy for the family to be seen by someone senior from the department within twenty-four hours and they felt this would be the ideal time to explain the study. Dr Hyde stated if the study involved an intervention then the family would be given more than twenty-four hours.

The committee stated that the chance of a premature baby being milk fed in the first twenty-four hours would be miniscule so they don't feel it would affect the study if this time was increased.

The committee noted that a block randomisation would be used and queried if any confounding factors would be considered.

Dr Hyde stated that all the data entered into BadgerNet or neonatal.net would be available so they can consider other confounding factors. However they are concerned over the quality of these other data points so are initially planning to restrict the study to factors they are sure they will get good quality date on.

<u>Care and protection of research participants; respect for potential and enrolled participants' welfare and dignity</u>

The committee noted that the data monitoring committee only meets twice a year and queried if it should be more frequently bearing in mind the size of the trial. The committee also queried if there were any stopping parameters.

Dr Hyde stated he was unsure and would need to check with Dr Gale.

<u>Informed consent process and the adequacy and completeness of participant information</u>

The committee stated that the statement 'This non-evidence based approach to neonatal care may involve more risk than being in a study like WHEAT which involves a carefully designed protocol and consistent monitoring.' in the Participant Information Sheet could be considered coercive and should be removed.

Dr Hyde stated there has recently been some research published in the Lancet that looked at wasted research. This has shown that even being in a control or placebo arm of a study means you receive better care than being outside of a research study. As such he feels strongly feel this statement should be left in, however they could revise the way it is phrased if the committee wanted this.

<u>Suitability of supporting information</u>

The committee stated they feel a poster should be developed to be put up in wards to increase awareness of the study. They also commented that it may be helpful to produce a leaflet for staff members.

Other general comments

The committee noted in private discussion that the PPI involvement in the study was very clear and this was a good thing that the study was meeting an identified need.

The committee queried if the study was funded yet.

Dr Hyde stated they are still waiting on this, the initial application failed at the preliminary point so they have reapplied.

Documents reviewed

The documents reviewed at the meeting were:

Document	*Version*	*Date*
Covering letter on headed paper		05 September 2014
Evidence of Sponsor insurance or indemnity (non NHS Sponsors only) [Arthur J. Gallagher International]		21 July 2014
Letter from sponsor [Imperial College]		21 August 2014
Participant information sheet (PIS)	1.3	30 August 2014
REC Application Form	3.5	07 September 2014
Research protocol or project proposal	1.3	11 August 2014
Summary CV for Chief Investigator (CI) [Christopher Gale]		

Membership of the Committee

The members of the Committee who were present at the meeting are listed on the attached sheet

Statement of compliance

The Committee is constituted in accordance with the Governance Arrangements for Research Ethics Committees and complies fully with the Standard Operating Procedures for Research Ethics Committees in the UK.

**Please quote this number on all correspondence**

Yours sincerely

Enclosures: *List of names and professions of members who were present at the meeting and those who submitted written comments.*

Copy to: Imperial College London

Chelsea and Westminster NHS Foundation Trust

Attendance at Committee meeting

Committee Members:

Name	*Profession*	*Present*	*Notes*
	Consultant Intensivist	No	
	Executive Producer and Media Consultant	No	
	Retired Civil Servant	Yes	
	Neonatal registrar	Yes	
	Clinical Professor of Children and Young People's Cancer Care	Yes	Chaired the meeting
	Pharmacist	No	
	Clinical Research Fellow	No	
	Statistician	Yes	
	Consultant in Clinical Pharmacology	No	
	Lay member - Hospital Chaplain	No	
	Retired Clinical Data Management Manager	Yes	
	Lecturer in Management Studies	Yes	
	Health Psychologist	No	
	Clinical Trials Manager	Yes	

Also in attendance:

Name	*Position (or reason for attending)*
	REC Assistant
	REC Manager

Imperial College London

Section of Neonatal Medicine
Imperial College London

4th Floor, Lift Bank D
Chelsea and Westminster Hospital
369 Fulham Road
London, SW10 9NH
Tel: +44 (0)203 3153519
Fax: +44 (0)203 3157998

christopher.gale@imperial.ac.uk

31st October 2014

Dr Christopher Gale MBBS MSc PhD MRCPCH

Dear

Study title: **The WHEAT trial: With Holding Enteral feeding Around packed red cell Transfusions in preterm neonates, a multicentre, superiority, randomised registry trial**

REC reference:
Protocol number:
IRAS project ID:

Thank you for taking the time to review the WHEAT trial. Please find responses to your requests for further information detailed below:

1. Please clarify why twenty-four hours has been chosen as the time-frame for families to be told about the study.

The opt-out consent process that we intend to use reflects our wish to help parents appreciate that WHEAT is a comparison of treatments already in accepted use rather than an evaluation of a new or experimental therapy. As such there should be no pressurised time limit for parents to decide whether or not to take part. Instead we aim to achieve a continuing dialogue about the way in which we seek to reduce widespread uncertainties in clinical care.
We empower parents with full ability to opt-out at any time during their baby's neonatal unit stay. The initial approach (in the first 24-48 hours) will be a simple explanation of the study.
Prior to giving each blood transfusion, in accordance with the principles of Good Clinical Practice in Clinical Trials, the local research team will confirm that parents are happy to continue participation.

We have chosen to introduce WHEAT early for the following reasons:

- *Many blood transfusions on a neonatal unit are given urgently or as an emergency (they are commonly given out of hours), therefore delaying discussion until a transfusion is required will shorten the period over which parents are able to consider and reflect on their decision (because the transfusion is clinically indicated and not readily delayed), and result in many parents not being offered the choice to participate in WHEAT (because they may be difficult to contact at short notice when a transfusion is indicated).*
- *Infants often need a blood transfusion when they are (or appear to be) more unwell; they have symptoms such as problems breathing or a fast heart rate. This is often a very stressful and difficult time for parents. Delaying discussion of WHEAT until this point would be more emotionally charged and stressful than explaining it in the first 24-48 hours and allowing parents time to fully consider and reflect on their baby's involvement.*

2. Please confirm that the staff involved on the units will be fully aware of the study and trained to take part. Please consider producing a leaflet to increase awareness among staff members.

Staff involved in the WHEAT trial will be made aware of the study through the NIHR Children's Research Network and local research nurses, co-ordinated via the Clinical Trials Unit. There exists a high level of expertise and training in relation to research across UK neonatal units: research is integral to neonatal care and participation in neonatal research studies is almost universal among UK neonatal units (151 neonatal units recruited into studies associated with the NIHR Neonatal Clinical Studies Group, 2011-2014 www.odp.nihr.ac.uk). For doctors in training research training is a core paediatric competency (RCPCH). In addition we will ensure that local research nurses and local investigators have undergone Good Clinical Practice training, this will be co-ordinated by the Clinical Trials Unit.

A pragmatic comparative effectiveness trial like WHEAT does not have complex protocols or pathways and we therefore feel that a leaflet would not be appropriate.

3. Please provide the full composition of the data monitoring committee. Please clarify why it only meets twice a year and if there are any stopping parameters.

As outlined in in the protocol and in the REC form, the data monitoring committee (DMC) will be established before recruitment starts. The proposed composition of the DMC is outlined in the protocol and follows advice from the DAMOCLES Study Group (HTA 2005). The names of the members of the DMC will be provided to the REC when finalised). In accordance with the guidance of the DAMOCLES Study Group the DMC will establish a Charter at their initial meeting that will formalise the terms of reference of the DMC. The DMC will be expected to meet at least 6 monthly with a planned interim analysis after 12 months of recruitment; this will be outlined in the DMC charter and the final decision regarding the number and timing of meetings will be at the discretion of the DMC. The point at which recruitment would be stopped will be determined by the DMC and in line with the DAMOCLES statement: "Statistical issues should be only one of several considerations that a DMC needs to take into account. Other considerations include the balance of primary risks and benefits, the internal consistency of results, the consistency with, and nature of, external evidence, and the likelihood that the results would affect clinical practice." Statistical criteria will be determined by the DMC at their initial meeting and clearly recorded in the DMC Charter (a copy of which will be provided to the REC when finalised) but these will be "regarded as guidelines for recommending stopping rather than rules" (DAMOCLES, Lancet 2005).

4. Please provide a Poster to be put up in neonatal wards.

Please find a copy of the poster attached.

5. Please amend the sentence 'This non-evidence based approach to neonatal care may involve more risk than being in a study like WHEAT which involves a carefully designed protocol and consistent monitoring.' under the heading 'Are there any benefits for my baby?' as it could be considered coercive.

We acknowledge the committee's point regarding the wording and have replaced the statement "This non-evidence based approach to neonatal care may involve more risk than being in a study like WHEAT which involves a carefully designed protocol and consistent monitoring" with "taking part in a research study may confer non-specific benefits" (changes highlighted in the Participant Information Sheet).

I hope these responses provide sufficient clarification, please do not hesitate to contact us if you require any further information.

Documents attached:

Document	Version	Date
Participant Information Sheet	1.4	31 October 2014
WHEAT trial poster	1.0	31 October 2014
DAMOCLES, HTA		2005
DAMOCLES, Lancet		2005

Yours Sincerely,

Dr Chris Gale
NIHR Clinical Lecturer in Paediatrics

This neonatal unit is taking part in the WHEAT trial

WHEAT is trying to find out how best to care for premature babies who need a blood transfusion.

We are comparing practices that already take place in neonatal units in the UK and are offering every baby born more than 10 weeks before their due date the opportunity to participate.

The WHEAT study is an opt-out study. This means that all babies born more than 10 weeks early will take part unless you opt out.

Please feel free to discuss this study with a doctor or nurse or call [insert local research nurse number]

This neonatal unit is taking part in the WHEAT trial

WHEAT is trying to find out how best to care for premature babies who need a blood transfusion.

We are comparing practices that already take place in neonatal units in the UK and are offering every baby born more than 10 weeks before their due date the opportunity to participate.

The WHEAT study is an opt-out study. This means that all babies born more than 10 weeks early will take part unless you opt out.

Please feel free to discuss this study with a doctor or nurse or call [insert local research nurse number]

20 November 2014

Dr Chris Gale
NIHR Clinical Lecturer
Imperial College London
Section of Academic Neonatal Medicine
Imperial College London, Chelsea and Westminster Campus
369 Fulham Road
London
SW10 9NH

Dear Dr Gale

**Study title:**	**The WHEAT trial: WithHolding Enteral feeding Around packed red cell Transfusions in preterm neonates, a multicentre, superiority, randomised registry trial**
**REC reference:**	
**Protocol number:**	
**IRAS project ID:**	

Thank you for your letter of 31 October 2014, responding to the Committee's request for further information on the above research and submitting revised documentation.

The further information was considered in correspondence by a Sub-Committee of the REC. A list of the Sub-Committee members is attached.

We plan to publish your research summary wording for the above study on the HRA website, together with your contact details. Publication will be no earlier than three months from the date of this opinion letter. Should you wish to provide a substitute contact point, require further information, or wish to make a request to postpone publication, please contact the REC Manager,

Confirmation of ethical opinion

On behalf of the Committee, I am pleased to confirm a favourable ethical opinion for the above research on the basis described in the application form, protocol and supporting documentation as revised, subject to the conditions specified below.

Conditions of the favourable opinion

The favourable opinion is subject to the following conditions being met prior to the start of the study.

You should notify the REC in writing once all conditions have been met (except for site approvals from host organisations) and provide copies of any revised documentation with updated version numbers. The REC will acknowledge receipt and provide a final list of the approved documentation for the study, which can be made available to host organisations to facilitate their permission for the study. Failure to provide the final versions to the REC may cause delay in obtaining permissions.

Management permission or approval must be obtained from each host organisation prior to the start of the study at the site concerned.

Management permission ("R&D approval") should be sought from all NHS organisations involved in the study in accordance with NHS research governance arrangements.

Guidance on applying for NHS permission for research is available in the Integrated Research Application System or at http://www.rdforum.nhs.uk.

Where a NHS organisation's role in the study is limited to identifying and referring potential participants to research sites ("participant identification centre"), guidance should be sought from the R&D office on the information it requires to give permission for this activity.

For non-NHS sites, site management permission should be obtained in accordance with the procedures of the relevant host organisation.

Sponsors are not required to notify the Committee of approvals from host organisations

Registration of Clinical Trials

All clinical trials (defined as the first four categories on the IRAS filter page) must be registered on a publically accessible database within 6 weeks of recruitment of the first participant (for medical device studies, within the timeline determined by the current registration and publication trees).

There is no requirement to separately notify the REC but you should do so at the earliest opportunity e.g. when submitting an amendment. We will audit the registration details as part of the annual progress reporting process.

To ensure transparency in research, we strongly recommend that all research is registered but for non-clinical trials this is not currently mandatory.

If a sponsor wishes to contest the need for registration they should contact Catherine Blewett (catherineblewett@nhs.net), the HRA does not, however, expect exceptions to be made. Guidance on where to register is provided within IRAS.

It is the responsibility of the sponsor to ensure that all the conditions are complied with before the start of the study or its initiation at a particular site (as applicable).

Ethical review of research sites

NHS sites

The favourable opinion applies to all NHS sites taking part in the study, subject to management permission being obtained from the NHS/HSC R&D office prior to the start of the study (see "Conditions of the favourable opinion" below).

Non-NHS sites

The Committee has not yet completed any site-specific assessment (SSA) for the non-NHS research site(s) taking part in this study. The favourable opinion does not therefore apply to any non-NHS site at present. We will write to you again as soon as an SSA application(s) has been reviewed. In the meantime no study procedures should be initiated at non-NHS sites.

Approved documents

The final list of documents reviewed and approved by the Committee is as follows:

Document	*Version*	*Date*
Copies of advertisement materials for research participants	1.0	31 October 2014
Covering letter on headed paper		05 September 2014
Evidence of Sponsor insurance or indemnity (non NHS Sponsors only) [Arthur J. Gallagher International]		21 July 2014
Letter from sponsor [Imperial College]		21 August 2014
Other [Health Technology Assessment NHS R&D HTA Programme]	March 2005	
Other [Proposed Charter for Clinical Trial DMC]	Vol 365	19 February 2005
Participant information sheet (PIS) [Parent]	1.4	31 October 2014
REC Application Form	3.5	07 September 2014
Research protocol or project proposal	1.3	11 August 2014
Response to Request for Further Information		31 October 2014
Summary CV for Chief Investigator (CI) [Christopher Gale]		

Statement of compliance

The Committee is constituted in accordance with the Governance Arrangements for Research Ethics Committees and complies fully with the Standard Operating Procedures for Research Ethics Committees in the UK.

After ethical review

Reporting requirements

The attached document *"After ethical review – guidance for researchers"* gives detailed guidance on reporting requirements for studies with a favourable opinion, including:

- Notifying substantial amendments
- Adding new sites and investigators
- Notification of serious breaches of the protocol
- Progress and safety reports
- Notifying the end of the study

The HRA website also provides guidance on these topics, which is updated in the light of changes in reporting requirements or procedures.

User Feedback

The Health Research Authority is continually striving to provide a high quality service to all applicants and sponsors. You are invited to give your view of the service you have received and the application procedure. If you wish to make your views known please use the feedback form available on the HRA website: http://www.hra.nhs.uk/about-the-hra/governance/quality-assurance/

HRA Training

We are pleased to welcome researchers and R&D staff at our training days – see details at http://www.hra.nhs.uk/hra-training/

**Please quote this number on all correspondence**

With the Committee's best wishes for the success of this project.

Yours sincerely

Signed on behalf of:

Chair

Email:

Enclosures:	*List of names and professions of members who were present at the meeting and those who submitted written comments*
	"After ethical review – guidance for researchers"
Copy to:	Imperial College London
	Chelsea and Westminster NHS Foundation Trust

Attendance at Sub-Committee of the REC

Committee Members:

Name	*Profession*	*Present*	*Notes*
	Consultant Intensivist	Yes	Chaired the meeting
	Clinical Professor of Children and Young People's Cancer Care	Yes	

Also in attendance:

Name	*Position (or reason for attending)*
	REC Manager
