# Supplementary material for: Research ethics committee decision-making in relation to an efficient neonatal trial
Source: Arch Dis Child Fetal Neonatal Ed. 2016 Sep 14;102(4):F291–8. doi: 10.1136/archdischild-2016-310935 (PMC5537508; doi:10.1136/archdischild-2016-310935)
Supplement: Supplementary data [file fetalneonatal-2016-310935supp009.pdf]

27 October 2014

Dr Chris Gale
NIHR Clinical Lecturer
Imperial College London
Section of Academic Neonatal Medicine,
Imperial College London, Chelsea and Westminster Campus,
369 Fulham Road, London
SW10 9NH

Dear Dr Gale

**Study Title:**	**The WHEAT trial: WithHolding Enteral feeding Around packed red cell Transfusions in preterm neonates, a multicentre, superiority, randomised registry trial**
**REC reference:**	
**Protocol number:**	
**IRAS project ID:**	

The Research Ethics Committee reviewed the above application at the meeting held on. Thank you for attending with Dr Hyde to discuss the application.

Provisional opinion

The Committee would be content to give a favourable ethical opinion of the research, subject to receiving a complete response to the request for further information set out below.

Authority to consider your response and to confirm the Committee's final opinion has been delegated to the Chair.

Further information or clarification required

- The Committee decided that, as there is an opportunity to do so, consent should be sought from parents. The design should be changed from opt out to opt in. Therefore, please submit a consent form for completion by parents.
- Please revise the Participant Information Sheet so that it is appropriate for the opt in design.
- Please remove the last sentence from the section headed *Are there any benefits for my baby?* in the Participant Information Sheet.
- Please add details for an independent contact for complaints to the Participant Information Sheet.

If you would find it helpful to discuss any of the matters raised above or seek further clarification from a member of the Committee, you are welcome to contact Miss Penistone.

When submitting a response to the Committee, the requested information should be electronically submitted from IRAS. A step-by-step guide on submitting your response to the REC provisional opinion is available on the HRA website using the following link: http://www.hra.nhs.uk/nhs-research-ethics-committee-rec-submitting-response-provisional-opinion/

Please submit revised documentation where appropriate underlining or otherwise highlighting the changes which have been made and giving revised version numbers and dates. You do not have to make any changes to the REC application form unless you have been specifically requested to do so by the REC.

The Committee will confirm the final ethical opinion within a maximum of 60 days from the date of initial receipt of the application, excluding the time taken by you to respond fully to the above points. A response should be submitted by no later than.

Summary of the discussion at the meeting

Social or scientific value; scientific design and conduct of the study

The Committee discussed the importance of this research.

The Committee advised that they were familiar with NEC so understood the importance of this study. The Committee understood that this would be a large study involving most neonatal units in the UK. You agreed and explained that this was a comparative effectiveness study.

The Committee understood that a survey had been carried out with neonatologists and also a meta-analysis had been conducted. You agreed.

Furthermore, the Committee understood that observational studies had shown an association between withholding feeds and a lower incidence of NEC. You explained that currently results are inconclusive. The 3 studies included in the meta-analysis only demonstrated associations by comparing the before and after. It was acknowledged that in these studies there had been changes in addition to the changes in feeds which could have had an impact. There are currently no published randomised controlled trials.

The Committee queried whether most neonatologists do not know which the best practice is. You advised that the survey suggests that there is equipoise. Currently 2/3 neonatal units do not alter feeds during blood transfusions.

The Committee asked if the incidence of NEC in this patient group was 1 in 20. You agreed.

The Committee understood that the study had been powered to detect a risk reduction from 4 percent to 2.5 percent. You advised that these figures are based on data and the meta-analysis.

The Committee asked whether the decision to stop feeds for 12 hours was based on current practice and was pragmatic. You explained that they looked at scientific evidence on gut transit time and 4 hours was sufficient time for 90 percent transit. The time taken for blood flow to return to normal after the transfusion is not known. They conducted a national survey and 4 hours was the most commonly accepted time. If feeds are withheld then the baby would receive fluid or nutrition as is clinical practice and most appropriate.

The Committee asked whether blood and dextrose would be co-infused and whether some babies would have a second cannula. You explained that the second cannula would be part of standard practice in a third of units. The study would reflect standard practice.

The Committee asked whether an interim analysis would be carried out. You advised that there would be an interim analysis halfway through recruitment. The data monitoring committee would meet throughout the trial.

Recruitment arrangements and access to health information, and fair participant selection

The Committee discussed the recruitment strategy.

The Committee had noted that 4,650 babies would be recruited to the study over 3 years in England and Wales. The Committee asked whether there would be study sites in Scotland. You explained that the data collected would have been collected routinely. There are agreements in place to extract data from the existing database in England and Wales but not yet in Scotland or Northern Ireland. Hopefully these agreements will be in place in the near future.

The Committee asked if there were no exclusion criteria. You advised that they would offer the study to all.

The Committee asked about those babies with congenital abnormalities or severe congenital heart disease. You explained that they would be included as they want to make the results as generalisable as possible.

The Committee asked whether randomisation would be stratified. You advised that they would stratify by birth weight.

Favourable risk benefit ratio; anticipated benefit/risks for research participants (present and future)

The Committee asked whether there was equipoise or whether it was a low risk study. Dr Hyde advised that there is equipoise. The risk of randomisation is low. There is an equally unknown risk for each arm of the trial which is why they need to carry out the trial.

The Committee asked if there were morbidity and mortality figures available. You advised that there are figures for the incidence of NEC and went on to explain the limitations of previously conducted studies. The result of this being that 1 of the 2 approaches may be more risky but this is currently unknown.

Care and protection of research participants; respect for potential and enrolled participants' welfare and dignity

The Committee noted the good standard of data protection.

The Committee asked about how the views of parent groups had impacted on the decision to randomise twins and triplets to the same arm of the trial. You advised that there had been parents and a representative from Bliss involved. One mother of twins had experience of a blinded RCT. She had felt that she would not be able to consent for her twins to participate in an unblinded RCT. The group TAMBA also hold a similar view and the researchers had taken their policy statement into account as they agreed that it was important to recognise these views.

Informed consent process and the adequacy and completeness of participant information

The Committee explained that they were concerned about opt out consent. They understood that transfusions were rarely in the first 24 hours so there was not a great urgency. The Committee asked what the argument for opt out consent was. You advised that there are several arguments in favour of opt out consent. Both arms of the trial are standard practice so from this point of view the study is low risk. They could also get higher recruitment rates and a

more representative sample than if it was opt in. Dr Hyde explained that opt out consent is rarely seen by ethics committees but it should be seen as a valid approach. Dr Hyde referred to the HRA guidance (Information Sheets & Consent Forms. Guidance for Researchers and Reviewers. Version 3.5 May 2009) which states that opt in consent results in a lower response rate and a biased sample. Dr Hyde advised that for a CTIMP study consent must be opt in but in this study the 2 treatments are standard practice. Effectively patients are already randomised and in the study the randomisation process would be formalised. If the recruitment timeframe was extended to power the study this would result in an increased length of time before an evidenced based, effective treatment.

The Committee had found the Participant Information Sheet to be commendable. They had thought that the statement that non-evidence based approach *may* involve more risk than being in the study was potentially coercive. You explained that this statement was based on evidence from neonatal studies, including the SUPPORT trial. It was not intended to be coercive but rather to be balanced.

The Committee advised that making this statement prior to the study was based on inductive reasoning. Dr Hyde described a previous experience with a parent who had felt that they had a right to be told up front about the potential for better care.

The Committee advised that it is not yet known for this study. Dr Hyde reiterated that the inclusion benefit has been demonstrated in a number of neonatal studies and they are keen to include this point in the Participant Information Sheet. They could reword this statement if the Committee had alternative wording to suggest. The Committee suggested that the wording is reconsidered.

The Committee asked whether parents who opt out would record their electronic signature to evidence this. You advised that no parents have opted out of their data being stored on the National Neonatal Research Database. Dr Hyde explained that if parents opted out the clinician would record this and it would then be impossible to randomise the baby into the study.

The Committee advised that there is normally a written record which allows the parent to keep a copy. Dr Hyde explained that as these parents would be opting out of the study they would not want to ask them to do anything. They wanted to make it as easy as possible to opt out. If parents do not opt out then this is recorded on the system as well as a note to advise that the study had been discussed.

The Committee advised that it would be good for parents to have evidence of their decision. You explained that this would be recorded in the notes. This would be a permanent and auditable record that parent could access.

The Committee asked whether the Participant Information Sheet had been tested. You explained that it had been written by a parent and a representative from the charity Bliss. They had had extensive involvement in drafting the document. You advised that it is a difficult concept and the intention was to keep the document brief and to prevent confusion. They could take suggestions for improvements.

The Committee advised reconsidering the use of double negatives as this made the document harder to read. You both agreed.

The Committee advised adding details for an independent contact for complaints to the Participant Information Sheet. You both agreed.

Other ethical issues were raised and resolved in preliminary discussion before your attendance at the meeting.

Documents reviewed

The documents reviewed at the meeting were:

Document	*Version*	*Date*
Covering letter on headed paper		05 September 2014
Evidence of Sponsor insurance or indemnity (non NHS Sponsors only)		05 September 2014
Letter from sponsor		05 September 2014
Participant information sheet (PIS)		02 September 2014
REC Application Form [REC_Form_10092014]		10 September 2014
Research protocol or project proposal		11 August 2014
Summary CV for Chief Investigator (CI)	1	05 September 2014

Membership of the Committee

The members of the Committee who were present at the meeting are listed on the attached sheet

Statement of compliance

The Committee is constituted in accordance with the Governance Arrangements for Research Ethics Committees and complies fully with the Standard Operating Procedures for Research Ethics Committees in the UK.

Please quote this number on all correspondence

Yours sincerely

On behalf of Chair

Email:

Enclosures: List of names and professions of members who were present at the meeting and those who submitted written comments.

Copy to: Chelsea and Westminster NHS Foundation Trust

Attendance at Committee meeting

Committee Members:

Name	*Profession*	*Present*	*Notes*
	Clinical Ethicist	Yes	
	Clinical Psychologist	No	
	Lay member	Yes	
	Pharmacist	Yes	
	Lay Plus Member	Yes	
	Lay member	Yes	
	Professor of Human Physiology	No	
	Quality Assurance Manager	No	
	Professor of Orthodontics	Yes	
	Trainee Clinical Scientist	No	
	Consultant (Anaesthesia/PICU)	Yes	
	Lay Member	Yes	

Also in attendance:

Name	*Position (or reason for attending)*
	REC Assistant
	REC Manager

Imperial College London

Section of Neonatal Medicine
Imperial College London

4th Floor, Lift Bank D
Chelsea and Westminster Hospital
369 Fulham Road
London, SW10 9NH
Tel: +44 (0)203 3153519
Fax: +44 (0)203 3157998

christopher.gale@imperial.ac.uk

6th November 2014

Dr Christopher Gale MBBS MSc PhD MRCPCH

Dear,

Study title: **The WHEAT trial: With Holding Enteral feeding Around packed red cell Transfusions in preterm neonates, a multicentre, superiority, randomised registry trial**

REC reference:
Protocol number:
IRAS project ID:

Thank you for taking the time to review the WHEAT trial. Please find responses to your requests for further information detailed below:

1. The Committee decided that, as there is an opportunity to do so, consent should be sought from parents. The design should be changed from opt out to opt in. Therefore, please submit a consent form for completion by parents. Please revise the Participant Information Sheet so that it is appropriate for the opt-in design.

We accept that studies using "opt-out" consent make rare appearances at RECs in the UK. We also realise that as yet NREAP have not provided clear guidance to RECs about how they should be handled (although it was discussed at their meeting on 17th October, 2012 - attached). This does not mean that opt-out approaches are unethical and we would like to ask the committee to reconsider this point in light of the following arguments:

1. We have chosen opt-out consent to make WHEAT as easy to understand as possible for parents. Evidence from neonatal research suggests that the use of a streamlined, opt-out consent process results in greater understanding of the research study than opt-in consent (Rogers et al, Journal of Pediatrics 1998; attached).

2. Opt-in consent is acknowledged to be associated with biased findings that are not applicable to the general population, and to lower recruitment rates. As clinicians we have an ethical imperative to reduce the uncertainty in the clinical decisions we make. In the context of this low risk comparative effectiveness trial, we believe that the opt-out approach is advantageous because it will allow us to reduce uncertainty more rapidly and effectively. This ethical imperative to reduce clinical uncertainty as quickly and as effectively as possible needs to be taken into account when the approach we have chosen is scrutinised.

3. The HRA's publication Information sheets and consent forms, guidance for researchers and reviewers (version 3.5) *discusses the validity of opt out consent. It quotes the conclusions of a randomised controlled trial of "opt-in" versus "opt-out" recruitment (Junghans et al., BMJ 2005 attached): "The opt-in approach to participant recruitment, increasingly required by ethics committees, resulted in*

lower response rates and a biased sample. We propose that the opt-out approach should be the default recruitment strategy for studies with low risk to participants." WHEAT (a comparative effectiveness trial comparing two routinely used clinical treatment pathways) is a study with low-risk to participants and therefore justifies the "opt-out" consent strategy implicitly suggested by the HRA in their published guidance.

4. There is precedent for the use of opt-out consent in neonatal comparative effectiveness research: The PREMFOOD trial (REC reference 12/LO/1391, approved by NRES Committee London, Fulham 10th December 2012; Clincaltrials.gov identifier NCT01686477) is a comparative effectiveness trial, where children are recruited and randomised within 72 hours of birth to two different feeding regimens. Parents are approached by the researcher and informed of the study. This is recorded by the researcher placing a sticker in the baby's clinical notes to say the parents have been approached and informed about the study. The parents can opt out at any time, but no signed "opt-in" consent is obtained from them. Parents have welcomed this approach to recruitment for such studies (i.e. opt-out for that which is a comparison of routine clinical care, opt-in for anything which is not).

5. Opt-out consent is acceptable in other settings for example in the USA: consent which "presents no more than minimal risk of harm to subjects and involves no procedures for which written consent is normally required outside of the research context" can be carried without the requirement to sign a consent form (Basic Health and Human Services Policy for Protection of Human Research Subject 45 CFR 46.117).

6. Despite using an opt-out model of consent, WHEAT will require physical confirmation of the parents' understanding and consent decision regarding the study. The member of the clinical research team who has explained WHEAT to the parents will provide physical confirmation in the electronic health record. (Access to the electronic health record is limited to members of the clinical team and requires a password, furthermore all data entered is both traceable and auditable; data entered cannot be permanently erased or altered). Recording consent within the electronic health record in this way will have the same standing as recording it in the paper notes.

We have produced a table balancing the positive and negative arguments for both opt-in and opt-out consent processes below:

	**Positive impacts**	**Negative impacts**
Opt-out	Less biased sample (Junghans et al., BMJ 2005)	Emotional risks for the parents of only later realising the significance of any failure to opt-out
	More generalisable results	Litigation risks for clinicians if parents deny they provided consent for their baby to be involved in the study
	Greater participation leading to a shorter trial	Possibility that babies are enrolled into a trial without parents fully understanding or agreeing to participation
	More rapid resolution of clinical uncertainty	
	Lower cost	
	Greater understanding of the research study by parents (Rogers et al., Journal of Pediatrics 1998)	
	Development of a continuous dialogue about research with parents empowered to opt-out at any time	

	Positive impacts	Negative impacts
Opt-in	Signed consent form provides documentary evidence of parental consent (but not of parental understanding or voluntariness, Euricon Study Group., Lancet 2000)	Biased sample
		Less generalisable results
		Longer trial
		Longer period of clinical uncertainty
		Greater cost
		Less understanding of study by parents
		Impression of a "time-limited" consent process forcing a decision on parents.
		Possibility that babies are enrolled into a trial without parents fully understanding or agreeing to participation (Euricon Study Group., Lancet 2000)
		Emotional risks for the parents of only later realising the significance of opting-in

We hope to reduce the negative risks set out above as follows:
1. Emotional risks for the parents of only later realising the significance of any failure to opt-out:

- *We have clarified the Parent Information Sheet to clarify the opt-out nature of the consent process. We have added the following statement in large, bold type to the Parent Information Sheet "***The WHEAT study is an opt-out study. This means that all babies will take part unless you let a member of the neonatal team know that you do not wish your baby to participate."**
- *We will provide card to parents when their baby has been randomised as suggested in point 4 below.*

2. Litigation risks for clinicians if parents deny they provided consent for their baby to be involved in the study:

- *Using an electronic health record means that documentation that the WHEAT trial and the opt-out consent process have been explained to and understood by parents are mandatory prior to randomisation. The documentation will be permanent, traceable and fully auditable.*

3. Possibility that babies are enrolled into a trial without parents fully understanding or agreeing to participation:

- *Using an electronic health record means that documentation that the WHEAT trial and the opt-out consent process have been explained to and understood by parents are mandatory prior to randomisation.*
- *This risk exists in opt-in research studies as well: in the EURICON study (Lancet 2000; attached) only 59 of 200 parents approached for informed consent using an opt-in process had given valid consent or refusal.*

2. Please remove the last sentence from the section headed "Are there any benefits for my baby?" in the Participant Information Sheet.

The evidence for inclusion benefit in neonatal clinical trials is compelling, with some of the most conclusive and recent evidence coming from a large clinical trial that enrolled only babies (Carlo et al, NEJM 2012; attached). Our statement thus represents current scientific knowledge. We feel it is important that this important information is not withheld from parents. Providing this information ensures that they are truly fully informed.

In order to make this section more balanced we have replaced the statement "This non-evidence based approach to neonatal care may involve more risk than being in a study like WHEAT which involves a carefully designed protocol and consistent monitoring" with "taking part in a research study may confer nonspecific benefits" (changes highlighted in the Participant Information Sheet).
We would encourage the committee to watch the following video clip by the renowned ethicist and Professor of Paediatric Bioethics John Lantos before they reject our appeal on this point. (https://www.youtube.com/watch?v=SmWJnOp1QaU) It explains our rationale for this statement. We do not feel parents can make an informed decision about a study without knowing both the risks and the potential benefits.

3. Please add details for an independent contact for complaints to the Participant Information Sheet.
This has been added to the PIS as follows: "You can also discuss this study with the Patient Advice and Liaison Services (PALS): [telephone number]."

I hope these responses provide sufficient clarification, please do not hesitate to contact us if you require any further information.

Documents attached:

Document	Version	Date
Participant Information Sheet	1.4	31 October 2014
Rogers et al., Pediatrics		1998
EURICON, 2000, Lancet		December 2000
Carlo et al., NEJM		2012
Junghans et al., BMJ		2005

Yours Sincerely,

Dr Chris Gale
NIHR Clinical Lecturer in Paediatrics

Health Research Authority

National Research Ethics Service

24 November 2014

Dr Chris Gale
NIHR Clinical Lecturer
Imperial College London
Section of Academic Neonatal Medicine,
Imperial College London, Chelsea and Westminster Campus,
369 Fulham Road, London
SW10 9NH

Dear Dr Gale

**Study Title:**	**The WHEAT trial: WithHolding Enteral feeding Around packed red cell Transfusions in preterm neonates, a multicentre, superiority, randomised registry trial**
**REC reference number:**	
**Protocol number:**	
**IRAS project ID**	

Thank you for your letter of 06 November 2014, responding to the Committee's request for further information on the above research, and enclosing the following revised documents:

Document	*Version*	*Date*
Other [Response letter to REC]	1	06 November 2014
Other [Patient Information Sheet]	1.4	31 October 2014
Other [Carlo 2013]	1	07 November 2012
Other [EURICON 2000]	1	07 November 2000
Other [Junghans BMJ]	1	07 November 2005
Other [Rogers 1998]	1	07 November 1998

The further information and revised documentation has been considered by the Committee. The Committee appreciated the further documentation. It was agreed that an opt-out approach is a valid approach but the Committee did not agree that it was an ethical approach to take in this study. As was previously discussed, the Committee agreed that as parents would be approached about the study and there would be an opportunity to seek consent, this should be done.

The Committee would still be grateful for a more complete response on the following points:

- The Committee decided that, as there is an opportunity to do so, consent should be sought from parents. The design should be changed from opt out to opt in. Therefore, please submit a consent form for completion by parents.
- Please revise the Participant Information Sheet so that it is appropriate for the opt in design.
- Please remove the last sentence from the section headed *Are there any benefits for my baby?* in the Participant Information Sheet.

Any further revised document submitted should be given a revised version number and date.

The 60 day clock for issue of a final ethical opinion on this application will re-start when the Committee has received a response on the outstanding points.

**Please quote this number on all correspondence**

Yours sincerely

REC Manager

Email:

Copy to: Chelsea and Westminster NHS Foundation Trust

Imperial College London

Section of Neonatal Medicine
Imperial College London

4th Floor, Lift Bank D
Chelsea and Westminster Hospital
369 Fulham Road
London, SW10 9NH
Tel: +44 (0)203 3153519
Fax: +44 (0)203 3157998

christopher.gale@imperial.ac.uk

14th January 2015

Dr Christopher Gale MBBS MSc PhD MRCPCH

Dear,

Study title: **The WHEAT trial: With Holding Enteral feeding Around packed red cell Transfusions in preterm neonates, a multicentre, superiority, randomised registry trial**

REC reference:
Protocol number:
IRAS project ID:

Thank you for taking the time to review the WHEAT trial. Please find responses to your requests for further information detailed below:

1. The Committee decided that, as there is an opportunity to do so, consent should be sought from parents. The design should be changed from opt out to opt in. Therefore, please submit a consent form for completion by parents.
2. Please revise the Participant Information Sheet so that it is appropriate for the opt-in design.
    - For the reasons outlined in our previous correspondence (improved understanding of the research study by parents, a less biased sample, more generalisable results and more rapid resolution of clinical uncertainty) we feel that an opt-out design is optimal in a low risk comparative effectiveness trial such as WHEAT. We are not willing to change the design to opt-in consent.
    - We note that in your previous correspondence "It was agreed that an opt-out approach is a valid approach but the Committee did not agree that it was an ethical approach to take in this study". Please can we have further clarification as to why this approach is not ethical in the WHEAT trial.
3. Please remove the last sentence from the section headed *Are there any benefits for my baby?* in the Participant Information Sheet.
    - We feel it is important that parents are fully informed about the potential benefits as well as risks of participating in research. Given the compelling evidence for inclusion benefit in neonatal trials we are not willing to remove this completely from the Participant Information Sheet. We would be very happy to consider alternate wording if the committee felt this appropriate.

I hope these responses provide sufficient clarification, please do not hesitate to contact us if you require any further information.

Yours Sincerely,

Dr Chris Gale, NIHR Clinical Lecturer in Paediatrics

Health Research Authority

National Research Ethics Service

21 January 2015

Dr Chris Gale
NIHR Clinical Lecturer
Imperial College London
Section of Academic Neonatal Medicine,
Imperial College London, Chelsea and Westminster Campus,
369 Fulham Road, London
SW10 9NH

Dear Dr Gale

**Study title:**	**The WHEAT trial: WithHolding Enteral feeding Around packed red cell Transfusions in preterm neonates, a multicentre, superiority, randomised registry trial**
**REC reference:**	
**Protocol number:**	
**IRAS project ID:**	

Thank you for your letter of 14 January 2015, responding to the Committee's request for further information on the above research.

The further information has been considered on behalf of the Committee by the Chair.

We plan to publish your research summary wording for the above study on the HRA website, together with your contact details. Publication will be no earlier than three months from the date of this favourable opinion letter. The expectation is that this information will be published for all studies that receive an ethical opinion but should you wish to provide a substitute contact point, wish to make a request to defer, or require further information, please contact the REC Manager. Under very limited circumstances (e.g. for student research which has received an unfavourable opinion), it may be possible to grant an exemption to the publication of the study.

Ethical opinion

The Committee is unable to give a favourable ethical opinion of the research, for the following reasons:

- The Committee maintained their opinion that consent should be sought from parents and that it should be an opt in study design rather than opt out.
- The Committee were also still of the opinion that the last sentence from the section headed *Are there any benefits for my baby?* in the Participant Information Sheet should be deleted.

I regret to inform you therefore that the application is not approved.

If you would find it helpful to discuss any of the matters raised above or seek further clarification from a member of the Committee, you are welcome to contact .

Options for further ethical review

You may submit a new application for ethical review, taking into account the Committee's concerns. You should enter details of this application on the application form and include a copy of this letter, together with a covering letter explaining what changes have been made from the previous application. We strongly recommend that you submit the new application to this REC. However, you may submit the application to a different REC if you prefer. The application should be booked through the Central Booking Service (CBS) and would be allocated for review in the normal way. You should let CBS know if you would like the application to be reviewed again by this Committee.

Alternatively, you may appeal against the decision of the Committee by seeking a second opinion on this application from another Research Ethics Committee. The appeal would be based on the application form and supporting documentation reviewed by this Committee, without amendment. If you wish to appeal, you should notify the relevant Research Ethics Service Manager (see below) in writing within 90 days of the date of this letter. If the appeal is allowed, another REC will be appointed to give a second opinion within 60 days and the second REC will be provided with a copy of the application, together with this letter and other relevant correspondence on the application. You will be notified of the arrangements for the meeting of the second REC and will be able to attend and/or make written representations if you wish to do so.

The contact point for appeals is:

HRA Improvement & Liaison Manager
National Research Ethics Service

Email:

Documents reviewed

The final list of documents reviewed by the Committee is as follows:

Document	*Version*	*Date*
Covering letter on headed paper		05 September 2014
Evidence of Sponsor insurance or indemnity (non NHS Sponsors only)		05 September 2014
Letter from sponsor		05 September 2014
Other [Response letter to REC]	1	06 November 2014
Other [Patient Information Sheet]	1.4	31 October 2014
Other [Carlo 2013]	1	07 November 2012
Other [EURICON 2000]	1	07 November 2000
Other [Junghans BMJ]	1	07 November

		2005
Other [Rogers 1998]	1	07 November 1998
Other [Response Letter]	2	14 January 2015
REC Application Form [REC_Form_10092014]		10 September 2014
Research protocol or project proposal		11 August 2014
Summary CV for Chief Investigator (CI)	1	05 September 2014

Statement of compliance

The Committee is constituted in accordance with the Governance Arrangements for Research Ethics Committees and complies fully with the Standard Operating Procedures for Research Ethics Committees in the UK.

After ethical review

You are invited to give your view of the service you have received from the National Research Ethics Service and the application procedure. If you wish to make your views known please use the feedback form available on the HRA website: http://www.hra.nhs.uk/about-the-hra/governance/quality-assurance/

We are pleased to welcome researchers and R & D staff at our NRES committee members' training days – see details at http://www.hra.nhs.uk/hra-training/

**Please quote this number on all correspondence**

Yours sincerely

On behalf of
Chair

Email:

Copy to: Chelsea and Westminster NHS Foundation Trust
