# Supplementary material for: Research ethics committee decision-making in relation to an efficient neonatal trial
Source: Arch Dis Child Fetal Neonatal Ed. 2016 Sep 14;102(4):F291–8. doi: 10.1136/archdischild-2016-310935 (PMC5537508; doi:10.1136/archdischild-2016-310935)
Supplement: Supplementary data [file fetalneonatal-2016-310935supp014.pdf]

NHS
Health Research Authority

23 October 2014

Dr Chris Gale
NIHR Clinical Lecturer
Imperial College London
Section of Academic Neonatal Medicine,
Imperial College London, Chelsea and Westminster Campus,
369 Fulham Road, London
SW10 9NH

Dear Dr Gale

**Study Title:**	**The WHEAT trial: WithHolding Enteral feeding Around packed red cell Transfusions in preterm neonates, a multicentre, superiority, randomised registry trial**
**REC reference:**	
**Protocol number:**	
**IRAS project ID:**	

The Research Ethics Committee reviewed the above application at the meeting held on.

Provisional opinion

The Committee is unable to give an ethical opinion on the basis of the information and documentation received so far. Before confirming its opinion, the Committee requests that you provide the further information set out below.

Authority to consider your response and to confirm the Committee's final opinion has been delegated to a meeting of the Subcommittee of the REC.

Further information or clarification required

1. Issues discussed in the study impact severely on premature babies. The evidence from observational studies on the transfusion protocol is mixed, consequently the Committee suggests that a definitive Randomised Controlled Trial may be needed. Please comment.

2. This could be traumatic time for some parents. Please clarify if they would be trained to handle such situations.

3. It is not clearly in A6-1 or A6-2 whether the babies will be fed human milk of formula milk. It is also not clarified if the milk will be expressed through mothers. Please clarify.

4. PIS as presented is bit complex and long, especially for parents in this situation. PIS could be made brief and be simplified with lay reader friendly language.

5. PIS should clearly say that risks for the babies will be same as any risks associated with the standard care.

6. Since there are no real benefits for babies or parents for taking part in the study, any reference to benefits of taking part should be removed from the PIS, as it could be undue persuasion.

7. The “opt out” option is mentioned in the study does not seem to be ethical. With the “opt out” there is always a subtle push towards taking part in the study. This should be changed to “opt in” as usual.

8. It is mentioned that Steering Committee will stop the trial on the advice of the Data Monitoring Committee, however the Steering Committee for the study has been only partially nominated and Monitoring Committee has not been nominated at all.

9. Funding for the study has not been secured yet. Please confirm when the funding has been secured.

If you would find it helpful to discuss any of the matters raised above or seek further clarification from a member of the Committee, you are welcome to contact REC Manager.

When submitting a response to the Committee, the requested information should be electronically submitted from IRAS. A step-by-step guide on submitting your response to the REC provisional opinion is available on the HRA website using the following link: http://www.hra.nhs.uk/nhs-research-ethics-committee-rec-submitting-response-provisional-opinion/

Please submit revised documentation where appropriate underlining or otherwise highlighting the changes which have been made and giving revised version numbers and dates. You do not have to make any changes to the REC application form unless you have been specifically requested to do so by the REC.

The Committee will confirm the final ethical opinion within a maximum of 60 days from the date of initial receipt of the application, excluding the time taken by you to respond fully to the above points. A response should be submitted by no later than 22 November 2014.

Documents reviewed

The documents reviewed at the meeting were:

Document	*Version*	*Date*
Covering letter on headed paper		05 September 2014
Evidence of Sponsor insurance or indemnity (non NHS Sponsors only)		05 September 2014
IRAS Checklist XML [Checklist_10092014]		10 September 2014
Letter from sponsor		05 September 2014
Participant information sheet (PIS)	1.3	02 September 2014
REC Application Form [REC_Form_10092014]		10 September 2014
Research protocol or project proposal	1.3	11 August 2014
Summary CV for Chief Investigator (CI)	1	05 September 2014

Membership of the Committee

The members of the Committee who were present at the meeting are listed on the attached sheet

There were no declarations of interest.

Statement of compliance

The Committee is constituted in accordance with the Governance Arrangements for Research Ethics Committees and complies fully with the Standard Operating Procedures for Research Ethics Committees in the UK.

**Please quote this number on all correspondence**

Yours sincerely

Chair

Enclosures: *List of names and professions of members who were present at the meeting and those who submitted written comments.*

Copy to:

Chelsea and Westminster NHS Foundation Trust

Attendance at Committee meeting

Committee Members:

Name	*Profession*	*Present*	*Notes*
	Consultant Psychiatrist	Yes	
	Physicist	Yes	
	Retired Solicitor	Yes	
	University Lecturer/Statistician	Yes	
	Consultant Renal Physician	No	
	GP	No	
	Visiting Professor, Pharmaceutical Medicine	Yes	
	Professor of Ethics & Care	Yes	
	Retired Assistant Chief Constable	Yes	
	Non-medical lay member	Yes	
	Training Consultant	Yes	
	Director Medical Law & Ethics	Yes	
	Consultant Psychiatrist & Honorary Senior Lecturer	No	
	Business Consultant	Yes	
	Senior Cancer Information Nurse	Yes	
	Retired Clinical Pathologist	Yes	

Also in attendance:

Name	*Position (or reason for attending)*
	REC Manager

Imperial College London

Section of Neonatal Medicine
Imperial College London

4th Floor, Lift Bank D
Chelsea and Westminster Hospital
369 Fulham Road
London, SW10 9NH
Tel: +44 (0)203 3153519
Fax: +44 (0)203 3157998

christopher.gale@imperial.ac.uk

6th November 2014

Dr Christopher Gale MBBS MSc PhD MRCPCH

Dear

Study title: **The WHEAT trial: With Holding Enteral feeding Around packed red cell Transfusions in preterm neonates, a multicentre, superiority, randomised registry trial**

REC reference:
Protocol number:
IRAS project ID:

Thank you for taking the time to review the WHEAT trial. Please find responses to your requests for further information detailed below:

1. Issues discussed in the study impact severely on premature babies. The evidence from observational studies on the transfusion protocol is mixed, consequently the Committee suggests that a definitive Randomised Controlled Trial may be needed. Please comment.

There are no randomised trials comparing the intervention to be tested in WHEAT. There have been several published observational studies that have used a historical control design. These suggest that withholding enteral feeds around transfusion is associated with a reduction in necrotising enterocolitis (please see section 6a in the protocol for additional information and an updated meta-analysis). Observational studies of this nature commonly over-estimate effect sizes, furthermore in neonatal care the incidence of necrotising enterocolitis has been decreasing over time in many units (probably due to improved infection control and greater use of maternal breast milk) so the effect seen in these studies is highly likely to be due, in part or in full, to changes in practice unrelated to withholding feeds around transfusion. A randomised controlled trial is the only method that will be able to determine whether this simple intervention leads to a reduction in necrotising enterocolitis. Consequently, we have chosen a randomised controlled trial as the design for WHEAT.

2. This could be traumatic time for some parents. Please clarify if they would be trained to handle such situations.

We are not clear who it is the committee are referring to here. If it is as to whether the parents "would be trained" the answer is no. Training does not happen currently in the NHS (and would impossible in many cases where preterm birth occurs precipitously) and will not happen as part of WHEAT. If it is as to whether staff involved in discussing and recruiting parents to the WHEAT the answer is yes. There exists a high level of expertise and training in relation to research across UK neonatal units: research is integral to neonatal care and participation in neonatal research studies is virtually universal among UK neonatal units (151 neonatal units recruited into studies associated with the NIHR Neonatal Clinical Studies Group, 2011-2014 www.odp.nihr.ac.uk). For

doctors in training research training is a core paediatric competency (RCPCH). In addition we will ensure that local research nurses and local investigators have undergone Good Clinical Practice training, this will be co-ordinated by the Clinical Trials Unit.

3. It is not clearly in A6-1 or A6-2 whether the babies will be fed human milk of formula milk. It is also not clarified if the milk will be expressed through mothers. Please clarify.

We would like to make it clear that WHEAT is a pragmatic trial examining the decision as to whether a baby should be fed or nor fed around the time of blood transfusion. In WHEAT all other clinical decisions (such as what type of milk a baby is fed) remain as standard care and are determined by the clinicians looking after the baby. We expect that wherever possible babies will be fed a diet of maternal breast milk as evidence suggests that this diet is optimal.

4. PIS as presented is bit complex and long, especially for parents in this situation. PIS could be made brief and be simplified with lay reader friendly language.

We wholeheartedly agree and commend the committee for suggesting further simplification. The WHEAT Parent Information Sheet has been designed and drafted by a parent of preterm twins (HR a member of the Trail Steering Group) and parent representatives from the national charity Bliss. They have endeavoured to keep this as concise as possible while maintaining clarity, but have found it difficult to shorten further. We have removed the following section for the sake of brevity: "What if relevant new information becomes available? If new information becomes available during the study this will be evaluated by an independent Data Monitoring Committee who will advise whether or not WHEAT should continue."

We would be grateful if the committee could advise us of other areas where the Parent Information Sheet could be shortened or simplified further.

5. PIS should clearly say that risks for the babies will be same as any risks associated with the standard care.

*We have added the following sentence to page 2, section 3 of the Parent Information Sheet: "*There are risks of born prematurely; these will be the same for babies in or out of WHEAT*" (highlighted).*

6. Since there are no real benefits for babies or parents for taking part in the study, any reference to benefits of taking part should be removed from the PIS, as it could be undue persuasion.

The evidence for inclusion benefit in neonatal clinical trials is compelling, with some of the most conclusive and recent evidence coming from a large clinical trial that enrolled only babies (Carlo et al, NEJM 2012; attached). Our statement thus represents current scientific knowledge. We feel it is important that this important information is not withheld from parents. Providing this information ensures that they are truly fully informed.

In order to make this section more balanced we have replaced the statement "This non-evidence based approach to neonatal care may involve more risk than being in a study like WHEAT which involves a carefully designed protocol and consistent monitoring" with "taking part in a research study may confer nonspecific benefits" (changes highlighted in the Participant Information Sheet).

We would encourage the committee to watch the following video clip by the renowned ethicist and Professor of Paediatric Bioethics John Lantos before they reject our appeal on this point. (https://www.youtube.com/watch?v=SmWJnOp1QaU) It explains our rationale for this statement. We do not feel parents can make an informed decision about a study without knowing both the risks and the potential benefits.

7. The "opt out" option is mentioned in the study does not seem to be ethical. With the "opt out" there is always a subtle push towards taking part in the study. This should be changed to "opt in" as usual.

We accept that studies using "opt-out" consent make rare appearances at RECs in the UK. We also realise that as yet NREAP have not provided clear guidance to RECs about how they should be handled (although it was discussed at their meeting on 17th October, 2012 - attached). This does not mean that opt-out approaches are unethical and we would like to ask the committee to reconsider this point in light of the following arguments:

1. *We have chosen opt-out consent to make WHEAT as easy to understand as possible for parents. Evidence from neonatal research suggests that the use of a streamlined, opt-out consent process results in greater understanding of the research study than opt-in consent (Rogers et al, Journal of Pediatrics 1998; attached).*
2. *Opt-in consent is acknowledged to be associated with biased findings that are not applicable to the general population, and to lower recruitment rates. As clinicians we have an ethical imperative to reduce the uncertainty in the clinical decisions we make. In the context of this low risk comparative effectiveness trial, we believe that the opt-out approach is advantageous because it will allow us to reduce uncertainty more rapidly and effectively. This ethical imperative to reduce clinical uncertainty as quickly and as effectively as possible needs to be taken into account when the approach we have chosen is scrutinised.*
3. *The HRA's publication* Information sheets and consent forms, guidance for researchers and reviewers (version 3.5) *discusses the validity of opt out consent. It quotes the conclusions of a randomised controlled trial of "opt-in" versus "opt-out" recruitment (Junghans et al., BMJ 2005 attached): "The opt-in approach to participant recruitment, increasingly required by ethics committees, resulted in lower response rates and a biased sample. We propose that the opt-out approach should be the default recruitment strategy for studies with low risk to participants." WHEAT (a comparative effectiveness trial comparing two routinely used clinical treatment pathways) is a study with low-risk to participants and therefore justifies the "opt-out" consent strategy implicitly suggested by the HRA in their published guidance.*
4. *There is precedent for the use of opt-out consent in neonatal comparative effectiveness research: The PREMFOOD trial (REC reference 12/LO/1391, approved by NRES Committee London, Fulham 10th December 2012; Clincaltrials.gov identifier NCT01686477) is a comparative effectiveness trial, where children are recruited and randomised within 72 hours of birth to two different feeding regimens. Parents are approached by the researcher and informed of the study. This is recorded by the researcher placing a sticker in the baby's clinical notes to say the parents have been approached and informed about the study. The parents can opt out at any time, but no signed "opt-in" consent is obtained from them. Parents have welcomed this approach to recruitment for such studies (i.e. opt-out for that which is a comparison of routine clinical care, opt-in for anything which is not).*
5. *Opt-out consent is acceptable in other settings for example in the USA: consent which "presents no more than minimal risk of harm to subjects and involves no procedures for which written consent is normally required outside of the research context" can be carried without the requirement to sign a consent form (Basic Health and Human Services Policy for Protection of Human Research Subject 45 CFR 46.117).*
6. *Despite using an opt-out model of consent, WHEAT will require physical confirmation of the parents' understanding and consent decision regarding the study. The member of the clinical research team who has explained WHEAT to the parents will provide physical confirmation in the electronic health record. (Access to the electronic health record is limited to members of the clinical team and requires a password, furthermore all data entered is both traceable and auditable; data entered cannot be permanently erased or altered). Recording consent within the electronic health record in this way will have the same standing as recording it in the paper notes.*

We have produced a table balancing the positive and negative arguments for both opt-in and opt-out consent processes below:

	Positive impacts	Negative impacts
Opt-out	Less biased sample (Junghans et al., BMJ 2005)	Emotional risks for the parents of only later realising the significance of any failure to opt-out
	More generalisable results	Litigation risks for clinicians if parents deny they provided consent for their baby to be involved in the study
	Greater participation leading to a shorter trial	Possibility that babies are enrolled into a trial without parents fully understanding or agreeing to participation
	More rapid resolution of clinical uncertainty	
	Lower cost	
	Greater understanding of the research study by parents (Rogers et al., Journal of Pediatrics 1998)	
	Development of a continuous dialogue about research with parents empowered to opt-out at any time	
	**Positive impacts**	**Negative impacts**
Opt-in	Signed consent form provides documentary evidence of parental consent (but not of parental understanding or voluntariness, Euricon Study Group., Lancet 2000)	Biased sample
		Less generalisable results
		Longer trial
		Longer period of clinical uncertainty
		Greater cost
		Less understanding of study by parents
		Impression of a "time-limited" consent process forcing a decision on parents.
		Possibility that babies are enrolled into a trial without parents fully understanding or agreeing to participation (Euricon Study Group., Lancet 2000)
		Emotional risks for the parents of only later realising the significance of opting-in

We hope to reduce the negative risks set out above as follows:
A. Emotional risks for the parents of only later realising the significance of any failure to opt-out:

- *We have clarified the Parent Information Sheet to clarify the opt-out nature of the consent process. We have added the following statement in large, bold type to the Parent Information Sheet "***The WHEAT study is an opt-out study. This means that all babies will take part unless you let a member of the neonatal team know that you do not wish your baby to participate."**

B. Litigation risks for clinicians if parents deny they provided consent for their baby to be involved in the study:

- *Using an electronic health record means that documentation that the WHEAT trial and the opt-out consent process have been explained to and understood by parents are mandatory prior to randomisation. The documentation will be permanent, traceable and fully auditable.*

C. Possibility that babies are enrolled into a trial without parents fully understanding or agreeing to participation:

- *Using an electronic health record means that documentation that the WHEAT trial and the opt-out consent process have been explained to and understood by parents are mandatory prior to randomisation.*
- *This risk exists in opt-in research studies as well: in the EURICON study (Lancet 2000; attached) only 59 of 200 parents approached for informed consent using an opt-in process had given valid consent or refusal.*

8. It is mentioned that Steering Committee will stop the trial on the advice of the Data Monitoring Committee, however the Steering Committee for the study has been only partially nominated and Monitoring Committee has not been nominated at all.

The data monitoring committee (DMC) will be established before recruitment starts. The proposed composition of the DMC is outlined in the protocol and follows advice from the DAMOCLES Study Group (HTA 2005). The names of the members of the DMC will be provided to the REC when finalised). In accordance with the guidance of the DAMOCLES Study Group the DMC will establish a Charter at their initial meeting that will formalise the terms of reference of the DMC. The DMC will be expected to meet at least 6 monthly with a planned interim analysis after 12 months of recruitment; this will be outlined in the DMC charter and the final decision regarding the number and timing of meetings will be at the discretion of the DMC. The point at which recruitment would be stopped will be determined by the DMC and in line with the DAMOCLES statement: "Statistical issues should be only one of several considerations that a DMC needs to take into account. Other considerations include the balance of primary risks and benefits, the internal consistency of results, the consistency with, and nature of, external evidence, and the likelihood that the results would affect clinical practice." Statistical criteria will be determined by the DMC at their initial meeting and clearly recorded in the DMC Charter (a copy of which will be provided to the REC when finalised) but these will be "regarded as guidelines for recommending stopping rather than rules" (DAMOCLES, Lancet 2005).

9. Funding for the study has not been secured yet. Please confirm when the funding has been secured.

Funding for WHEAT is currently being applied for (NIHR HTA). We will confirm to the committee when funding has been secured.

I hope these responses provide sufficient clarification, please do not hesitate to contact us if you require any further information.

Documents attached:

Document	Version	Date
Participant Information Sheet	1.4	2 November 2014
Rogers et al., Pediatrics		1998
EURICON, 2000, Lancet		December 2000
Carlo et al., NEJM		2012
Junghans et al., BMJ		2005

Yours Sincerely,

Dr Chris Gale
NIHR Clinical Lecturer in Paediatrics

25 November 2014

Dr Chris Gale
NIHR Clinical Lecturer
Imperial College London
Section of Academic Neonatal Medicine,
Imperial College London, Chelsea and Westminster Campus
369 Fulham Road
London SW10 9NH

Dear Dr Gale

Study title: **The WHEAT trial: WithHolding Enteral feeding Around packed red cell Transfusions in preterm neonates, a multicentre, superiority, randomised registry trial**
REC reference:
Protocol number:
IRAS project ID:

Thank you for your letter of 6 November 2014, responding to the Committee's request for further information on the above research and submitting revised documentation.

The further information was considered in correspondence by a Sub-Committee of the REC. A list of the Sub-Committee members is attached.

We plan to publish your research summary wording for the above study on the HRA website, together with your contact details. Publication will be no earlier than three months from the date of this opinion letter. Should you wish to provide a substitute contact point, require further information, or wish to make a request to postpone publication, please contact the REC Manager**.**

Confirmation of ethical opinion

On behalf of the Committee, I am pleased to confirm a favourable ethical opinion for the above research on the basis described in the application form, protocol and supporting documentation as revised, subject to the conditions specified below.

Conditions of the favourable opinion

The favourable opinion is subject to the following conditions being met prior to the start of the study.

Management permission or approval must be obtained from each host organisation prior to the start of the study at the site concerned.

Management permission ("R&D approval") should be sought from all NHS organisations involved in the study in accordance with NHS research governance arrangements.

Guidance on applying for NHS permission for research is available in the Integrated Research Application System or at http://www.rdforum.nhs.uk.

Where a NHS organisation's role in the study is limited to identifying and referring potential participants to research sites ("participant identification centre"), guidance should be sought from the R&D office on the information it requires to give permission for this activity.

For non-NHS sites, site management permission should be obtained in accordance with the procedures of the relevant host organisation.

Sponsors are not required to notify the Committee of approvals from host organisations

Registration of Clinical Trials

All clinical trials (defined as the first four categories on the IRAS filter page) must be registered on a publically accessible database within 6 weeks of recruitment of the first participant (for medical device studies, within the timeline determined by the current registration and publication trees).

There is no requirement to separately notify the REC but you should do so at the earliest opportunity e.g when submitting an amendment. We will audit the registration details as part of the annual progress reporting process.

To ensure transparency in research, we strongly recommend that all research is registered but for non clinical trials this is not currently mandatory.

If a sponsor wishes to contest the need for registration they should contact, the HRA does not, however, expect exceptions to be made. Guidance on where to register is provided within IRAS.

It is the responsibility of the sponsor to ensure that all the conditions are complied with before the start of the study or its initiation at a particular site (as applicable).

Ethical review of research sites

NHS sites

The favourable opinion applies to all NHS sites taking part in the study, subject to management permission being obtained from the NHS/HSC R&D office prior to the start of the study (see "Conditions of the favourable opinion" below).

Non-NHS sites

The Committee has not yet completed any site-specific assessment (SSA) for the non-NHS research site(s) taking part in this study. The favourable opinion does not therefore apply to any non-NHS site at present. We will write to you again as soon as an SSA application(s) has been reviewed. In the meantime no study procedures should be initiated at non-NHS sites.

Approved documents

The final list of documents reviewed and approved by the Committee is as follows:

Document	*Version*	*Date*
Covering letter on headed paper		05 September 2014
Evidence of Sponsor insurance or indemnity (non NHS Sponsors only)		05 September 2014
IRAS Checklist XML [Checklist_10092014]		10 September 2014
IRAS Checklist XML [Checklist_17112014]		17 November 2014
Letter from sponsor		05 September 2014
Other [Response letter to REC]	1	06 November 2014
Other [Patient Information Sheet]	1.4	02 November 2014
Other [Carlo 2013]	1	07 November 2013
Other [EURICON 2000]	1	07 November 2000
Other [Junghans BMJ]	1	07 November 2005
Other [Rogers 1998]	1	07 November 1998
Participant information sheet (PIS)		02 September 2014
REC Application Form [REC_Form_10092014]		10 September 2014
REC Application Form [REC_Form_17112014]		17 November 2014
Research protocol or project proposal		11 August 2014
Summary CV for Chief Investigator (CI)	1	05 September 2014

Statement of compliance

The Committee is constituted in accordance with the Governance Arrangements for Research Ethics Committees and complies fully with the Standard Operating Procedures for Research Ethics Committees in the UK.

After ethical review

<u>Reporting requirements</u>

The attached document *"After ethical review – guidance for researchers"* gives detailed guidance on reporting requirements for studies with a favourable opinion, including:

- Notifying substantial amendments
- Adding new sites and investigators
- Notification of serious breaches of the protocol
- Progress and safety reports
- Notifying the end of the study

The HRA website also provides guidance on these topics, which is updated in the light of changes in reporting requirements or procedures.

User Feedback

The Health Research Authority is continually striving to provide a high quality service to all applicants and sponsors. You are invited to give your view of the service you have received and the application procedure. If you wish to make your views known please use the feedback form available on the HRA website:
http://www.hra.nhs.uk/about-the-hra/governance/quality-assurance/

HRA Training

We are pleased to welcome researchers and R&D staff at our training days – see details at http://www.hra.nhs.uk/hra-training/

	**Please quote this number on all correspondence**

With the Committee's best wishes for the success of this project.

Yours sincerely

Enclosures: *List of names and professions of members who were present at the meeting and those who submitted written comments*
"After ethical review – guidance for researchers"

Copy to: *Chelsea and Westminster NHS Foundation Trust*

Attendance at Sub-Committee of the REC meeting in correspondence

Committee Members:

Name	*Profession*	*Present*	*Notes*
	Physicist	Yes	
	Retired Assistant Chief Constable	Yes	
	Consultant Psychiatrist & Honorary Senior Lecturer	Yes	

Also in attendance:

Name	*Position (or reason for attending)*
	REC Assistant
