# Supplementary material for: Research ethics committee decision-making in relation to an efficient neonatal trial
Source: Arch Dis Child Fetal Neonatal Ed. 2016 Sep 14;102(4):F291–8. doi: 10.1136/archdischild-2016-310935 (PMC5537508; doi:10.1136/archdischild-2016-310935)
Supplement: Supplementary data [file fetalneonatal-2016-310935supp015.pdf]

Imperial College
London

Research Ethics Committee decision making in relation to novel methodologies for efficient trial design

Report to the HRA

Authors: Dr Chris Gale and Dr Matthew J. Hyde,
Neonatal Medicine, Imperial College London

30th October, 2015

Overview of the project

Background

It has been estimated that up to 85% of research investment is wasted(1). Inefficient and disproportionate biomedical research regulation and management constitute major sources of research waste(2) and can lead to studies being underpowered, delayed or too costly. Additional waste occurs when regulation restricts methodologies aiming to make research more efficient, for example draft guidance issued by the Office for Human Research Protections' in relation to "Disclosing Reasonably Foreseeable Risks" in comparative effectiveness research(3, 4).

Large simple trials can reduce research waste but require effective recruitment and efficient data collection(5). Evidence based methods to increase recruitment include informing participants of the potential benefits of enrolling in a clinical trial(6), a consent process involving enrolment as the default (with a simple procedure for opting-out)(7), and a simplified participant information sheet(8, 9). The efficiency of data collection can be improved by using electronic patient records (EPR) in what is referred to as "point-of-care trials"(10). However, despite evidence for the effectiveness of these approaches(11, 12) they have not been widely applied. It is not clear whether regulatory barriers contribute to the low uptake of trial methodologies.

We aimed to determine the acceptability to UK RECs of methodologies to facilitate efficient, large, simple clinical trials.

We tested the hypothesis that such methodologies would be acceptable and that decision making between different UK RECs would be consistent. In this report we outline our findings and summarise our experiences

Methods

To examine this hypothesis an identical REC application for a large simple neonatal trial incorporating methodologies to increase recruitment and efficiency was submitted to 12 different RECs.

Participating RECs did not know the application was being submitted to multiple committees or was part of a comparative study.

The submitted application outlined the WHEAT trial: a comparative effectiveness study, using a multicentre, superiority, point-of-care trial design. The trial proposes to compare two routine practices around blood transfusion among preterm babies and aims to reduce the incidence of necrotising enterocolitis (see box 1). The WHEAT study is a real trial in development with an investigator group and the support of the neonatal clinical care community within the UK(13). The WHEAT trial has been developed with extensive parent involvement: it was designed to address the third most important research question in the field of preterm birth, as prioritised by service users and clinicians(14), it incorporated parent involvement from inception (on the trial development group) and is supported by a National Institute of Health Research (NIHR) Enabling Involvement grant.

Box 1: Lay Summary of the Study as given on the REC form.

A6-1.Summary of the study. *Please provide a brief summary of the research (maximum 300 words) using language easily understood by lay reviewers and members of the public. Where the research is reviewed by a REC within the UK Health Departments Research Ethics Service, this summary will be published on the website of the National Research Ethics Service following the ethical review.*

The purpose of WHEAT is to compare two practices that are widely used in neonatal units across the UK and around the world to see if one reduces the risk of necrotising enterocolitis (NEC) in babies born early (premature).

NEC is a serious gut disease that affects about 1 in 20 very premature babies (approximately 500 each year); about 1 in 3 of these babies will die of NEC and survivors often have longterm health and developmental problems. In 2014 prevention of NEC was ranked the third most important research priority by parents and perinatal health professionals.

Premature babies receive frequent milk feeds (every 13 hours) and they often need blood transfusions because they become anaemic (they do not have enough red blood cells). Some doctors worry that feeding babies during a blood transfusion may increase the risk of NEC. Others, however, think that it is more dangerous to stop feeds. Because of this, the way babies are cared for during blood transfusions varies across the country; some babies have milk feeds stopped before, during and after a transfusion (around 12 hours in total) while others have feeds continued.

The purpose of WHEAT is to determine which approach is best. We will do this by comparing babies who have feeds stopped with those who have feeds continued during blood transfusions. Whether feeds will be stopped or continued will be decided by randomisation. Randomisation is done by computer and ensures that each baby has an equal chance of receiving either approach. WHEAT will compare standard UK practices and involves nothing new. WHEAT will take place in neonatal units all over the UK and will involve about 4,500 babies.

Professional opinion (NHS Blood and Transplant, NIHR Children's Clinical Research Network and most UK neonatal units) supports the need for a trial like WHEAT.

Research Ethics Committee application

A NHS REC application was prepared consisting of a NHS REC form (appendix 1), a participant information sheet (appendix 2), a full study protocol, researchers CVs, a valid sponsor letter and insurance certificate (both from Imperial College London). The WHEAT trial included the following methodological approaches to facilitate efficiency:

1. A point-of-care trial design where all trial data would be extracted from an existing national neonatal EPR.

2. A streamlined parent information sheet mentioning the possibility of inclusion benefit through participation. Inclusion benefit is well described in the context of neonatal randomised controlled trials (6, 15).
3. A consent process involving enrollment as the default unless a parent opted-out of participation for their infant.

The ethical issues raised by these methodological approaches were outlined and discussed in the REC form (appendix 1).

Defence of the REC application

Six RECs were selected *a-priori* and at random for researchers to attend the meetings in person; for the other committee meetings researchers were available by phone but did not physically attend the meeting.

Written responses to comments from RECs were standardised where possible to ensure all committees received the same information upon which to base their opinion. When challenged in relation to the three methodological approaches outlined above, the researchers responded to defend the ethical validity of the proposed approach but did not agree to its removal from the application. Where committees raised challenges about other aspects of the application the researchers ceded to the RECs' suggestions.

Outcomes

The primary outcome was the number of RECs who granted a final favourable opinion following all necessary correspondence.

Secondary outcome was the decision making in relation to each of the three approaches proposed to increase trial efficiency.

Results

One researcher (MJH) attended two meetings and two researchers (MJH and CG) attended four meetings. One REC asked the researchers to attend two meetings, the first was attended by one researcher (MJH) and the second by two (MJH and CG). Researchers were available by telephone for six meetings, four RECs made use of this to call one of the researchers (MJH) during the REC meeting. Eleven RECs required written responses from the researchers.

REC decisions

One REC rejected the application outright and without correspondence. Eleven RECs corresponded with the researchers, a favourable opinion was granted by nine of these and two provided an unfavourable opinion.

The three RECs that provided an unfavourable opinion did so because they considered opt-out consent invalid. One committee accepted that while there were justifications for an opt-out approach to consent they did not apply in relation to the WHEAT study, and two other committees indicated that they considered an opt-out approach to be more universally invalid . One committee stated *"as there is an opportunity to do so,* [opt in] *consent should be sought from parents"* while another indicated that *"the 'opt-out' consent is not a concept that the Committee recognises"* deeming it to be *"recruitment without consent".*

Process metrics

Responses from researchers ranged from 1 to 13 pages (median 3) and from 164 to 5227 words (median 941). Eleven RECs required study documents to be amended, amendments ranged from 1 to 7 documents (median 2). Time from committee meeting to final decision ranged from 4 to 33 working days (median 14).

Operational consistency

Initial responses

While most committee coordinators had no problem with the lack of a consent form in our study, some coordinators did query this, despite us clearly stating on the checklist the reason for not uploading a PIS. One coordinator argued at length that it could not be a valid study without a consent form. We successfully argued that there were many study scenarios where a consent form might not be needed and requested that the committee, and not the coordinator, be allowed to make the decision about the validity of the study.

One committee was trialling a pre-REC review, which entailed the researchers being sent a list of points for them to consider and/or respond to prior to the meeting. These included minor changes to the wording of the PIS, and requests for further information. Although this might appear a burdensome approach to the review process, in reality we, as researchers, found it beneficial. It helped alert us to what might come up at committee and allowed us to provide further material for committee consideration prior to the committee meeting. We suggest that there is value in this being available for all applications to RECs in the future.

In all cases, correspondence was prompt and clear instructions were provided about time of committee meetings and also their location.

Committee meetings: logistical issues

The venues of REC we attended varied widely, including university, hospital, hotel, and hospice premises. We used public transport to reach all venues. For the most part venues were accessible by this means but a few were located a considerable distance from a reasonable public transport route (with at least an hourly service). While we realise that outside of city locations, most researchers will drive to committee meetings, we suggest that ease of accessibility via public transport should be a consideration when choosing venues for REC meetings.

Waiting facilities varied greatly. Generally locations outside hospital settings had the best waiting facilities. Meetings held in hospitals often used a couple of chairs in the corridor outside as a waiting facility. Consideration should be given to how many researchers may be waiting, at one meeting there were researchers from three studies waiting at one point: two chairs may not always be sufficient.

It should be noted that some of the committee locations we visited were not sound proof so the waiting researchers could hear the discussion taking place by the committee, raising concerns about the confidentiality of the REC process.

While waiting facilities are in one way extraneous to the process, they become more important as the time spent waiting to be interviewed increases. Waiting time at the committees (beyond the

scheduled time) was on average 26 minutes (range 0 to 70 minutes). Where waiting times were long, venues providing free Wi-Fi were much appreciated.

We recommend:

1. That committees regularly test their premises to ensure waiting researchers cannot hear the committee's discussions

2. Committees endeavour to keep to time, and timeliness should be monitored

Committee meetings: operational issues

On the whole attending the committee meetings was an enjoyable experience. The committees appeared genuinely interested in the research and provided much helpful feedback that will be built into future studies.

While the majority of the committees were very welcoming, several with a distinctively informal feel, one committee had a confrontational atmosphere in which the researchers felt very much in the dock, with the chair acting as barrister and the committee as the jury. Several comments backed up this atmosphere, in particular the remark that "you could never trust a researcher and that was why REC existed".

The main difficulty we faced as researchers was that we were unable to identify the different members of the committee or determine their expertise. While all committee chairs introduced themselves, only one of twelve committees used name cards that were both clearly visible to the researchers and contained information about the members' expertise/experience. As a result of this, we answered all questions in plain English, effectively treating all committee members as Lay members. Some committee members were unhappy with these lay answers and suggested that we were "speaking down to them" when they were asking more specialist questions. We recommend that all committees should use name cards that are:

1. Clearly visible to the researchers.

2. Contain both the committee members name but also any relevant expertise, i.e. Lay, statistician, clinician (with specialism).

Telephone meetings

Interviewing the researcher by phone was a positive experience as far as we, the researchers, were concerned. The committees that phoned went beyond the call of duty in dealing with intermittent reception, and phoning the researcher multiple times to deal with this. The wait time for phone calls was longer than for face-to-face interview (mean 36 minutes; range 15-70); this did make planning difficult and a call from the committee administrator at the agreed time to explain any delays giving an updated time would have been useful.

Similarly, where committees state that they might telephone the researcher but then either cannot, or do not need to, it would be useful for the committee inform the researcher of this. In two cases we sat by the phone for up to 2 hours waiting for calls that never came.

Committee members need to remember that if a conference phone is being used, it will pick up conversation that is unexpected. On one phone call uncomplimentary comments about the researcher were overheard ("does he think we are fools, speaking in this simple language") while the chair was discussing a point with the researcher.

We recommend:

1. Telephone meetings should be available to researchers

2. Where a telephone meeting has been agreed, researchers should be informed by telephone at the agreed time if the meeting is delayed or if the researchers are not going to be called

Timeliness of responses

Responses from the committees were all received in a timely manner. One REC unusually requested us to attend a second meeting, to answer questions arising from our response. We understand this is unusual practice, but given the complexity of the study we were proposing we welcomed the opportunity to be able to discuss the study in this manner, and for the time the committee gave to considering the study.

Committee meeting minutes

The minutes of the meetings were of high quality. In one or two instances the letters could have been improved by attention to the presentation (some contained multiple typographic errors), but this did not affect the overall service. The minutes varied widely in length (Mean: 6 pages; range: 4-9), reflecting the discussions that took place at the meetings.

The minutes are divided into ethical domains, however discussion at the committee meeting rarely follow this order; as a consequence it is sometimes difficult to recall the meeting from the minutes, as they are structured.

Electronic responses to the committee

It was not made clear, in either the general guidance on the HRA website or in the correspondence from the REC, that responses to the committee's would only be accepted when electronically uploaded via IRAS. Using this electronic response system was inefficient and cumbersome: when we wanted to respond to a committee we had to first request that the committee coordinator activate the electronic system, a process which often took more that 24 hours.

We recommend:

1. That it is made clearer to researchers that all correspondence must be submitted electronically via the IRAS system

2. The process for electronic submission of response letters via IRAS be activated immediately following the initial committee response

Consistency in decision-making:

Comparative effectiveness research:

The submitted application, WHEAT, was a comparative effectiveness study that randomised two clinical procedures currently used in neonatal intensive care units in the UK. Currently babies receive one or other treatment on the basis of either the unit where they were treated (if the unit has a standard protocol/treatment), or the clinician caring for them. Where standardised protocols are in use these are not based on high-quality evidence and in most UK units there is equipoise concerning which treatment is best. The WHEAT trial sought to replace this subjective, non-evidence based

variation in treatment with formal randomisation and measurement to determine the optimal approach. The risk of taking part in the study therefore relates to the risk associated with the randomisation process and not the treatment. We explained this in the PIS as follows:

> ***Does my baby have to take part?***
>
> *We are comparing practices that already take place in neonatal units in the UK and are offering every baby the opportunity to participate. Your baby does not have to take part if you don't want them to, in this case please tell a member of the local clinical team (names and contact details are provided at the end of this leaflet) that you would like to "opt-out" (have your baby excluded from the WHEAT study).*
>
> *If you do want your baby to take part in WHEAT, you don't need to do anything.*
>
> ***What will happen if I opt-out?***
>
> *If you "opt-out" your baby will still have feeds either stopped or continued during transfusions in the same way as in WHEAT but the decision will be made by the local clinical team and the policy of the neonatal unit, and information about your baby will not be included in the study.*
>
> ***What will happen if I do not opt-out (agree to my baby participating in WHEAT)?***
>
> *If your baby needs a blood transfusion, and is receiving milk feeds, the decision about whether to stop or continue feeds during the transfusion will be decided by a process called "randomisation". Randomisation is done by computer and means that every baby has an equal chance of either having feeds stopped or continued. If your baby is randomised to have feeds stopped this will be for 4 hours before, after, and during this and any subsequent blood transfusions. It is quite common for premature babies to have their feeds withheld for a number of reasons. When this happens babies are given nutrition into a vein by drip to ensure their blood sugar level does not drop and to reduce any feelings of hunger they might have. Babies in WHEAT who have their milk feeds stopped around a blood transfusion will be given nutrition into a vein in the same way. If your baby is randomised to have feeds continued, there will be no change in how your baby is fed.*
>
> *<u>Babies in WHEAT will not have any extra tests</u> and in all other respects will be looked after in the same way as a premature baby not taking part in the study.*
>
> ***Are there any risks for my baby?***
>
> *There are no risks for your baby from taking part in WHEAT.*
>
> ***Are there any benefits for my baby?***
>
> *Each of the two options in the WHEAT study is currently used by doctors in the UK because we do not know which one is better. For babies not taking part in WHEAT, the choice of whether or not to stop feeds is made according to the preference of the local medical team. This non-evidence based approach to neonatal care may involve more risk than being in a study like WHEAT which involves a carefully designed protocol and consistent monitoring.*

This approach was acceptable to 8 committees, who both understood the risks involved in such comparative effectiveness research and were happy with the way we had described it in the PIS. One committee asked us to clearly state that, "risks for the babies will be the same as any risks associated with standard care." Four committees however raised concerns about the "risks" associated with one

arm as opposed to the other arm of the trial (for example the additional intravenous cannula that might be used in one arm compared to the other), suggesting that they had not clearly understood that this arm (and the intravenous cannula associated with it) is already part of accepted UK practice. These committees requested the addition of statements explaining this be included in the PIS in the form: “if your baby was born in a unit where X was the normal practice and it received treatment Y, the additional risks were A; whereas if you baby was born in a unit where Y was the practice and it received X, the reduction in risk was B.”

Given there is little choice where your preterm baby is born, we consider these statements to be largely academic, at best unhelpful to the decision making process and at worse confusing and misleading to parents. In our view this somewhat heavy-handed approach appeared to stem from a misunderstanding of the comparative effectiveness design. Given that such research is increasingly encouraged in the NHS, we recommend:

1. That committees are provided with training about comparative effectiveness research and the research ethics issues associated with it

2. That formal guidance is provided to committees about describing “risks” in different arms in comparative effectiveness studies

Participant information sheets:

For WHEAT we designed a two A4 page information sheet in conjunction with parents and parent advocacy groups. The rationale for this short “streamlined” PIS was based on evidence that short information sheets result in better retention of information by participants and that comparative effectiveness research, such as WHEAT, where there was no intervention beyond randomisation, carries low risk.

While a number of committees were supportive of this approach (with one committee commenting that they would like to see it shortened further), one committee did not recognise the PIS as being valid and required that we include all sections outlined by the HRA guidance on PIS that was developed for Clinical Trials of Investigational Medicinal Products (CTIMPs). We are concerned that requiring such complete adherence undermines the involvement of patients and the public in research design.

There was considerable variation in the additions that committees required, for example details of local Patient Advice and Liaison Service (PALS). This was non-negotiable to some committees but not mentioned by others.

We recommend:

1. Guidance should be provided about the appropriateness of different PIS structures in different research settings.

2. A “minimum heading set for PIS” should be generated for low risk research to assist researchers when involving patients and the public in developing such information.

Participant involvement in research:

WHEAT benefitted from extensive parent and parent group involvement: the research question is based on a James Lind Alliance priority setting partnership, parent focus groups informed trial design, parents have been on the trial development group from its outset and wrote the initial draft of the PIS. This was generally well-received by the committees. One committee asked for proof of the

participant involvement (in the form of minutes or something similar) in relation to several aspects of the study. We feel that this is good practice and should be encouraged to ensure transparency. Unfortunately because this had not been pre-specified we had not formally recorded the development of the PIS.

We recommend:

1. That guidance should be issued to researchers about the importance of documenting patient and public involvement, with examples of different, acceptable approaches.

Opt-out consent and how consent is recorded:

The WHEAT study proposed making use of an "opt-out" approach to consent, as described in the PIS above, with this "opt-out" consent recorded by the healthcare professional in the electronic patient record; no parent/guardian signature was required. We were surprised by how rigidly some committees view the perceived requirement that the Declaration of Helsinki require for consent in writing.

While we vigorously uphold the principle of informed consent, we propose that different ways of recording consent can be equally valid. The electronic health record that WHEAT proposed to uses individual password protected access, creates a full audit trail of any changes, is secure, backed up, and cannot be mislaid as can paper consent forms. The electronic system also allowed additional safeguards to be added, for example a baby could not be randomised without the clinician recording that the study had been fully explained to the parents and they had not opted out.

Despite this, some committees considered a signed paper consent form the minimum for a randomised study to be considered "ethical": one committee stated that *"the 'opt-out' consent is not a concept that the Committee recognises"* deeming it to be *"recruitment without consent"*. This contrasts with the decision reached by nine other RECs that recognised and approved the "opt-out" consent model we proposed.

We recommend that the validity of "opt-out" models of consent is clarified, in particular in relation to:

1. What types of research it may be appropriate for

2. What, if any, safeguards that should be incorporated

Inclusion benefit:

We were particularly impressed by the way the committees engaged with us concerning our mention of the possibility of inclusion benefit for the baby in the PIS. We initially worded this as:

> *"Each of the two options in the WHEAT study is currently used by doctors in the UK because we do not know which one is better. For babies not taking part in the WHEAT study, the choice of whether or not to stop feeds is made according to the preference of the local medical team.*
>
> *This non-evidence based approach to neonatal care may involve more risk than being in a study like WHEAT which involves a carefully designed protocol and consistent monitoring."*

This statement relating to inclusion benefit was based on evidence from neonatal clinical trials, including most recently the SUPPORT trial (15).

The nine RECs that provided a favourable opinion initially raised concerns about this language. One, inexplicably, gave as a condition for approval: "Removal of information which states that there is an inclusion benefit to participants; this will not be the case as participants are babies." We know of no evidence that the Hawthorne effect does not apply to babies and had specifically referenced data from neonatal trials in the REC application to support our use of a statement about inclusion benefit in the PIS.

Through discussion with the committees the following modified statement was developed and included in the approved parent information sheet in all 9 cases:

> *"Each of the two options in the WHEAT study is currently used by doctors in the UK because we do not know which one is better. For babies not taking part in the WHEAT study, the choice of whether or not to stop feeds is made according to the preference of the local medical team.*
>
> *Taking part in a research study may confer non-specific benefits."*

We believe that patients should be informed of the potential benefits associated with research participation, in much the same way as they are with potential risks. We welcome the way committees worked with us in relation to this.

Conclusions

We believe that the review provided by UK RECs is robust and promotes high quality, ethical research. The suggestions made by the committees were overwhelmingly beneficial in relation to the design and conductance of the research.

While some variation in REC decisions is to be expected, we believe our findings are within this tolerance except in relation to "opt-out" models of consent, where the current variation between committees leads to unacceptable inconsistency in final decision-making.

We suggest that guidance relating to pragmatic research methodologies, such as those outlined here, should be provided to committees.

We commend the HRA for independently examining committee consistency in this study and recommend that work should be ongoing, and should seek to determine the effectiveness of any interventions (such as guidance directed at committees) in the same way that the effectiveness of a medical treatment is determined.

References

1. Chalmers I, Glasziou P. Avoidable waste in the production and reporting of research evidence. Lancet. 2009 Jul 4;374(9683):86-9.
2. Al-Shahi Salman R, Beller E, Kagan J, Hemminki E, Phillips RS, Savulescu J, et al. Increasing value and reducing waste in biomedical research regulation and management. Lancet. 2014 Jan 11;383(9912):176-85.
3. OHRP. Draft Guidance on Disclosing Reasonably Foreseeable Risks in Research Evaluating Standards of Care. www.hhs.gov2014 [13/5/2015]; Available from: http://www.hhs.gov/ohrp/newsroom/rfc/comstdofcare.html.
4. OHRP and standard-of-care research. N Engl J Med. 2014 Nov 27;371(22):2125-6.

5. Grossmann C, Sanders J, English RA. Large simple trials and knowledge generation in a learning healthcare system. In: Medicine Io, editor. Washington D.C.: The National Academies Press; 2013.
6. Lantos JD. The "inclusion benefit" in clinical trials. J Pediatr. 1999 Feb;134(2):130-1.
7. Berry JG, Ryan P, Duszynski KM, Braunack-Mayer AJ, Carlson J, Xafis V, et al. Parent perspectives on consent for the linkage of data to evaluate vaccine safety: a randomised trial of opt-in and opt-out consent. Clin Trials. 2013;10(3):483-94.
8. Treweek S, Altman DG, Bower P, Campbell M, Chalmers I, Cotton S, et al. Making randomised trials more efficient: report of the first meeting to discuss the Trial Forge platform. Trials. 2015 Jun 5;16(1):261.
9. Man MS, Healthlines Study G, Rick J, Bower P, Group M-S. Improving recruitment to a study of telehealth management for long-term conditions in primary care: two embedded, randomised controlled trials of optimised patient information materials. Trials. 2015;16(1):309.
10. van Staa TP, Dyson L, McCann G, Padmanabhan S, Belatri R, Goldacre B, et al. The opportunities and challenges of pragmatic point-of-care randomised trials using routinely collected electronic records: evaluations of two exemplar trials. Health Technol Assess. 2014 Jul;18(43):1-146.
11. Treweek S, Lockhart P, Pitkethly M, Cook JA, Kjeldstrom M, Johansen M, et al. Methods to improve recruitment to randomised controlled trials: Cochrane systematic review and meta-analysis. BMJ open. 2013;3(2).
12. Frobert O, Lagerqvist B, Olivecrona GK, Omerovic E, Gudnason T, Maeng M, et al. Thrombus aspiration during ST-segment elevation myocardial infarction. N Engl J Med. 2013 Oct 24;369(17):1587-97.
13. Gale C MN. Neonatal randomised point-of-care trials are feasible and acceptable in the

UK: results from two national surveys. Arch Dis Child Fetal Neonatal Ed. 2015;in press.
14. Duley L, Uhm S, Oliver S, Preterm Birth Priority Setting Partnership Steering G. Top 15 UK research priorities for preterm birth. Lancet. 2014 Jun 14;383(9934):2041-2.
15. Carlo WA, Bell EF, Walsh MC, Network SSGotEKSNNR. Oxygen-saturation targets in extremely preterm infants. N Engl J Med. 2013 May 16;368(20):1949-50.
