# Supplementary material for: Research ethics committee decision-making in relation to an efficient neonatal trial
Source: Arch Dis Child Fetal Neonatal Ed. 2016 Sep 14;102(4):F291–8. doi: 10.1136/archdischild-2016-310935 (PMC5537508; doi:10.1136/archdischild-2016-310935)
Supplement: Supplementary data [file fetalneonatal-2016-310935supp016.pdf]

RESEARCH ETHICS COMMITTEE DECISION MAKING IN RELATION TO NOVEL METHODOLOGIES FOR EFFICIENT TRIAL DESIGN

MANAGEMENT RESPONSE

1. SUMMARY & BACKGROUND

This report is the response to the recommendations made in the report entitled ‘Research Ethics Committee decision making in relation to novel methodologies for efficient trial design’, presented to the HRA by Dr Christopher Gale and Dr Matthew Hyde; and should be read in conjunction with the full report.

This exercise was undertaken at the request of the researchers, Dr Gale and Dr Hyde. The aim was to test the acceptability by UK Research Ethics Committees (RECs) (the RECs involved were all in England but some of the principles in the management response apply more broadly across the UK as part of the UK Research Ethics Service) of strategies applied to point of care clinical trials by submitting the same application to a number of different RECs as though each was an actual submission. This gave the researchers a unique position of being able to experience different approaches between different RECs and administration staff. In particular the researchers wanted to test the ethical acceptability of an opt-out consent process prior to finalising the study which would be submitted to a REC independent of this process for a formal ethical review. The accompanying report details the findings and observations made by the researchers. Within this response, we have responded to the specific recommendations made and also answer some of the broader points raised within the report.

The HRA is aware that opt-out consent is an issue which attracts strong opinions and additionally in this study there was the addition of the vulnerability of the research population i.e. neonates.

The anonymity of the study was broken by two members of the administrative staff. The staff agreed to retain this information in confidence and RECs were not notified.

2. STRATEGIC OVERVIEW

The HRA has a number of approaches to monitor and review the service which it provides to service users and stakeholders. This is to ensure the service can be kept under continual review and improvement as well as taking action to prevent the recurrence of any issue where necessary.

- **Shared Ethics Debate (ShED) -** A previously reviewed application is sent to a number of RECs as an exercise to identify differing practices, etc. This process is similar to the exercise undertaken in this instance but the internal ShEDs are known to be a formerly reviewed application. RECs receive individual reports on their review of the ShED and are asked to

consider this within the REC meeting to reflect on the outcome in comparison to other RECs. The ShED exercise has been continually reviewed and improved and further work will be undertaken to look at the use of training to assist in addressing any issues raised.

- **Quality Checks (QC)** - Each REC is assessed as a minimum on an annual basis by managers against set standards including compliance with Standard Operating Procedures, the HRA Assessment Review Portal (HARP) minimum data set and Operational Management Guidance. This includes an annual meeting observation where an operational manager attends the REC meeting to observe proceedings. Additionally, each REC undergoes a full Quality Assurance audit every three years.
- **User satisfaction reports** - Collated biannually and reviewed by the HRA Operational Management Group to identify any required action and submitted to HRA Executive Management Team.
- **Review of complaints** - Action to prevent recurrence is taken in response to all complaints relating to the REC service which are upheld.
- **Improvement audits** - The improvement team undertakes audits looking at various areas of the REC service. This is to identify any potential areas for service improvement, to establish the root cause of issues and to develop projects to look at how the service could be improved.
- **Policies and Procedures** – The documents – Guidance for Researchers attending a meeting and Guidance for the conduct of REC meetings - have been updated to include, where necessary, those recommendations contained in this report.

> Consistency of REC review – National Research Ethics Advisers' Panel (NREAP)
>
> "Whilst consistency in terms of content (i.e. REC opinions and their associated justifying reasons) is desirable, different committees may legitimately come to different decisions about

The roll out of HRA Approval has also meant that there is an additional mechanism available to maximise the quality of the service which is being provided with RECs provided with a copy of the HRA assessment to inform their ethical review.

Additionally, a piece of work was undertaken by the National Research Ethics Advisory Panel (NREAP) which looked at the issue of consistency in REC review (available at http://www.hra.nhs.uk/about-the-hra/our-committees/panels-and-advisory-groups/nreap-guidance/). A progress report on implementing the suggestions contained in the report has been provided to NREAP. This document recognises the need for consistency in terms of process and decision making but also acknowledges that, by the very nature of different committees being made up of different individuals and combinations of experience and professional expertise, there may legitimately be

some variation in the decision making. However, there is a limit to the range of decisions that are acceptable. In light of this report, further work has been undertaken to monitor consistency and to take action when there are concerns that process has not been followed or there is misunderstanding or misapplication of guidance. It is noted that Dr Gale and Dr Hyde do acknowledge in their conclusion that some differentiation between committees would be expected and in general the committees in this exercise were within an acceptable tolerance of difference with the exception of the acceptability of opt out consent.

3. OPERATIONAL CONSISTENCY

3.1. Committee meetings: logistical issues

It is noted that the report makes reference to some committee meetings not being easily accessible by public transport and suggests that this should be something given consideration when choosing locations. Accessibility of a venue is something that is taken into consideration when choosing a venue, as well as other considerations such as cost of a venue and research activity in the local area. Applicants may attend a venue which is convenient for them dependent on their method of transport, whether they choose to drive or are travelling by public transport, so having a mix of city based and non-city based venues is generally preferable. For the purposes of this exercise, it would have been necessary to attend venues which may not ordinarily have been chosen based on their location. The service has not previously received any concern about the accessibility of meeting venues.

Recommendation 1: That committees regularly test their premises to ensure waiting researchers cannot hear the committee's discussions
Response 1: A meeting observation is undertaken for each REC on a biannual basis. The person undertaking the meeting observation (usually the Regional or Deputy Regional Manager) is expected to provide a response to the following question: Was the meeting held in a room that ensures confidentiality is met? To assess this, the person undertaking the meeting observation will be expected to check whether the REC Members can be heard from the location where applicants are asked to wait. Persons undertaking the meeting observation will be asked to pay particular attention to this question and undertaking this check when next attending a REC meeting and additionally, we will ensure an early inspection is undertaken of the venues used in this exercise to ensure that confidentiality is being maintained.

Recommendation 2: Committees endeavour to keep to time, and timeliness should be monitored

Response2: Adherence to the times detailed on the REC meeting agenda are also monitored during the meeting observation. The following question must be answered: Did applicants attend the

meeting? If yes were they dealt with courtesy and respect, kept waiting an acceptable length of time and have an acceptable place to wait? Persons undertaking the meeting observation will be asked to pay particular attention to this question when next attending a REC meeting. Our guidance provided to applicants in relation to attendance at meetings explains that applicants should be kept apprised of any delays and apologies given where appropriate and this expectation is reiterated to staff. Unfortunately, some meetings do overrun where an application requires detailed and prolonged discussion. Where we are made aware of meetings regularly over-running, discussions are held with the REC Manager and Chair to try and ascertain reasons for this and put in place measures to improve the system.

The REC service is also in the process of rolling out an administrative review of REC applications which involves the REC Manager identifying any contradictory, missing or unclear information and raising with the applicant in advance of the meeting. One of the benefits which we have seen from this administrative review is that the discussion required during the REC meeting when trying to understand the information within the application has reduced. Additionally, the roll out of the HRA Approval programme, which provides assurances to the REC, may reduce the length of the discussion required for each application. This will hopefully streamline REC meetings going forward and will reduce overruns of agenda slots. The applicant for the WHEAT study noted that they found the administrative review by the REC Manger beneficial, which is positive.

2 Committee meetings: operational issues

2.1 Committee members and expertise

Recommendation 3: We recommend that all committees should use name cards that are clearly visible to the researchers.

Response 3: REC staff are aware that name plates should always be used at REC meetings and this requirement is checked as part of the meeting observation process. The report does not specify how many meetings did not have any name cards but the expectation is that all meetings do have name cards as a rule.

Recommendation 4: We recommend that all committees should use name cards that contain both the committee members name but also any relevant expertise, i.e. Lay, statistician, clinician (with specialism).

Response 4 : It has been agreed by the UK Research Ethics Development Group to include the list of committee members, including their profession or whether they are a lay member, with the validation letter. This is so that applicants are aware of who the members of the committee are in advance of the

meeting. The members should also have their name plates in front of them so that applicants attending the meeting can identify each member and additionally, any member of the REC addressing the applicant should introduce themselves, including their profession or role on the committee; this is particularly important when the applicant is attending by telephone. This was considered to be a preferable way forward to respond to the recommendation.

4.2.2 Telephone meetings

Recommendation 5: Telephone meetings should be available to researchers

Response 5: This requirement is covered in the research ethics service Standing Operating Procedures. Wherever possible and on request, arrangements can be made to attend a REC meeting via telephone. This may not always be possible, i.e. due to there being no telephone facilities in the meeting room and a poor mobile phone signal, but all REC staff do have access to mobile phones and mobile speakers which can be taken to meetings if required.

Recommendation 6: Where a telephone meeting has been agreed, researchers should be informed by telephone at the agreed time if the meeting is delayed or if the researchers are not going to be called

Response 6 : REC staff and Members have been advised that if arrangements have been made to contact an applicant via telephone, the applicant should be contacted even if the committee decides that it has no questions to ask. This should be extended to ensuring that the applicant is kept up to date in relation to changes to the original time stated. This information is also explained in our guidance for applicants attending meetings.

> SOP 2.25. *The REC should offer the Chief Investigator the alternative of being available by phone, tele-conference or video-conference at the time of the review. Wherever possible, speakerphone facilities should be arranged so that all members present in the room may question the Chief Investigator and hear the responses, and to enable the REC Manager to take full minutes. If this is not possible, the Chair or lead reviewer may hold a phone conversation with the CI and repeat their responses to the rest of the Committee.*

2.3 Electronic responses to the committee

Recommendation 7: That it is made clearer to researchers that all correspondence must be submitted electronically via the IRAS system.

Response 7: Electronic submission via IRAS was a relatively new initiative during the period of time the WHEAT application was being submitted and reviewed. However, a review has been undertaken of all correspondence sent out for this study, where there was a requirement to submit further information. It has been identified that the standard letter when issuing a provisional opinion did include clear instructions to resubmit documents electronically via IRAS but the favourable opinion

with additional conditions letter did not include this information, even when changes to the supporting documentation was required. This has now been updated.

Recommendation 8: The process for electronic submission of response letters via IRAS be activated immediately following the initial committee response.

Response 8: REC staff will usually enable resubmission once a decision has been issued which requires something to be resubmitted. The system was relatively new at the time of the exercise and there may have been occasions when this did not happen. REC Staff will be reminded via an Operational Management E-mail Alert (OMEA) that this should happen.

Wording from the on-line guidance - examples and templates

http://www.hra-decisiontools.org.uk/consent/

We have provided a framework to help you start to develop your Participant Information Sheet. We suggest that you use this framework in association with the guidance provided on this site.

The template gives you some suggested subheadings and highlights some of the issues you may need to cover.

1. Consistency in decision-making:

3. Comparative effectiveness research:

Recommendation 9: That committees are provided with training about comparative effectiveness research and the research ethics issues associated with it

Response 9 : This has been raised with the HRA Training Department to be considered as a potential training need for possible inclusion in the training programme for REC members.

Recommendation 10: That formal guidance is provided to committees about describing “risks” in different arms in comparative effectiveness studies

Response 10 : This has been forwarded to the HRA Ethics Guidance and Strategy Manager. The expectation is that this work could be linked with a current piece of work looking at point of care comparative effectiveness trials.

4 Participant information sheets:

Recommendation 11: Guidance should be provided about the appropriateness of different Participant Information Sheet (PIS) structures in different research settings.

Response 11: A recent piece of work was undertaken by the HRA in collaboration with the Medical Research Council (MRC) in relation to information sheet and consent forms to provide comprehensive guidance. This on-line guidance does make it clear that one size does not fit all when it comes to information sheets and consent forms and that they will need to be tailored appropriately depending on the type of study etc.

This topic will be covered during member training days to promote consistency of the understanding and application of the published guidance.

Recommendation 12: A "minimum heading set for PIS" should be generated for low risk research to assist researchers when involving patients and the public in developing such information.

Response 12: The purpose of the guidance is to provide examples and suggested text, however, in keeping with the premise that one size does not fit all, minimum requirements are not provided. The examples and suggested text provided within the on-line tool are intended to be a flexible framework.

5. Participant involvement in research:

Recommendation 13: That guidance should be issued to researchers about the importance of documenting patient and public involvement, with examples of different, acceptable approaches.

Response 13 : Work is currently being undertaken by the HRA Public Involvement Lead which is looking at the information which is currently being provided to RECs about patient and public involvement at the application stage, including looking for good examples of involvement. The expectation is that this work will lead to improved guidance.

Wording from the on-line guidance - Home

http://www.hra-decisiontools.org.uk/consent/

We have provided some examples and suggested text. The guidance should be considered as a framework, not a rigid template: we would encourage you to think carefully about how best to inform potential participants. One size does not fit all: you do not need to produce the same PIS and consent form to support consent for a questionnaire study as you would to recruit into a drug trial. The best way to make sure your consent documentation is fit for purpose is to test it with patient groups or other members of the public.

6. Opt-out consent and how consent is recorded:

Recommendation 14: What types of research it may be appropriate for

Response 14 : Since this exercise was undertaken in 2014, the HRA has issued proposed guidance for consultation in relation to seeking consent for simple trials and a summary report of the consultation was published on the HRA website in July 2015. This work is currently ongoing.

With regards to opt out consent as a concept more broadly, the expectation is that a piece of work will be undertaken involving relevant stakeholders to fully explore expectations and limitations around opt out consent and to ensure that there is consistency of understanding and approach across the board. Initial discussions have been held with the Chairman of NREAP who is keen to take on this project. For example, it would be important to ensure that the approach taken by the Information Commissioner's Office in relation to opt out consent for access to data is reflected in any guidance issued by the HRA.

Recommendation 15: What, if any, safeguards that should be incorporated

Response 15: This would be incorporated in the further work undertaken.
