# Supplementary material for: Research ethics committee decision-making in relation to an efficient neonatal trial
Source: Arch Dis Child Fetal Neonatal Ed. 2016 Sep 14;102(4):F291–8. doi: 10.1136/archdischild-2016-310935 (PMC5537508; doi:10.1136/archdischild-2016-310935)
Supplement: Supplementary data [file fetalneonatal-2016-310935supp005.pdf]

Health Research Authority

07 November 2014

Dr Chris Gale
NIHR Clinical Lecturer
Imperial College London
Section of Academic Neonatal Medicine,
Imperial College London, Chelsea and Westminster Campus,
369 Fulham Road, London
SW10 9NH

Dear Dr Gale

**Study title:**	**The WHEAT trial: WithHolding Enteral feeding Around packed red cell Transfusions in preterm neonates, a multicentre, superiority, randomised registry trial**
**REC reference:**	
**Protocol number:**	
**IRAS project ID:**	

The Research Ethics Committee reviewed the above application at the meeting held on 4.

We plan to publish your research summary wording for the above study on the HRA website, together with your contact details, unless you expressly withhold permission to do so. Publication will be no earlier than three months from the date of this favourable opinion letter. Should you wish to provide a substitute contact point, require further information, or wish to withhold permission to publish, please contact the REC Manager,

Ethical opinion

The members of the Committee present gave a favourable ethical opinion of the above research on the basis described in the application form, protocol and supporting documentation, subject to the conditions specified below. .

Conditions of the favourable opinion

The favourable opinion is subject to the following conditions being met prior to the start of the study.

1. The person conducting the consent discussion should provide a clear, well written documentation in patient paper notes to document the entire consent process and clarifying the option taken (opt in/ opt out) by the parents.

You should notify the REC in writing once all conditions have been met (except for site approvals from host organisations) and provide copies of any revised documentation with updated version numbers. The REC will acknowledge receipt and provide a final list of the approved documentation for the study, which can be made available to host organisations to facilitate their permission for the study. Failure to provide the final versions to the REC may cause delay in obtaining permissions.

Management permission or approval must be obtained from each host organisation prior to the start of the study at the site concerned.

Management permission ("R&D approval") should be sought from all NHS organisations involved in the study in accordance with NHS research governance arrangements.

Guidance on applying for NHS permission for research is available in the Integrated Research Application System or at http://www.rdforum.nhs.uk.

Where a NHS organisation's role in the study is limited to identifying and referring potential participants to research sites ("participant identification centre"), guidance should be sought from the R&D office on the information it requires to give permission for this activity.

For non-NHS sites, site management permission should be obtained in accordance with the procedures of the relevant host organisation.

Sponsors are not required to notify the Committee of approvals from host organisations.

Registration of Clinical Trials

All clinical trials (defined as the first four categories on question 2 of the IRAS filter page) must be registered on a publically accessible database within 6 weeks of recruitment of the first participant (for medical device studies, within the timeline determined by the current registration and publication trees).

There is no requirement to separately notify the REC but you should do so at the earliest opportunity e.g. when submitting an amendment. We will audit the registration details as part of the annual progress reporting process.

To ensure transparency in research, we strongly recommend that all research is registered but for non-clinical trials this is not currently mandatory.

If a sponsor wishes to contest the need for registration they should contact Catherine Blewett (catherineblewett@nhs.net), the HRA does not, however, expect exceptions to be made. Guidance on where to register is provided within IRAS.

It is the responsibility of the sponsor to ensure that all the conditions are complied with before the start of the study or its initiation at a particular site (as applicable).

Ethical review of research sites

NHS Sites

The favourable opinion applies to all NHS sites taking part in the study taking part in the study, subject to management permission being obtained from the NHS/HSC R&D office prior to the start of the study (see "Conditions of the favourable opinion" below).

Non NHS sites

The Committee has not yet completed any site-specific assessment(s) (SSA) for the non-NHS research site(s) taking part in this study. The favourable opinion does not therefore apply to any non-NHS site at present. I will write to you again as soon as an SSA application(s) has been reviewed. In the meantime no study procedures should be initiated at non-NHS sites.

Summary of discussion at the meeting

Recruitment arrangements and access to health information, and fair participant selection

The Chair asked Dr Hyde to explain his role in the study. Dr Hyde informed members that he was assisting the Chief Investigator Dr Gale with the study and that he was also a member on the Riverside Committee.

The Chair discussed with Dr Hyde why the researcher team had decided for an opt-out consenting process and some members wondered if written consent was a safer way of documenting consent for both parties. Dr Hyde explained that the opt-out consent process was an acceptable consenting process in this study as there was no intervention or changes to the practice already in use.

Dr Hyde added that the study is comparing two existent, different clinical practices but there was no clear evidence of which is better. They are looking to standardise already existing practice, if they were looking at novel procedures then they would proceed with an opt in consent.

Suitability of supporting information

The Chair asked whether any information would be collected for babies with regard to them being given formula or breast milk. Dr Hyde assured the Committee that daily data of meals and clinical data would be recorded, also that the outcome measures of the feeding data are dependent on the local practice of the unit; he added that this was not key to the data quality of the study.

Members of the Committee informed Dr Hyde that other research had proven that babies who were breast fed were less likely to suffer with NEC (Necrotising enterocolitis). Dr Hyde said that they would consider future national studies to

incorporate secondary outcomes measures such as these. Dr Hyde said that as each unit is different he was unsure of any other outcome measures.

<u>Care and protection of research participants; respect for potential and enrolled participants' welfare and dignity</u>

The Committee asked Dr Hyde to explain how consent would be recorded. Dr Hyde explained that the researchers would discuss the study and care of the baby with parents/ guardians of participants and then record whether the mother/carer had agreed or not (opt-in or opt-out) of the study; this information would be entered on to a database, Furthermore participants can opt out at a later stage if they wish and their previous notes removed from the database.

<u>Informed consent process and the adequacy and completeness of participant information</u>

Dr Hyde explained that the reason for the opt-out consent process was that the results with this type of trial could be skewed if opt in consent was used. In addition, opting out would allow for a greater number of participants in the study which is low risk study. Dr Hyde said that the aim is to approach participants as soon as the baby is born and that the opt-out process would carry on throughout the study.

The Chair asked if the clinicians would feel exposed using this method as there is no documentation of consent/randomisation. Dr Hyde said that they had approached leads and so far all clinicians have supported the opt-out design, he added that electronic signatures are regarded the same as written consent. Dr Hyde confirmed that the CI Dr Gale and others are content with the set up.

The Chair explained that it was about protection on both sides to have a consent form. Dr Hyde informed the committee that everything entered onto the database is audit proof and can be monitored.

Other ethical issues were raised and resolved in preliminary discussion before your attendance at the meeting.

Dr Hyde quoted SOP's 2009 and the HRA website to say that clinical criteria for opt-out studies are acceptable if treatments were already available as routine clinical practice and being used. Dr Hyde informed the Committee that minutes from NREAP meetings also state that the opt-out option is acceptable when dealing with comparison studies.

Approved documents

The documents reviewed and approved at the meeting were:

Document	*Version*	*Date*
Covering letter on headed paper		05 September 2014
Evidence of Sponsor insurance or indemnity (non NHS Sponsors only)		05 September 2014
IRAS Checklist XML [Checklist_12092014]		12 September 2014
Letter from sponsor		05 September 2014
Participant information sheet (PIS)		02 September 2014
REC Application Form [REC_Form_12092014]		12 September 2014

Research protocol or project proposal		11 August 2014
Summary CV for Chief Investigator (CI)	1	05 September 2014

Membership of the Committee

The members of the Ethics Committee who were present at the meeting are listed on the attached sheet.

After ethical review

<u>Reporting requirements</u>

The attached document "After ethical review – guidance for researchers" gives detailed guidance on reporting requirements for studies with a favourable opinion, including:

- Notifying substantial amendments
- Adding new sites and investigators
- Notification of serious breaches of the protocol
- Progress and safety reports
- Notifying the end of the study

The NRES website also provides guidance on these topics, which is updated in the light of changes in reporting requirements or procedures.

User Feedback

The Health Research Authority is continually striving to provide a high quality service to all applicants and sponsors. You are invited to give your view of the service you have received and the application procedure. If you wish to make your views known please use the feedback form available on the HRA website: http://www.hra.nhs.uk/about-the-hra/governance/quality-assurance/

HRA Training

We are pleased to welcome researchers and R&D staff at our training days – see details at http://www.hra.nhs.uk/hra-training/

Please quote this number on all correspondence

With the Committee's best wishes for the success of this project.

Yours sincerely

Chair

E-mail:

Enclosures:	*List of names and professions of members who were present at the meeting and those who submitted written comments*<br><br>*"After ethical review – guidance for researchers"* [*SL-AR2 for other studies]*
Copy to:	*Chelsea and Westminster NHS Foundation Trust*

Attendance at Committee meeting

Committee Members:

Name	*Profession*	*Present*	*Notes*
	Retired Civil Servant	Yes	
	Research Postgraduate	No	
	Pharmacologist	Yes	
	Manager	Yes	
	Pharmaceutical Consultant	Yes	
	Consultant in Paediatric Intensive Care	Yes	
	Retired	Yes	
	Specialist in Special Care Dentistry	No	
	Clinical Pharmacology Study Data Manager	Yes	
	Consultant Neonatologist	Yes	
	Clinical Lecturer	Yes	
	Consultant Paediatric Cardiologist/Intensivist	No	
	Consultant Paediatric Gastroenterologist	Yes	
	Senior Clinical Research Associate	No	
	Bioethics Researcher	Yes	

Also in attendance:

Name	*Position (or reason for attending)*
	REC Manager

Imperial College London

Section of Neonatal Medicine
Imperial College London

4th Floor, Lift Bank D
Chelsea and Westminster Hospital
369 Fulham Road
London, SW10 9NH
Tel: +44 (0)203 3153519
Fax: +44 (0)203 3157998

christopher.gale@imperial.ac.uk

12th January 2015

Dr Christopher Gale MBBS MSc PhD MRCPCH

Dear

Study title: **The WHEAT trial: With Holding Enteral feeding Around packed red cell Transfusions in preterm neonates, a multicentre, superiority, randomised registry trial**

REC reference:
Protocol number:
IRAS project ID:

Thank you for taking the time to review the WHEAT trial, please find attached the revised trial documents reflecting the conditions for the favourable opinion.

The person conducting the consent discussion should provide a clear, well written documentation in patient paper notes to document the entire consent process and clarifying the option taken (opt in/ opt out) by the parents.

1. The person conducting the consent discussion should provide a clear, well written documentation in patient paper notes to document the entire consent process and clarifying the option taken (opt in/ opt out) by the parents.
   - We have amended the protocol to reflect this requirement. We would like the committee to be aware that increasingly neonatal units (and hospitals more generally) are going “paperless” whereby patient paper notes cease to be used. In these cases documentation will be recorded within the electronic health record.

I hope these responses provide sufficient clarification, please do not hesitate to contact us if you require any further information.

Documents attached:

Document	Version	Date
Protocol	1.4	12 January 2015

Yours Sincerely,

Dr Chris Gale
NIHR Clinical Lecturer in Paediatrics

15 January 2015

Dr Chris Gale
NIHR Clinical Lecturer
Imperial College London
Section of Academic Neonatal Medicine,
Imperial College London, Chelsea and Westminster Campus,
369 Fulham Road, London
SW10 9NH

Dear Dr Gale

Study title: **The WHEAT trial: WithHolding Enteral feeding Around packed red cell Transfusions in preterm neonates, a multicentre, superiority, randomised registry trial**

REC reference:
Protocol number:
IRAS project ID:

Thank you for your letter of 12.01.2015. I can confirm the REC has received the documents listed below and that these comply with the approval conditions detailed in our letter dated 07 November 2014

Documents received

The documents received were as follows:

Document	*Version*	*Date*
Covering letter on headed paper		12 January 2015
Research protocol or project proposal		12 January 2015

Approved documents

The final list of approved documentation for the study is therefore as follows:

Document	*Version*	*Date*

Covering letter on headed paper		05 September 2014
Covering letter on headed paper		12 January 2015
Evidence of Sponsor insurance or indemnity (non NHS Sponsors only)		05 September 2014
IRAS Checklist XML [Checklist_12092014]		12 September 2014
Letter from sponsor		05 September 2014
Participant information sheet (PIS)		02 September 2014
REC Application Form [REC_Form_12092014]		12 September 2014
Research protocol or project proposal		12 January 2015
Summary CV for Chief Investigator (CI)	1	05 September 2014

You should ensure that the sponsor has a copy of the final documentation for the study. It is the sponsor's responsibility to ensure that the documentation is made available to R&D offices at all participating sites.

**Please quote this number on all correspondence**

Yours sincerely

REC Manager

Copy to: *Imperial College London and Imperial College Healthcare NHS Trust*

Chelsea and Westminster NHS Foundation Trust
