# Supplementary material for: Research ethics committee decision-making in relation to an efficient neonatal trial
Source: Arch Dis Child Fetal Neonatal Ed. 2016 Sep 14;102(4):F291–8. doi: 10.1136/archdischild-2016-310935 (PMC5537508; doi:10.1136/archdischild-2016-310935)
Supplement: Supplementary data [file fetalneonatal-2016-310935supp006.pdf]

NHS
Health Research Authority

14 October 2014

Dr Chris Gale
NIHR Clinical Lecturer
Imperial College London
Section of Academic Neonatal Medicine,
Imperial College London, Chelsea and Westminster Campus,
369 Fulham Road, London
SW10 9NH

Dear Dr Gale

**Study Title:**	**The WHEAT trial: WithHolding Enteral feeding Around packed red cell Transfusions in preterm neonates, a multicentre, superiority, randomised registry trial**
**REC reference:**	
**Protocol number:**	
**IRAS project ID:**	

The Research Ethics Committee reviewed the above application at the meeting held. Thank you for arranging for Mr Hyde to attend and discuss the application.

Provisional opinion

The Committee is unable to give an ethical opinion on the basis of the information and documentation received so far. Before confirming its opinion, the Committee requests that you provide the further information set out below.

Authority to consider your response and to confirm the Committee's final opinion has been delegated to the Chair.

Further information or clarification required

1. You are required to provide evidence of scientific review for the study.

2. You are required to confirm and explain the existing consent in place for access to the neonatal database for the purposes of this study.

3. A hard copy consent form must be created, in line with NRES guidance. The process for this must be added to the protocol. Please follow the following link for access to the

NRES guidance;
http://www.hra.nhs.uk/resources/before-you-apply/consent-and-participation/consent-and-participant-information/

4. The participant information sheet should be re-written/re-formatted in line with NRES guidance, to ensure all pertinent areas are covered. Please follow the following link for access to the NRES guidance;
http://www.hra.nhs.uk/resources/before-you-apply/consent-and-participation/consent-and-participant-information/

5. The following additional changes to the information sheet must be made:
    a) The word 'important' must be removed from the sentence in the first paragraph reading '…we want you to know about an important study'.
    b) The risks associated with additional cannulation must be added.
    c) The sentence within the 'Are there any benefits for my baby?' section stating 'This non evidence based approach to neonatal care may involve more risk than being in a study like WHEAT which involves a carefully designed protocol and consistent monitoring' must be removed. Note: Giving this kind of information to potential participants by inclusion in a general information leaflet about research is considered to be reasonable, though the particular wording should be reviewed within the institution guidelines.

6. A one page summary of the study must be created, to be presented alongside the full participant information sheet.

7. The following changes are required to the protocol:
    a) Information must be added to the protocol confirming that any units that were found to have overridden the randomisation process a significant number of times, as a result of preference towards a particular approach, would be withdrawn from the study.
    b) The point at which recruitment would be stopped, as a result of sufficient evidence being gathered in favour of one approach, must be clearly outlined.
    c) You are required to clarify the safeguards in place to ensure excessive attempts at cannulation do not occur.
    d) The consent process must be amended to reflect the inclusion of the consent form, as above.

If you would find it helpful to discuss any of the matters raised above or seek further clarification from a member of the Committee, you are welcome to contact the REC Manager,

When submitting a response to the Committee, the requested information should be electronically submitted from IRAS. A step-by-step guide on submitting your response to the REC provisional opinion is available on the HRA website using the following link: http://www.hra.nhs.uk/nhs-research-ethics-committee-rec-submitting-response-provisional-opinion/

Please submit revised documentation where appropriate underlining or otherwise highlighting the changes which have been made and giving revised version numbers and dates. You do not have to make any changes to the REC application form unless you have been specifically requested to do so by the REC.

The Committee will confirm the final ethical opinion within a maximum of 60 days from the date of initial receipt of the application, excluding the time taken by you to respond fully to the above points. A response should be submitted by no later than 13 November 2014.

Summary of the discussion at the meeting

The committee noted Mr Hyde's statement that he was not a clinician, and that clinically related queries may be more appropriately addressed in correspondence to the CI.

<u>Social or scientific value; scientific design and conduct of the study</u>

Members discussed the two approaches that would be tested in the study, and queried whether there was true scientific equipoise between the two. It was noted there appeared to be no strong evidence in favour of either approach, but units tended to create policies to ensure practice was consistent and performed to particular standards.

The Committee queried whether the data could have been collected as part of an audit looking at historical information. Following discussion it was noted the Chief Investigator had already carried out a similar review. In addition, the Cochrane Report had reviewed data and found no solid evidence in favour of either approach, though the meta-analysis favoured the 'no feeding during blood transfusion' approach..

The Committee commented this looked like an important trial, but queried whether there was really equipoise between the two approaches in the medical community. They asked whether units that already had an established approach would participate, given it would involve them changing their standard practice. They further asked whether only units who had not established a policy on which approach to take would be included. *The applicant informed the Chief Investigator had carried out an extensive survey of neonatal units, with the majority confirming they would be willing to enrol in the study. This suggested that clinicians did not feel there was a conflict. He added units that had put guidelines in place had done so predominantly to standardise practice, and not as a result of a particular evidence base. Because there was currently a lack of evidence in favour of either approach clinicians seemed willing to support the trial.*

Members asked whether it would be appropriate to indicate that those with an adopted policy in place may wish not to participate, given it will require them to carry out practices against their usual policies. They added it could be an inclusion criteria only to include units where there was genuine equipoise i.e. there was no set policy in place. *The applicant informed, as he understood it, that Chelsea and Westminster had a policy, however, they acknowledged there was equipoise of opinion between the two approaches and had indicated they were willing to override their current policy in order to participate.*

Members suggested there could be a statement in the protocol indicating if there were a substantial number of clinicians within any given unit who favoured a particular approach, with the potential that they may therefore override randomisation process in favour or their preferred approach, these units should be excluded from participation. *The applicant informed that, in the past, study teams had monitored the randomisation process closely. He added if, during monitoring, there were found to be a substantial number of 'overrides' within a unit, that unit could be withdrawn.*

The Committee noted multiple births would be randomised to the same arm. *The applicant confirmed this, stating that the possible bias had been accounted for in the statistical analysis. He added the team had taken advice from parents on this.*

Members commented that some neonatal units may have higher or lower than average mortality rates. In addition, some of these may be electing to feed during transfusions, and others not to feed. They asked whether these existing variations would be taken in to account, in order that they do not bias data. They also asked whether participants would be recruited from an equal cross section of units to account for this. They commented the babies' condition would depend on many variables, and wished to know how the team would establish whether outcomes were as a result of the approach to feeding, or existing issues such as a lower than average mortality rate. *The applicant responded this was a rare and devastating condition, and so far approximately 90% of the units approached had indicated they would like to be involved in the trial. He added the team would have full mortality data so they could compare this against existing data, in order to undertake post-hoc analysis. He believed it was likely the coverage of units in the UK would be large enough to mitigate such issues.*

The Committee asked whether it was possible there may be an 'inverse Hawthorne Effect' in units that are being asked to change their practice away from their current standard of care. In effect, this may be attempting to change the culture of the unit and the opinions of those who work within it. *The applicant stated each unit would need to make a clear and informed decision they were happy to randomise to both approaches. They would therefore need to review their practices or policies to ensure they were able to do this.* Members commented if clinicians did not believe in the alternative approach, this may bias outcomes. *The applicant stated they would not wish to include any unit that did not have a genuine position of equipoise towards the study. He added the team felt strongly about pioneering the approach to this research, and building an evidence base that was currently lacking.*

The Committee commented the applicant had presented the study's reasoning well.

Favourable risk benefit ratio; anticipated benefit/risks for research participants (present and future)

The Committee discussed the Data Monitoring Committees (DMC) role in the study, and wished to query whether a stopping point would be implemented when it became apparent one approach had better outcomes. They noted these rules would be set out by the DMC at their first meeting. Members stated participants should not continue to be recruited if data is already sufficient. Given approximately 15% of the 'highest-risk' babies might get NEC, which was a life threatening illness, members noted it was a delicate issue, and asked for reassurance that appropriate stopping guidelines were in place. *The applicant was unable to comment, but agreed to provide a full response in correspondence.*

The Committee were keen to confirm there were safeguards in place to prevent excessive cannulation attempts, where clinicians were finding it difficult to fit additional IV lines. *The applicant stated he could refer this to the Chief Investigator to provide a full response. He added he believed it was unlikely many babies would require a 2nd cannulation. He added second cannulations for feeding were likely to be carried out in some hospitals for the purposes of feeding as part of standard care*. The Committee commented the clinicians involved should be assessing the risks during the procedure, in order to make the appropriate decision. They added it would be beneficial to document a limit on the number of attempts at cannulation in the protocol, in order to mitigate the risk.

In conclusion, the Committee confirmed the ‘stopping rules’ with regard to continuation of the trial needed to be clarified in the protocol.   Clarification was also required around safeguards to prevent excessive attempts at cannulation which may harm the baby.

Informed consent process and the adequacy and completeness of participant information

The Committee commented they did not agree with the rationale given for not seeking formal consent.   They acknowledged the justification that as both approaches are currently implemented as ‘standard practice’ across the country the researchers consider there is no need to seek consent.   It was the Committee’s view that these participants would be undergoing randomisation for the purposes of a research trial, and that they may receive treatment they would not ordinarily have received in standard care.   *The applicant noted this, but stated patients would be naturally randomised in standard care, as the approach taken would depend on where they lived and which hospital they attended.*   The committee did not agree that this was random allocation.

The Committee queried how this could be justified given the Declaration of Helsinki’s statement that informed consent should be given for the purposes of research studies.   *The applicant stated parents would have the opportunity to provide ‘opt out’ consent.   He explained there would be an electronic system in place, whereby when the baby was admitted the clinician would be alerted that they were eligible for the study.   The clinician would then speak with the parents about the study.   There would be a ‘yes’ and ‘no’ option on the screen, and if parents did not wish to participate the clinician would be able to select the appropriate option.   If they were happy to proceed their child would be randomised into the trial.   The applicant added the parents could opt out at any point, with the clinician having the ability to revisit the system and withdraw their consent.   They would retain the option to opt in or out throughout.*

The Committee noted this was a binary system, and it should not be about just saying yes or no, but rather they needed to be sure participants were making a fully informed decision about what exactly they were consenting to.   This could include access to data and randomisation into the trial, amongst other elements.   The Committee felt it was a matter of ethics, and law in the case of access to data, that participants should have a clear list of what they were consenting to so that they could give fully informed and explicit consent to all aspects of the study.   *The applicant stated that informed consent was already in place to extract data from the established neonatal database.*   Members commented specific consent to extract data for the purposes of this study would be needed.   *The applicant informed consent for this had already been taking using the ‘opt out’ consent as part of standard practice; this covered consent to access data for research purposes.   These participants had already been given the opportunity to opt out, and so the process of opt out consent had already been practiced.   To date no one had ever opted out.*

The Committee commented an opt out process would be an unusual approach, and they did not feel the arguments put forward justified a departure from usual practice.   *The applicant stated the standard consent process could be a barrier to the research.   He added if the research involved a new intervention he would agree that ‘opt out’ consent was not appropriate, however, this was a treatment being carried out in standard practice, with the approach taken currently being decided purely on the basis of which hospital you a patient attended.   The team wished to formalise this randomisation in order to build an evidence base and establish the best*

approach. The applicant stated as both approaches were already being used in hospitals the study was low risk, and a formal consent process in this case would add unnecessary burden to the parents. He confirmed participants would be approached at various points throughout the study to ask if they are still happy to be involved, and would have the participant information sheet in order to inform their initial decision.

Members asked whether the participant would have a clear list of what they were consenting to. The applicant stated they would not, however, the team could potentially produce something electronic. *The Committee asked whether a hard copy could be provided, so that participants could physically sign each consent point to indicate they agree to each element. The applicant stated this could be possible, but he would need to discuss it with the Chief Investigator as there was an effort being made to move away from paper based activity. He added electronic documentation was more secure and could be linked directly to data and medical records.* The Committee commented a report from Snowden Research indicated parents would prefer hard copies. *The applicant stated parents would have a hard copy of the information sheet, and the team had also discussed creating a document confirming they have opted out, or that they have agreed to participate.* Again, members commented they would prefer participants to have an explicit list of what they were consenting so that they could proactively sign off against each element. This would be much clearer for the participant and ensure they are fully informed. *The applicant commented surely this would be implicit within the information sheet.* The Committee stated they would take a view on whether the information sheet was adequate for this purpose.

With regard to the participant information sheet, the Committee stated they were unhappy with the statement that participating in research improved the care of patients. *The applicant outlined evidence from other trials indicating participants on randomised controlled trials did better, even if they were part of the control group. He stated it was important to tell participants the positives as well as the negatives. Given there was no evidence that either treatment approach worked better than the other, the applicant felt that participating was surely more beneficial than not participating.* The Committee were concerned it may unduly induce parents to participate. *The applicant stated if only the risks are outlined it would not be a balanced view, and would therefore not enable participants to make an informed decision. He added other trials in neonatal care had clearly shown the Hawthorne Effect had occurred, and it was important that this needed to be acknowledged by the research community.* The Committee stated they understood the applicant's view point, and would discuss the issue in private.

The Committee stated the sentence 'we want you to know about an important study' was not appropriate. *The applicant agreed, and was happy to remove the word 'important'.* In addition, there are no risks listed as a result of participation. Members felt the risks associated with a possible extra cannulation should be added.

In discussion following the applicant's departure, the Committee noted the statement around participating in research improving the care of patients was not featured in the information sheet. There was, however, a sentence in the 'Are there any benefits for my baby?' section stating 'This non evidence based approach to neonatal care may involve more risk than being in a study like WHEAT which involves a carefully designed protocol and consistent monitoring'. The Committee agreed this statement may be coercive and was not accurate. They agreed it should be removed. [Chair's note: The committee was concerned about the use of this information as an inducement to give consent to this particular study, rather than as an encouragement to participate in medical research in the form of a reassurance that to do so is generally associated with improved outcomes even in control groups. The committee would have no objection to issuing this to potential participants as an inclusion in a general information

document outlining the benefits of medical research to the community and to potential participants as is already available and commonly in use.

In terms of the consent process, members commented the study involved a point of randomisation, purely for the purposes of the study, and access to personal data. Members felt strongly that participants required the opportunity to knowingly agree to randomisation. This randomisation may result in babies within the same neonatal unit undergoing different care. Members felt this may cause concern for parents, and could be exacerbated by having no clear record to reflect on in terms of what they had consented to. The Committee did not agree that formal consent may decrease the number of parents who agree to participate. They commented participants should be making an active step to consent and 'opt in' to the study. The only justifications to an exemption of this process, in the Committee's view, would be in an emergency care situation or in other special circumstances e.g. in epidemiological studies, and/or with section 251 approval. The Committee agreed a formal, hard copy, consent form should be provided to participants, containing a full list of the activities to which participants were consenting. Participants should be required to initial against each point, clearly indicating that they understood and consented. The consent form should be created in line with NRES guidance.

Members also wished to confirm that all parents enrolled would have already given consent for their children's' data to be extracted from the neonatal database, for the purposes of this research. [Chair's note: it may be worth considering the possibility (even though this has apparently never happened) that such general consent may have been declined, and if so, would it be reasonable to be excluded from this study as a result or would a study-specific arrangement be in place?]

Members commented the participant information sheet should be reviewed and re-written in line with NRES guidance, to ensure all pertinent information was included. Given the sensitive nature of the research, it was agreed a one page summary of the study should also be created. This would enable parents to digest the key points of the study during what may be a time of distress. The fuller information sheet would then also be provided to explain the important back ground information. This could be read at the participant's leisure.

<u>Suitability of the applicant and supporting staff</u>

The Committee asked for confirmation that the study would be conducted to Good Clinical Practice (GCP) standards. *The applicant confirmed the Chief Investigator was fully GCP trained*. The Committee noted it was the sponsor's responsibility to ensure GCP training was in place.

<u>Independent review</u>

The Committee wished to receive evidence of the scientific reviews that had been carried out for the study, as indicated at A54 within the application form.

Documents reviewed

The documents reviewed at the meeting were:

Document	*Version*	*Date*
Covering letter on headed paper		05 September 2014
Evidence of Sponsor insurance or indemnity (non NHS Sponsors only)		21 July 2014
IRAS Checklist XML [Checklist_10092014]		10 September 2014
Letter from sponsor		21 August 2014
Participant information sheet (PIS) [WHEAT Parent Information Sheet]	1.3	31 August 2014
REC Application Form [REC_Form_10092014]		08 September 2014
Research protocol or project proposal	1.3	11 August 2014
Summary CV for Chief Investigator (CI)	1	05 September 2014

Membership of the Committee

The members of the Committee who were present at the meeting are listed on the attached sheet

One member declared a potential conflict of interest, however, the Committee agreed there was no conflict and the member could play a full part in discussion.

Statement of compliance

The Committee is constituted in accordance with the Governance Arrangements for Research Ethics Committees and complies fully with the Standard Operating Procedures for Research Ethics Committees in the UK.

Please quote this number on all correspondence

Yours sincerely

Chair

Email:

Enclosures: *List of names and professions of members who were present at the meeting and those who submitted written comments.*
Copy to: *Chelsea and Westminster NHS Foundation Trust*

Attendance at Committee meeting

Committee Members:

Name	*Profession*	*Present*
	Pharmacist	Yes
	Retired Consultant Head of Medical Physics	Yes
	Consultant Neonatologist	Yes
	Project Commercial Assistant	No
	Scientific Consultant	Yes
	Retired Company Director	Yes
	Retired Solicitor	Yes
	Consultant Oncologist	Yes
	Retired Patent Agent	Yes
	PPI Representative	No
	PhD Student in Genetics of Heart Disease	Yes
	Senior Nurse	Yes
	Media Consultant & Retired Principal Lecturer	Yes

Also in attendance:

Name	*Position (or reason for attending)*
	Deputy Regional Manager
	REC Manager

Imperial College London

Section of Neonatal Medicine
Imperial College London

4th Floor, Lift Bank D
Chelsea and Westminster Hospital
369 Fulham Road
London, SW10 9NH
Tel: +44 (0)203 3153519
Fax: +44 (0)203 3157998

christopher.gale@imperial.ac.uk

6th November 2014

Dear

Study title: **The WHEAT trial: With Holding Enteral feeding Around packed red cell Transfusions in preterm neonates, a multicentre, superiority, randomised registry trial**

REC reference:
Protocol number:
IRAS project ID:

Thank you for taking the time to review the WHEAT trial. Please find our numbered responses to each point made by the Research Ethics Committee (REC) detailed below. Changes to the Parent Information Sheet (PIS) are highlighted in the enclosed copy.

1 You are required to provide evidence of scientific review for the study

The study has received the following scientific review.

1.1 We conducted a large-scale questionnaire exercise directed at every neonatal unit in England. Responses were received from 111 of 163 neonatal units; these responses informed study design (for example in determining the duration of time that feeds should be withheld following blood transfusion in WHEAT).

1.2 After reviewing the proposed WHEAT protocol 87% of responding neonatal unit leads (97/111) stated that they would be willing to take part. This process and the results of this national consultation have been submitted for publication to the peer-reviewed journal Archives of Disease in Childhood. A confidential copy of the submitted journal article could be made available to the REC if required.

1.3 The WHEAT research team, listed in the application in section A63, is multi-disciplinary. This includes clinical, non-clinical, and basic science researchers, statisticians and data analysts, and patient charities and parents.

1.4 WHEAT has been favourably reviewed by the London NIHR Research Design Service (please see attached document).

1.5 WHEAT has been reviewed by the Neonatal Nutrition Network, N3 http://www.nicunutrition.com/. Please contact Dr N Embleton, Neonatal Nutrition Network Chair (nicholas.embleton@newcastle.ac.uk) if more information is required.

1.6 WHEAT has been presented by Dr Gale to Perinatal Medicine 2014, a conference of international perinatologists, hosted earlier this year in Harrogate.

2 *You are required to confirm consent in place for access to the neonatal database for the purposes of this study.*

The National Neonatal Research Database (NNRD) is a REC approved research database (10/H0803/151); this application is being made as explicit additional approval is required for all research that is not a component of the original application that established the NNRD.

3 *A hard copy consent form must be created, in line with NRES guidance. The process for this must be added to the protocol.*

We request the REC to reconsider this requirement. We are unable to accept this for the reasons listed below.

3.1 "Opt-out" studies are not inherently unethical, a point discussed by the National Research Ethics Advisory Panel (NREAP) on 17th October 2012 (attached).

3.2 The HRA discuss the validity of opt-out consent in their publication "*Information sheets and consent forms, guidance for researchers and reviewers (version 3.5)"*. They quote the conclusions of a randomised controlled trial of "opt-in" versus "opt-out" recruitment (Junghans et al, BMJ 2005; attached), namely "*The opt-in approach to participant recruitment, increasingly required by ethics committees, resulted in lower response rates and a biased sample. We propose that the opt-out approach should be the default recruitment strategy for studies with low risk to participants."* Speedy resolution of the clinical uncertainty addressed by WHEAT is clearly desirable, and as it is a low-risk comparative effectiveness trial comparing two routinely used clinical treatment pathways the "opt-out" consent strategy is justified.

3.3 The opt-out consent process also reflects our wish to reassure parents that WHEAT is a comparison of treatments already in accepted use (one of which their child will receive, even if they opt-out) rather than an evaluation of a new or experimental therapy. Please note too that the use of a streamlined, opt-out consent process results in greater understanding of the research study than opt-in consent (Rogers et al, Journal of Pediatrics 1998; attached).

3.4 There is precedent for the use of opt-out consent in neonatal comparative effectiveness research in the UK. The PREMFOOD trial (REC reference 12/LO/1391, approved by NRES Committee London, Fulham 10th December 2012; Clincaltrials.gov identifier NCT01686477) is a comparative effectiveness trial, where children are recruited and randomised within 72 hours of birth to two different feeding regimens. Parents are informed of the study and this is recorded by placing a sticker in the baby's clinical notes. It is made clear that parents can opt out at any time, but no signed "opt-in" consent is obtained.

3.5 At the REC meeting the Declaration of Helsinki was cited as only allowing opt-in consent. While we uphold the Declaration, we dispute this interpretation. The Declaration is an evolving statement that has had several amendments, the most recent of which was in 2013. Our proposals are wholly in accord with cardinal ethical principles; we will be fully informing patients (parents), and they have opportunity to opt-out of participation freely and at any time. We would also draw to your attention that opt-out consent processes are a component of current proposals by the NIHR.

3.6 There is other precedent for opt-out consent; for example in the USA consent which "presents no more than minimal risk of harm to subjects and involves no procedures for which written consent is normally required outside of the research context" can be carried without the requirement to sign a consent form (Basic Health and Human Services Policy for Protection of Human Research Subject 45 CFR 46.117).

3.7 Please also note that WHEAT **will** require documented confirmation that parents have received information about the study and documented confirmation of the decision they

reach. The member of the clinical team who explains WHEAT to parents will confirm this in the baby's electronic health record. Access to the electronic health record is limited to members of the clinical team and requires a password; all data entered are traceable and auditable. Recording the parent decision within the electronic health record in this way serves the same purpose as recording on paper.

3.8 We would ask the committee to consider the following statement with which we concur, *"It follows that consent is not always improved by trying to ensure that it is given to more, or more specific, propositions; more specific consent is not invariably better consent. Complex forms that request consent to numerous, highly specific propositions may be reassuring for administrators (they protect against litigation), and may have their place in recruiting research subjects, yet they will backfire if patients or practitioners come to see requesting and giving consent as a matter of ticking boxes. Our aim should, I suggest, be to achieve* genuine *consent, and this may not always be best done by seeking specific consent to a great many propositions"* (O'Neill, J Med Ethics, 2003).

3.9 The opt-out design and the absence of a consent form with a series of tick boxes are the result of the extensive parent involvement in WHEAT.

4 The participant information sheet should be re-written/re-formatted in line with NRES guidance, to ensure all pertinent areas are covered.

We request the REC to reconsider this requirement; we are unable to accept this for the reasons listed below.

4.1 We draw your attention to the minutes of the UK National Research Ethics Advisors' Panel (NREAP), 17th October 2012 (attached). The panel stated "*there should be greater insistence on researchers showing how they have engaged with the relevant patient population to design and validate their information sheets*" and that it is "*important to distract RECs from a pre-conceived idea of what an information sheet should look like i.e. to move them away from an 'English teacher' approach to one where they focus on the ethical challenges of presenting the required information in order to gain meaningful consent*". The HRA specifically advise REC "*to give appropriate weight to the views of patient groups or potential participants that have been consulted"* in their guidance on PIS and consent forms. WHEAT has benefited from extensive parent and parent group involvement in relation to the design of the Patient Information Sheet.

4.2 NRES guidance states clearly that the template is "*Not offered as a rigid template, but rather a flexible framework*" and the headings they provide are *"suggested.*" We have carefully considered the template and cannot find any point that we do not feel is already covered in our PIS. If the REC would indicate specific points that we have omitted we would be happy to consider these.

5 The following additional changes to the information sheet must be made:

a) The word 'important' must be removed from the sentence in the first paragraph reading '...we want you to know about an important study'.

We have removed the word "important".

b) ***The risks associated with additional cannulation must be added.***

As both treatment arms, including cannulation if an intravenous line is not already in place, constitute routine clinical care (Parige et al, ADC FN 2013; attached) we do not believe this request is justified. Our reasoning has been discussed with and shaped by the parent member of the trial development group (a parent of extremely preterm twins) and representatives of the national charity Bliss.

c) The sentence within the 'Are there any benefits for my baby?' section stating 'This non evidence based approach to neonatal care may involve more risk than being in a study like WHEAT which involves a carefully designed protocol and consistent monitoring' must be removed. Note: Giving this kind of information to potential participants by inclusion in a general information leaflet about research is considered to be reasonable, though the particular wording should be reviewed within the institution guidelines.

We have replaced the statement "*This non-evidence based approach to neonatal care may involve more risk than being in a study like WHEAT which involves a carefully designed protocol and consistent monitoring*" with "*Taking part in a research study may confer non-specific benefits*" (changes highlighted in the PIS). As we consider it an obligation to fully inform parents we do not feel the request to remove information about inclusion benefit is justified. The evidence for inclusion benefit in neonatal clinical trials is strong (e.g Carlo et al, NEJM 2012; attached). We do not feel parents can make an informed decision without being informed about both potential risks and potential benefits in relation to the specific study and that it is insufficient to rely on them having read and comprehended a statement on a generic information sheet.

We draw to your attention the following video clip by the well-known ethicist John Lantos, Professor of Paediatric Bioethics (https://www.youtube.com/watch?v=SmWJnOp1QaU). We believe the Committee may find this of interest.

6 *A one page summary of the study must be created, to be presented alongside the full participant information sheet.*

We question the need for an additional summary. This is a randomized evaluation of treatments in established use neither of which would involve the provision of written information in day-to-day clinical care. We therefore consider a short PIS is appropriate. Adding an additional information sheet would be contrary to this aim, and to the advice received from our PPI advisors.

7 The following changes are required to the protocol:

a) *Information must be added to the protocol confirming that any units that were found to have overridden the randomisation process a significant number of times, as a result of preference towards a particular approach, would be withdrawn from the study.*

We have added the following statement to the protocol on page 30: "A *site inspection will be triggered if sites are deemed to have deviated significantly from the randomly assigned treatment allocations. Further deviation will be result in withdrawal of the site from trial participation"*.

b) The point at which recruitment would be stopped, as a result of sufficient evidence being gathered in favour of one approach, must be clearly outlined.

As outlined in in the protocol and in the REC form, the data monitoring committee (DMC) will be established before recruitment starts. In accordance with the guidance of the DAMOCLES Study Group (Lancet 2005) the DMC will establish a Charter at their initial meeting that will formalise their terms of reference. The DMC will meet at least 6 monthly; there will be a planned interim analysis after 12 months of recruitment. The point at which recruitment would be stopped will be determined by the DMC in line with the DAMOCLES statement "*Statistical issues should be only one of several considerations that a DMC needs to take into account. Other considerations include the balance of primary risks and benefits, the internal consistency of results, the consistency with, and nature of, external evidence, and the likelihood that the results would affect clinical practice.*" Statistical criteria will be determined by the DMC at their initial meeting and recorded in the DMC Charter.

c) You are required to clarify the safeguards in place to ensure excessive attempts

at cannulation do not occur.

WHEAT is a comparative effectiveness trial. All UK neonatal units assess the risks and benefits associated with any procedure or practice, including cannulation, in the course of day-to-day practice and will continue to do so in relation to WHEAT. Neonatal units have established procedures in place to ensure that excessive attempts at cannulation do not occur (for example limiting the number of cannulation attempts that junior clinical staff are permitted before a more senior member takes over) and these apply to WHEAT. We have added a statement to the protocol (page 9) to ensure this is clear "*The clinical team should follow local procedures and practices to avoid excessive attempts at cannulation*".

d) The consent process must be amended to reflect the inclusion of the consent form, as above.

Please see our response above to point 3.

The following documents are attached:

Document	Version	Date
NIHR London RDS review	EIF AppID: 123	
Minutes of National Research Ethics Advisory Panel (NREAP)		17 October 2012
Junghans et al., BMJ		2005
Rogers et al., Journal of Pediatrics		1998
O'Neill, J Med Ethics		2003
Parige et al., ADC FN		2013
Carlo et al., NEJM		2012
DAMOCLES, Lancet		2005
Participant Information Sheet	1.4	13 October 2014
Protocol		

I hope this provides adequate clarification. I look forward to hearing your further response.

Yours sincerely,

Dr Christopher Gale MBBS MSc PhD MRCPCH
NIHR Clinical Lecturer in Paediatrics

Health Research Authority

21 November 2014

Dr Chris Gale
NIHR Clinical Lecturer
Imperial College London
Section of Academic Neonatal Medicine,
Imperial College London, Chelsea and Westminster Campus,
369 Fulham Road, London
SW10 9NH

Dear Dr Gale

**Study Title:**	**The WHEAT trial: WithHolding Enteral feeding Around packed red cell Transfusions in preterm neonates, a multicentre, superiority, randomised registry trial**
**REC reference number:**	
**Protocol number:**	
**IRAS project ID**	

Thank you for attending the , along with Mr Hyde, to discuss your application. The documents submitted as part of your response, and subsequently reviewed by the Committee, are listed below:

Document	*Version*	*Date*
Other [Response letter to REC]	1	06 November 2014
Other [PIS]	1.4	06 November 2014
Other [Project Protocol]	1.4	06 November 2014
Other [Carlo 2013]	1	07 November 2013
Other [DAMOCLES Lancet]	1	07 November 2005
Other [EIF 123]	1	07 November 2014
Other [Junghans BMJ]	1	07 November 2005
Other [NREAP minutes]	1	17 October 2012
Other [O'Neill 2003]	1	07 November 2003
Other [Parige 2013]	1	07 November 2013
Other [Rogers 1998]	1	07 November 1998

Following discussion, the Committee agreed the following further information or clarification was required:

Further information or clarification required, along with actions resolved

Actions are listed in line with the original provisional opinion letter, taking account of discussions at the meeting and any subsequent decisions on alternative ways forward.

1. **You are required to provide evidence of scientific review.**

   The Committee are satisfied with the proposal for full review to be carried out by the NIGB, and require evidence of this review prior to approval.

2. **You are required to confirm consent in place for access to the neonatal database for the purposes of this study.**

   The Committee are satisfied with the response provided.

3. **A hard copy consent form must be created, in line with NRES guidance. The process for this must be added to the protocol.**

   The REC remained unhappy with an opt-out process. They discussed the possibility of providing a consent form for parents to evidence they had opted out, but on reflection agreed if this was possible it should be possible to provide an opt-in consent form and change the process accordingly. They noted parents could be given the consent form alongside the information sheet, to enable them to proactively sign and agree to the study. The Committee agreed it was essential parents were asked to sign an agreement (either opt-out or opt-in) to ensure they personally understood exactly what they were consenting their children to.

   The Committee felt the concept of an opt-out study would indicate to parents there was no risk involved in the study, which would influence their decision to participate. The Committee remained unconvinced there were no risks associated with the study. They acknowledged the process was easier for the research team, but did not feel it was necessarily in the best interests of participants. They acknowledged there were justifications for opt-out studies, but did not believe they applied in this case.

   The REC require the consent process to be changed to an 'opt in' system, and a consent form provided for review. This should be provided to parents alongside the information sheet, and the protocol updated to reflect this. The NRES templates can be accessed for guidance at the following link: http://www.hra.nhs.uk/resources/before-you-apply/consent-and-participation/consent-and-participant-information/

4. **The participant information sheet should be re-written/re-formatted in line with NRES guidance, to ensure all pertinent areas are covered.**

   The Committee acknowledge much work has been done to develop the information sheet, and accepted the applicant's statement that the NRES template is to be used as guidance only.

The Committee require the applicants to review and compare their information sheet to the NRES template and consider if there is any additional valuable information that can be added. Justifications should be provided should no additional information be added.

A point-by point commentary on a draft version of their PIS from the researchers relating to each NRES template paragraph would significantly assist the committee in deciding if the researchers' version was suitable for approval.

5. ***The following additional changes to the information sheet are required:***

a) ***The word 'important' must be removed from the sentence in the first paragraph reading '…we want you to know about an important study'.***

The Committee were satisfied with this response.

b) ***The risks associated with additional cannulation must be added.***

Following discussion, the Committee noted the applicant's response that extra cannulation or less cannulation may be part of standard care anyway. They asked a sentence be added to the 'Are there any risks for my baby?' section, stating being involved in the study may involve an extra cannulation or one less cannulation, depending on their randomised.

c) ***The sentence within the 'Are there any benefits for my baby?' section stating 'This non evidence based approach to neonatal care may involve more risk than being in a study like WHEAT which involves a carefully designed protocol and consistent monitoring' must be removed.***

The Committee were satisfied with this response.

6. ***A one page summary of the study must be created, to be presented alongside the full participant information sheet.***

The Committee are satisfied this may not be required, and are content to leave the judgment on this to the applicants.

7. **The following changes are required to the protocol:**

a) **Information must be added to the protocol confirming that any units that were found to have overridden the randomisation process a significant number of times, as a result of preference towards a particular approach, would be withdrawn from the study.**

The Committee were satisfied with this response.

b) **The point at which recruitment would be stopped, as a result of sufficient evidence being gathered in favour of one approach, must be clearly outlined.**

The Committee were satisfied with the applicant's guarantee that recruitment would not begin until stopping rules had been set by the Data Monitoring Committee. The Committee require a copy of the stopping rules, for information, when they have been set.

c) **You are required to clarify the safeguards in place to ensure excessive attempts at cannulation do not occur.**

The Committee were satisfied with the response given in discussion, pending inclusion of information in point 5b).

d) **The consent process must be amended to reflect the inclusion of the consent form, as above.**

The Committee requires this action to be completed, as per the original decision letter.

If you would find it helpful to discuss any of the matters raised above or seek further clarificatiohe REC Manager, Helen Wakefield, at nrescommittee.eastofengland-essex@nhs.net

When submitting a response to the Committee, the requested information should be electronically submitted from IRAS. A step-by-step guide on submitting your response to the REC provisional opinion is available on the HRA website using the following link: http://www.hra.nhs.uk/nhs-research-ethics-committee-rec-submitting-response-provisional-opinion/

Please submit revised documentation where appropriate underlining or otherwise highlighting the changes which have been made and giving revised version numbers and dates. You do not have to make any changes to the REC application form unless you have been specifically requested to do so by the REC.

Summary of the discussion at the meeting

The Committee welcomed the applicants, and informed they had reviewed their initial response and supporting evidence but still had some reservations. The main issue related to the opt-out consent process.

The researchers were asked to confirm that the application for the project was 'genuine', rather than having a concealed purpose, as the committee felt that the previous material, discussions and responses were considered to be an 'outlier' from the norm in content and tone.

The researchers confirmed the study was genuine, but did not directly confirm or deny there was any concealed purpose. The committee noted these responses, and proceeded to review.

It was agreed the applicants and Committee would go through each of the unresolved provisional opinion action points in turn, and discuss the response.

Point 3 and 7d) – A hard copy consent form must be created, in line with NRES guidance. The process for this must be added to the protocol. The consent process must be amended to reflect the inclusion of the consent form, as per point 3.

The Committee asked the applicants how the process would be protected from fraud or misunderstanding, given a 'no objection' would be recorded by the computer operator rather than by the parents themselves. *The applicants stated fraud was a possibility in any research study*. The Committee accepted this, however, they commented if a parent could take away evidence of their specific agreement to a trial this would act as a safeguard against counterfeit or consenting errors. *The applicants stated parents would still take away*

a form as part of the opt-out process. The Committee noted this, but stated they won't have carried out an action to proactively consent. *The applicants commented in an opt-in study participants are free to withdraw at any time, however, they are not asked to sign a form as evidence of withdrawal, and therefore there was no record of withdrawal in that situation*. The Committee agreed participants were not usually required to sign a withdrawal form, however, they did not believe this was the same situation. *The applicants commented, as in all studies, it was necessary to rely on the integrity of the research team.* The Committee commented it was their role to safeguard participants in cases where there may not be the level of integrity there should be, which was a key reason Research Ethics Committees existed.

The Committee acknowledged the evidence that had been provided in support of opt-out studies, and agreed there were arguments for this approach in certain cases. However, they did not feel an opt-out consent was justified in the case of this study. *The applicants commented individuals outwith the study would effectively be randomised to one or the other approach depending on the area they lived in. They added there was no particular evidence in favour of either of the approaches*.

The Committee commented it appeared 2/3rds of neonatal units did not currently feed, in line with the meta analysis which had found not feeding may be a better approach. *The applicants acknowledged this but added when approached the majority of clinics had been happy to participate, as they recognised there was not enough evidence to draw a sufficiently definite conclusion and a randomised controlled trial was needed. They added in the case of this study opt-out consent would make the whole process easier for parents, and evidence suggested participants understood the process better when it was simplified. Both approaches would be carried out as part of standard care anyway, in different clinics, and therefore the applicants did not believe being randomised as part of the trial would be any more dangerous than standard care.*

Members commented the applicants could not know whether randomisation as part of the trial was more risky until it was known which of the approaches was safer.. *The applicants agreed, however, they commented the risk of randomisation itself was not materially greater than treatment being determined by being born in one location or another.*

The Committee commented if participants were born in an area that favoured one approach, but were then randomised to the other approach, the risk may be increased for them depending on which approach is better, and due to a lower level of familiarity with the 'non-favoured' arm. They added the willingness of physicians to join the trial did not, in their view, make the opt-out approach acceptable. They commented the most important element was that parents were able to give clear and informed consent to the trial. *The applicants stated the intervention was minimal when compared to standard care. The physicians and staff involved would have much experience in the procedures involved in the trial, as they would be administered on a regular basis during standard care.*

The Committee commented it may be counter-cultural for some units to take an approach that was not their preferred or usual approach. *The applicants asked how the Committee determined the risk associated with this.* The Committee commented it may be subtle, but such a conflict could affect how procedures were conducted.

The applicants commented given the interventions involved (putting up IV fluids for a period of time), this was done very commonly on neonatal units for a variety of reasons and clinicians would be used to this. They added all babies at this gestation would have periods of no or minimal intravenous feeding. The Committee commented this would ordinarily be a clinical decision, and not the result of randomisation to a trial. The study would be asking some clinicians to take an approach outside of what would be their usual clinical decision, which therefore created an additional risk.

The applicants stated there were studies to suggest that, in neonatal studies, an opt-out process results in greater recall about the study for the parents.

<u>Point 2 – You are required to confirm consent in place for access to the neonatal database for the purposes of this study.</u>

The Committee sought to clarify consent to use the neonatal database for the study would not be in place unless the study received ethical approval. The applicant confirmed the NNRD (neonatal database) had permission to use its data for the purposes of a research database, for access by all Research Ethics Committee approved trials. The Committee were satisfied with this response.

<u>Point 4 – The participant information sheet should be re-written/re-formatted in line with NRES guidance, to ensure all pertinent areas are covered.</u>

It was noted the applicants had responded the NRES template headings provided were ‘suggested’. They stated they had benefitted from extensive parent and parent group involvement in designing the information sheet.

The applicants commented the key point was there had been a lot of parent input in the design of the information sheet over the last year, and the team had also looked at the NRES guidance and other neonatal study information sheets to inform this. The process had been driven by parents. The Committee asked whether it would be possible for the applicants to demonstrate the extensiveness of this input. *The applicant informed communications had mainly been via teleconferences.* The Committee noted the response stated there had been ‘extensive parent and parent group involvement’. *The applicants informed there had been wider involvement from BLISS, the parent group, and one parent whose child had been on a neonatal unit.*

The Committee commented the initial action was a question of ensuring all the pertinent information was covered within the information sheet, and they would need to look at this prior to providing a further response. *The applicants stated NRES were clear their template was for guidance only, and it was for each study to design an information sheet as appropriate. If the Committee felt there were any particular sentences missing to ensure parents were fully informed they would be happy to add these.* The Committee commented the NRES template was based on best practice, and was a good tool for this reason. *The applicants stated their parent representative has an awareness of the NRES template as she had been involved in other trials. The study information sheet had been compared to the NRES template and other examples and decisions had been made as the process developed in terms of what to take our or add in*. The Committee acknowledged much work had been done to develop the information sheet, and commented the response had not

reflected this as it could have done. The Committee would discuss this following the departure of the applicants and decide the way forward.

The applicants stated the NRES template is also guidance specifically for CTIMPs, and they had wished to simplify it. For example it was not felt a parent with a ill child in a neonatal unit would not want to have to read half a page of insurance statement. The team had wanted to remove all extraneous information to make the process less burdensome and more clear for parents during what was a sensitive time.

Point 5b) – The risks associated with additional cannulation must be added to the information sheet.

The applicants stated they had been clear in the IRAS form that cannulation is an element of standard care, and would not be additional in units currently withholding feeds. They added they felt adding additional information would blow the issue out of proportion. They commented they felt it would require an overly complex explanation to demonstrate that depending on which arm babies were randomised to, and depending on what each units usual standard of care would have been, babies may receive either an additional cannulation or one less cannulation than they would ordinarily have received. Given the number of cannulations would vary across units and depending on the arm they were randomised to, it made the information very difficult to convey in an understandable way.

The Committee commented as standard care depended on the unit involved, it needed to be clear babies may be receiving different treatment than usual as the study was exploring both treatment options. They asked the information be added in simple language to inform due to the differences between units across the country, babies may receive an extra cannulation or one less cannulation than they would have done, should they join the study. This would depend on which arm they were randomised to. *The applicants commented some may not need cannulation at all for standard care, or alternatively some may require additional cannulation for other clinical reasons.* The Committee noted this.

Point 6 – A one page summary of the study must be created, to be presented alongside the full participant information sheet.

The Committee acknowledged the requirement for this would depend on whether the main information sheet required significant lengthening. They agreed in its initially-submitted form a one page summary would not be necessary. They would discuss this following departure of the applicants.

Point 7b) – The point at which recruitment would be stopped, as a result of sufficient evidence being gathered in favour of one approach, must be clearly outlined.

The Committee asked when the Data Monitoring Committee and steering group would be appointed. *The applicants informed they would be appointed and have their first meeting before recruitment commenced, in line with guidance. The Data Monitoring Committee would be independent and would establish the stopping rules at their first meeting. This would be based on other elements, including safety, as well as statistical information. They confirmed they would not start the study until the stopping rules had been set, in line with the DAMOCLES terms of reference.* The Committee acknowledged this, noting it was particular important in this case that the study was stopped as soon as there was sufficient data to

demonstrate which was the better approach. *The applicants agreed, adding they had left the application blank in this respect as they wanted it to be clear the Data Monitoring Committee was independent and it was for them to decide appropriate stopping rules.*

Point 7c) – You are required to clarify the safeguards in place to ensure excessive attempts at cannulation do not occur.

The applicants stated this was a very pragmatic trial reflecting standard practice across the UK, so they had tried not to protocolise it too much to enable the results to be applicable widely. Across all units there was a general agreement that cannulations would be attempted twice on babies, before approaching the registrar for advice. This may not be formalised in a policy in all units, however, all units would have established approaches to minimise the risk. The Committee asked whether the applicants could assure them there was similar practice across the UK. *The applicants stated in their experience all units would keep cannulation attempts to a minimum as no units would want to risk harm to babies. They added they could not confirm whether all units had the procedure in writing.* The Committee noted this and would discuss the response following departure of the applicants.

Point 1 – You are required to provide evidence of scientific review for the study.

The applicants informed they were awaiting NIGB review of the application for funding, which would involve a thorough peer review. The Committee commented they would wish to see evidence of this prior to approving the study. The applicants noted this, adding the reason they had applied early was because they were aware they would need to recruit a large number of babies to the study. The simple opt out consent process, as well as achieving REC approval as quickly as possible, would help recruitment.

Following discussion of the key points, the Committee returned to the issue of an opt-out versus an opt-in approach.

The Committee asked, if they insisted on an opt-in approach, whether the applicants would be willing to begin recruitment in this way. If it appeared to be negatively affecting recruitment they could potentially submit an amendment at a later date with a rationale for changing the consent process. *The applicants noted this, but added they would prefer the opt-out consent process from the beginning.* The Committee commented they could treat it as a pilot of the opt-in (Chair's note: or opt-out) process, to see whether it negatively affected their recruitment. If it did not, they could continue . They added this was just an idea. *The applicants agreed it sounded a sensible approach, but they would need to think about it. They asked if the Committee were suggesting a formal pilot study, or just using the opt-in process initially and seeing what happened.* The Committee confirmed they were referring to the latter, rather than a formal pilot study. This would give the research team an idea as to whether the opt-out consent process was as crucial to recruitment as they thought.

The applicants noted this, but commented the opt-out consent process was not preferred purely to assist recruitment. The primary reason was about ensuring parents had a full and clear understanding of the study, by providing clear information and a simple process to follow.

Members noted parents were provided a contact if they wished to opt out at a later time, and queried what would happen if the contact wasn't available. *The applicants confirmed they could tell any member of the clinical team, who would complete the relevant paperwork and ensure they were immediately withdrawn.* Members stated this was not clear in the information sheet. *The applicants noted this.*

The Committee queried what evidence was available to show parents understood more about studies using an opt-out consent process*. The applicants informed the Euricon Project provided evidence that in opt in studies approximately 50% of parents did not fully understand the study they had consented to, although they remained happy to participate. The Committee commented this was more likely to be related to the nature and clarify of the information provided to parents, rather than whether it was an opt in or opt out process.*

The applicants informed a study in the USA showed that in opt out studies, when interviewed afterwards, parents had demonstrated a better understanding of the study than in opt in studies. They noted this was for very large trials. The Committee commented, nevertheless, the quality of information provided was the most important element to ensure understanding. *The applicants agreed, but added it appeared that less information of very good quality enabled better understanding.* The Committee commented this had been the purpose of suggesting a one page summary, should the main information sheet become lengthy. *The applicants commented there may be a risk of parents not reading the full information sheet, when provided with a summary*. The Committee agreed there was some risk, but participants would often go through the information sheet in detail at a later time, as would their friends and relatives.

The applicants informed this was the second opt out study they had sought approval for, with the first being approved. They asked whether a separate study to test the consent and understanding rates for a dummy study, one using an opt in and other an opt out process, would be valuable. The Committee commented it would probably entirely solve the Committee's concerns, however, even if not, it would be an interesting study. *The applicants added neonatal units often have good study recruitment rates, however, the main aim was to try to ensure a better understanding of the studies.*

With regard to the information sheet, the Committee commented a representative from BLISS and one parent constituted two people involved in the development of the document. They commented a small number such as this was not always as effective as involving a Patient and Public Involvement (PPI) Group who were outside of the study and could generate input from more individuals. They added they applauded the involvement of parents in the development of the document, but the involvement of a separate independent advocacy group could perhaps improve the process further. *The applicants stated they had endeavoured from the beginning to have the design of the information sheet led by parents, rather than by the study team. Unfortunately the development process had not been fully documented in order to provide evidence.*

The Committee thanked Dr Gale for attending the meeting, and Mr Hyde for joining the Committee for a second time. They added it was really helpful to have them there in person. T*he applicants thanked the Committee for their time*.

The Committee confirmed a letter regarding next steps would be sent out very shortly.

The 60 day clock for issue of a final ethical opinion on this application will not re-start until the Committee has received a satisfactory response on outstanding points.

**Please quote this number on all correspondence**

Yours sincerely

REC Manager

Email:

Copy to: *Chelsea and Westminster NHS Foundation Trust*

Imperial College London

Section of Neonatal Medicine
Imperial College London

4th Floor, Lift Bank D
Chelsea and Westminster Hospital
369 Fulham Road
London, SW10 9NH
Tel: +44 (0)203 3153519
Fax: +44 (0)203 3157998

christopher.gale@imperial.ac.uk
Dr Christopher Gale MBBS MSc PhD MRCPCH

14th January 2015

Dear Dr Lamont,

**Study title:**	**The WHEAT trial: With Holding Enteral feeding Around packed red cell Transfusions in preterm neonates, a multicentre, superiority, randomised registry trial**
**REC reference:**	
**Protocol number:**	
**IRAS project ID:**	

Thank you for taking the time to review the WHEAT trial. Please find responses detailed below to your correspondence following the discussion on . For clarity we have included only points where further action was required.

1. You are required to provide evidence of scientific review.
The Committee are satisfied with the proposal for full review to be carried out by the NIGB, and require evidence of this review prior to approval.

- We would like to make it clear to the committee that full review will be carried out by the National Institute of Health Research (NIHR) not the NIGB (National Information Governance Board) (the NIGB no longer exists; its functions, which do not include routine peer review, have been taken over by the Confidentiality Advisory Group of the Health Research Authority).

3. A hard copy consent form must be created, in line with NRES guidance. The process for this must be added to the protocol.
The REC remained unhappy with an opt-out process. They discussed the possibility of providing a consent form for parents to evidence they had opted out, but on reflection agreed if this was possible it should be possible to provide an opt-in consent form and change the process accordingly. They noted parents could be given the consent form alongside the information sheet, to enable them to proactively sign and agree to the study. The Committee agreed it was essential parents were asked to sign an agreement (either opt-out or opt-in) to ensure they personally understood exactly what they were consenting their children to.
The Committee felt the concept of an opt-out study would indicate to parents there was no risk involved in the study, which would influence their decision to participate. The Committee remained unconvinced there were no risks associated with the study. They acknowledged the process was easier for the research team, but did not feel it was necessarily in the best interests of participants. They acknowledged there were justifications for opt-out studies, but did not believe they applied in this case. The REC require the consent process to be changed to an 'opt in' system, and a consent form provided for review. This should be provided to parents alongside the information sheet, and the protocol updated to reflect this. The NRES templates can be accessed for guidance at the following link: http://www.hra.nhs.uk/resources/before-you-apply/consent-and-participation/consent-and-participant-information/

- For the reasons outlined in our previous correspondence (improved understanding of the research study by parents, a less biased sample, more generalisable results and more rapid resolution of clinical uncertainty) we feel that an opt-out design is optimal in a low risk

comparative effectiveness trial such as WHEAT. We are not willing to change the design to opt-in consent.

4. The participant information sheet should be re-written/re-formatted in line with NRES guidance, to ensure all pertinent areas are covered.
The Committee acknowledge much work has been done to develop the information sheet, and accepted the applicant's statement that the NRES template is to be used as guidance only. The Committee require the applicants to review and compare their information sheet to the NRES template and consider if there is any additional valuable information that can be added. Justifications should be provided should no additional information be added.
A point-by point commentary on a draft version of their PIS from the researchers relating to each NRES template paragraph would significantly assist the committee in deciding if the researchers' version was suitable for approval.

- We have provided a point by point summary as requested.

5. The following additional changes to the information sheet are required:
b) The risks associated with additional cannulation must be added.
Following discussion, the Committee noted the applicant's response that extra cannulation or less cannulation may be part of standard care anyway. They asked a sentence be added to the 'Are there any risks for my baby?' section, stating being involved in the study may involve an extra cannulation or one less cannulation, depending on their randomised.

- The Participant Information Sheet has been amended.

6. A one page summary of the study must be created, to be presented alongside the full participant information sheet.
The Committee are satisfied this may not be required, and are content to leave the judgment on this to the applicants.

- Thank you, we judge that this is not required.

7. The following changes are required to the protocol:
b) The point at which recruitment would be stopped, as a result of sufficient evidence being gathered in favour of one approach, must be clearly outlined.
The Committee were satisfied with the applicant's guarantee that recruitment would not begin until stopping rules had been set by the Data Monitoring Committee. The Committee require a copy of the stopping rules, for information, when they have been set.

- Copies will be provided when available.

d) ***The consent process must be amended to reflect the inclusion of the consent form, as above.***
The Committee requires this action to be completed, as per the original decision letter.

- Please see our response to point 3, above.

Documents attached:

Document	Version	Date
Participant Information Sheet	1.5	14 January 2015
Participant Information Sheet - point by point summary		14 January 2015

Please do not hesitate to contact me if you require any further information.
Yours sincerely

Dr Chris Gale, NIHR Clinical Lecturer in Paediatrics

We have copied the template below from the Consent and Participant Information Sheet Preparation Guidance (March 2014) as found on the HRA website: http://www.hra-decisiontools.org.uk/consent/docs/PIS-Template_version1.pdf

The template from the HRA is in blue, we have cut responded to each point in turn, in red, quoting directly from the Participant Information Sheet using italics.

Participant Information Sheet (PIS) Template

This is not offered as a rigid template, but rather a flexible framework.

We have suggested sub-headings which you may decide are appropriate to use or not, depending on the type of study you are planning and what is involved.

Remember the aim of a PIS is to provide sufficient information, in an understandable format to support potential participants in making the right decision for them: to take part in your study, or to decline participation.

Study title

Remember: I.P.O.C - Intervention, Population, Outcome, Comparator (if appropriate) is a rule that helps produce a meaningful study title.

- We have deliberately kept the study title brief but can put it in a more I.P.O.C. form (below)
- The WHEAT Study: Does withholding milk feeds around transfusion in preterm babies reduce necrotising enterocolitis when compared with continuing milk feeds?

Invitation and brief summary

Potential participants should be given very brief information about your study: just enough to decide if they wish to read further.

- We provided a brief invitation about the study in the first paragraph: "*We understand this is a difficult time and it may not seem a good moment to ask you to think about something extra, but we want you to know about a study for premature babies (babies born before their due date).*"

There may be specific issues to address here when you are inviting someone else to give consent on behalf of another, or you are consulting someone to give their opinion on the inclusion of another (e.g. adults not able to consent for themselves)

What's involved?

Explanation: purpose of and background to the research and invitation

What is the nature of what you are proposing?

- *"If your baby needs a blood transfusion, and is receiving milk feeds, the decision about whether to stop or continue feeds during the transfusion will be decided by a process called "randomisation"."*

Why are you doing this research? What is already known?

- *"It is quite common for premature babies to have blood transfusions because they become anaemic (they do not have enough red blood cells). Premature babies are also vulnerable to a bowel condition called necrotising enterocolitis (NEC). This can be serious and can have long-term effects on how babies grow and develop. Some doctors worry that feeding babies during a blood transfusion may increase the risk of NEC, others however, think that it is more risky to stop feeds; the problem is that we do not know. Because of this, the way babies are cared for during blood transfusions varies across the country; some babies have feeds stopped before, during and after a transfusion (around 12 hours in total), and others have feeds continued. The purpose of WHEAT is to determine which approach is best."*

How many will be involved in the study?

- *"WHEAT is taking place in neonatal units all over the UK and will involve about 4,500 babies."*

What alternatives are available to potential participants'?

- *"If you choose not to take part your baby will still have feeds either stopped or continued during blood transfusions in the same way as in WHEAT but the decision will be made by the local clinical team and the policy of the neonatal unit, and information about your baby will not be included in the study."*

You should try to keep this brief and avoiding cutting and pasting directly from a protocol; keep your language understandable.

What would taking part involve?

You should give potential participants an idea of what they should expect if they agree to take part. It is important that you consider their perspective and likely view of any impacts on them, their lives and those close to them.

- *"If your baby needs a blood transfusion, and is receiving milk feeds, the decision about whether to stop or continue feeds during the transfusion will be decided by a process called "randomisation". Randomisation is done by computer and means that every baby has an equal chance of either having feeds stopped or continued. If your baby is randomised to have feeds stopped this will be for 4 hours before, after, and during this and any subsequent blood transfusions. It is quite common for premature babies to have their feeds withheld for a number of reasons. When this happens babies are given nutrition into a vein by drip to ensure their blood sugar level does not drop and to reduce any feelings of hunger they might have. Babies in WHEAT who have their milk feeds stopped around a blood transfusion will be given nutrition into a vein in the same way. If your baby is randomised to have feeds continued, there will be no change in how your baby is fed."*

Potential participants need to know what they are being asked to give consent to, so make it clear what elements are additional to standard care, and/or what elements of standard care they may not receive if they agree to take part.

- *"Babies in WHEAT will not have any extra tests and in all other respects will be looked after in the same way as a premature baby not taking part in the study."*

There will be specific issues pertinent to your particular study and the types of participant you intend to recruit which must be considered here (e.g. adults not able to consent for themselves or children / young people). Specific issues may include:

- Impacts on possible pregnancy and breast feeding, including young people and pregnancy
  - Not applicable in the neonatal population
- In therapeutic research – what are the clinical alternatives
  - *"If you choose not to take part your baby will still have feeds either stopped or continued during blood transfusions in the same way as in WHEAT but the decision will be made by the local clinical team and the policy of the neonatal unit, and information about your baby will not be included in the study."*
- Randomisation and blinding
  - *"the decision about whether to stop or continue feeds during the transfusion will be decided by a process called "randomisation". Randomisation is done by computer and means that every baby has an equal chance of either having feeds stopped or continued."*
  - WHEAT is unblinded so it is not appropriate to discuss blinding
- Screening and exclusion
  - Not applicable to WHEAT as it does not involve screening and has no exclusion criteria
- Therapeutic studies - what happens when the research study stops?
  - Not applicable to WHEAT as it is a comparative effectiveness research project comparing routinely practised approaches and will only include infants while they are on the neonatal unit. The study continues until all included babies have been discharged from the neonatal unit and are therefore not eligible for the different treatment arms.
- Tissue samples
  - Not applicable to WHEAT as it does not involve tissue samples
- Research databases and tissue banks
  - In WHEAT parents will have already agreed to inclusion of their baby's details in the National Neonatal Research Database (NNRD)
- Expenses and payments
  - Not applicable to WHEAT as it does not involve expenses or payments
- Genetic research
  - Not applicable to WHEAT as it does not involve genetics samples
- Exposure to ionising radiation
  - Not applicable to WHEAT as it does not involve ionising radiation
- Accessing ONS, GROS and other registry data
  - Not applicable to WHEAT other registry data
- Generic consent etc.
  - Not applicable to WHEAT

What are the possible benefits of taking part?

It is likely that you cannot guarantee any specific treatment benefits, and this should be made clear to potential participant. However, research does deliver wider benefits to society / others with a similar condition and some indirect benefits might be foreseeable for participants themselves.

- ***"Are there any benefits for my baby?***
  Each of the two options in the WHEAT study is currently used by doctors in the UK because we do not know which one is better. Taking part in a research study may confer non-specific benefits."

What are the possible disadvantages and risks of taking part?

You should include details of all significant risks of harm, risks to confidentiality and psychological risk.

- ***"Are there any risks for my baby?***
  There are no risks for your baby from taking part in WHEAT. Rarely, being involved in WHEAT may involve an either an extra intravenous drip or one less intravenous drip, depending on how your baby is randomised."

Some specific issues you should consider include:

• Impact on possible pregnancy and breast feeding, including young people and pregnancy

- Not applicable in the neonatal population

• Side effects of treatments / therapies in trials

- *"If your baby is randomised to have feeds stopped this will be for 4 hours before, after, and during this and any subsequent blood transfusions. It is quite common for premature babies to have their feeds withheld for a number of reasons. When this happens babies are given nutrition into a vein by drip to ensure their blood sugar level does not drop and to reduce any feelings of hunger they might have. Babies in WHEAT who have their milk feeds stopped around a blood transfusion will be given nutrition into a vein in the same way."*

• Discovering health related findings

- ***"What if relevant new information becomes available?***
  If new information becomes available during the study this will be evaluated by an independent Data Monitoring Committee who will advise whether or not WHEAT should continue. "

• Impact on insurance

- Not applicable in this comparative effectiveness trial

• Ionising radiation etc.

- Not applicable to WHEAT as it does not involve ionising radiation

Try to describe the likelihood of adverse things happening, as well as severity in language all potential participants are likely to understand.

Further supporting information

Finally you should provide potential participants with more details of what is involved so that you can fully support them in making an appropriate decision.

Some of the issues that might be appropriate here include:

- What if something goes wrong?
  - ***"What if there is a problem?***
    If at any stage you have any questions about the study or the way it has been carried out, please contact the Local Principal Investigator or Local Research Nurse (contact details below)."
- What will happen if I don't want to carry on with the study?
  - ***"What if I change my mind and decide to withdraw my baby from WHEAT at a later date?***
    You can change your mind and opt-out of WHEAT at any time and without having to give a reason. Just let us know."
- How will my information be kept confidential?
  - ***"Will my taking part in WHEAT be kept confidential?***
    We will record relevant information about you and your baby; this will be kept securely and will only be seen by the study team and authorized regulatory authorities. You and your baby will not be identified in any report or publication about WHEAT."
- What will happen to the results of this study?
  - ***"What will happen to the results of WHEAT?***
    The results will be used to improve the way premature babies are looked after. The results will be published in a medical journal. We will also send a copy of the results to you."
- Who is organising and funding this study?
  - ***"Who is managing and funding WHEAT?***
    WHEAT is managed by the Clinical Trials Unit at the University of Manchester. It is funded by the Health Technology Assessment programme of the National Institute for Health Research."
- How have patients and the public been involved in this study?
  - We have not included any mention of the extensive parent and parent representative involvement in the Participant Information Sheet but could do if requested.
- Who has reviewed this study?
  - ***"Who is managing and funding WHEAT?***
    WHEAT is managed by the Clinical Trials Unit at the University of Manchester. It is funded by the Health Technology Assessment programme of the National Institute for Health Research."
- Further information and contact details
  - *"Thank you for reading this leaflet; please discuss WHEAT with the doctor or nurse who is looking after your baby if you have any questions."*

**Local Principal Investigator**	**Local Research Nurse**	**Lead Investigators**
		Professor Neena Modi &<br>Dr Chris Gale<br>Imperial College London,<br>0203 315 5101<br>n.modi@imperial.ac.uk<br>christopher.gale@imperial.ac.uk

- What to expect during the consent process

- *We are comparing practices that already take place in neonatal units in the UK and are offering every baby the opportunity to participate. Your baby does not have to take part if you don't want them to, in this case please tell a member of the local clinical team (names and contact details are provided at the end of this leaflet) that you would like to "opt-out" (have your baby excluded from the WHEAT study).*

  If you do want your baby to take part in WHEAT, you don't need to do anything.

  What will happen if I opt-out?

  If you "opt-out" your baby will still have feeds either stopped or continued during transfusions in the same way as in WHEAT but the decision will be made by the local clinical team and the policy of the neonatal unit, and information about your baby will not be included in the study.

- What if relevant new information becomes available?
  - ***"What if relevant new information becomes available?***
    If new information becomes available during the study this will be evaluated by an independent Data Monitoring Committee who will advise whether or not WHEAT should continue."
- Involvement of General Practitioner / other healthcare practitioner
  - Not applicable as the General Practitioner will not be involved, and the babies will not have been discharged into the community as yet, so will have no registration with a General Practitioner.
- What will happen to the samples I give?
  - Not applicable to WHEAT as it does not involve samples
- Commercial exploitation etc.
  - Not applicable to WHEAT as it is not a commercial study

Version control

All of your consent documents (and other study documents) should have a version number and/or date, to ensure that any changes or amendments can be more easily implemented.

- Version control is already used on all documents

Health Research Authority

21 November 2014

Dr Chris Gale
NIHR Clinical Lecturer
Imperial College London
Section of Academic Neonatal Medicine,
Imperial College London, Chelsea and Westminster Campus,
369 Fulham Road, London
SW10 9NH

Dear Dr Gale

**Study Title:**	**The WHEAT trial: WithHolding Enteral feeding Around packed red cell Transfusions in preterm neonates, a multicentre, superiority, randomised registry trial**
**REC reference number:**	
**Protocol number:**	
**IRAS project ID**	

Thank you for your letter of 10 November 2014, responding to the Committee's request for further information on the above research, and enclosing the following revised documents:

Document	*Version*	*Date*
Other [Response letter to REC]	1	06 November 2014
Other [PIS]	1.4	06 November 2014
Other [Project Protocol]	1.4	06 November 2014
Other [Carlo 2013]	1	07 November 2013
Other [DAMOCLES Lancet]	1	07 November 2005
Other [EIF 123]	1	07 November 2014
Other [Junghans BMJ]	1	07 November 2005
Other [NREAP minutes]	1	17 October 2012
Other [O'Neill 2003]	1	07 November 2003
Other [Parige 2013]	1	07 November 2013
Other [Rogers 1998]	1	07 November 1998

The further information and revised documentation has been considered on behalf of the Committee by the Chair.

Unfortunately, the Committee was not satisfied with the responses to any of the points raised in the provisional opinion letter, and would be grateful for a more complete response in line with the actions required.

Given the complexity of the issues and the apparent disparity of opinion, the Chair would like to discuss your response in more detail at the next full meeting of the Research Ethics Committee, in order to establish the best way forward.

The Chair expressed regret that the Chief Investigator was unable to attend the previous meeting, and would welcome his attendance at the next meeting to enable a full and productive discussion. The meeting will take place on

The Chair would welcome the opportunity to discuss the research application with the Chief Investigator in advance of this meeting. Please contact the REC Manager, who can provide you with the relevant contact details for this.

The 60 day clock for issue of a final ethical opinion on this application will not re-start until the Committee has received a satisfactory response on outstanding points.

**Please quote this number on all correspondence**

Yours sincerely

REC Manager

Email:

Copy to:

Chelsea and Westminster NHS Foundation Trust

NHS
Health Research Authority

20 January 2015

Dr Chris Gale
NIHR Clinical Lecturer
Imperial College London
Section of Academic Neonatal Medicine,
Imperial College London, Chelsea and Westminster Campus,
369 Fulham Road, London
SW10 9NH

Dear Dr Gale

**Study title:**	**The WHEAT trial: WithHolding Enteral feeding Around packed red cell Transfusions in preterm neonates, a multicentre, superiority, randomised registry trial**
**REC reference:**	
**Protocol number:**	
**IRAS project ID:**	

Thank you for your letter of 14 January 2015 responding to the Committee's request for further information on the above research.

The further information has been considered on behalf of the Committee by the Chair, in consultation with other members.

We plan to publish your research summary wording for the above study on the HRA website, together with your contact details. Publication will be no earlier than three months from the date of this favourable opinion letter. The expectation is that this information will be published for all studies that receive an ethical opinion but should you wish to provide a substitute contact point, wish to make a request to defer, or require further information, please contact the REC Manager,

Under very limited circumstances (e.g. for student research which has received an unfavourable opinion), it may be possible to grant an exemption to the publication of the study.

Ethical opinion

The Committee is unable to give a favourable ethical opinion of the research, for the following reasons:

1. The Committee does not consider the response to be acceptable, as the researchers refuse to move to a documented, signed-for record of consent, whether opt-in or opt-out. The Committee does not feel it is acceptable not to formally record the consent process, and draws on the following guidance in support of this stance:

   a) The procedure should follow the requirements of clause 24 of the Declaration of Helsinki:

      "After ensuring that the potential subject has understood the information, the physician or another appropriately qualified individual must then seek the potential subject's freely-given informed consent, preferably in writing. If the consent cannot be expressed in writing, the non-written consent must be formally documented and witnessed."

   b) HEALTH TECHNOLOGY ASSESSMENT 2014 VOL. 18 NO. 42 (Death, bereavement and randomised controlled trials (BRACELET): a methodological study of policy and practice in neonatal and paediatric intensive care trials Claire Snowdon, Peter Brocklehurst, Robert Tasker, Martin Ward Platt, Sheila Harvey and Diana Elbourne) advises that in trials concerning neonates who may die:

      "The significance of trial paperwork

      Within CTUs, we need to be aware, when writing trial communications, that the BRACELET study has shown that much of the trial-related paperwork (letters, consent forms, information leaflets, newsletters etc.) might be kept among the different valued items that parents had preserved in memory of their child's short life. This might argue for producing items which are durable, for instance printed on a high quality paper, and which articulate clearly the importance of the contribution that babies and families make to a trial."

The Chair wishes to advise that there is an opportunity to appeal through the NRES system, and the Committee would be delighted to see an outcome that satisfies all concerned.

I regret to inform you therefore that the application is not approved.

If you would find it helpful to discuss any of the matters raised above or seek further clarification from a member of the Committee, you are welcome to contact the REC Manager,

Options for further ethical review

You may submit a new application for ethical review, taking into account the Committee's concerns. You should enter details of this application on the application form and include a copy of this letter, together with a covering letter explaining what changes have been made from the previous application. We strongly recommend that you submit the new application to this REC. However, you may submit the application to a different REC if you prefer. The application should be booked through the Central Booking Service (CBS) and would be allocated for review in the normal way. You should let CBS know if you would like the application to be reviewed again by this Committee.

Alternatively, you may appeal against the decision of the Committee by seeking a second opinion on this application from another Research Ethics Committee. The appeal would be

based on the application form and supporting documentation reviewed by this Committee, without amendment. If you wish to appeal, you should notify the relevant Research Ethics Service Manager (see below) in writing within 90 days of the date of this letter. If the appeal is allowed, another REC will be appointed to give a second opinion within 60 days and the second REC will be provided with a copy of the application, together with this letter and other relevant correspondence on the application. You will be notified of the arrangements for the meeting of the second REC and will be able to attend and/or make written representations if you wish to do so.

The contact point for appeals is:

HRA Improvement & Liaison Manager
National Research Ethics Service

Email:

Documents reviewed

The final list of documents reviewed by the Committee is as follows:

Document	*Version*	*Date*
Covering letter on headed paper		05 September 2014
Evidence of Sponsor insurance or indemnity (non NHS Sponsors only)		21 July 2014
IRAS Checklist XML [Checklist_14012015]		14 January 2015
Letter from sponsor		21 August 2014
Other [Response letter to REC]	1	06 November 2014
Other [Project Protocol]	1.4	06 November 2014
Other [Carlo 2013]	1	07 November 2013
Other [DAMOCLES Lancet]	1	07 November 2005
Other [EIF 123]	1	07 November 2014
Other [Junghans BMJ]	1	07 November 2005
Other [NREAP minutes]	1	17 October 2012
Other [O'Neill 2003]	1	07 November 2003
Other [Parige 2013]	1	07 November 2013
Other [Rogers 1998]	1	07 November 1998
Other [Response Letter]	2	14 January 2015
Other [PIS Point by point amendment]	1	14 January 2015
Other [Amended PIS]	1.5	14 January 2015
REC Application Form [REC_Form_10092014]		08 September 2014
Summary CV for Chief Investigator (CI)	1	05 September 2014

Statement of compliance

The Committee is constituted in accordance with the Governance Arrangements for Research Ethics Committees and complies fully with the Standard Operating Procedures for Research Ethics Committees in the UK.

After ethical review

You are invited to give your view of the service you have received from the National Research Ethics Service and the application procedure. If you wish to make your views known please use the feedback form available on the HRA website: http://www.hra.nhs.uk/about-the-hra/governance/quality-assurance/

We are pleased to welcome researchers and R & D staff at our NRES committee members' training days – see details at http://www.hra.nhs.uk/hra-training/

**Please quote this number on all correspondence**

Yours sincerely

Chair

Email:

Copy to: *Chelsea and Westminster NHS Foundation Trust*
