# Supplementary material for: Research ethics committee decision-making in relation to an efficient neonatal trial
Source: Arch Dis Child Fetal Neonatal Ed. 2016 Sep 14;102(4):F291–8. doi: 10.1136/archdischild-2016-310935 (PMC5537508; doi:10.1136/archdischild-2016-310935)
Supplement: Supplementary data [file fetalneonatal-2016-310935supp007.pdf]

NHS
Health Research Authority

07 October 2014

Dr Chris Gale
NIHR Clinical Lecturer
Imperial College London
Section of Academic Neonatal Medicine,
Imperial College London, Chelsea and Westminster Campus,
369 Fulham Road, London
SW10 9NH

Dear Dr Gale

**Study Title:**	**The WHEAT trial: WithHolding Enteral feeding Around packed red cell Transfusions in preterm neonates, a multicentre, superiority, randomised registry trial**
**REC reference:**	
**Protocol number:**	
**IRAS project ID:**	

The Research Ethics Committee reviewed the above application at the meeting held on . Thanks were expressed to Mr Matthew Hyde for being available by telephone to discuss the application.

Provisional opinion

The Committee is unable to give an ethical opinion on the basis of the information and documentation received so far. Before confirming its opinion, the Committee requests that you provide the further information set out below.

Authority to consider your response and to confirm the Committee's final opinion has been delegated to a meeting of the Sub-Committee of the REC.

Further information or clarification required

1. The Committee requests further justification for the necessity of approaching parents within the first 24 hours of their babies being admitted to NICU. As the researcher stated in discussion with the Committee, it is unlikely that babies will need a blood transfusion within the first 24 to 48 hours, therefore the Committee sees no reason why parents need to be approached regarding the study during this timeframe, which is very stressful and emotionally charged period. Approaching parents during this time will significantly reduce the validity of any decision making and undermines the principle of fully informed consent, whether this is opt-in or opt-out.

2. The Committee sees no reason why parents cannot be introduced briefly to the study by

providing an invitation letter during the early part of the admission, but then fully informed of the research by providing a PIS and time for a discussion when it becomes apparent that their baby needs a blood transfusion. This would also have the merit of reducing the number of babies recruited to the study who will not then be randomised due to their not requiring a blood transfusion.

3. The Committee requests further justification for the proposed use of opt-out consent. The Committee agrees that there needs to be a balance of the rigour of scientific design against the ethical implications of using this method. In this connection please supply details of the study that had been mentioned as an example involving opt-in consent.

4. The Committee requests further justification for not requiring a physical confirmation of the parents' understanding and consent decision regarding the study. This could take many forms, including an electronic signature or other method of recording the decision. The Committee has concerns that the absence of any form of acknowledgment of a consent decision from the parents lacks rigour, and does not feel that it is adequate for a researcher just to tick a box on an electronic database. In particular, consent normally involves a sequence of items listed so as to ensure adequate understanding of what is involved.

5. The Committee requests clarification of the process should a parent decide to opt-out of the study, and confirmation that no undue pressure or influence would be placed on them, even if unintentionally (for example, by asking them to sign a form to opt-out, when this is not required at the time of initial consent).

6. The Committee requests that the Protocol is amended to formally state as an exclusion criteria for the study, that any babies whose parents have not agreed to the inclusion of their data on the registry database for research purposes, will not be included.

7. The Committee requests that a statement is added to the PIS explaining that, due to participation in the study, some babies may require an additional IV line that they might not otherwise require in routine clinical care.

8. The Committee requests that the PIS is amended as follows:
   a. Please remove the final sentence of the section 'Are there any benefits for my baby?'. It is disingenuous to include this statement about "non-evidence based approach" in the PIS since evidence based care simply means care that is compatible with the current state of evidence.
   b. In the same section, please amend the second sentence to read: "For babies not taking part in WHEAT, the decision of whether or not to stop feeds is made according to the usual clinical practice of your local medical team."
   c. In the section 'Why has my baby been chosen?', please add the following sentence: "You have agreed for your baby's data to be used for research purposes in the national registry of premature babies (the National Neonatal Research Database)."
   d. In the section 'Does my baby have to take part?', please start the section with "No." Please then move the second sentence to follow this one.
   e. In the section 'What will happen to the results of WHEAT?', please amend the first sentence to read: "We hope that the results may be used…"

If you would find it helpful to discuss any of the matters raised above or seek further clarification from a member of the Committee, you are welcome to contact

REC Manager.

When submitting a response to the Committee, the requested information should be electronically submitted from IRAS. A step-by-step guide on submitting your response to the REC provisional opinion is available on the HRA website using the following link: http://www.hra.nhs.uk/nhs-research-ethics-committee-rec-submitting-response-provisional-opinion/

Please submit revised documentation where appropriate underlining or otherwise highlighting the changes which have been made and giving revised version numbers and dates. You do not have to make any changes to the REC application form unless you have been specifically requested to do so by the REC.

The Committee will confirm the final ethical opinion within a maximum of 60 days from the date of initial receipt of the application, excluding the time taken by you to respond fully to the above points. A response should be submitted by no later than 06 November 2014.

Summary of the discussion at the meeting

Recruitment arrangements and access to health information, and fair participant selection

The Committee sought clarification of the normal time period between the babies' first 24 hours in NICU and their first blood transfusion.

The researcher stated that this time period was variable, but it was unusual for babies to require a blood transfusion within the first 48 hours in NICU.

The Committee sought justification for the need for parents to be approached regarding the study during their baby's first 24 hours on NICU.

The researcher stated that it would be better for a clinician to answer this question, but stated that they had chosen this approach to ensure that they include as many babies in the study as possible and it will be easiest to approach their parents during this time period when the clinicians are speaking to them about treatment and care.

Informed consent process and the adequacy and completeness of participant information

The Committee sought clarification of the primary justification for choosing an opt-out consent method.

The researcher stated that the primary reason that they had chosen this approach was to increase the recruitment rate while reducing the burden of decision making on parents. He stated that both treatment methods are used in routine clinical care. He stated that the research team felt that participants having to make a decision to opt-in can feel more pressured than making a decision to opt-out. He stated that, due to the need to move the field forward with regards to this condition, the researchers need to include as many babies as possible.

The Committee stated that the researchers could extend the time period to recruit the numbers required for the study.

The researcher stated that as there is as yet no evidence base for either treatment, it would be unethical to continue to expose babies to a treatment that might prove to be detrimental, and that it would be unethical to extend the time taken to determine which one was detrimental.

The Committee stated that, as there is genuine equipoise, the study could demonstrate that both treatment methods are equally beneficial or harmful.

The Committee queried the likely impact on a parent of offering 'opt-out' consent if their baby then dies, and whether or not in this litigious age the researcher might feel more threatened in the absence of a conventional opt-in consent form signed by the participant.

The researcher stated that he felt they would be well able to justify the study itself and the consent process. He had no particular fears of legal action.

The Committee asked for further clarification of the details of electronic procedure for taking and recording consent.

The researcher stated that the 'consent' or decision to opt out will be recorded electronically on the database which is used to record all the babies' data. The database will be programmed to automatically ask if parents have discussed the study with clinicians, and then if they have decided to opt-out. He confirmed that babies will only be enrolled and randomised to a treatment arm after both these data screens have been completed. If the parents later changed their mind and withdrew their baby, their data will be removed from the database.

The Committee sought confirmation of the accuracy of recording a decision to opt-out on the database, and whether this could accurately reflect the discussion to ensure that the decision was fully informed.

The researcher stated that the database is fully auditable, and all information recorded on it is date and time stamped and identifies the person recording the data. He stated that, in this sense, the database could be considered to be a more accurate record than a paper consent form, which could easily be misplaced. He stated that this is how clinical consent is taken and recorded on a daily basis in practice, so he was not sure why it would be considered to be different for research.

Independent review

The Committee was pleased to note that there had been extensive Patient and Public Involvement in the design of the study as well as extensive review as part of the funding application. However, the Committee sought clarification as to whether or not specific views had been sought on the proposal for opt-out consent.

The researcher stated that this had been discussed, and that parents had been involved since the beginning in designing the research and had been very supportive of the proposal. He stated that the study is similar in design to another on-going study where they have successfully used opt-out consent.

Other ethical issues were raised and resolved in preliminary discussion before your attendance at the meeting.

NHS
Health Research Authority

Documents reviewed

The documents reviewed at the meeting were:

Document	*Version*	*Date*
Covering letter on headed paper		05 September 2014
Evidence of Sponsor insurance or indemnity (non NHS Sponsors only)		21 July 2014
Letter from sponsor		21 August 2014
Participant information sheet (PIS)	1.3	02 September 2014
REC Application Form [REC_Form_10092014]		10 September 2014
Research protocol or project proposal	1.3	11 August 2014
Summary CV for Chief Investigator (CI)	1	05 September 2014

Membership of the Committee

The members of the Committee who were present at the meeting are listed on the attached sheet

Statement of compliance

The Committee is constituted in accordance with the Governance Arrangements for Research Ethics Committees and complies fully with the Standard Operating Procedures for Research Ethics Committees in the UK.

Please quote this number on all correspondence

Yours sincerely

Chair

Email:

Enclosures: *List of names and professions of members who were present at the meeting and those who submitted written comments.*

Copy to: *Chelsea and Westminster NHS Foundation Trust*

Health Research Authority

Attendance at Committee meeting

Committee Members:

Name	*Profession*	*Present*
	Clinical Psychologist (Retired)	Yes
	Consultant Urological Surgeon	Yes
	Pharmacist	Yes
	Lecturer, Mental Health Studies	Yes
	Lecturer in Radiography	Yes
	Oncology Research Nurse	No
	Mathematician (Retired)	Yes
	Chartered Engineer (Retired)	Yes
	Principal Lecturer - Public Health, Social Care and Research Design Lead	Yes
	Principal Psychology Lecturer	No
	Course Leader, M.Sc. Clinical Exercise Science	No
	Consultant Psychiatrist	Yes

Also in attendance:

Name	*Position (or reason for attending)*
	REC Manager

Section of Neonatal Medicine
Imperial College London

4th Floor, Lift Bank D
Chelsea and Westminster Hospital
369 Fulham Road
London, SW10 9NH
Tel: +44 (0)203 3153519
Fax: +44 (0)203 3157998

christopher.gale@imperial.ac.uk

Imperial College London

15th October 2014

Dr Christopher Gale MBBS MSc PhD MRCPCH

Dear

Study title: **The WHEAT trial: With Holding Enteral feeding Around packed red cell Transfusions in preterm neonates, a multicentre, superiority, randomised registry trial**

REC reference:
Protocol number:
IRAS project ID:

Thank you for taking the time to review the WHEAT trial. Please find responses to your requests for further information detailed below:

1. The Committee requests further justification for the necessity of approaching parents within the first 24 hours of their babies being admitted to NICU. As the researcher stated in discussion with the Committee, it is unlikely that babies will need a blood transfusion within the first 24 to 48 hours, therefore the Committee sees no reason why parents need to be approached regarding the study during this timeframe, which is very stressful and emotionally charged period. Approaching parents during this time will significantly reduce the validity of any decision making and undermines the principle of fully informed consent, whether this is opt-in or opt-out:

We would like to emphasise that the WHEAT trial is comparing two treatment options that are both considered standard of care in the UK. All preterm infants born in the UK who require a blood transfusion while on a neonatal unit will currently receive one of the treatment options being compared in WHEAT; they will either have feeds withheld or feeds continued around the blood transfusion. At present the decision as to whether an individual baby receives one treatment or the other depends on the clinician looking after them at the time of blood transfusion or the neonatal unit they are admitted to. All WHEAT is doing is randomising the decision in order to determine which approach is best. WHEAT does not involve any new or experimental treatment. Because WHEAT is simply a randomised evaluation of existing treatment choices we would like to introduce it to parents during their first few days on the neonatal unit, when a discussion about the neonatal unit and about research is neonatal care unit would normally take place.

The opt-out consent process that we intend to use reflects our wish to help parents appreciate that WHEAT is a comparison of treatments already in accepted use rather than an evaluation of a new or experimental therapy. As such there should be no pressurised time limit for parents to decide whether or not to take part. Instead we aim to achieve a continuing dialogue about the way in which we seek to reduce widespread uncertainties in clinical care. We empower parents with full ability to opt-out at any time during their baby's neonatal unit stay. The initial approach (in the first 24-48 hours) will be a simple explanation of the study.

Prior to giving each blood transfusion, in accordance with the principles of Good Clinical Practice in Clinical Trials, the local research team will confirm that parents are happy to continue participation.

2. The Committee sees no reason why parents cannot be introduced briefly to the study by providing an invitation letter during the early part of the admission, but then fully informed of the research by providing a PIS and time for a discussion when it becomes apparent that their baby needs a blood transfusion. This would also have the merit of reducing the number of babies recruited to the study who will not then be randomised due to their not requiring a blood transfusion.

We do not consider this the best approach for the following reasons:

- *Many blood transfusions on a neonatal unit are given urgently or as an emergency (they are commonly given out of hours), therefore delaying discussion until a transfusion is required will shorten the period over which parents are able to consider and reflect on their decision (because the transfusion is clinically indicated and not readily delayed), and result in many parents not being offered the choice to participate in WHEAT (because they may be difficult to contact at short notice when a transfusion is indicated).*

- *Infants often need a blood transfusion when they are (or appear to be) more unwell; they have symptoms such as problems breathing or a fast heart rate. This is often a very stressful and difficult time for parents. Delaying discussion of WHEAT until this point would be more emotionally charged and stressful than explaining it in the first 24-48 hours and allowing parents time to fully consider and reflect on their baby's involvement.*

- *We would expect more than 95% of babies eligible for WHEAT to require a blood transfusion during their neonatal unit stay. Delaying discussion about WHEAT until the point that a baby needs a blood transfusion will not therefore substantially reduce number of babies randomised but not requiring transfusion.*

We agree with the committee that presenting parents with a complicated Participant Information Sheet on admission is not optimal, but we feel on balance the benefits to both the parents, and the study, of presenting them with the information within the first 48 hours of admission, rather than at the time of blood transfusion, outweigh the disadvantages. With the burden on the parents in mind we have designed the Participant Information Sheet (with considerable parent and parent group involvement, and to be as short, simple and informative as is possible. As we state above we hope to change the act of "consenting" for a trial into an ongoing dialogue between parents and the clinical and research staff, where parents are empowered to opt-out at any time.

3. The Committee requests further justification for the proposed use of opt-out consent. The Committee agrees that there needs to be a balance of the rigour of scientific design against the ethical implications of using this method. In this connection please supply details of the study that had been mentioned as an example involving opt-in consent.

The study referred to by Dr Hyde is the PREMFOOD trial (REC reference 12/LO/1391, approved by NRES Committee London, Fulham 10th December 2012; Clincaltrials.gov identifier NCT01686477). Briefly: this study is a similar comparative effectiveness trial, where children are recruited and randomised within 72 hours of birth to two different feeding regimens. Parents are approached by the researcher and informed of the study. This is recorded by the researcher placing a sticker in the baby's clinical notes to say the parents have been approached and informed about the study. The parents can opt out at any time, but no signed "opt-in" consent is obtained from them. Subsequent to the feeding study parents have the option of participating in an additional aspect of the trial that consists of an MRI scan to look at the impact of the feeding on infant body composition. This is an opt-in element and signed consent from the parents is obtained in the normal way.

4. The Committee requests further justification for not requiring a physical confirmation of the parents' understanding and consent decision regarding the study. This could take many forms, including an electronic signature or other method of recording the decision. The Committee has concerns that the absence of any form of acknowledgment of a consent decision from the parents lacks rigour, and does not feel that it is adequate for a researcher just to tick a box on an electronic database. In particular, consent normally involves a sequence of items listed so as to ensure adequate understanding of what is involved.

*WHEAT **will** require physical confirmation of the parents' understanding and consent decision regarding the study. The member of the clinical research team who has explained WHEAT to the parents will provide physical confirmation in the electronic health record. Access to the electronic health record is limited to members of the clinical team and requires a password, furthermore all data entered is both traceable and auditable; data entered cannot be permanently erased or altered).*

We have chosen opt-out consent in preference to a list of items on a consent form to make WHEAT as easy to understand as possible for parents. Evidence from neonatal research suggests that the use of a streamlined, opt-out consent process results in **greater** *understanding of the research study than opt-in consent (Rogers et al, Journal of Pediatrics 1998; attached).*

We would also like to draw the committee's attention to the minutes of the UK National Research Ethics Advisors' Panel (NREAP), 17th October 2012. The panel stated "there should be greater insistence on researchers showing how they have engaged with the relevant patient population to design and validate their information sheets" and that it is "important to distract RECs from a pre-conceived idea of what an information sheet should look like i.e. to move them away from an 'English teacher' approach to one where they focus on the ethical challenges of presenting the required information in order to gain meaningful consent". As you note in your letter, WHEAT has benefited from extensive parent and parent group involvement in relation to the design of the Patient Information Sheet; the opt-out design and the absence of a sequence of items listed on a consent form are the result of this parent involvement.

5. The Committee requests clarification of the process should a parent decide to opt-out of the study, and confirmation that no undue pressure or influence would be placed on them, even if unintentionally (for example, by asking them to sign a form to opt-out, when this is not required at the time of initial consent).

If a parent or carer chooses to opt out of WHEAT they will be able to do this by informing a member of the clinical or research team. They will not be required to sign a form to opt-out.

6. The Committee requests that the Protocol is amended to formally state as an exclusion criteria for the study, that any babies whose parents have not agreed to the inclusion of their data on the registry database for research purposes, will not be included.

The protocol has been amended to reflect this criterion (changes have been highlighted). Please note however that in the 2 years we have been obtaining permission for data to be included in the National Neonatal Research Database no parent in the UK has opted out.

The Committee requests that a statement is added to the PIS explaining that, due to participation in the study some babies may require an additional IV line that they might not otherwise require in routine clinical care.

Both of the treatment arms in WHEAT are in common use across the UK (Parige et al, ADC FN 2013, attached). Therefore both treatment options (including the additional IV line) are part of routine clinical care in the UK. While it is true that in neonatal units where feeds are

*not currently withheld around transfusion, and where a baby is randomised to have feeds withheld they may require an IV line that they would not have required **at that unit** had they not been in the trial, this IV line is **not** outside of routine clinical care in the UK. Conversely, in neonatal units where feeds are routinely withheld around transfusion, where a baby is randomised to feeding around transfusion, they will **avoid** an IV line that they would have otherwise required had they not been in the trial.*

Explaining both of these possible scenarios (requiring and additional IV line or avoiding an IV line dependent on the neonatal unit or clinician) will make the PIS confusing and the study more difficult to understand. For this reason and because both treatment arms are routine clinical care (and the IV line is therefore not a research procedure), we are reluctant to lengthen the Participant Information Sheet in this manner. This decision has been discussed extensively with, and ultimately shaped by, the parent member of the trial development group (a parent of 26 week gestation twins) and representatives of the national charity Bliss.

7. The Committee requests that the PIS is amended as follows:
   a) Please remove the final sentence of the section 'Are there any benefits for my baby?'. It is disingenuous to include this statement about "non-evidence based approach" in the PIS since evidence based care simply means care that is compatible with the current state of evidence.

The evidence for inclusion benefit in neonatal clinical trials is compelling, with some of the most conclusive and recent evidence coming from a large clinical trial that enrolled only babies (Carlo et al, NEJM 2012; attached). Our statement thus represents current scientific knowledge. We feel it is important that this important information is not withheld from parents. Providing this information ensures that they are truly fully informed.

We acknowledge the committee's point regarding the use of the term "evidence based" and have replaced the statement "This non-evidence based approach to neonatal care may involve more risk than being in a study like WHEAT which involves a carefully designed protocol and consistent monitoring" with "taking part in a research study may confer non-specific benefits" (changes highlighted in the Participant Information Sheet).

   b) In the same section, please amend the second sentence to read: "For babies not taking part in WHEAT, the decision of whether or not to stop feeds is made according to the usual clinical practice of your local medical team."

We have amended the PIS accordingly (changes highlighted).

   c) In the section 'Why has my baby been chosen?', please add the following sentence: "You have agreed for your baby's data to be used for research purposes in the national registry of premature babies (the National Neonatal Research Database)."

We do not agree with this suggested change. As we stated earlier, as far as we aware there have been no parents opting their baby out of the NNRD for the entire time of its existence. Consequently the need for this sentence is questionable, and it will only increase the length and unnecessary reading for the parent at a time when we are especially keen to reduce that burden. We agree that if the opt-out rate had been anything other than zero, it would have justified insertion of this statement.

We are happy to include this as an exclusion criteria in the protocol (as noted above) and to make it clear to members of the research team that this exclusion criteria exists, but we do not feel it necessary to labour this further with parents or to add it to the Participant Information Sheet.

   d) In the section 'Does my baby have to take part?', please start the section with "No." Please then move the second sentence to follow this one.

We have amended the Participant Information Sheet accordingly (changes highlighted).

e) In the section ‘What will happen to the results of WHEAT?’, please amend the first sentence to read: “We hope that the results may be used…”

We have amended the Participant Information Sheet accordingly (changes highlighted).

I hope these responses provide sufficient clarification, please do not hesitate to contact us if you require any further information.

Documents attached:

Document	Version	Date
Participant Information Sheet	1.4	14 October 2014
Protocol	1.4	14 October 2014
Rogers et al., Pediatrics		1998
Parige et al., ADC FN		2013
Carlo et al., NEJM		2012
NREAP minutes		17 October 2012

Yours Sincerely,

Dr Chris Gale
NIHR Clinical Lecturer in Paediatrics

NHS
Health Research Authority

27 October 2014

Dr Chris Gale
NIHR Clinical Lecturer
Imperial College London
Section of Academic Neonatal Medicine,
Imperial College London, Chelsea and Westminster Campus,
369 Fulham Road, London
SW10 9NH

Dear Dr Gale

**Study Title:**	**The WHEAT trial: WithHolding Enteral feeding Around packed red cell Transfusions in preterm neonates, a multicentre, superiority, randomised registry trial**
**REC reference number:**	
**Protocol number:**	
**IRAS project ID**	

Thank you for your letter of 15 October 2014, responding to the Committee's request for further information on the above research, and enclosing the following revised documents:

Document	*Version*	*Date*
IRAS Checklist XML [Checklist_16102014]		16 October 2014
Other [Supporting paper - Parige et al]	1	16 October 2013
Other [Response letter to REC]	1	15 October 2014
Other [Supporting paper - NREAP]		17 October 2012
Other [Amended Protocol]	1.4	14 October 2014
Other [Amended PIS]	1.4	14 October 2014
Other [Supporting paper - Rogers]	1	16 October 1998

The further information and revised documentation has been considered on behalf of the Committee by a Sub-Committee of the REC.

The Committee was satisfied with the responses to points 5, 6 and 7 of the Provisional Opinion Letter as follows:

Point 5 – the Committee accepts the reassurance that has been provided by the researchers.

Point 6 – the exclusion criteria in the Protocol have been amended as requested by the Committee. The request to add a statement to the PIS regarding the IV line has not been met, but the Committee accepts the argument for not doing so presented by the researchers.

Point 7b, d and e – these have been addressed as requested. The Committee accepts the argument presented by the researchers in response to point 7c. With regards to the

amended wording in response to point 7a, the Committee has no objection to the new wording.

However, the Committee would be grateful for a more complete response on the following points:

Points 1 and 2 – The Committee is not wholly satisfied with the answers provided. The Committee does accept that approaching parents regarding the study only at the point at which a blood transfusion is required will add unnecessary stress for the parents and give them little time to consider their involvement. However, the Committee still stands by the expert advice it received and that was confirmed during the telephone conversation with Dr Matthew Hyde, that it is unlikely that the babies will need a transfusion within the first 24–48 hours of their admission to NICU. The Committee fully accepts that parents are introduced to the trial “during their first few days in the neonatal unit” as stated in your response, but would like to see a clear separation between the obligatory initial “discussion about the neonatal unit and about research” (that could of course mention the WHEAT trial) and that of explaining the trial in more detail and electronically recording consent, whether it be on an opt-in or an opt-out basis. The Committee sees this separation as one that will give potential participants time to assess, on behalf of their vulnerable baby, the implications of taking part, including their full understanding of the fact that both arms involve standard care, and that they have a clear understanding of the consenting process.

Point 3 – The Committee notes the argument presented by way of reference to a precedent, namely that of the PREMFOOD trial. However, the Committee requests further justification and argument for the use of opt-out consent, as opposed to opt-in consent, in this particular trial. The Committee requests a clearly presented argument for both methods, including an indication of how this trial might be impacted, both positively and negatively, by the use of each method. The Committee does not necessarily object to the principle of opt-out consent, but the Committee wishes to see an argument that clearly weighs the benefits of this approach against the risks of the study (which include emotional risks for the parents of only later realising the significance of any failure to opt-out).

Point 4 – The Committee accepts that physical confirmation by way of a traceable, auditable electronic record is available to researchers, but that left the participant with no such confirmation of what had been agreed. While the Committee is pleased to see confirmation of the fact that prior to each blood transfusion “the local research team will confirm that parents are happy to continue participation”, it suggests that, in order to give parents a physical reminder that their baby is enrolled in a study and that they are free to withdraw at any time, parents should, whether opting-out or opting-in to the study, be presented with a card (like a medical alert card for clinical trials), reminding them that their baby is enrolled in the study, and who to contact if they have any queries or wish to withdraw at any point.

While drawing attention to NREAP minutes is quite appropriate and the selected quotations were of course accurate, the Committee would like to point out that the context was that of a discussion, not one of guidance. This discussion was followed by the HRA issuing draft guidance on proportionate consent for simple trials such as those involving a comparison of rival current treatments. This draft is still under discussion but in the case of individual participant randomisation it proposes opt-in consent.

Any further revised document submitted should be given a revised version number and date.

The 60 day clock for issue of a final ethical opinion on this application will re-start when the Committee has received a response on the outstanding points.

Please quote this number on all correspondence

Yours sincerely

Chair
Email:

Copy to: *Chelsea and Westminster NHS Foundation Trust*

Attendance at Sub-Committee of the REC meeting by correspondence

Committee Members:

Name	*Profession*	*Present*
	Mathematician (Retired)	Yes
	Consultant Psychiatrist	Yes
	Consultant Urologist	Yes

Imperial College London

Section of Neonatal Medicine
Imperial College London

4th Floor, Lift Bank D
Chelsea and Westminster Hospital
369 Fulham Road
London, SW10 9NH
Tel: +44 (0)203 3153519
Fax: +44 (0)203 3157998

christopher.gale@imperial.ac.uk

31st October 2014

Dr Christopher Gale MBBS MSc PhD MRCPCH

Dear

Study title: **The WHEAT trial: With Holding Enteral feeding Around packed red cell Transfusions in preterm neonates, a multicentre, superiority, randomised registry trial**

REC reference:
Protocol number:
IRAS project ID:

Thank you for your letter of 27 October 2014. Please find responses to your requests for further information detailed below:

Points 1 and 2: The Committee is not wholly satisfied with the answers provided. The Committee does accept that approaching parents regarding the study only at the point at which a blood transfusion is required will add unnecessary stress for the parents and give them little time to consider their involvement. However, the Committee still stands by the expert advice it received and that was confirmed during the telephone conversation with Dr Matthew Hyde, that it is unlikely that the babies will need a transfusion within the first 24–48 hours of their admission to NICU. The Committee fully accepts that parents are introduced to the trial "during their first few days in the neonatal unit" as stated in your response, but would like to see a clear separation between the obligatory initial "discussion about the neonatal unit and about research" (that could of course mention the WHEAT trial) and that of explaining the trial in more detail and electronically recording consent, whether it be on an opt-in or an opt-out basis. The Committee sees this separation as one that will give potential participants time to assess, on behalf of their vulnerable baby, the implications of taking part, including their full understanding of the fact that both arms involve standard care, and that they have a clear understanding of the consenting process.

> *While an "opt-in" study ultimately has to impose a timeframe in which a decision must be made, we are keen to move away from this using an "opt-out" approach. The 24-48 hour period stated in the WHEAT protocol has been chosen pragmatically to reflect the period when frequent discussions between the medical team and parents are likely (and therefore where a discussion about WHEAT could also occur). We see no reason that a discussion about WHEAT could not occur after 48 hours if deemed appropriate by the clinician, and could in fact occur at any time prior to the first blood transfusion. We elected to provide a time frame (24-48 hours) so that WHEAT is proposed to parents early enough to allow them plenty of time to consider participation prior to the first blood transfusion.*
>
> *We would be happy to extend the suggested time for discussion to 96 hours, but it is important to stress that we hope to develop an ongoing dialogue with parents about their involvement in WHEAT, where consent is confirmed prior to each blood transfusion (in accordance with the principles of Good Clinical Practice). We are keen to make it*

clear that the upper limit of this time frame is guidance rather than rigid time limit for participation. We have modified the protocol to reflect the suggested discussion time (highlighted).
We agree that a general "discussion about the neonatal unit and about research" should be separate from discussions about individual research studies (although we see no reason why these could not happen at the same time if appropriate). General discussions of this nature currently occur according to local unit practice (although the national charity Bliss is planning to produce more standardised literature).

Point 3 – The Committee notes the argument presented by way of reference to a precedent, namely that of the PREMFOOD trial. However, the Committee requests further justification and argument for the use of opt-out consent, as opposed to opt-in consent, in this particular trial. The Committee requests a clearly presented argument for both methods, including an indication of how this trial might be impacted, both positively and negatively, by the use of each method. The Committee does not necessarily object to the principle of opt-out consent, but the Committee wishes to see an argument that clearly weighs the benefits of this approach against the risks of the study (which include emotional risks for the parents of only later realising the significance of any failure to opt-out).

	**Positive impacts**	**Negative impacts**
Opt-out	Less biased sample (Junghans et al., BMJ 2005)	Emotional risks for the parents of only later realising the significance of any failure to opt-out
	More generalisable results	Litigation risks for clinicians if parents deny they provided consent for their baby to be involved in the study
	Greater participation leading to a shorter trial	Possibility that babies are enrolled into a trial without parents fully understanding or agreeing to participation
	More rapid resolution of clinical uncertainty	
	Lower cost	
	Greater understanding of the research study by parents (Rogers et al., Journal of Pediatrics 1998)	
	Development of a continuous dialogue about research with parents empowered to opt-out at any time	
	**Positive impacts**	**Negative impacts**
Opt-in	Signed consent form provides documentary evidence of parental consent (but not of parental understanding or voluntariness, Euricon Study Group., Lancet 2000)	Biased sample
		Less generalisable results
		Longer trial
		Longer period of clinical uncertainty
		Greater cost
		Less understanding of study by parents
		Impression of a "time-limited" consent process forcing a decision on parents.
		Possibility that babies are enrolled into a trial without parents fully understanding or agreeing to participation (Euricon Study Group.,

		Lancet 2000)
		Emotional risks for the parents of only later realising the significance of opting-in

We hope to reduce the negative risks of opt out consent as follows:
1. Emotional risks for the parents of only later realising the significance of any failure to opt-out:

- *We have clarified the Parent Information Sheet to clarify the opt-out nature of the consent process. We have added the following statement in large, bold type to the Parent Information Sheet "***The WHEAT study is an opt-out study. This means that all babies will take part unless you let a member of the neonatal team know that you do not wish your baby to participate."**
- *We will provide card to parents when their baby has been randomised as suggested in point 4 below.*

2. Litigation risks for clinicians if parents deny they provided consent for their baby to be involved in the study:

- *Using an electronic health record means that documentation that the WHEAT trial and the opt-out consent process have been explained to and understood by parents are mandatory prior to randomisation. The documentation will be permanent, traceable and fully auditable.*

3. Possibility that babies are enrolled into a trial without parents fully understanding or agreeing to participation:

- *Using an electronic health record means that documentation that the WHEAT trial and the opt-out consent process have been explained to and understood by parents are mandatory prior to randomisation.*
- *This risk exists in opt-in research studies as well: in the EURICON study (Lancet 2000) only 59 of 200 parents approached for informed consent using an opt-in process had given valid consent or refusal.*

Point 4 – The Committee accepts that physical confirmation by way of a traceable, auditable electronic record is available to researchers, but that left the participant with no such confirmation of what had been agreed. While the Committee is pleased to see confirmation of the fact that prior to each blood transfusion "the local research team will confirm that parents are happy to continue participation", it suggests that, in order to give parents a physical reminder that their baby is enrolled in a study and that they are free to withdraw at any time, parents should, whether opting-out or opting-in to the study, be presented with a card (like a medical alert card for clinical trials), reminding them that their baby is enrolled in the study, and who to contact if they have any queries or wish to withdraw at any point.

We agree that this is an important issue in relation to ensuring ongoing consent. We find the committee's suggestion of a physical reminder to be an elegant solution and have produced a card to be given to parents after WHEAT has been explained, where parents have not opted-out.

I hope these responses provide sufficient clarification, please do not hesitate to contact us if you require any further information.

Documents attached:

Document	Version	Date
Participant Information Sheet	1.5	31 October 2014
Protocol	1.5	31 October 2014
EURICON, 2000, Lancet		December 2000
WHEAT trial participant card	1.0	31 October 2014

Yours Sincerely,

Dr Chris Gale
NIHR Clinical Lecturer in Paediatrics

Health Research Authority

11 November 2014

Dr Chris Gale
NIHR Clinical Lecturer
Imperial College London
Section of Academic Neonatal Medicine,
Imperial College London, Chelsea and Westminster Campus,
369 Fulham Road, London
SW10 9NH

Dear Dr Gale

Study title: **The WHEAT trial: WithHolding Enteral feeding Around packed red cell Transfusions in preterm neonates, a multicentre, superiority, randomised registry trial**
REC reference:
Protocol number:
IRAS project ID:

Thank you for your letter of 07 November 2014, responding to the Committee's request for further information on the above research and submitting revised documentation.

The further information was considered in correspondence by a Sub-Committee of the REC. A list of the Sub-Committee members is attached.

We plan to publish your research summary wording for the above study on the HRA website, together with your contact details. Publication will be no earlier than three months from the date of this opinion letter. Should you wish to provide a substitute contact point, require further information, or wish to make a request to postpone publication, please contact the REC Manager.

Confirmation of ethical opinion

On behalf of the Committee, I am pleased to confirm a favourable ethical opinion for the above research on the basis described in the application form, protocol and supporting documentation as revised, subject to the conditions specified below.

Conditions of the favourable opinion

The favourable opinion is subject to the following conditions being met prior to the start of the study:

1. The initial general discussion about the neonatal unit and research should be clearly separated from the first more specific discussion about the WHEAT trial (as you have agreed should be the case) and not given at the same time (as we have consistently requested). This

condition is intended to ensure that potential participants have a breathing space allowing them to assimilate the implications of their clinical briefing before turning their minds to the nature of the WHEAT trial and gaining a proper understanding of the opt-out procedure. All local PIs should be made aware of this requirement.

2. On page 9 of the Protocol, the two sentences "Parents/carers who not opt out will be enrolled in WHEAT. Parents/carers can opt-out at any time" are corrected and extended to be replaced by "Parents/carers who do not opt out will have their baby enrolled in WHEAT. Parents/carers can opt-out at any time. They will be provided with a WHEAT Trial Participation Card indicating that their baby is enrolled in the study but that they are free to withdraw their baby at any time."

3. On the Trial Participation Card, the words "If you do not want your baby to be in" should be replaced by "If at any time you wish to withdraw your baby from".

You should notify the REC in writing once all conditions have been met (except for site approvals from host organisations) and provide copies of any revised documentation with updated version numbers. The REC will acknowledge receipt and provide a final list of the approved documentation for the study, which can be made available to host organisations to facilitate their permission for the study. Failure to provide the final versions to the REC may cause delay in obtaining permissions.

Management permission or approval must be obtained from each host organisation prior to the start of the study at the site concerned.

Management permission ("R&D approval") should be sought from all NHS organisations involved in the study in accordance with NHS research governance arrangements.

Guidance on applying for NHS permission for research is available in the Integrated Research Application System or at http://www.rdforum.nhs.uk.

Where a NHS organisation's role in the study is limited to identifying and referring potential participants to research sites ("participant identification centre"), guidance should be sought from the R&D office on the information it requires to give permission for this activity.

For non-NHS sites, site management permission should be obtained in accordance with the procedures of the relevant host organisation.

Sponsors are not required to notify the Committee of approvals from host organisations

Registration of Clinical Trials

All clinical trials (defined as the first four categories on the IRAS filter page) must be registered on a publically accessible database within 6 weeks of recruitment of the first participant (for medical device studies, within the timeline determined by the current registration and publication trees).

There is no requirement to separately notify the REC but you should do so at the earliest opportunity e.g. when submitting an amendment. We will audit the registration details as part of the annual progress reporting process.

To ensure transparency in research, we strongly recommend that all research is registered but for non-clinical trials this is not currently mandatory.

If a sponsor wishes to contest the need for registration they should contact , the HRA does not, however, expect exceptions to be made. Guidance on where to register is provided within IRAS.

It is the responsibility of the sponsor to ensure that all the conditions are complied with before the start of the study or its initiation at a particular site (as applicable).

Ethical review of research sites

NHS sites

The favourable opinion applies to all NHS sites taking part in the study, subject to management permission being obtained from the NHS/HSC R&D office prior to the start of the study (see "Conditions of the favourable opinion" below).

Approved documents

The final list of documents reviewed and approved by the Committee is as follows:

Document	*Version*	*Date*
Covering letter on headed paper		05 September 2014
Evidence of Sponsor insurance or indemnity (non NHS Sponsors only)		21 July 2014
IRAS Checklist XML [Checklist_07112014]		07 November 2014
Letter from sponsor		21 August 2014
Other [Supporting paper - Rogers]	1	16 October 1998
Other [Supporting paper - Parige et al]	1	16 October 2013
Other [Participant card]	1	31 October 2014
Other [Supporting paper - NREAP]		17 October 2012
Other [Project Protocol]	1.5	31 October 2014
Other [Response letter to REC]	1	31 October 2014
Other [Response letter to REC]	1	15 October 2014
Other [Patient Information Sheet]	1.5	31 October 2014
REC Application Form [REC_Form_10092014]		10 September 2014
Summary CV for Chief Investigator (CI)	1	05 September 2014

Statement of compliance

The Committee is constituted in accordance with the Governance Arrangements for Research Ethics Committees and complies fully with the Standard Operating Procedures for Research Ethics Committees in the UK.

After ethical review

Reporting requirements

The attached document *"After ethical review – guidance for researchers"* gives detailed guidance on reporting requirements for studies with a favourable opinion, including:

- Notifying substantial amendments
- Adding new sites and investigators
- Notification of serious breaches of the protocol
- Progress and safety reports
- Notifying the end of the study

The HRA website also provides guidance on these topics, which is updated in the light of changes in reporting requirements or procedures.

User Feedback

The Health Research Authority is continually striving to provide a high quality service to all applicants and sponsors. You are invited to give your view of the service you have received and the application procedure. If you wish to make your views known please use the feedback form available on the HRA website:
http://www.hra.nhs.uk/about-the-hra/governance/quality-assurance/

HRA Training

We are pleased to welcome researchers and R&D staff at our training days – see details at
http://www.hra.nhs.uk/hra-training/

**Please quote this number on all correspondence**

With the Committee's best wishes for the success of this project.

Yours sincerely

Chair
Email:

Enclosures: *List of names and professions of members who were present at the meeting*
"After ethical review – guidance for researchers" *[SL-AR2]*

Copy to:

Attendance at Sub-Committee of the REC meeting by correspondence

Committee Members:

Name	*Profession*	*Present*
	Consultant Urological Surgeon	Yes

	Mathematician (Retired)	Yes
	Consultant Psychiatrist	Yes

Imperial College London

Section of Neonatal Medicine
Imperial College London

4th Floor, Lift Bank D
Chelsea and Westminster Hospital
369 Fulham Road
London, SW10 9NH
Tel: +44 (0)203 3153519
Fax: +44 (0)203 3157998

christopher.gale@imperial.ac.uk

12th January 2015

Dr Christopher Gale MBBS MSc PhD MRCPCH

Dear,

Study title: **The WHEAT trial: With Holding Enteral feeding Around packed red cell Transfusions in preterm neonates, a multicentre, superiority, randomised registry trial**

REC reference:
Protocol number:
IRAS project ID:

Thank you for your letter of 11th November 2014. Please find responses to the conditions for favourable opinion below:

1. The initial general discussion about the neonatal unit and research should be clearly separated from the first more specific discussion about the WHEAT trial (as you have agreed should be the case) and not given at the same time (as we have consistently requested). This condition is intended to ensure that potential participants have a breathing space allowing them to assimilate the implications of their clinical briefing before turning their minds to the nature of the WHEAT trial and gaining a proper understanding of the opt-out procedure. All local PIs should be made aware of this requirement.
   - We have amended the protocol (page 9) to reflect this and will ensure that local PIs are made aware of this requirement.

2. On page 9 of the Protocol, the two sentences "Parents/carers who not opt out will be enrolled in WHEAT. Parents/carers can opt-out at any time" are corrected and extended to be replaced by "Parents/carers who do not opt out will have their baby enrolled in WHEAT. Parents/carers can opt-out at any time. They will be provided with a WHEAT Trial Participation Card indicating that their baby is enrolled in the study but that they are free to withdraw their baby at any time."
   - We have amended the protocol accordingly.

3. On the Trial Participation Card, the words "If you do not want your baby to be in" should be replaced by "If at any time you wish to withdraw your baby from".
   - We have amended the Trial Participation Card accordingly.

I hope these responses provide sufficient confirmation, please do not hesitate to contact us if you require any further information.

Documents attached:

Document	Version	Date
WHEAT trial - protocol	v1.6	120115

Yours Sincerely,

Dr Chris Gale
NIHR Clinical Lecturer in Paediatrics

16 January 2015

Dr Chris Gale
NIHR Clinical Lecturer
Imperial College London
Section of Academic Neonatal Medicine,
Imperial College London, Chelsea and Westminster Campus,
369 Fulham Road, London
SW10 9NH

Dear Dr Gale

**Study title:**	**The WHEAT trial: WithHolding Enteral feeding Around packed red cell Transfusions in preterm neonates, a multicentre, superiority, randomised registry trial**
**REC reference:**	
**Protocol number:**	
**IRAS project ID:**	

Thank you for your letter of 12 January 2015. I can confirm the REC has received the documents listed below and that these comply with the approval conditions detailed in our letter dated 12 November 2014

Documents received

The documents received were as follows:

Document	*Version*	*Date*
Other [Response Letter]	1	12 January 2015
Other [Amended Protocol]	1.6	12 January 2015
Other [Trial Participation Card]	1.1	12 January 2015

Approved documents

The final list of approved documentation for the study is therefore as follows:

Document	*Version*	*Date*
Covering letter on headed paper		05 September 2014
Evidence of Sponsor insurance or indemnity (non NHS Sponsors only)		21 July 2014
IRAS Checklist XML [Checklist_07112014]		07 November 2014
Letter from sponsor		21 August 2014
Other [Response letter to REC]	1	15 October 2014
Other [Supporting paper - NREAP]		17 October 2012
Other [Supporting paper - Parige et al]	1	16 October 2013
Other [Supporting paper - Rogers]	1	16 October 1998

Other [Response letter to REC]	1	31 October 2014
Other [Patient Information Sheet]	1.5	31 October 2014
Other [Response Letter]	1	12 January 2015
Other [Amended Protocol]	1.6	12 January 2015
Other [Trial Participation Card]	1.1	12 January 2015
REC Application Form [REC_Form_10092014]		10 September 2014
Summary CV for Chief Investigator (CI)	1	05 September 2014

You should ensure that the sponsor has a copy of the final documentation for the study. It is the sponsor's responsibility to ensure that the documentation is made available to R&D offices at all participating sites.

**Please quote this number on all correspondence**

Yours sincerely

REC Assistant

E-mail:

Copy to: *Chelsea and Westminster NHS Foundation Trust*
