# Supplementary material for: Research ethics committee decision-making in relation to an efficient neonatal trial
Source: Arch Dis Child Fetal Neonatal Ed. 2016 Sep 14;102(4):F291–8. doi: 10.1136/archdischild-2016-310935 (PMC5537508; doi:10.1136/archdischild-2016-310935)
Supplement: Supplementary data [file fetalneonatal-2016-310935supp008.pdf]

NHS
Health Research Authority

09 October 2014

Dr Chris Gale
NIHR Clinical Lecturer
Imperial College London
Section of Academic Neonatal Medicine
Imperial College London, Chelsea and Westminster Campus
369 Fulham Road
London
SW10 9NH

Dear Dr Gale

**Study Title:**	**The WHEAT trial: WithHolding Enteral feeding Around packed red cell Transfusions in preterm neonates, a multicentre, superiority, randomised registry trial**
**REC reference:**	
**Protocol number:**	
**IRAS project ID:**	

The Research Ethics Committee reviewed the above application at the meeting held on .

Provisional opinion

The Committee would be content to give a favourable ethical opinion of the research, subject to receiving a complete response to the request for further information set out below.

Authority to consider your response and to confirm the Committee's final opinion has been delegated to the Chair together with.

Further information or clarification required

1. Clarification regarding whether a centre would be able to participate in the trial if not all of the clinicians at that centre were happy to participate.

2. The following amendments to the Participant Information Sheet:
    a. Clarification at the beginning of the document in large, bold letters that in order not to participate in the research parents must opt out, not opt in.
    b. Removal of the information which states that there is an inclusion benefit to participants; this will not be the case as participants are babies.
    c. Clarification that a transfusion will be required for babies in the future at which point their treatment will be randomised and that this treatment will be the same for all future transfusions.
    d. Addition of information about other treatment options.

If you would find it helpful to discuss any of the matters raised above or seek further clarification from a member of the Committee, you are welcome to contact the REC Manager, .

When submitting a response to the Committee, the requested information should be electronically submitted from IRAS. A step-by-step guide on submitting your response to the REC provisional opinion is available on the HRA website using the following link: http://www.hra.nhs.uk/nhs-research-ethics-committee-rec-submitting-response-provisional-opinion/

Please submit revised documentation where appropriate underlining or otherwise highlighting the changes which have been made and giving revised version numbers and dates. You do not have to make any changes to the REC application form unless you have been specifically requested to do so by the REC.

The Committee will confirm the final ethical opinion within a maximum of 60 days from the date of initial receipt of the application, excluding the time taken by you to respond fully to the above points. A response should be submitted by no later than 08 November 2014.

Summary of the discussion at the meeting

The Chair welcomed Mr Matthew Hyde (Key Investigator) to the meeting and thanked him for attending by teleconference.

Mr Hyde was advised that an observer was present in the room; Mr Hyde consented to the observer staying.

Social or scientific value; scientific design and conduct of the study

It was agreed that this was a valuable and well-written study.

The REC was pleased with the way the inclusion of twins had been dealt with.

The Committee discussed whether it was necessary for parents to sign a document to state that they had received the information sheet and understood it, however, it was concluded that this was acceptable without an additional document because consent would not be taken as part of the opt-out process and that randomisation would not be performed without an electronic record to state that the parents had opted out of the trial.

Members considered whether opt-out data should also be collected to compare normal practices.

It was considered that it may be beneficial to randomise centres as opposed to babies, however the REC agreed that the current study design was acceptable and would be able to reach the required outcomes.

The Committee questioned whether participants knowing what the normal practice was in their centre would influence their decision about whether to participate. The REC considered that there could also be a risk of participants being treated differently, however, it was concluded that it was possible for every centre to do either intervention as part of standard care and this would therefore not be an issue.

It was considered that there could be longer term effects of both interventions which would not be studied as part of this research and that it would be beneficial to do so.

After discussion it was concluded that babies could take part in other trials as long as it did not affect their participation in either trial and therefore there would be no adverse effect on research carried out in half of the Paediatric Intensive Care Units in the country.

Members questioned whether the NNRD recorded a lot of outcomes and whether all of the outcomes required by the applicants would be available from this source.

Mr Hyde confirmed that that would be the case and that this information would include the PN start and stop times. The information would be that which was clinically entered and it was known that the quality of the data which was entered varied by outcome. The outcomes you had chosen to study were well-recorded and you could therefore guarantee that this information was robust. He added that other data could be used for secondary outcomes of the research but that if it was used it would be with the understanding that the data would not be as robust as that used for the primary outcome.

It was queried whether this data would be received automatically.

Mr Hyde clarified that you would be able to access only the data you required by pulling it out of the system.

The Committee considered whether clinicians should or would be able to opt-out of participating at any centre, for example, if a child had been randomised to receive a treatment different to that which they believed would be more beneficial.

Mr Hyde responded that clinicians would be able to opt-out if they chose but added that there would have to be equipoise within the units. Most units around the country were willing to join the research. If there was no equipoise then the unit and the baby would not be included in the research. If a clinician withdrew a participant from the research this would be recorded on the database.

Members accepted this clarification but questioned how you would ensure that centres would be able to opt-out if required. It was questioned whether centres should take part if not all of the clinicians were amenable to participating.

Mr Hyde replied that within the unit the policy would have to be to participate in the research or not so that there was equipoise. If one particular clinician in a centre did not want to take part in the research as a whole this would be a different matter, and he requested you responded to this point in correspondence. He commented that the decision on how to treat patients was often based upon cost and not necessarily which treatment was better, as this was not known.

<u>Informed consent process and the adequacy and completeness of participant information</u>

The REC discussed whether an opt-out consent was acceptable for parents of participants, and it was concluded that it was. However, the REC agreed that it must be clear within the Participant Information Sheet at the very beginning of the document that parents must opt-out of the research, not opt-in.

Mr Hyde agreed that this could be amended.

Mr Hyde was informed that there would be further items which required amendment in the study documentation and that these would be dealt with in correspondence.

Mr Hyde left the teleconference.

Other ethical issues were raised and resolved in preliminary discussion before Mr Hyde's attendance at the meeting.
Documents reviewed

The documents reviewed at the meeting were:

Document	Version	Date
Covering letter on headed paper		05 September 2014
Evidence of Sponsor insurance or indemnity (non NHS Sponsors only)		05 September 2014
Letter from sponsor		05 September 2014
Participant information sheet (PIS)	1.3	02 September 2014
REC Application Form [REC_Form_10092014]		10 September 2014
Referee's report or other scientific critique report	Email from Matthew Hyde	16 September 2014
Research protocol or project proposal	1.3	11 August 2014
Summary CV for Chief Investigator (CI)	1	05 September 2014

Membership of the Committee

The members of the Committee who were present at the meeting are listed on the attached sheet

Statement of compliance

The Committee is constituted in accordance with the Governance Arrangements for Research Ethics Committees and complies fully with the Standard Operating Procedures for Research Ethics Committees in the UK.

**Please quote this number on all correspondence**

Yours sincerely

Chair

Email:

Enclosures: *List of names and professions of members who were present at the meeting and those who submitted written comments.*

Copy to: *Imperial College London*
Chelsea and Westminster NHS Foundation Trust

Attendance at Committee meeting

Committee Members:

Name	*Profession*	*Present*	*Notes*
	Clinical Studies Officer	No	
	Senior Research Ethics Administrator	Yes	
	Retired Multimedia Project Manager	Yes	
	Retired Professor of Visualization	Yes	
	ACF ST in General Adult Psychiatry	Yes	
	Senior Lecturer in Nursing	Yes	
	Consultant Neurologist	Yes	
	Retired Special Needs Coordinator	Yes	
	Emeritus Professor of Nursing Research	No	
	Consultant Oncologist	No	
	Speciality Trainee in Anaesthesia	Yes	
	Foundation Year 2 Doctor	No	
	Consultant Paediatric Surgeon	Yes	
	Speciality Training Registrar - Surgery	Yes	
	Consultant ENT Surgeon	Yes	
	Assistant Chief Pharmacist	No	

Also in attendance:

Name	*Position (or reason for attending)*
	Research Sister
	REC Manager

Imperial College London

Section of Neonatal Medicine
Imperial College London

4th Floor, Lift Bank D
Chelsea and Westminster Hospital
369 Fulham Road
London, SW10 9NH
Tel: +44 (0)203 3153519
Fax: +44 (0)203 3157998

christopher.gale@imperial.ac.uk

15th October 2014

Dr Christopher Gale MBBS MSc PhD MRCPCH

Dear,

Study title: **The WHEAT trial: With Holding Enteral feeding Around packed red cell Transfusions in preterm neonates, a multicentre, superiority, randomised registry trial**

REC reference:
Protocol number:
IRAS project ID:

Thank you for taking the time to review the WHEAT trial. Please find responses to your requests for further information detailed below:

1. Clarification regarding whether a centre would be able to participate in the trial if not all of the clinicians at that centre were happy to participate:

We see no reason why a centre cannot participate if some, but not all, clinicians at that centre were in equipoise and willing to randomise babies into WHEAT. Please note though that normal practice in neonatal units is for participation in a research study to be determined by the whole team.

Also please note that the key issue is whether clinicians who were not happy to participate in WHEAT would be happy to deliver the allocated intervention to infants already enrolled and randomised to one or other arm. If this were NOT the case then the centre would not be able to participate, as delivery of the trial protocol would be compromised.

2. The following amendments to the Participant Information Sheet:
   a. Clarification at the beginning of the document in large, bold letters that in order not to participate in the research parents must opt out, not opt in.

*We have amended the Participant Information Sheet as indicated (highlighted), by inserting the following in bold at the head of the sheet: "***The WHEAT study is an opt-out study. This means that all babies will take part unless you let a member of the neonatal team know that you do not wish your baby to participate.***"*

   b. Removal of the information which states that there is an inclusion benefit to participants; this will not be the case as participants are babies.

We are unclear as to why the committee feels that there will not be an inclusion benefit because participants are babies. The reason for inclusion benefit for clinical trial participants is uncertain though a plausible explanation is that this derives from the closely monitored, protocol driven care within a trial. Some of the most conclusive and recent evidence of

inclusion benefit comes from a large clinical trial that enrolled only babies (Carlo et al, NEJM 2013; attached). Inclusion benefit is highly likely in neonatal trials. In the Participant Information Sheet we endeavour to provide complete information to parents.

c. Clarification that a transfusion will be required for babies in the future at which point their treatment will be randomised and that this treatment will be the same for all future transfusions.

*We have clarified that transfusion will be required for almost all babies (greater than 90%) in the second section of the Participant Information Sheet (highlighted). In this we already state in section 6 that the allocated treatment will be the same for all future transfusions: "*If your baby is randomised to have feeds stopped this will be for 4 hours before, after, and during this and any subsequent blood transfusions*." We feel that this is sufficient and therefore have not modified this passage further.*

d. Addition of information about other treatment options.

We are unsure as to what other treatment options the committee refers to. The two management options in standard use (continued feeding or withholding feeds; there are no other options that we are aware of; Parige et al, ADC FN 2013; attached) are both represented in WHEAT. WHEAT is investigating the optimal feeding practice during blood transfusion after a clinical decision has been made that blood transfusion is indicated. WHEAT is ***not*** *examining different treatment options for anaemia of prematurity. We have accurately described the alternative to the study in the section "*What will happen if I opt out?*" which reads as follows: "*If you "opt-out" your baby will still have feeds either stopped or continued during transfusions in the same way as in WHEAT but the decision will be made by the local clinical team and the policy of the neonatal unit, and information about your baby will not be included in the study.*"*

I hope these responses provide sufficient clarification, please do not hesitate to contact us if you require any further information.

Documents attached:

Document	Version	Date
Participant Information Sheet	1.4	15 October 2014
Carlo et al., NEJM		2013
Parige et al., ADC FN		2013

Yours Sincerely,

Dr Chris Gale
NIHR Clinical Lecturer in Paediatrics

20 October 2014

Dr Chris Gale
NIHR Clinical Lecturer
Imperial College London
Section of Academic Neonatal Medicine
Imperial College London, Chelsea and Westminster Campus
369 Fulham Road
London
SW10 9NH

Dear Dr Gale

**Study title:**	**The WHEAT trial: WithHolding Enteral feeding Around packed red cell Transfusions in preterm neonates, a multicentre, superiority, randomised registry trial**
**REC reference:**	
**Protocol number:**	
**IRAS project ID:**	

Thank you for your letter of 15 October 2014, responding to the Committee's request for further information on the above research and submitting revised documentation.

The further information has been considered on behalf of the Committee by the Chair together with.

We plan to publish your research summary wording for the above study on the HRA website, together with your contact details. Publication will be no earlier than three months from the date of this opinion letter. Should you wish to provide a substitute contact point, require further information, or wish to make a request to postpone publication, please contact the REC Manager.

Confirmation of ethical opinion

On behalf of the Committee, I am pleased to confirm a favourable ethical opinion for the above research on the basis described in the application form, protocol and supporting documentation as revised, subject to the conditions specified below.

Conditions of the favourable opinion

The favourable opinion is subject to the following conditions being met prior to the start of the study.

1. Removal of the following sentence in the Participant Information Sheet: "This non-evidence based approach to neonatal care may involve more risk than being in a study like WHEAT which involves a carefully designed protocol and consistent monitoring."

You should notify the REC in writing once all conditions have been met (except for site approvals from host organisations) and provide copies of any revised documentation with updated version numbers. The REC will acknowledge receipt and provide a final list of the approved documentation for the study, which can be made available to host organisations to facilitate their permission for the study. Failure to provide the final versions to the REC may cause delay in obtaining permissions.

Management permission or approval must be obtained from each host organisation prior to the start of the study at the site concerned.

Management permission ("R&D approval") should be sought from all NHS organisations involved in the study in accordance with NHS research governance arrangements.

Guidance on applying for NHS permission for research is available in the Integrated Research Application System or at http://www.rdforum.nhs.uk.

Where a NHS organisation's role in the study is limited to identifying and referring potential participants to research sites ("participant identification centre"), guidance should be sought from the R&D office on the information it requires to give permission for this activity.

For non-NHS sites, site management permission should be obtained in accordance with the procedures of the relevant host organisation.

Sponsors are not required to notify the Committee of approvals from host organisations

Registration of Clinical Trials

All clinical trials (defined as the first four categories on the IRAS filter page) must be registered on a publicly accessible database within 6 weeks of recruitment of the first participant (for medical device studies, within the timeline determined by the current registration and publication trees).

There is no requirement to separately notify the REC but you should do so at the earliest opportunity e.g. when submitting an amendment. We will audit the registration details as part of the annual progress reporting process.

To ensure transparency in research, we strongly recommend that all research is registered but for non-clinical trials this is not currently mandatory.

If a sponsor wishes to contest the need for registration they should contact, the HRA does not, however, expect exceptions to be made. Guidance on where to register is provided within IRAS.

It is the responsibility of the sponsor to ensure that all the conditions are complied with before the start of the study or its initiation at a particular site (as applicable).

Ethical review of research sites

NHS sites

The favourable opinion applies to all NHS sites taking part in the study, subject to management permission being obtained from the NHS/HSC R&D office prior to the start of the study (see "Conditions of the favourable opinion" below).

Approved documents

The final list of documents reviewed and approved by the Committee is as follows:

Document	*Version*	*Date*
Covering letter on headed paper		05 September 2014
Evidence of Sponsor insurance or indemnity (non NHS Sponsors only)		05 September 2014
Letter from sponsor		05 September 2014
Other [Parige et al paper]		
Other [Participant Information Sheet]	1.4	14 October 2014
Other [Carlo et al paper]		
Other [Response to Provisional Opinion]		15 October 2014
REC Application Form [REC_Form_10092014]		10 September 2014
Referee's report or other scientific critique report	Email from Matthew Hyde	16 September 2014
Research protocol or project proposal	1.3	11 August 2014
Summary CV for Chief Investigator (CI)	1	05 September 2014

Statement of compliance

The Committee is constituted in accordance with the Governance Arrangements for Research Ethics Committees and complies fully with the Standard Operating Procedures for Research Ethics Committees in the UK.

After ethical review

Reporting requirements

The attached document *"After ethical review – guidance for researchers"* gives detailed guidance on reporting requirements for studies with a favourable opinion, including:

- Notifying substantial amendments
- Adding new sites and investigators
- Notification of serious breaches of the protocol
- Progress and safety reports
- Notifying the end of the study

The HRA website also provides guidance on these topics, which is updated in the light of changes in reporting requirements or procedures.

User Feedback

The Health Research Authority is continually striving to provide a high quality service to all applicants and sponsors. You are invited to give your view of the service you have received and the application procedure. If you wish to make your views known please use the feedback form available on the HRA website: http://www.hra.nhs.uk/about-the-hra/governance/quality-assurance/

HRA Training

We are pleased to welcome researchers and R&D staff at our training days – see details at http://www.hra.nhs.uk/hra-training/

**Please quote this number on all correspondence**

With the Committee's best wishes for the success of this project.

Yours sincerely

Chair

Email:

Enclosures: "After ethical review – guidance for researchers" *SL-AR2*

Copy to: *Imperial College London*

Chelsea and Westminster NHS Foundation Trust

Imperial College London

Imperial College London

Section of Neonatal Medicine
Imperial College London

4th Floor, Lift Bank D
Chelsea and Westminster Hospital
369 Fulham Road
London, SW10 9NH
Tel: +44 (0)203 3153519
Fax: +44 (0)203 3157998

christopher.gale@imperial.ac.uk

31st October 2014

Dr Christopher Gale MBBS MSc PhD MRCPCH

Dear,

Study title: **The WHEAT trial: With Holding Enteral feeding Around packed red cell Transfusions in preterm neonates, a multicentre, superiority, randomised registry trial**

REC reference:
Protocol number:
IRAS project ID:

Thank you for the letter confirming your favourable opinion on the WHEAT trial. We note that this is dependent on the following condition:

1. Removal of the following sentence in the Participant Information Sheet: "This non-evidence based approach to neonatal care may involve more risk than being in a study like WHEAT which involves a carefully designed protocol and consistent monitoring."

We feel it is important that parents are fully informed about the potential benefits as well as risks of participating in research. Given the compelling evidence for inclusion benefit in neonatal trials we are not willing to remove this completely from the Participant Information Sheet. Rather than removing this statement, would the committee agree to it with "taking part in a research study may confer non-specific benefits"?

We have made this change on the enclosed Participant Information Sheet (highlighted).

We feel strongly that parents cannot make an informed decision about a study without knowing the potential benefits of involvement as well as the potential risks. Consequently we feel that potential benefits should be included on the patient information sheet in the same way that potential risks would be. We would encourage the committee to watch the following video clip by the renowned ethicist and Professor of Paediatric Bioethics John Lantos. (https://www.youtube.com/watch?v=SmWJnOp1QaU). It explains our rationale for this request.

I hope this change may be agreeable. Please do not hesitate to contact us if you require any further information.

Documents attached:

Document	Version	Date
Participant Information Sheet	1.5	14 October 2014

.

Yours Sincerely,

Dr Chris Gale
NIHR Clinical Lecturer in Paediatrics

Imperial College London

Section of Neonatal Medicine
Imperial College London

4th Floor, Lift Bank D
Chelsea and Westminster Hospital
369 Fulham Road
London, SW10 9NH
Tel: +44 (0)203 3153519
Fax: +44 (0)203 3157998

christopher.gale@imperial.ac.uk

12th January 2015

Dr Christopher Gale MBBS MSc PhD MRCPCH

Dear,

Study title: **The WHEAT trial: With Holding Enteral feeding Around packed red cell Transfusions in preterm neonates, a multicentre, superiority, randomised registry trial**

REC reference:
Protocol number:
IRAS project ID:

Thank you for the letter confirming your favourable opinion on the WHEAT trial. Please find attached our response to the specified condition.

1. Removal of the following sentence in the Participant Information Sheet: “This non-evidence based approach to neonatal care may involve more risk than being in a study like WHEAT which involves a carefully designed protocol and consistent monitoring.”
    - We have modified the Participant Information Sheet accordingly.

I hope this change may be agreeable. Please do not hesitate to contact us if you require any further information.

Documents attached:

Document	Version	Date
Participant Information Sheet	1.5	12 January 2015

Yours Sincerely,

Dr Chris Gale
NIHR Clinical Lecturer in Paediatrics

NHS
Health Research Authority

20 January 2015

Dr Chris Gale
NIHR Clinical Lecturer
Imperial College London
Section of Academic Neonatal Medicine,
Imperial College London, Chelsea and Westminster Campus,
369 Fulham Road
London
SW10 9NH

Dear Dr Gale

Study title: **The WHEAT trial: WithHolding Enteral feeding Around packed red cell Transfusions in preterm neonates, a multicentre, superiority, randomised registry trial**
REC reference:
Protocol number:
IRAS project ID:

Thank you for your letter of 19th January 2014. I can confirm the REC has received the documents listed below and that these comply with the approval conditions detailed in our letter dated 20 October 2014

Documents received

The documents received were as follows:

Document	*Version*	*Date*
Participant information sheet (PIS)	1.6	18 January 2015

Approved documents

The final list of approved documentation for the study is therefore as follows:

Document	*Version*	*Date*
Covering letter on headed paper		05 September 2014
Evidence of Sponsor insurance or indemnity (non NHS Sponsors only)		05 September 2014

Letter from sponsor		05 September 2014
Other [Response to Provisional Opinion]		15 October 2014
Other [Carlo et al paper]		
Other [Parige et al paper]		
Participant information sheet (PIS)	1.6	18 January 2015
REC Application Form [REC_Form_10092014]		10 September 2014
Referee's report or other scientific critique report	Email from Matthew Hyde	16 September 2014
Research protocol or project proposal	1.3	11 August 2014
Summary CV for Chief Investigator (CI)	1	05 September 2014

You should ensure that the sponsor has a copy of the final documentation for the study. It is the sponsor's responsibility to ensure that the documentation is made available to R&D offices at all participating sites.

**Please quote this number on all correspondence**

Yours sincerely

Research Ethics Committee Assistant

E-mail:

Copy to: *Imperial College London*

Chelsea and Westminster NHS Foundation Trust

20 October 2014
(reissued 20 January 2015)

Dr Chris Gale
NIHR Clinical Lecturer
Imperial College London
Section of Academic Neonatal Medicine
Imperial College London, Chelsea and Westminster Campus
369 Fulham Road
London
SW10 9NH

Dear Dr Gale

**Study title:**	**The WHEAT trial: WithHolding Enteral feeding Around packed red cell Transfusions in preterm neonates, a multicentre, superiority, randomised registry trial**
**REC reference:**	
**Protocol number:**	
**IRAS project ID:**	

Thank you for your letter of 15 October 2014, responding to the Committee's request for further information on the above research and submitting revised documentation.

The further information has been considered on behalf of the Committee by the Chair together with.

We plan to publish your research summary wording for the above study on the HRA website, together with your contact details. Publication will be no earlier than three months from the date of this opinion letter. Should you wish to provide a substitute contact point, require further information, or wish to make a request to postpone publication, please contact the REC Manager.

Confirmation of ethical opinion

On behalf of the Committee, I am pleased to confirm a favourable ethical opinion for the above research on the basis described in the application form, protocol and supporting documentation as revised, subject to the conditions specified below.

Conditions of the favourable opinion

The favourable opinion is subject to the following conditions being met prior to the start of the study.

1. Addition of the following sentence to the Participant Information Sheet: "Research suggests that taking part in a study like WHEAT may confer non-specific benefits, this might be because babies in these studies are looked after according to carefully designed protocols and receive consistent monitoring".

You should notify the REC in writing once all conditions have been met (except for site approvals from host organisations) and provide copies of any revised documentation with updated version numbers. The REC will acknowledge receipt and provide a final list of the approved documentation for the study, which can be made available to host organisations to facilitate their permission for the study. Failure to provide the final versions to the REC may cause delay in obtaining permissions.

Management permission or approval must be obtained from each host organisation prior to the start of the study at the site concerned.

Management permission ("R&D approval") should be sought from all NHS organisations involved in the study in accordance with NHS research governance arrangements.

Guidance on applying for NHS permission for research is available in the Integrated Research Application System or at http://www.rdforum.nhs.uk.

Where a NHS organisation's role in the study is limited to identifying and referring potential participants to research sites ("participant identification centre"), guidance should be sought from the R&D office on the information it requires to give permission for this activity.

For non-NHS sites, site management permission should be obtained in accordance with the procedures of the relevant host organisation.

Sponsors are not required to notify the Committee of approvals from host organisations

Registration of Clinical Trials

All clinical trials (defined as the first four categories on the IRAS filter page) must be registered on a publicly accessible database within 6 weeks of recruitment of the first participant (for medical device studies, within the timeline determined by the current registration and publication trees).

There is no requirement to separately notify the REC but you should do so at the earliest opportunity e.g. when submitting an amendment. We will audit the registration details as part of the annual progress reporting process.

To ensure transparency in research, we strongly recommend that all research is registered but for non-clinical trials this is not currently mandatory.

If a sponsor wishes to contest the need for registration they should contact , the HRA does not, however, expect exceptions to be made. Guidance on where to register is provided within IRAS.

It is the responsibility of the sponsor to ensure that all the conditions are complied with before the start of the study or its initiation at a particular site (as applicable).

Ethical review of research sites

NHS sites

The favourable opinion applies to all NHS sites taking part in the study, subject to management permission being obtained from the NHS/HSC R&D office prior to the start of the

study (see "Conditions of the favourable opinion" below).

Approved documents

The final list of documents reviewed and approved by the Committee is as follows:

Document	*Version*	*Date*	
Covering letter on headed paper		05 September 2014	
Evidence of Sponsor insurance or indemnity (non NHS Sponsors only)		05 September 2014	
Letter from sponsor		05 September 2014	
Other [Parige et al paper]			
Other [Participant Information Sheet]	1.4	14 October 2014	
Other [Carlo et al paper]			
Other [Response to Provisional Opinion]		15 October 2014	
REC Application Form [REC_Form_10092014]		10 September 2014	
Referee's report or other scientific critique report	Email from Matthew Hyde	16 September 2014	
Research protocol or project proposal	1.3	11 August 2014	
Summary CV for Chief Investigator (CI)	1	05 September 2014	

Statement of compliance

The Committee is constituted in accordance with the Governance Arrangements for Research Ethics Committees and complies fully with the Standard Operating Procedures for Research Ethics Committees in the UK.

After ethical review

Reporting requirements

The attached document *"After ethical review – guidance for researchers"* gives detailed guidance on reporting requirements for studies with a favourable opinion, including:

- Notifying substantial amendments
- Adding new sites and investigators
- Notification of serious breaches of the protocol
- Progress and safety reports
- Notifying the end of the study

The HRA website also provides guidance on these topics, which is updated in the light of changes in reporting requirements or procedures.

User Feedback

The Health Research Authority is continually striving to provide a high quality service to all applicants and sponsors. You are invited to give your view of the service you have received and the application procedure. If you wish to make your views known please use the feedback form available on the HRA website: http://www.hra.nhs.uk/about-the-hra/governance/quality-assurance/

HRA Training

We are pleased to welcome researchers and R&D staff at our training days – see details at http://www.hra.nhs.uk/hra-training/

Letter reissued on 20th January 2015 to vary the opinion (condition of favourable opinion amended)

**Please quote this number on all correspondence**

With the Committee's best wishes for the success of this project.

Yours sincerely

pp

Chair

Email:

Enclosures: "After ethical review – guidance for researchers" *SL-AR2*

Copy to: *Imperial College London*

Chelsea and Westminster NHS Foundation Trust

Imperial College London
