# Supplementary material for: Research ethics committee decision-making in relation to an efficient neonatal trial
Source: Arch Dis Child Fetal Neonatal Ed. 2016 Sep 14;102(4):F291–8. doi: 10.1136/archdischild-2016-310935 (PMC5537508; doi:10.1136/archdischild-2016-310935)
Supplement: Supplementary data [file fetalneonatal-2016-310935supp010.pdf]

09 October 2014

Dr Chris Gale
NIHR Clinical Lecturer
Imperial College London
Section of Academic Neonatal Medicine,
Chelsea and Westminster Campus,
369 Fulham Road, London
SW10 9NH

Dear Dr Gale

Study title: **The WHEAT trial: With Holding Enteral feeding Around packed red cell Transfusions in preterm neonates, a multicentre, superiority, randomised registry trial**
REC reference:
Protocol number:
IRAS project ID:

The Research Ethics Committee reviewed the above application at the meeting held on.

We plan to publish your research summary wording for the above study on the HRA website, together with your contact details, unless you expressly withhold permission to do so. Publication will be no earlier than three months from the date of this opinion letter. Should you wish to provide a substitute contact point, require further information, or wish to make a request to postpone publication, please contact the REC Manager .

Ethical opinion

The members of the Committee present decided to issue an unfavourable opinion for the following reasons:

1. The Committee did not accept that it was appropriate for patients to be entered into this study without prior consent from parents. The 'opt-out' consent is not a concept that the Committee recognises; it is recruitment without consent. This raised many ethical issues which have not been addressed. The Committee also considered that written evidence of consent would protect researchers from future action by parents or authorities in the event of adverse outcomes, which are not rare in this very vulnerable patient population.
2. The Committee had concerns how staff and units will manage those participants randomised to the arm which is not usual care for that site and the additional risks this might pose.

3. The Committee were unsure if researchers would be contacting parents in the future for consent to assess their child's medical notes. If they obtained consent at the beginning of the study they would not have to contact parents later thus raising further ethical issues, especially if outcome is poor.
4. The Committee thought the Parent Information Sheet was coercive in places, stating it was better to be in the study than not.
5. The Committee noted that the Parent Information Sheet did not cover that there may be extra risks taking part in the study, as some child may receive additional interventions which they would not receive if they were not part of the study.
6. The Committee were unsure why parents were being informed of the results before peer-review publications.
7. The Committee would like more clarification around the 1.5% absolute risk reduction which may be addressed in the independent scientific review.

I regret to inform you therefore that the application is not approved.

If you would find it helpful to discuss any of the matters raised above or seek further clarification from a member of the Committee, you are welcome to contact

Options for further ethical review

You may submit a new application for ethical review, taking into account the Committee's concerns. You should enter details of this application on the application form and include a copy of this letter, together with a covering letter explaining what changes have been made from the previous application. We strongly recommend that you submit the new application to this REC. However, you may submit the application to a different REC if you prefer.

Alternatively, you may appeal against the decision of the Committee by seeking a second opinion on this application from another Research Ethics Committee. The appeal would be based on the application form and supporting documentation reviewed by this Committee, without amendment. If you wish to appeal, you should notify the relevant Research Ethics Service manager (see below) in writing within 90 days of the date of this letter. If the appeal is allowed, another REC will be appointed to give a second opinion within 60 days and the second REC will be provided with a copy of the application, together with this letter and other relevant correspondence on the application. You will be notified of the arrangements for the meeting of the second REC and will be able to attend and/or make written representations if you wish to do so.

The contact point for appeals is:

HRA Improvement & Liaison Manager
National Research Ethics Service

Email:

Summary of discussion at the meeting

Ethical issues raised by the Committee in private discussion, together with responses given by the researcher when invited into the meeting

The Chair phoned Dr Matthew Hyde and thanked him for being available to discuss the study.

Social or scientific value; scientific design and conduct of the study

The Committee thought the study had a good argument for scientific and social value. Previous studies have been inconclusive due to small numbers. The Committee noted that previous studies have looked in retrospect at medical notes.

The Committee noted that A59 assumes 50% of units joining the study, the protocol p54 (15) indicated 75%

The Committee were unsure around the percentage reductions. A60 p28 last paragraph: 4% assumed baseline incidence – 1.5 % incidence reduction is not the same as an "absolute risk reduction of 1.5%"

The Committee questioned why siblings in multiple births were not being randomised separately, especially as multiple births make up a large proportion of preterm neonates. Dr Hyde explained that any multi births will be randomised into the same group. They have done this as they received feedback from PPI groups that parents would be happier if multi birth babies received the same treatment. Dr Hyde explained that they received strong feedback from two parents with twins that they would want both children to receive the same treatment.

Favourable risk benefit ratio; anticipated benefit/risks for research participants (present and future)

The Committee queried if some babies will require an additional cannula above the usual care for their hospital and whether this could lead to additional risks which haven't been covered in the risks on IRAS or mentioned to parents in the PIS. Dr Hyde explained that some babies may have to have an extra cannula due to being on the study but this would be a very low percentage, less than half.

The Committee queried if there would be an increased risk for babies who are on the arm of the study which is not standard care at that hospital. Will staff have the appropriate training and equipment to offer the same standard of care? Dr Hyde explained that this was not his area and that Dr Gale may be more informed to answer this question. However there may be a slight increased risk for babies who are not on the standard of treatment arm but both treatments are used widely in UK hospitals and could not see that the risk would be high.

Informed consent process and the adequacy and completeness of participant information

The Committee discussed whether the opt-out process was appropriate for this type of study and if it was being delivered effectively. The Committee noted that this study needs large amount of participants to achieve good results and that the opt-out process would help with achieving high recruitment rates. However this is not an emergency situation and time can be taken to ensure parents are properly informed and consented. The Committee thought the opt-out process could be coercive and that parents will be highly stressed at that time and should be not have the extra burden of having to opt-out of a study.

The Committee queried how the researchers will record that parents have discussed the study with clinicians and have been given the chance to op-out.

The Committee asked why they were asking parents to opt-out rather than take consent. Dr Hyde explained that opting out was an ongoing process in which they will approach the parents 24hrs after birth and then again if their baby is having a blood transfusion they will be approached to make sure they are aware of the study and the opt-out process. Dr Hyde explained that if parents chose to opt-out this will be recorded on electronic database. Dr Hyde explained the database will flag up any babies which are suitable for the study to the clinician. The clinician will then go through the information sheet explaining the study to the parents and the opt-out process. If the parents do not want to opt-out the clinician will enter this into the database that the parents have not opted out and then the database will randomise the baby

into an arm. If the parents do opt-out again this will be entered into the database and will remove the child's records from the study. If the parents later choose to opt-out again this will be entered in to database and the child's records will be removed from the study.

Dr Hyde explained that if this was a new intervention then they would definitely go down the consenting process rather than the opting out process. However both treatments are being used in hospitals around the country, babies would receive either treatment it would just depend on where the babies are born. Dr Hyde explained that similar studies have been approved with the opting out recruitment which are running on the neonatal unit. Dr Hyde explained that the opting out process would remove the burden from parents. The Committee queried that the opting out process sounds very like a consenting process and are still unsure why parents can not be consented. The Committee said they accept this is an important study and that large numbers are needed and opting out would maximise recruitment. But researchers need to be make sure parents are aware of the study and what it involves and the risks associated with it. Having a consent form demonstrated that parents have accepted all these things and that they agreed to include their baby in the study. The Committee did not see that this was more of a burden on parents. The Committee felt that also the consent form covers the researchers in the case of an adverse outcome. The researchers will have a signed form stating that the parents agreed to be part of the study. The Committee noted that there have been previous studies in this age group where parents have denied giving consent, but a signed consent form had provided evidence to the contrary. Dr Hyde explained that consent form can go missing and that with a opting out system this would not be the case.

The Committee asked if the electronic database had been developed. Dr Hyde explained that it is being design at the moment. The Committee asked if the database could be design to record opting in. Dr Hyde explained that yes it could.

The Committee noted that in the application it mentions that parents may be contacted in the future to consent for their child's notes to be looked at. However if they consented the parents at the beginning they would not have to do this.

The Committee noted the PIS was coercive is places and said it was better to be in study. Also under the heading 'Are there any risks for my baby?' they have said there are no risks which is inaccurate as the researchers do not know if there are and if being on non-routine care will carry extra risks.

<u>Other general comments</u>

The Committee queried why parents are being informed of the results before peer-review publications.

The Committee thought the study title was biased and a more natural title would be better.

Documents reviewed

The documents reviewed at the meeting were:

Document	*Version*	*Date*
Covering letter on headed paper		05 September 2014
Evidence of Sponsor insurance or indemnity (non NHS Sponsors only)		21 July 2014
IRAS Checklist XML [Checklist_10092014]		10 September 2014
Letter from sponsor		21 August 2014
Participant information sheet (PIS)	1.3	01 August 2014
REC Application Form [REC_Form_10092014]		10 September 2014
Research protocol or project proposal	1.3	11 August 2014

Summary CV for Chief Investigator (CI)	1	05 September 2014

Membership of the Committee

The members of the Ethics Committee who were present at the meeting are listed on the attached sheet.

Statement of compliance

The Committee is constituted in accordance with the Governance Arrangements for Research Ethics Committees and complies fully with the Standard Operating Procedures for Research Ethics Committees in the UK.

User Feedback

The Health Research Authority is continually striving to provide a high quality service to all applicants and sponsors. You are invited to give your view of the service you have received and the application procedure. If you wish to make your views known please use the feedback form available on the HRA website: http://www.hra.nhs.uk/about-the-hra/governance/quality-assurance/

HRA Training

We are pleased to welcome researchers and R&D staff at our training days – see details at http://www.hra.nhs.uk/hra-training/

**Please quote this number on all correspondence**

Yours sincerely

Chair

Email:

Enclosures:	*List of names and professions of members who were present at the meeting and those who submitted written comments.*
Copy to:	*Chelsea and Westminster NHS Foundation Trust*

Attendance at Committee meeting

Committee Members:

Name	*Profession*	*Present*	*Notes*
	Retired Pharmacist	Yes	
	Research Governance and Administration Manager	Yes	
	Consultant in Head & Neck Surgery	Yes	
	Lay Member	Yes	
	Project Consultant and Pension Fund Trustee	No	
	Lay member	Yes	
	Consultant in Communicable Diseases	Yes	
	Consultant Anaesthetist	Yes	
	Lay Member	No	
	Lay Member	Yes	
	Transplant Surgery Registrar	No	
	Consultant in Public Health Medicine	No	

Also in attendance:

Name	*Position (or reason for attending)*
	REC Manager
	REC Assistant
