# Supplementary material for: Research ethics committee decision-making in relation to an efficient neonatal trial
Source: Arch Dis Child Fetal Neonatal Ed. 2016 Sep 14;102(4):F291–8. doi: 10.1136/archdischild-2016-310935 (PMC5537508; doi:10.1136/archdischild-2016-310935)
Supplement: Supplementary data [file fetalneonatal-2016-310935supp011.pdf]

Telephone: 0191 4283563

26 November 2014

Dr Chris Gale
NIHR Clinical Lecturer
Imperial College London
Section of Academic Neonatal Medicine
Imperial College London, Chelsea and Westminster Campus
369 Fulham Road
London
SW10 9NH

Dear Dr Gale

Study Title: **The WHEAT trial: WithHolding Enteral feeding Around packed red cell Transfusions in preterm neonates, a multicentre, superiority, randomised registry trial**
REC reference:
Protocol number:
IRAS project ID:

The Research Ethics Committee reviewed the above application at the meeting held on . Thank you and Dr Matthew Hyde for attending to discuss the application.

Provisional opinion

The Committee is unable to give an ethical opinion on the basis of the information and documentation received so far. Before confirming its opinion, the Committee requests that you provide the further information set out below.

Authority to consider your response and to confirm the Committee's final opinion has been delegated to the Chair.

Further information or clarification required

1 The role of and tasks allocated to, the Data Monitoring Committee (DMC) should be amended to accord with normal practice in clinical trials.
2 The study stopping rules should be decided by the research team and submitted for

review by the REC. The DMC should monitor these rules (among other tasks) but not determine them.

3 Recruitment instructions should be prepared for use at for all sites and submitted for review. The instruction should stipulate that, at all recruiting sites:

Wherever possible, both parents/guardians should be present and involved when the study is discussed and consent is sought. Where this is not possible the other parent/guardian must be consulted at the first possible opportunity and their opinion sought.

In all cases where both parent/guardians are consulted if *both* parent/guardians do not agree that their child should take part the child must be considered to be ineligible and cannot take part in the study.

First approach for recruitment must only take place after a live birth has occurred and the mother has survived and is in a stable condition. No parent/guardian should be approached for recruitment purposes before their child has been born.

4 Question A17 – 2 states that there is no exclusion criteria in relation to babies with congenital anomalies. A justification is required as to why babies with any abnormalities or other conditions which would affect their ability to absorb and/or digest feeding are not excluded.

5 Feedback of the results from the study should be given at cohort level only and not individually.

6 Provide details of what steps will be taken to ensure that only the parents of babies who are still alive *at that time* will be contacted to feedback results.

7 The participant information sheet:

- *What is the purpose ... ?* section - where it is states that how babies are cared for during blood transfusions varies across the country; include the information that in 2/3rds of units in the UK babies feeds continue during blood transfusions and in 1/3rd of units in the UK babies feeds are stopped during blood transfusions.

- *Are there any risks ... ?* section - include the information that if they take part some babies will have an additional cannula fitted (re A 19). Give information on what proportion of and under what circumstances (in lay person's language) a participant would require this additional cannula and the risks involved in having an additional cannula fitted.

- *Are there any Benefits* ... ? section - delete the last sentence.

If you would find it helpful to discuss any of the matters raised above or seek further clarification from a member of the Committee, you are welcome to contact REC Manager, in the first instance.

When submitting a response to the Committee, the requested information should be electronically submitted from IRAS. A step-by-step guide on submitting your response to the REC provisional opinion is available on the HRA website using the following link:

http://www.hra.nhs.uk/nhs-research-ethics-committee-rec-submitting-response-provisional-opinion/

Please submit revised documentation where appropriate underlining or otherwise highlighting the changes which have been made and giving revised version numbers and dates. You do not have to make any changes to the REC application form unless you have been specifically requested to do so by the REC.

The Committee will confirm the final ethical opinion within a maximum of 60 days from the date of initial receipt of the application, excluding the time taken by you to respond fully to the above points.   A response should be submitted by no later than 26 December 2014.

Summary of the discussion at the meeting

Other ethical issues were raised and resolved in preliminary discussion before your attendance at the meeting.

You and Dr Matthew Hyde joined the meeting for discussion.

<u>Social or scientific value; scientific design and conduct of the study</u>

Members stated that it appeared that the Data Monitoring Committee (DMC) had not been established yet and that there appeared to be no stopping rules for the study.

You informed members that you knew who you would like to be involved with the DMC and were going to establish it in due course.   You stated that at the first meeting of this DMC the stopping rules for the study would be discussed and guidance subsequently established.   You confirmed that this would be established prior to the start of the trial.

Members stated that it appeared that the applicants were intending that the REC would approve the study, the DMC would then be established and then a substantial amendment would be submitted to the REC regarding stopping rules and the role and tasks assigned to the DMC.

You stated that you did not think that this would be classed as substantial amendment.

Members outlined the role of the DMC in a study and informed the applicants that it was the Chief Investigator's responsibility to undertake the tasks described in A 34 and to establish stopping rules which would be applied and monitored by the DMC.

Members informed you that the sample size calculation for the study could be reproduced. However given the quality of registry data is often not very good, members queried how you would have evidence of quality for the study.

You stated that you did have quality data and the outcomes for the study had been validated against this data although the sample size may have to change.

<u>Favourable risk benefit ratio; anticipated benefit/risks for research participants (present and future)</u>

Members enquired whether it would be possible for a participant to withdraw from the study after not opting out.

You confirmed that there was no time limited point for withdrawal and the participant can go on to have normal care after withdrawing.

Members enquired the meaning of withdrawing from the study.

You stated that this meant that the participants' data would not be used.

Members queried if a baby was randomised to the not-withheld arm of the study and then withdrew, whether there would be any change in care.

You stated that nothing different would happen to the care received and standard care would continue. You stated that this procedure is variable across the country as different centres operate different policies and some units do not withhold at all. Dr Hyde stated that if a participant withdraws from the study, this decision is logged onto the hospital system and no data would be used.

Members noted that in the protocol it says that participants allocated to the feeds withheld arm would also have any enteral medication withheld and it was queried whether there was another way of the participant receiving this medication.

You stated that the medication would be administered by other routes such as IV if possible but some enteral medications are not available in another form. You stated that these medications are prescribed to address issues with feeding such as reflux and so withholding them when feeding was withheld would not be a problem.

Informed consent process and the adequacy and completeness of participant information

Members enquired about the opt-out consenting process that would be used for the study and the process that would be used if both parents did not agree to participation in the study and that with the opt-out approach, there is no written record kept.

You stated that you did not think this process was different to the opt-in approach and that there was proof that participation had been discussed with the participants.

Members enquired whether both parent/guardians would be consulted and if only one was available would the other parent be spoken to after initial consent had been received. Members queried what would happen if both parents were consulted but did not agree.

You confirmed that consent would be reaffirmed throughout the study and you would be guided by the local clinical team. If there were any issues such as parent/guardian disagreements the child would probably not be eligible at most sites.

You and Dr Hyde left the meeting.

Documents reviewed

The documents reviewed at the meeting were:

Document	*Version*	*Date*
Covering letter on headed paper		05 September 2014
Evidence of Sponsor insurance or indemnity (non NHS Sponsors only)		21 July 2014
IRAS Checklist XML [Checklist_10092014]		10 September 2014
Letter from sponsor		21 August 2014
Participant information sheet (PIS)	1.3	01 August 2014
REC Application Form [REC_Form_10092014]		10 September 2014
Research protocol or project proposal	1.3	11 August 2014
Summary CV for Chief Investigator (CI)	1	05 September 2014

Membership of the Committee

The members of the Committee who were present at the meeting are listed on the attached sheet

Statement of compliance

The Committee is constituted in accordance with the Governance Arrangements for Research Ethics Committees and complies fully with the Standard Operating Procedures for Research Ethics Committees in the UK.

Please quote this number on all correspondence

Yours sincerely

pp

Chair

Email:

Enclosures: *List of names and professions of members who were present at the meeting and those who submitted written comments.*

Copy to: Imperial College London

Chelsea and Westminster NHS Foundation Trust

Attendance at Committee meeting

Committee Members:

Name	*Profession*	*Present*	*Notes*
	NCRN Team Lead	Yes	
	Research Assistant/Statistician	No	
	Retired Educationalist	Yes	
	Historian	Yes	
	Head of Cardiac and Respiratory Services	Yes	
	Consultant Psychiatrist	No	
	Principal Lecturer in Research Governance	Yes	
	Part-time Biology Lecturer (Retired)	No	
	Clinical Lead Pharmacist	Yes	
	Consultant	Yes	
	Statistician	Yes	
	Data Manager, Clinical Research	Yes	
	Clinical Trials Co-ordinator	No	
	Part-time Biology Lecturer (Retired)	No	

Also in attendance:

Name	*Position (or reason for attending)*
	Phase 1 Oncology Trials Co-ordinator
	REC Manager

Imperial College London

Section of Neonatal Medicine
Imperial College London

4th Floor, Lift Bank D
Chelsea and Westminster Hospital
369 Fulham Road
London, SW10 9NH
Tel: +44 (0)203 3153519
Fax: +44 (0)203 3157998

christopher.gale@imperial.ac.uk

14th January 2015

Dr Christopher Gale MBBS MSc PhD MRCPCH

Dear

Study title: **The WHEAT trial: With Holding Enteral feeding Around packed red cell Transfusions in preterm neonates, a multicentre, superiority, randomised registry trial**

REC reference:
Protocol number:
IRAS project ID:

Thank you for taking the time to review the WHEAT trial. Please find responses to REC comments detailed below.

1. *The role of and tasks allocated to, the Data Monitoring Committee (DMC) should be amended to accord with normal practice in clinical trials.*
 - Please see below.

2. *The study stopping rules should be decided by the research team and submitted for review by the REC. The DMC should monitor these rules (among other tasks) but not determine them.*
 - As discussed at the committee meeting, the data monitoring committee (DMC) will be established before recruitment starts. In accordance with advice from the DAMOCLES Study Group (Lancet 2005) the DMC will establish a Charter at their initial meeting that will formalise the terms of reference of the DMC. The DMC will be expected to meet at least 6 monthly with a planned interim analysis after 12 months of recruitment; this will be outlined in the DMC charter. The point at which recruitment would be stopped will be determined by the DMC in line with the DAMOCLES statement: "*Statistical issues should be only one of several considerations that a DMC needs to take into account. Other considerations include the balance of primary risks and benefits, the internal consistency of results, the consistency with, and nature of, external evidence, and the likelihood that the results would affect clinical practice.*" Statistical criteria will be determined by the DMC at their initial meeting and recorded in the DMC Charter (a copy of which will be provided to the REC when finalised); these will be "regarded as guidelines for recommending stopping rather than rules" (DAMOCLES, Lancet 2005). This has been clarified in the protocol (section 21, page 29)

3. *Recruitment instructions should be prepared for use at for all sites and submitted for review. The instruction should stipulate that, at all recruiting sites:*
- Wherever possible, both parents/guardians should be present and involved when the study is discussed and consent is sought. Where this is not possible the other parent/guardian must be consulted at the first possible opportunity and their opinion sought.

- In all cases where both parent/guardians are consulted if both parent/guardians do not agree that their child should take part the child must be considered to be ineligible and cannot take part in the study.
- First approach for recruitment must only take place after a live birth has occurred and the mother has survived and is in a stable condition. No parent/guardian should be approached for recruitment purposes before their child has been born.

- Recruitment instructions have been prepared and include these stipulations.

4. *Question A17 – 2 states that there are no exclusion criteria in relation to babies with congenital anomalies. A justification is required as to why babies with any abnormalities or other conditions which would affect their ability to absorb and/or digest feeding are not excluded.*
   - WHEAT is a pragmatic trial and as such we have endeavoured to limit the exclusion criteria as much as possible in order to make the results as generalisable to neonatal practice. A baby born with a condition that permanently limits their ability to be enterally fed would not be eligible for inclusion in WHEAT and we have made this explicit in the protocol (section 10, page 9).
   - In cases where a condition precludes immediate enteral feeding but is reversible (for example duodenal atresia), such a baby would be eligible for inclusion (with consent sought in the usual way).

5. *Feedback of the results from the study should be given at cohort level only and not individually.*
   - It is usual practice in multicentre neonatal trials to provide feedback of trial results (in the form of a standardised letter or email) to all parents. This has been the case for previous neonatal clinical trials (PROGRAMS, BOOST II, PIPS, TOBY) and other neonatal studies (EPICURE 1 and EPICURE 2).

6. Provide details of what steps will be taken to ensure that only the parents of babies who are still alive *at that time* will be contacted to feedback results.
   - We are aware of no evidence that indicates that parents whose baby died should not informed of the results of research in which their baby participated. Professionals are fearful of adding to distress of parents. However, focus group work with parents of babies admitted to neonatal intensive care shows that they would rather be included, and be sent the results of research in which their baby took part, even if their baby died. We therefore feel that it is paternalistic to decide which parents should and should not be given the results of research, and that instead information should be provided that is appropriate and sensitively phrased. To ensure this, the feedback process will be led by the parent and parent representative members of the trial steering group.

7. *The participant information sheet:*
What is the purpose ... ? section - where it is states that how babies are cared for during blood transfusions varies across the country; include the information that in 2/3rds of units in the UK babies feeds continue during blood transfusions and in 1/3rd of units in the UK babies feeds are stopped during blood transfusions.
   - We have added the following statement to this section: "In 2011 approximately 1 in 3 neonatal units in England stopped feeds in this way while the remaining neonatal units did not."

Are there any risks ... ? section - include the information that if they take part some babies will have an additional cannula fitted (re A 19). Give information on what proportion of and under what circumstances (in lay person's language) a participant would require this additional cannula and the risks involved in having an additional cannula fitted.
   - Both of the treatment arms in WHEAT are in common use across the UK (Parige et al, ADC FN 2013, attached). Therefore both treatment options (including the additional IV line) are part of routine clinical care in the UK. While it is true that in neonatal units

where feeds are not currently withheld around transfusion, and where a baby is randomised to have feeds withheld they may require an IV line that they would not have required **at that unit** had they not been in the trial, this IV line is **not** outside of routine clinical care in the UK. Conversely, in neonatal units where feeds are routinely withheld around transfusion, where a baby is randomised to feeding around transfusion, they will **avoid** an IV line that they would have otherwise required had they not been in the trial.

- Explaining both of these possible scenarios (requiring and additional IV line or avoiding an IV line dependent on the neonatal unit or clinician) will make the PIS confusing and the study more difficult to understand. For this reason and because both treatment arms are routine clinical care (and the IV line is therefore not a research procedure), we are reluctant to lengthen the Participant Information Sheet in this manner. This decision has been discussed extensively with, and ultimately shaped by, the parent member of the trial development group (a parent of 26 week gestation twins) and representatives of the national charity Bliss.
- If the REC insist on this point we would be willing to insert a sentence into the Patient Information Sheet that attempts to clearly explain both potential scenarios: "If your baby is being cared for in a unit which does not currently withhold feeds during blood transfusion, and does not already have a cannula to supply sugar or food directly into the blood, and is randomised to not receive feeding during their transfusion, they may require an additional cannula in order to provide them with sugar during their blood transfusion (this will only apply to a very small number of babies). Conversely, if your baby is in a unit which currently withholds feeds and it randomised to receive feeds during blood transfusion, then your baby may be spared a cannula that it may have required if it was not taking part in the WHEAT trial." We are however concerned that the addition of this statement may result in a biased study population (whereby consent is greater in units where current practice is to withhold feeds) meaning that any study results are less generalisable.

Are there any Benefits ... ? section - delete the last sentence.

- The evidence for inclusion benefit in neonatal clinical trials is compelling, with some of the most conclusive and recent evidence coming from a large clinical trial that enrolled only babies (Carlo et al, NEJM 2012; attached). Our statement thus represents current scientific knowledge. We feel it is important that this important information is not withheld from parents. Providing this information ensures that they are truly fully informed.
- We acknowledge the committee's point regarding the wording and have replaced the statement "*This non-evidence based approach to neonatal care may involve more risk than being in a study like WHEAT which involves a carefully designed protocol and consistent monitoring*" with "*taking part in a research study may confer non-specific benefits*" (changes highlighted in the Participant Information Sheet).
  We would encourage the committee to watch the following video clip by the renowned ethicist and Professor of Paediatric Bioethics, John Lantos, that further illustrates the rationale for our view (https://www.youtube.com/watch?v=SmWJnOp1QaU). We do not feel parents can make an informed decision about a study without knowing both the risks and the potential benefits. We feel that it is not sufficient for these benefits to simply be alluded to on a generic information sheet about research participation, but they should be treated in the same way as potential research risks, and included on the Participant Information Sheet.

I hope these responses provide sufficient clarification. Please do not hesitate to contact us if you require any further information.

Documents attached:

Document	Version	Date
Participant Information Sheet	1.4	12 January 2015

Recruitment Instructions	1.0	13 January 2015
Carlo et al., NEJM		2012
Parige et al., ADC FN		2013
Protocol	1.4	12 January 2015

Yours sincerely

Dr Chris Gale
NIHR Clinical Lecturer in Paediatrics

Imperial College London

Section of Neonatal Medicine
Imperial College London

4th Floor, Lift Bank D
Chelsea and Westminster Hospital
369 Fulham Road
London, SW10 9NH
Tel: +44 (0)203 3153519
Fax: +44 (0)203 3157998

christopher.gale@imperial.ac.uk

19th January 2015

Dr Christopher Gale MBBS MSc PhD MRCPCH

Dear ,

Study title: **The WHEAT trial: With Holding Enteral feeding Around packed red cell Transfusions in preterm neonates, a multicentre, superiority, randomised registry trial**

REC reference:
Protocol number:
IRAS project ID:

Please find attached further correspondence in relation to point 6, apologies that this was not included in the response dated 14/1/2015.

6. Provide details of what steps will be taken to ensure that only the parents of babies who are still alive *at that time* will be contacted to feedback results.
   - In addition to the points raised in our previous correspondence dated 14/1/2015 we would like to draw the Committee's attention to the results of the BRACELET study. This study considered bereavement subsequent to enrolment in neonatal intensive care trials through qualitative and quantitative methodology. Parents who had taken part in a neonatal trials and whose baby had died were interviewed and "in almost every interview parents said that they would want to have the results". The authors state, "This almost unanimous view that the parents should have access to the trial results was an important finding".
   - The NIHR HTA Journal full report of the BRACELET study is found at the following URL (the relevant section is pages 185-203): http://www.journalslibrary.nihr.ac.uk/__data/assets/pdf_file/0009/121131/FullReport-hta18420.pdf

I hope this provides additional clarification and will be considered in conjunction with the previous letter (dated 14/1/2015).

Yours sincerely

Dr Chris Gale
NIHR Clinical Lecturer in Paediatrics

The WHEAT Study (With-Holding Enteral feeds Around Transfusion)

Recruitment Instructions

Inclusion criteria:
Infants eligible for the WHEAT trial must comply with all of the following at randomisation:
1. Post-menstrual age at birth <30 weeks (up to and including 29+6 weeks).

Exclusion criteria:
Babies with a condition that limits their ability to absorb/digest feeds, where this condition is not reversible (for example through a surgical procedure).

Infants enrolled in other interventional studies are eligible for participation in the WHEAT trial unless prohibited by the other study.

Recruitment will be by "opt-out consent". Parents/carers will be approached by a member of the local clinical team who will explain the WHEAT study and the "opt out" process within 24 hours of admission (a discussion between parents/cares and a senior member of the medical team within 24 hours of neonatal unit admission is a nationally agreed standard and is part of the National Neonatal Audit Progamme). Parents/carers who do not opt out will be enrolled in WHEAT. Parents/carers can opt-out at any time.

First approach for recruitment must only take place after a live birth has occurred and the mother has survived and is in a stable condition. No parent/guardian should be approached for recruitment purposes before their child has been born.

In participating units, data entered electronically into the "admission summary" will be interrogated in real time to identify and infants meeting the WHEAT study inclusion criteria will be flagged; an electronic reminder will appear on the electronic health record (Badger system) at the participating unit. This "flag" will inform the health professional that the infant is eligible for the WHEAT trial, provide trial information, investigator contact details, and link to electronic copies of the patient information sheet. The electronic health record will subsequently present the health care professional with a question asking whether the WHEAT trial and the "opt-out" process have been fully explained to the parents. If parents "opt-out" this will be recorded in the EHR. If parents do not "opt-out", randomisation will occur electronically after the health professional has recorded that the WHEAT trial and the "opt-out" process have been fully explained.

Wherever possible, both parents/guardians should be present and involved when the study is discussed and opt-out consent is sought. Where this is not possible the other parent/guardian must be consulted at the first possible opportunity and their opinion sought. In all cases where both parent/guardians are consulted if both parent/guardians do not agree that their infant should take part the infant must be considered to be ineligible and cannot take part in the study.

Health Research Authority

27 January 2015

Dr Chris Gale
NIHR Clinical Lecturer
Imperial College London
Section of Academic Neonatal Medicine,
Imperial College London, Chelsea and Westminster Campus,
369 Fulham Road
London SW10 9NH

Dear Dr Gale

**Study title:**	**The WHEAT trial: WithHolding Enteral feeding Around packed red cell Transfusions in preterm neonates, a multicentre, superiority, randomised registry trial**
**REC reference:**	
**Protocol number:**	
**IRAS project ID:**	

Thank you for your letter of 14 January 2015, responding to the Committee's request for further information on the above research [and submitting revised documentation].

The further information was considered at the meeting of the Committee held on . A list of the members who were present at the meeting is attached.

We plan to publish your research summary wording for the above study on the HRA website, together with your contact details. Publication will be no earlier than three months from the date of this favourable opinion letter. The expectation is that this information will be published for all studies that receive an ethical opinion but should you wish to provide a substitute contact point, wish to make a request to defer, or require further information, please contact the REC Manager. Under very limited circumstances (e.g. for student research which has received an unfavourable opinion), it may be possible to grant an exemption to the publication of the study.

Confirmation of ethical opinion

On behalf of the Committee, I am pleased to confirm a **favourable ethical opinion** for the above research on the basis described in the application form, protocol and supporting documentation [as revised], subject to the conditions specified below.

Conditions of the favourable opinion

The favourable opinion is subject to the following conditions being met prior to the start of the study.

1 When feeding back trial result no results from individual participants should be given to the participants' parent/guardian only the aggregated results (study findings at cohort level) should be given.

2 The text,

“If your baby is being cared for in a unit which does not currently withhold feeds during blood transfusion, and does not already have a cannula to supply sugar or food directly into the blood, and is randomised to not receive feeding during their transfusion, they may require an additional cannula in order to provide them with sugar during their blood transfusion (this will only apply to a very small number of babies). Conversely, if your baby is in a unit which currently withholds feeds and it randomised to receive feeds during blood transfusion, then your baby may be spared a cannula that it may have required if it was not taking part in the WHEAT trial.” to be added to the ‘Are there any risks ... ? section of the Participant Information Sheet’.

You should notify the REC in writing once all conditions have been met (except for site approvals from host organisations) and provide copies of any revised documentation with updated version numbers. The REC will acknowledge receipt and provide a final list of the approved documentation for the study, which can be made available to host organisations to facilitate their permission for the study. Failure to provide the final versions to the REC may cause delay in obtaining permissions.

<u>Management permission or approval must be obtained from each host organisation prior to the start of the study at the site concerned.</u>

Management permission ("R&D approval") should be sought from all NHS organisations involved in the study in accordance with NHS research governance arrangements.

Guidance on applying for NHS permission for research is available in the Integrated Research Application System or at http://www.rdforum.nhs.uk.

Where a NHS organisation’s role in the study is limited to identifying and referring potential participants to research sites ("participant identification centre"), guidance should be sought from the R&D office on the information it requires to give permission for this activity.

For non-NHS sites, site management permission should be obtained in accordance with the procedures of the relevant host organisation.

Sponsors are not required to notify the Committee of approvals from host organisations

Registration of Clinical Trials

All clinical trials (defined as the first four categories on the IRAS filter page) must be registered on a publically accessible database. This should be before the first participant is recruited but no later than 6 weeks after recruitment of the first participant.

There is no requirement to separately notify the REC but you should do so at the earliest opportunity e.g. when submitting an amendment. We will audit the registration details as part of the annual progress reporting process.

To ensure transparency in research, we strongly recommend that all research is registered but for non-clinical trials this is not currently mandatory.

If a sponsor wishes to request a deferral for study registration within the required timeframe, they should contact hra.studyregistration@nhs.net. The expectation is that all clinical trials will be registered, however, in exceptional circumstances non registration may be permissible with prior agreement from NRES. Guidance on where to register is provided on the HRA website.

It is the responsibility of the sponsor to ensure that all the conditions are complied with before the start of the study or its initiation at a particular site (as applicable).

Ethical review of research sites

NHS sites

The favourable opinion applies to all NHS sites taking part in the study, subject to management permission being obtained from the NHS/HSC R&D office prior to the start of the study (see "Conditions of the favourable opinion" below).

Non-NHS sites

The Committee has not yet completed any site-specific assessment (SSA) for the non-NHS research site(s) taking part in this study. The favourable opinion does not therefore apply to any non-NHS site at present. We will write to you again as soon as an SSA application(s) has been reviewed. In the meantime no study procedures should be initiated at non-NHS sites.

Approved documents

The final list of documents reviewed and approved by the Committee is as follows:

Document	*Version*	*Date*
Covering letter on headed paper		05 September 2014
Evidence of Sponsor insurance or indemnity (non NHS Sponsors only)		21 July 2014
IRAS Checklist XML [Checklist_14012015]		14 January 2015
Letter from sponsor		21 August 2014
Other [Response Letter]	1	14 January 2015
Other [Carlo - Supporting Paper]	1	14 January 2015
Other [Parige - Supporting Paper]	1	14 January 2015

Other [Recruitment Instructions]	1	13 January 2015
Other [Revised PIS]	1.4	12 January 2015
Other [Revised Protocol]	1.4	12 January 2015
Other [Additional response to provisional]		19 January 2015
REC Application Form [REC_Form_10092014]		10 September 2014
Response to Request for Further Information		14 January 2015
Summary CV for Chief Investigator (CI)	1	05 September 2014

Statement of compliance

The Committee is constituted in accordance with the Governance Arrangements for Research Ethics Committees and complies fully with the Standard Operating Procedures for Research Ethics Committees in the UK.

After ethical review

Reporting requirements

The attached document *"After ethical review – guidance for researchers"* gives detailed guidance on reporting requirements for studies with a favourable opinion, including:

- Notifying substantial amendments
- Adding new sites and investigators
- Notification of serious breaches of the protocol
- Progress and safety reports
- Notifying the end of the study

The HRA website also provides guidance on these topics, which is updated in the light of changes in reporting requirements or procedures.

User Feedback

The Health Research Authority is continually striving to provide a high quality service to all applicants and sponsors. You are invited to give your view of the service you have received and the application procedure. If you wish to make your views known please use the feedback form available on the HRA website:
http://www.hra.nhs.uk/about-the-hra/governance/quality-assurance/

HRA Training

We are pleased to welcome researchers and R&D staff at our training days – see details at http://www.hra.nhs.uk/hra-training/

Please quote this number on all correspondence

With the Committee's best wishes for the success of this project.

Yours sincerely

pp

Chair

Email:

Enclosures:	*List of names and professions of members who were present at the meeting and those who submitted written comments*
	“After ethical review – guidance for researchers
Copy to:	Imperial College London
	Chelsea and Westminster NHS Foundation Trust

Attendance at Committee meeting

Committee Members:

Name	*Profession*	*Present*	*Notes*
	ECMC & CTU Manager	Yes	
	Research Assistant/Statistician	Yes	
	Retired Educationalist	Yes	
	Historian	No	
	Head of Cardiac and Respiratory Services	No	
	Consultant Psychiatrist	Yes	
	Principal Lecturer in Research Governance	Yes	
	Part-time Biology Lecturer (Retired)	Yes	
	Clinical Lead Pharmacist	Yes	
	Consultant	Yes	
	Statistician	No	
	Data Manager, Clinical Research	Yes	
	Clinical Trials Co-ordinator	No	

Also in attendance:

Name	*Position (or reason for attending)*
	REC Assistant
	REC Manager
