# Supplementary material for: Research ethics committee decision-making in relation to an efficient neonatal trial
Source: Arch Dis Child Fetal Neonatal Ed. 2016 Sep 14;102(4):F291–8. doi: 10.1136/archdischild-2016-310935 (PMC5537508; doi:10.1136/archdischild-2016-310935)
Supplement: Supplementary data [file fetalneonatal-2016-310935supp012.pdf]

Health Research Authority

27 November 2014

Dr Chris Gale
Imperial College London,
Chelsea and Westminster Campus,
369 Fulham Road, London
SW10 9NH

Dear Dr Gale,

**Study Title:**	**The WHEAT trial: WithHolding Enteral feeding Around packed red cell Transfusions in preterm neonates, a multicentre, superiority, randomised registry trial**
**REC reference:**	
**Protocol number:**	
**IRAS project ID:**	

The Research Ethics Committee reviewed the above application at the meeting held . Thank you to yourself and Matthew J. Hyde for attending to discuss the application.

Provisional opinion

The Committee is unable to give an ethical opinion on the basis of the information and documentation received so far. Before confirming its opinion, the Committee requests that you provide the further information set out below.

Authority to consider your response and to confirm the Committee's final opinion has been delegated to the Chair.

Further information or clarification required

1) *PALS (or its equivalent) to be included in the Participant Information Sheet.*
2) *Clarification as to how long parents will have to decide if they would want to participate to be included in the PIS*
3) *The REC name should be included under the heading "Who has reviewed the study?"*
4) *Clarification as to whether Wales will be included in the trial as stated in the IRAS form documents will be translated if necessary.*
5) *An opt out form should be included for parents to keep for their own records.*
6) *Confirmation of trial registration is required.*

If you would find it helpful to discuss any of the matters raised above or seek further clarification from a member of the Committee, you are welcome to contact the REC Manager.

When submitting a response to the Committee, the requested information should be electronically submitted from IRAS. A step-by-step guide on submitting your response to the REC provisional opinion is available on the HRA website using the following link: http://www.hra.nhs.uk/nhs-research-ethics-committee-rec-submitting-response-provisional-opinion/

Please submit revised documentation where appropriate underlining or otherwise highlighting the changes which have been made and giving revised version numbers and dates. You do not have to make any changes to the REC application form unless you have been specifically requested to do so by the REC.

The Committee will confirm the final ethical opinion within a maximum of 60 days from the date of initial receipt of the application, excluding the time taken by you to respond fully to the above points. A response should be submitted by no later than 27 December 2014.

Summary of the discussion at the meeting

Social or scientific value; scientific design and conduct of the study

- The committee questioned if an audit could have been carried out first rather than the study trial. If permission could be granted from the NIGB to look at data collected already. *You explained the number of observational studies already completed suggests there is a benefit to completing a trial*

Recruitment arrangements and access to health information, and fair participant selection

- It was questioned if the inclusion of the units involved would have a bias to how the trial is conducted and how this would affect the outcome. *You explained this is something you will have to be aware of. If a unit had a clear bias they would not be included. You had carried out a national survey and around 90% would be willing to randomise for the study.*

- It was asked who would be approaching parents, and when and how this would be achieved. *You explained the approach would depend on the different units. This can either be by the Clinical Team or by a research nurse of which are trained in approaching parents and consenting. Parents would be approached after 24 hours and would be given the information sheet of which an opt out process has been incorporated instead of opting in. An ongoing discussion will be had with parents until the blood transfusion as some babies could have this early on. You explained it is difficult to say what the exact timing would be.*

- The committee questioned what the extra burden for parents would be. *You commented in the majority of cases parents would have longer than the 24 hours to decide, as some would have many days or possibly even weeks to discuss on this process.*

- It was asked as parents would be opting out of the trial, if they could have a signed note from the Clinician to say they have done this. *You explained a record of this would be kept electronically and a note would be kept on the babies cots to say they are not in the trial.* The committee agreed although visual notes are good, a hard copy of this agreement for parents would be better and asked if this would be something that could be done if it was asked for. *You explained this could be done.*

- The committee questioned who would be able to see the labels on the cots to say which particular babies are involved in the trial. *You confirmed it is standard practice to have these written on the side of the cots and will be visible to everyone to see. You also said parents like the labels on cots and will often discuss with each other about their involvement.*

- It was asked why there had been no exclusion criteria included in the application and if babies who are extremely compromised would not be included as this would be an extra burden on participants. *You explained this would be left up to the Clinician's judgement.* The committee asked if something would be included in the protocol to reflect this. *You confirmed this will be included.*

Informed consent process and the adequacy and completeness of participant information

- It was noted the IRAS form states documents will be translated into Welsh when needed, however could not find any evidence Wales would be participating in the study. Clarification is sought.

Other ethical issues were raised and resolved in preliminary discussion before your attendance at the meeting.

Documents reviewed

The documents reviewed at the meeting were:

Document	*Version*	*Date*
Covering letter on headed paper		05 September 2014
Evidence of Sponsor insurance or indemnity (non NHS Sponsors only)		05 September 2014
IRAS Checklist XML [Checklist_10092014]		10 September 2014
Letter from sponsor		05 September 2014
Participant information sheet (PIS)		02 September 2014
REC Application Form [REC_Form_10092014]		10 September 2014
Research protocol or project proposal		11 August 2014
Summary CV for Chief Investigator (CI)	1	05 September 2014

Membership of the Committee

The members of the Committee who were present at the meeting are listed on the attached sheet

Statement of compliance

The Committee is constituted in accordance with the Governance Arrangements for Research Ethics Committees and complies fully with the Standard Operating Procedures for Research Ethics Committees in the UK.

**Please quote this number on all correspondence**

Yours sincerely,

Chair

Email:

Enclosures: *List of names and professions of members who were present at the meeting and those who submitted written comments.*

Copy to: *Chelsea and Westminster NHS Foundation Trust*

Attendance at Committee meeting

Committee Members:

Name	*Profession*	*Present*	*Notes*
	Senior Lecturer in Nursing	Yes	
	Industrial Pharmacy Consultant and Locum Pharmacist	Yes	
	Lay Plus Member	Yes	
	Lay Member	Yes	
	Staff Nurse	Yes	
	Head of Research -MND Association	Yes	
	Senior Research Officer	Yes	
	cardiology research nurse	Yes	
	Lay Member	Yes	
	Lay Plus Member	Yes	
	Medical Devices Manager	Yes	

Also in attendance:

Name	*Position (or reason for attending)*
	REC Manager

Imperial College London

Section of Neonatal Medicine
Imperial College London

4th Floor, Lift Bank D
Chelsea and Westminster Hospital
369 Fulham Road
London, SW10 9NH
Tel: +44 (0)203 3153519
Fax: +44 (0)203 3157998

christopher.gale@imperial.ac.uk

12th January 2015

Dr Christopher Gale MBBS MSc PhD MRCPCH

Dear,

**Study title:**	**The WHEAT trial: With Holding Enteral feeding Around packed red cell Transfusions in preterm neonates, a multicentre, superiority, randomised registry trial**
**REC reference:**	
**Protocol number:**	
**IRAS project ID:**	

Thank you for taking the time to review the WHEAT trial. Please find responses to your requests for further information detailed below:

1) PALS (or its equivalent) to be included in the Participant Information Sheet.
- We have added the following statement to the end of the Participant Information Sheet: "You can also discuss this study with the Patient Advice and Liaison Services (PALS): [telephone number]"

2) Clarification as to how long parents will have to decide if they would want to participate to be included in the PIS
- We have included the following statement in the section entitled **Does my baby have to take part?** in the Participant Information Sheet: "within 4 days of your baby being born."

3) The REC name should be included under the heading "Who has reviewed the study?"
- We have modified this section accordingly.

4) Clarification as to whether Wales will be included in the trial as stated in the IRAS form documents will be translated if necessary.
- The National Neonatal Research Database (NNRD) does not currently hold the necessary approvals for Wales so at present Wales will not be included in WHEAT. We hope to gain these approvals in the future, at which point we will apply for an amendment and translate the documents as required.

5) An opt out form should be included for parents to keep for their own records.
- We agree with the committee that a physical reminder for parents that they have agreed to enrol their baby in WHEAT is important. We have produced a card to be given to parents to remind them that their baby is taking part in WHEAT and who to contact if they have any queries or wish to withdraw at any point.

6) Confirmation of trial registration is required.

- We can confirm that the trial will be registered following REC approval and prior to recruitment. Registration at clinicaltrials.gov is in process but cannot be completed until REC approval has been obtained.

I hope these responses provide sufficient clarification, please do not hesitate to contact us if you require any further information.

Documents attached:

Document	Version	Date
Participant Information Sheet	1.4	12th January 2015
WHEAT trial participant card	1.0	12th January 2015

Yours Sincerely,

Dr Chris Gale
NIHR Clinical Lecturer in Paediatrics

Your baby is in the WHEAT trial.
WHEAT is trying to find out how best to care for premature babies who need a blood transfusion.

If at any time you wish to withdraw your baby from WHEAT please speak to any doctor or nurse or call [insert local research nurse number]

20 January 2015

Dr Chris Gale
Imperial College London,
Chelsea and Westminster Campus,
369 Fulham Road, London
SW10 9NH

Dear Dr Gale,

**Study title:**	**The WHEAT trial: WithHolding Enteral feeding Around packed red cell Transfusions in preterm neonates, a multicentre, superiority, randomised registry trial**
**REC reference:**	
**Protocol number:**	
**IRAS project ID:**	

Thank you for your letter of 12 January 2015, responding to the Committee's request for further information on the above research and submitting revised documentation.

The further information has been considered on behalf of the Committee by the Chair.

We plan to publish your research summary wording for the above study on the HRA website, together with your contact details. Publication will be no earlier than three months from the date of this favourable opinion letter. The expectation is that this information will be published for all studies that receive an ethical opinion but should you wish to provide a substitute contact point, wish to make a request to defer, or require further information, please contact the REC Manager. Under very limited circumstances (e.g. for student research which has received an unfavourable opinion), it may be possible to grant an exemption to the publication of the study.

Confirmation of ethical opinion

On behalf of the Committee, I am pleased to confirm a favourable ethical opinion for the above research on the basis described in the application form, protocol and supporting documentation as revised, subject to the conditions specified below.

Conditions of the favourable opinion

The favourable opinion is subject to the following conditions being met prior to the start of the study.

Management permission or approval must be obtained from each host organisation prior to the start of the study at the site concerned.

Management permission ("R&D approval") should be sought from all NHS organisations involved in the study in accordance with NHS research governance arrangements.

Guidance on applying for NHS permission for research is available in the Integrated Research Application System or at http://www.rdforum.nhs.uk.

Where a NHS organisation's role in the study is limited to identifying and referring potential participants to research sites ("participant identification centre"), guidance should be sought from the R&D office on the information it requires to give permission for this activity.

For non-NHS sites, site management permission should be obtained in accordance with the procedures of the relevant host organisation.

Sponsors are not required to notify the Committee of approvals from host organisations

Registration of Clinical Trials

All clinical trials (defined as the first four categories on the IRAS filter page) must be registered on a publically accessible database. This should be before the first participant is recruited but no later than 6 weeks after recruitment of the first participant.

There is no requirement to separately notify the REC but you should do so at the earliest opportunity e.g. when submitting an amendment. We will audit the registration details as part of the annual progress reporting process.

To ensure transparency in research, we strongly recommend that all research is registered but for non-clinical trials this is not currently mandatory.

If a sponsor wishes to request a deferral for study registration within the required timeframe, they should contact hra.studyregistration@nhs.net. The expectation is that all clinical trials will be registered, however, in exceptional circumstances non registration may be permissible with prior agreement from NRES. Guidance on where to register is provided on the HRA website.

It is the responsibility of the sponsor to ensure that all the conditions are complied with before the start of the study or its initiation at a particular site (as applicable).

Ethical review of research sites

NHS sites

The favourable opinion applies to all NHS sites taking part in the study, subject to management permission being obtained from the NHS/HSC R&D office prior to the start of the study (see "Conditions of the favourable opinion" below).

Non-NHS sites

The Committee has not yet completed any site-specific assessment (SSA) for the non-NHS research site(s) taking part in this study. The favourable opinion does not therefore apply to any non-NHS site at present. We will write to you again as soon as an SSA application(s) has been reviewed. In the meantime no study procedures should be initiated at non-NHS sites.

Approved documents

The final list of documents reviewed and approved by the Committee is as follows:

Document	*Version*	*Date*
Covering letter on headed paper		05 September 2014
Evidence of Sponsor insurance or indemnity (non NHS Sponsors only)		05 September 2014
IRAS Checklist XML [Checklist_19012015]		19 January 2015
Letter from sponsor		05 September 2014
Other [WHEAT Participation Card]	1.0	12 January 2015
Participant information sheet (PIS)		02 September 2014
Participant information sheet (PIS)	1.4	12 January 2015
REC Application Form [REC_Form_19012015]		19 January 2015
Research protocol or project proposal		11 August 2014
Response to Request for Further Information		12 January 2015
Summary CV for Chief Investigator (CI)	1	05 September 2014

Statement of compliance

The Committee is constituted in accordance with the Governance Arrangements for Research Ethics Committees and complies fully with the Standard Operating Procedures for Research Ethics Committees in the UK.

After ethical review

<u>Reporting requirements</u>

The attached document *"After ethical review – guidance for researchers"* gives detailed guidance on reporting requirements for studies with a favourable opinion, including:

- Notifying substantial amendments
- Adding new sites and investigators
- Notification of serious breaches of the protocol
- Progress and safety reports
- Notifying the end of the study

The HRA website also provides guidance on these topics, which is updated in the light of changes in reporting requirements or procedures.

User Feedback

The Health Research Authority is continually striving to provide a high quality service to all applicants and sponsors. You are invited to give your view of the service you have received and the application procedure. If you wish to make your views known please use the feedback form available on the HRA website: http://www.hra.nhs.uk/about-the-hra/governance/quality-assurance/

HRA Training

We are pleased to welcome researchers and R&D staff at our training days – see details at http://www.hra.nhs.uk/hra-training/

**Please quote this number on all correspondence**

With the Committee's best wishes for the success of this project.

Yours sincerely,

Chair

Enclosures: "After ethical review – guidance for

researchers"

Copy to: *Chelsea and Westminster NHS Foundation Trust*
