# Supplementary material for: Research ethics committee decision-making in relation to an efficient neonatal trial
Source: Arch Dis Child Fetal Neonatal Ed. 2016 Sep 14;102(4):F291–8. doi: 10.1136/archdischild-2016-310935 (PMC5537508; doi:10.1136/archdischild-2016-310935)
Supplement: Supplementary data [file fetalneonatal-2016-310935supp013.pdf]

NHS
Health Research Authority

13 October 2014

Dr Chris Gale
NIHR Clinical Lecturer
Imperial College London
Section of Academic Neonatal Medicine,
Imperial College London, Chelsea and Westminster Campus,
369 Fulham Road, London
SW10 9NH

Dear Dr Gale

**Study Title:**	**The WHEAT trial: WithHolding Enteral feeding Around packed red cell Transfusions in preterm neonates, a multicentre, superiority, randomised registry trial**
**REC reference:**	
**Protocol number:**	
**IRAS project ID:**	

The Research Ethics Committee reviewed the above application at the meeting held on . Dr Matthew Hyde joined the meeting by telephone.

Provisional opinion

The Committee is unable to give an ethical opinion on the basis of the information and documentation received so far. Before confirming its opinion, the Committee requests that you provide the further information set out below.

Authority to consider your response and to confirm the Committee's final opinion has been delegated to the Chair.

Further information or clarification required

1. Please formally document the parents' decision to not opt out of the study.

2. Please remove the last sentence of the 'Are there any benefits to my baby?' section of the Patient Information and replace with 'taking part in a research study may confer

non-specific benefits.'

3. Please add PALS contact details to the Patient Information Sheet for information.

If you would find it helpful to discuss any of the matters raised above or seek further clarification from a member of the Committee, you are welcome to contact the REC Manager at .

When submitting a response to the Committee, the requested information should be electronically submitted from IRAS. A step-by-step guide on submitting your response to the REC provisional opinion is available on the HRA website using the following link: http://www.hra.nhs.uk/nhs-research-ethics-committee-rec-submitting-response-provisional-opinion/

Please submit revised documentation where appropriate underlining or otherwise highlighting the changes which have been made and giving revised version numbers and dates. You do not have to make any changes to the REC application form unless you have been specifically requested to do so by the REC.

The Committee will confirm the final ethical opinion within a maximum of 60 days from the date of initial receipt of the application, excluding the time taken by you to respond fully to the above points. A response should be submitted by no later than 12 November 2014.

Summary of the discussion at the meeting

Social or scientific value; scientific design and conduct of the study

The Committee asked about the need for the study and whether the objective could be reached by auditing currently available data.

The applicant said similar audit studies had not been conclusive and that it would be difficult to identify a causal link without conducting a randomised trial.

Informed consent process and the adequacy and completeness of participant information

The Committee asked why the study had been designed as an opt-out trial whereby participants are part of the trial unless they specifically choose to opt out.

The applicant explained the study is not an interventional trial and is a study of two current practices. The babies could already have been considered to be randomised due to different hospitals choosing to use one of the practices in preference to the other.

The study was designed as opt out to maximise recruitment to the trial and also to reduce the burden of decision making on parents whose baby is admitted to the neonatal unit. It was also considered that an opt in study may result in biased groups. The applicant advised parents have been involved in the design of the study and were comfortable with the opt out approach.

The Committee referred to the Declaration of Helsinki's statement which states informed consent should be given for the purposes of research studies either by the participant or

the participant’s legal representative. It was requested that the parents’ decision not to opt out of the study be formally documented by the research team.

The applicant agreed.

The Committee asked about the timeframe available for parents to opt out of the trial.

The applicant said this would be broadly defined by the baby’s health and there would be plenty of opportunity to opt out. It is expected the initial approach would be made to the parents by the senior clinician on the unit within 24 hours of the baby being admitted and revisited at the next discussion.

It was confirmed that if at any point the parents wish to opt out of the study the electronic system used would automatically remove the baby from the randomisation process.

<u>Suitability of supporting information</u>

The Committee asked for the details of PALS to be added to the Patient Information Sheet for information.

The applicant agreed and said that the research nurses in the units would also be familiar with the study and able to offer information to parents.

The Committee asked whether the applicant has considered adding further information about necrotising enterocolitis.

The applicant replied that this had been considered but had been decided against as they did not want to worry parents unnecessarily.

The Committee asked for the last sentence of the ‘Are there any benefits to my baby?’ section of the Patient Information Sheet to be removed as it may be seen to be coercive by suggesting that not taking part in the trial increases the risk to the baby.

The applicant told the Committee there was no wish to coerce parents but the Hawthorne Effect had been clearly noted in other trials and that it was important that this was acknowledged.

The Committee asked for the last sentence of the section to be changed to ‘taking part in a research study may confer non-specific benefits.’

Documents reviewed

The documents reviewed at the meeting were:

Document	*Version*	*Date*
Covering letter on headed paper		05 September 2014
Evidence of Sponsor insurance or indemnity (non NHS Sponsors only)		05 September 2014
IRAS Checklist XML [Checklist_10092014]		10 September 2014
Letter from sponsor		05 September 2014

Participant information sheet (PIS)	1.3	02 September 2014
REC Application Form [REC_Form_10092014]		10 September 2014
Research protocol or project proposal	1.3	11 August 2014
Summary CV for Chief Investigator (CI)	1	05 September 2014

Membership of the Committee

The members of the Committee who were present at the meeting are listed on the attached sheet

Statement of compliance

The Committee is constituted in accordance with the Governance Arrangements for Research Ethics Committees and complies fully with the Standard Operating Procedures for Research Ethics Committees in the UK.

Please quote this number on all correspondence

Yours sincerely

Chair

Email:

Enclosures: *List of names and professions of members who were present at the meeting and those who submitted written comments.*

Copy to:

Attendance at Committee meeting

Committee Members:

Name	*Profession*	*Present*	*Notes*
	Dentist	Yes	
	Senior Biomedical Scientist	Yes	
	Solicitor (non-practising)	No	
	Retired Head Teacher	Yes	
	Social Scientist	Yes	
	Assistant Manager, Local Authority - Retired	Yes	
	Midwife	No	
	Consultant Paediatrician	Yes	
	Consultant Physician - Chair	Yes	
	Consultant in Pain Management - Vice Chair	Yes	
	Chartered Engineer - Retired	Yes	
	REC Manager	Yes	
	Head of Radiology, Solihull Hospital	No	

Imperial College London

Section of Neonatal Medicine
Imperial College London

4th Floor, Lift Bank D
Chelsea and Westminster Hospital
369 Fulham Road
London, SW10 9NH
Tel: +44 (0)203 3153519
Fax: +44 (0)203 3157998

christopher.gale@imperial.ac.uk

13th October 2014

Dr Christopher Gale MBBS MSc PhD MRCPCH

Dear

**Study title:**	**The WHEAT trial: With Holding Enteral feeding Around packed red cell Transfusions in preterm neonates, a multicentre, superiority, randomised registry trial**
**REC reference:**	
**Protocol number:**	
**IRAS project ID:**	

Thank you for taking the time to review the WHEAT trial. Please find responses to your requests for further information detailed below. Changes to the PIS are highlighted in the enclosed copy:

1. Please formally document the parents' decision to not opt out of the study.
We confirm that the parents' decision not to opt out of the WHEAT trial will be recorded electronically in the electronic health record. The electronic health record will require the member of the clinical team who has spoken to the parents 1) to confirm that both the WHEAT trial and opt-out consent have been explained, and 2) whether the parents have chosen to opt-out of their baby participating in the WHEAT trial.

2. Please remove the last sentence of the 'Are there any benefits to my baby?' section of the Patient Information and replace with 'taking part in a research study may confer non-specific benefits.'
This has been changed on the PIS.

3. Please add PALS contact details to the Patient Information Sheet for information.
This has been added to the PIS as follows: "You can also discuss this study with the Patient Advice and Liaison Services (PALS): [telephone number]."

I hope these responses provide sufficient clarification, please do not hesitate to contact us if you require any further information.

Documents attached:

Document	Version	Date
Participant Information Sheet	1.4	13 October 2014

Yours Sincerely,

Dr Chris Gale
NIHR Clinical Lecturer in Paediatrics

NHS
Health Research Authority

16 October 2014

Dr Chris Gale
NIHR Clinical Lecturer
Imperial College London
Section of Academic Neonatal Medicine,
Imperial College London, Chelsea and Westminster Campus,
369 Fulham Road, London
SW10 9NH]

Dear Dr Gale

**Study title:**	**The WHEAT trial: WithHolding Enteral feeding Around packed red cell Transfusions in preterm neonates, a multicentre, superiority, randomised registry trial**
**REC reference:**	
**Protocol number:**	
**IRAS project ID:**	

Thank you for your letter dated 13October 2014, responding to the Committee's request for further information on the above research.

The further information has been considered on behalf of the Committee by the Chair.

We plan to publish your research summary wording for the above study on the HRA website, together with your contact details. Publication will be no earlier than three months from the date of this opinion letter. Should you wish to provide a substitute contact point, require further information, or wish to make a request to postpone publication, please contact the REC Manager.

Confirmation of ethical opinion

On behalf of the Committee, I am pleased to confirm a favourable ethical opinion for the above research on the basis described in the application form, protocol and supporting documentation, subject to the conditions specified below.

Conditions of the favourable opinion

The favourable opinion is subject to the following conditions being met prior to the start of the study.

Management permission or approval must be obtained from each host organisation prior to the start of the study at the site concerned.

Management permission ("R&D approval") should be sought from all NHS organisations involved in the study in accordance with NHS research governance arrangements.

Guidance on applying for NHS permission for research is available in the Integrated Research Application System or at http://www.rdforum.nhs.uk.

Where a NHS organisation's role in the study is limited to identifying and referring potential participants to research sites ("participant identification centre"), guidance should be sought from the R&D office on the information it requires to give permission for this activity.

For non-NHS sites, site management permission should be obtained in accordance with the procedures of the relevant host organisation.

Sponsors are not required to notify the Committee of approvals from host organisations

Registration of Clinical Trials

All clinical trials (defined as the first four categories on the IRAS filter page) must be registered on a publically accessible database within 6 weeks of recruitment of the first participant (for medical device studies, within the timeline determined by the current registration and publication trees).

There is no requirement to separately notify the REC but you should do so at the earliest opportunity e.g when submitting an amendment. We will audit the registration details as part of the annual progress reporting process.

To ensure transparency in research, we strongly recommend that all research is registered but for non clinical trials this is not currently mandatory.

If a sponsor wishes to contest the need for registration they should contact, the HRA does not, however, expect exceptions to be made. Guidance on where to register is provided within IRAS.

It is the responsibility of the sponsor to ensure that all the conditions are complied with before the start of the study or its initiation at a particular site (as applicable).

Ethical review of research sites

NHS sites

The favourable opinion applies to all NHS sites taking part in the study, subject to management

permission being obtained from the NHS/HSC R&D office prior to the start of the study (see "Conditions of the favourable opinion" above).

Approved documents

The final list of documents reviewed and approved by the Committee is as follows:

Document	*Version*	*Date*
Covering letter on headed paper		13 October 2014
Evidence of Sponsor insurance or indemnity (non NHS Sponsors only)		05 September 2014
IRAS Checklist XML [Checklist_10092014]		10 September 2014
Letter from sponsor		05 September 2014
Participant information sheet (PIS)	1.4	13 October 2014
REC Application Form [REC_Form_10092014]		10 September 2014
Research protocol or project proposal	1.3	11 August 2014
Summary CV for Chief Investigator (CI)	1	05 September 2014

Statement of compliance

The Committee is constituted in accordance with the Governance Arrangements for Research Ethics Committees and complies fully with the Standard Operating Procedures for Research Ethics Committees in the UK.

After ethical review

Reporting requirements

The attached document *"After ethical review – guidance for researchers"* gives detailed guidance on reporting requirements for studies with a favourable opinion, including:

- Notifying substantial amendments
- Adding new sites and investigators
- Notification of serious breaches of the protocol
- Progress and safety reports
- Notifying the end of the study

The HRA website also provides guidance on these topics, which is updated in the light of changes in reporting requirements or procedures.

User Feedback

The Health Research Authority is continually striving to provide a high quality service to all applicants and sponsors. You are invited to give your view of the service you have received and the application procedure. If you wish to make your views known please use the feedback form available on the HRA website:
http://www.hra.nhs.uk/about-the-hra/governance/quality-assurance/

HRA Training

We are pleased to welcome researchers and R&D staff at our training days – see details at http://www.hra.nhs.uk/hra-training/

Please quote this number on all correspondence

With the Committee's best wishes for the success of this project.

Yours sincerely

Chair

Enclosures:	"After ethical review – guidance for researchers"

Copy to:
